# Group versus individual antenatal and first year postpartum care: Study protocol for a multi-country cluster randomized controlled trial in Kenya and Nigeria

**DOI:** 10.12688/gatesopenres.12867.2

**Published:** 2019-07-04

**Authors:** Mark M. Kabue, Lindsay Grenier, Stephanie Suhowatsky, Jaiyeola Oyetunji, Emmanuel Ugwa, Brenda Onguti, Eunice Omanga, Anthony Gichangi, Jonesmus Wambua, Charles Waka, Joseph Enne, Jennyfer Don-Aki, Mairo Ali, Maryam Buba, Jenipher Ang’aha, Daniel Iya, Elizabeth Washika, Diwakar Mohan, Jeffrey M. Smith

**Affiliations:** 1 Jhpiego, Baltimore, Maryland, USA; 2 Jhpiego, Nigeria, Abuja, FCT, Nigeria; 3 Jhpiego, Kenya, Nairobi, Kenya; 4 Nasarawa State Ministry of Health, Akwanga, Nasarawa, Nigeria; 5 Ministry of Health, Nairobi, Kenya; 6 Johns Hopkins Bloomberg School of Public Health, Baltimore, Maryland, USA

**Keywords:** Group antenatal care, facility-based delivery, postpartum family planning, experience of care, self-efficacy, health literacy, Kenya, Nigeria

## Abstract

**Background:** Antenatal care (ANC) in many low- and middle-income countries is under-utilized and of sub-optimal quality. Group ANC (G-ANC) is an intervention designed to improve the experience and provision of ANC for groups of women (cohorts) at similar stages of pregnancy.

**Methods:** A two-arm, two-phase, cluster randomized controlled trial (cRCT) (non-blinded) is being conducted in Kenya and Nigeria. Public health facilities were matched and randomized to either standard individual ANC (control) or G-ANC (intervention) prior to enrollment. Participants include pregnant women attending first ANC at gestational age <24 weeks, health care providers, and sub-national health managers. Enrollment ended in June 2017 for both countries. In the intervention arm, pregnant women are assigned to cohorts at first ANC visit and receive subsequent care together during five meetings facilitated by a health care provider (Phase 1). After birth, the same cohorts meet four times over 12 months with their babies (Phase 2). Data collection was performed through surveys, clinical data extraction, focus group discussions, and in-depth interviews. Phase 1 data collection ended in January 2018 and Phase 2 concludes in November 2018. Intention-to-treat analysis will be used to evaluate primary outcomes for Phases 1 and 2: health facility delivery and use of a modern method of family planning at 12 months postpartum, respectively. Data analysis and reporting of results will be consistent with norms for cRCTs. General estimating equation models that account for clustering will be employed for primary outcome analyzes.

**Results:** Overall 1,075 and 1,013 pregnant women were enrolled in Nigeria and Kenya, respectively. Final study results will be available in February 2019.

**Conclusions:** This is the first cRCT on G-ANC in Africa. It is among the first to examine the effects of continuing group care through the first year postpartum.

**Registration**: Pan African Clinical Trials Registry PACTR201706002254227 May 02, 2017

## Background and study rationale

The primary purpose of antenatal care (ANC) is to help women have a healthy pregnancy and safe delivery ^1^. Through ANC, health care providers can identify emergent complications of pregnancy, but ANC also serves as a primary health care platform for identification of underlying chronic health issues and support for healthy behaviors and preventive measures, such as oral iron supplementation, use of long-lasting insecticidal nets (LLIN), tetanus immunization, and counseling for postpartum family planning (PPFP) ^2^. ANC often serves as an entry point for further interaction with the health system. Both multiple ANC visits and high-quality ANC contacts are positively associated with facility delivery and skilled birth attendance ^3– 5^.

Current service delivery models in low- and middle-income countries (LMICs), where the greatest proportion of maternal, perinatal, and newborn deaths occur, may not allow for optimal provision of all curative, preventive, and promotive services ^6– 8^. Globally, according to UNICEF, 86% of pregnant women attend one antenatal care visit (ANC1) with a skilled provider, and 62% attend four or more visits (ANC4). Inadequate ANC attendance can partially be explained by poor provision and experiences of care ^9– 12^. Most women do not receive the full range of recommended ANC services ^13– 15^. Women report long wait times, unpleasant experience with providers, lack of provider attentiveness, lack of privacy, and limited provision of services, all of which result in low client satisfaction ^16– 18^. Contact with providers may be brief, as reported in Tanzania where the average contact time was 12 minutes for the first contact and 6.5 minutes for subsequent visits ^19^. Health care providers face numerous barriers to providing high-quality ANC (e.g., poorly resourced work environments, heavy workloads, long working hours), which cause stress, poor job satisfaction, and uncaring behavior toward clients ^20, 21^.

Group ANC (G-ANC) is an alternative ANC service delivery model. Where implemented, G-ANC is described and offered as an alternative to individual care when women come to their first ANC visit. Women who choose G-ANC are placed in groups, or cohorts, with other women of similar gestational age (GA) and receive subsequent ANC together during scheduled meetings facilitated by a health care provider at the health facility. Implementation and research on G-ANC to date has primarily been in high-income countries ^22, 23^. These studies found that, when compared to individual ANC, G-ANC resulted in increases in several outcomes: uptake of family planning at 6, 9, and 12 months postpartum; length of gestation; birth weight; breastfeeding initiation and duration; attendance at ANC; health literacy; and patient satisfaction; as well as decreased transmission of sexually transmitted infections during pregnancy ^24– 26^. A Cochrane review in 2015 was not conclusive on the health outcomes of the intervention, however, it found participants viewed G-ANC positively, and no negative outcomes for mothers or their babies were identified ^27^.

This evidence was considered by the World Health Organization (WHO) in the development of new ANC recommendations in 2016. Those recommendations state that G-ANC may be offered as an alternative to individual ANC in the context of rigorous research ^2^. To date, there have been a limited number of studies on the effect of G-ANC in LMICs, primarily from single site studies in Malawi, Tanzania, Nigeria, and Ghana ^28– 34^. Available results indicate acceptability and feasibility, but the single site nature of these studies limits generalizability.

We therefore designed a cluster randomized controlled trial (cRCT) to test the feasibility, acceptability, and effectiveness of G-ANC in multiple facility contexts, including a mix of urban, peri-urban, and rural sites, in two African countries. The cRCT design enables implementation in sites with lower ANC census numbers than would be necessary for an individually randomized study (i.e., twice the census would be needed to form cohorts while individually randomizing). In addition, the cluster design decreases the risk of contamination between study arms.

The study will be conducted in two phases. The primary outcome of Phase 1 is an increase in the percentage of women who have a facility-based delivery. In Phase 1, the study will test whether re-organization of care from individual ANC to G-ANC, and the corresponding emphasis on participatory care and learning, will: improve the provision and experience of ANC; empower women and health care providers; and increase health literacy—together, leading to greater practice of healthy behaviors and health service utilization among women experiencing group care. The primary outcome for Phase 2 is an increase in the use of a modern family planning (FP) method at one year after birth. In Phase 2, the study will test whether women who remain in the same cohorts and receive care in groups for the first year postpartum report improved practice of health, hygiene, and early childhood development behaviors and increased health service utilization. Outcomes are measured at the individual participant level.

## Methods

### Study design

The study is a multi-country, two-phase, cRCT implemented in Machakos and Kisumu Counties, Kenya and in Nasarawa State, Nigeria. It was designed in collaboration with the Kenyan Ministry of Health (MOH) and the Nasarawa State MOH and Primary Health Care Development Agency in Nigeria.

In each country, 20 health facilities were selected and matched in pairs by: type of health facility (e.g., health center, hospital); client caseload for first ANC visit (i.e., ANC1); location (urban, peri-urban, rural); culturally similar catchment populations; and availability of FP services. The pairs had to be either within the same state (Nigeria) or county (Kenya), but did not need to be geographically adjacent. In Kenya, 10 clusters were assigned to each of the two counties where the study took place.

The unit of randomization was the health facility, which formed the clusters. For each pair of matched health facilities, randomization (1:1) was done to the intervention (group care) or control arm (the current standard of individual care according to country guidelines). Assignment to study arms was done through a lottery using pieces of paper in a basket bearing the words “intervention” or “control”.

**Figure 1.**
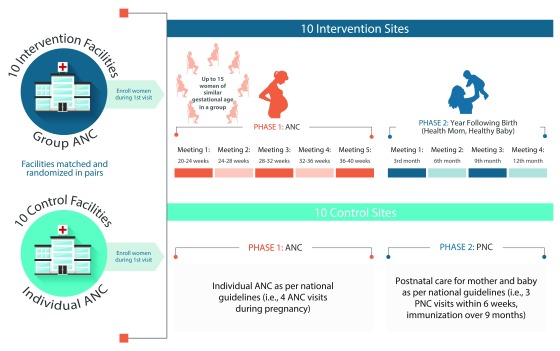
Study design. Description of randomization to intervention and control group and the intervention package for each study group.

In both study arms, all women provided written consent to participate in the study. In each health facility, 50–54 participants were consented and enrolled, along with three health care providers. For the intervention arm, four to five cohorts (groups of women) were formed per facility. Figure 1 summarizes the study design.

### Study setting

The study is being implemented in Machakos and Kisumu Counties in Kenya and in Nasarawa State, Nigeria. Countries were chosen based on interest to the funder. In-country locations were chosen in collaboration with local colleagues, based on adequate security for women to travel to ANC; functional local health system governance and structure; Jhpiego’s ability to leverage existing maternal, newborn, and child health/FP (MNCH/FP) projects to reduce implementation costs; and where possible, avoidance of other ongoing projects that could introduce bias. Table 1 presents key indicators for Kenya and Nigeria, comparing the national and state/county-level indicators in each country as appropriate ^35, 36^.

**Table 1.** Comparison of key MNCH/FP indicators for Kenya and Nigeria. Summary of key maternal newborn and child health/family planning (MNCH/FP) indicators based on DHIS2 data for Kenya (Kisumu and Machakos Counties) and Nasarawa, Nigeria.

	Kenya (DHS 2014)			Nigeria (DHS 2013)	
Indicator	National	Kisumu County	Machakos County	National	Nasarawa State
Maternal mortality ratio (per 100,000)	362	na	na	576	na
Neonatal mortality rate (per 1,000)	22	na	na	37	na
No ANC	3.9%	1.3%	1.0%	33.9%	na
Any ANC from a skilled provider	95.5%	98.4%	98.8%	60.6%	63.2%
ANC4	57.6%	na	na	51.1%	na
Median GA at first ANC	5.4 months	na	na	5.0 months	na
Birth with skilled provider	61.8%	69.2%	63.4%	38.1%	40.7%
Birth in a health facility	61.2%	69.5%	62.9%	35.8%	40.1%
Postnatal care (PNC) within 2 days (mother)	52.9%	na	na	39.6%	49.4%
PNC within 2 days (baby)	35.6%	na	na	14.0%	18.9%
Exclusive breastfeeding	61.0%	na	na	17.0%	
Contraceptive prevalence rate (modern method), currently married	53.2%	na	na	9.8%	16.3%

na = data not available—maternal mortality ratio and neonatal mortality rate are computed only at national level. DHS = Demographic and Health Survey

In 2016, Kenya had an estimated population of 45 million. Machakos and Kisumu County populations were 1.1 million and 0.96 million, respectively. While ANC attendance is nearly universal, fewer than six in ten (57.6%) pregnant women attend four or more visits during pregnancy ^35^. The facility-based delivery is 61.2% nationally, 62.9% in Machakos County, and 69.5% in Kisumu County ^35^. Nationally, 52.9% of women receive PNC within two days of giving birth ^35^. FP use among postpartum women was estimated at 36% based on analysis of 2008–2009 Kenya Demographic and Health Survey (DHS) data ^37^.

Nigeria is the most populous nation in Africa, with an estimated 198 million inhabitants. Nasarawa State has an estimated population of 2.5 million. One-third of pregnant women (33.9%) in Nigeria do not receive any ANC ^36^. Half (51.1%) receive four or more ANC visits ^36^. The facility-based delivery rate is 35.8% nationally and 40.1% in Nasarawa State ^36^. Both ANC and delivery with a skilled birth attendant coverage have stagnated ^38^. Quality of ANC is poor ^39^. PNC within two days is 39.6% for the mother and 14.0% for the newborn ^37^. FP use among postpartum women was estimated at 15% based on analysis of 2008 Nigeria DHS data ^37^.

Both countries have national policy documents that promote key evidence-based MNCH/FP interventions—at least four ANC visits and a facility-based delivery with a skilled provider ^40– 44^.

### Participants

***Eligibility for clusters.*** The intervention is multilevel with activities targeted to different participants in the selected health facilities.

**Health facilities (clusters):** A list of health facilities offering ANC and postnatal care (PNC) services in each study location was prepared by the study team, based on data from the national health information systems (HIS), which use the open source software platform District Health Information System 2 (DHIS2). Within the study areas, at the time of site selection, 551 health facilities in Nigeria and 410 in Kenya were providing ANC services. Study criteria dictate that women in group care need to be 20–24 weeks GA at the time of the first group meeting, thus a minimum ANC1 census was deemed the most important criteria to determine the ability to form cohorts. HIS data for the 12 months preceding the study was used to identify potential sites. In Nigeria, all facilities with more than 20 new ANC clients/month who initiated care at or before 20 weeks GA were considered for inclusion. GA at ANC1 was not available in the Kenya HIS. Therefore, assuming approximately 50% of women enter care < 24 weeks (participant criteria), all facilities with a minimum of 30 new ANC clients/month were considered. Members of the study team then visited all facilities to verify HIS ANC census numbers, by examining the ANC registers for the previous three months, and to verify additional selection and matching criteria. Facilities were selected in conjunction with county/state health officials and included consideration of “best match” ability as well as inclusion of urban/peri-urban/rural sites and varied levels of the health system ( Table 2).

**Table 2.** Eligibility for health facilities and participants. Description of the inclusion criteria for health facilities and participants included in the study.

Category	Inclusion Criteria
**Health facilities**	• Located in one of the selected two counties in Kenya, or Nasarawa State in Nigeria • A minimum of new ANC clients monthly: 30 clients in Kenya; 20 clients ≤ 20 weeks GA in Nigeria • Availability of community health extension workers (CHEWs), government employed facility-based staff • A minimum of two staff during all working hours • On-site availability of ANC, PNC, and FP services • Permission granted by health facility management to participate in study • Public health facility
**Pregnant women**	• Minimum age of 15 years at the time of enrollment * • Appropriate GA as best estimated by provider using combination of last menstrual period, pelvic exam, fundal height, quickening, ultrasound, and/or timing of fetal heart tones dependent on availability o Control sites: < 24 weeks GA o Intervention sites: Between 20–24 weeks GA at the time of first group meeting; and reply “yes” or “unsure” when asked if can return for first cohort meeting • Willing and able to provide a phone number through which she can be reached • Detailed locator information for follow-up (all who provide cell phone number AND also for those without cellphone but traceable). • Planning to stay in area for the next year, and not planning to be away from home (near this facility) for more than four weeks in a row at any time during pregnancy or more than three months in a row in the year after the baby is born • Willing to participate and consent to follow-up for up to 12 months post-delivery
**Health care** **providers**	• Working in a participating/selected health facility and providing ANC or PNC services • Willing to participate in the study
**Sub-national health** **managers**	• Working in the study state or county as a government official managing health activities and/or service delivery • Willing to participate in the study

*Note, In Kenya, pregnant women under the age of 18 are considered mature minors and are able to give consent for themselves, if they can demonstrate evidence of clear understanding of the requirements for the research they are consenting to participate in Kenya ^45^. In Nigeria, persons aged 13 and above can consent for themselves (i.e., without parental consent) to participate in non-therapeutic research ^46^.

***Eligibility for participants.*** Study participants include pregnant women, health care providers, and sub-national health managers. The inclusion criteria are detailed in Table 2. There were no additional exclusion criteria.

**Pregnant women**: Pregnant women 15 years and older attending ANC1 in any of the study health facilities. Additional criteria on GA and availability were applied depending on the study arm.

**Health care providers:** Providers working in the participating health facilities primarily providing ANC and PNC services.

**Sub-national health managers:** Included either government officials who work in sub-national level health management positions or health facility in-charge in study settings.

### Intervention

The intervention is provision of an alternative service delivery model that delivers care in a group, compared to the current standard of care provided individually (i.e., one-on-one between a provider and an ANC client). Women in the intervention arm were invited to attend five G-ANC meetings in addition to the first visit (Phase 1) and four Healthy Mother, Healthy Baby (HMHB) meetings (Phase 2) in the year following birth.

***Phase 1: G-ANC.*** At the first ANC visit (ANC1), pregnant women are assessed for GA. If eligible, women in the intervention group are offered the option of G-ANC for their subsequent ANC. Interested women are placed in a cohort of 8–15 other women of similar GA, all of whom will be 20–24 weeks pregnant at the time of a pre-scheduled cohort start date (Meeting 1). Thereafter, they attend subsequent routine visits together every month as G-ANC meetings. Five total scheduled meetings are held and recorded as ANC contacts, each lasting approximately two hours.

In each intervention facility, two ANC providers (nurses or midwives) and one CHEW were trained as G-ANC facilitators. In both countries, CHEWs are facility-based and provide some level of clinical care. Two facilitators were assigned to each cohort and were advised to co-facilitate meetings for that group throughout the intervention.

Prior to initiating the study, inputs were made to all facilities, in both arms, in the same quantities, to ensure comparable capacity for service delivery ( Table 3). The inputs included a select set of ANC commodities that would ordinarily be provided through the public sector health system. In addition, a one- to two-day refresher training on ANC and PPFP counseling was provided for all ANC providers at control sites and for G-ANC facilitators at intervention sites. Intervention facilities received additional G-ANC specific inputs as outlined in Table 3.

**Table 3.** Inputs provided to control and intervention clusters. Description of the elements of support provided to the two study arms in accordance to the group assignment.

Input	Intervention Clusters (Group Care)	Control Clusters (Current Standard Individual Care)
**Provision of key ANC commodities:** LLIN; intermittent preventive treatment in pregnancy for malaria using sulfadoxine-pyrimethamine (IPTp-SP); iron and low dose folic acid (IFA) tablets; HIV test kits; syphilis test kits; urine dipsticks; three CRADLE Vital Sign Alert automatic blood pressure devices per facility	X	X
**One- to two-day ANC and PPFP counseling update for ANC service providers**	X	X
**Five-day PPFP clinical training (inclusive of postpartum intrauterine device [IUD] and implant** **insertion and removal) followed by clinical practice**	X	X
**Five-day G-ANC training for facilitators** in logistical planning and facilitation of G-ANC for two ANC providers and one CHEW per facility	X	
**Four-day HMHB training for facilitators**	X	
**G-ANC and HMHB specific materials:** facilitator’s guide, large illustration cards, self-assessment cards and markers, women’s take-home booklets, longitudinal group cohort registers, privacy screens, chairs	X	
**Tea/snacks during group meetings**	X	
**Ongoing mentoring** specific to group care by study staff (i.e., goal of attending 80% of meetings of first two cohorts)	X	
**Debriefing tools,** including fidelity and facilitation skills checklists to be used by mentors and as self-evaluation by G-ANC facilitators	X	
**Mobile phone credit (air time) for reminder calls to women prior to group meetings**	X	

* Where inputs identical, they were provided during similar time periods and in equal quantities

Clinical care in both study arms was provided according to national clinical standards and guidelines ^47– 50^. No changes were made to clinical care protocols or content in any health facility. No additional activities were undertaken in either arm to improve the quality of clinical care (e.g., support for management of complications; integration of PPFP in postnatal wards). The curriculum content was divided into five sessions during antenatal care visits and four sessions for antenatal visits in the intervention arm ( Table 4).

**Table 4.** G-ANC and HMHB meeting content. Summary of activities and content covered during each group meeting before and after delivery.

Meeting	Timing and Content	
**G-ANC**		
**Meeting 1**	**20–24 weeks GA** • Introduction to group care • Preventing problems in pregnancy	
**Meeting 2**	**24–28 weeks GA** • Recognizing problems in pregnancy • Partner negotiation and communication (e.g., sexually transmitted infection prevention)	
**Meeting 3**	**28–32 weeks GA: Partners encouraged to attend** (if group agrees) • Birth planning • Healthy timing and spacing of pregnancy and PPFP method options	
**Meeting 4**	**32–36 weeks GA** • Review individual birth plans and PPFP intent • Preventing problems after birth (maternal and newborn) • Labor signs	
**Meeting 5**	**36–40 weeks GA** • What to expect in labor and birth • Recognizing postpartum problems (maternal and newborn)	
HMHB		
**Meeting 1**	**3 months after** **delivery**	Clinical care is offered to babies and mothers at every meeting. Each meeting covers similar topics with content specific to the age of the baby: **Raising Baby** • Understand your baby: Developmental milestones; responsive and positive parenting • Maximize love and learning: Four age-appropriate activities for early childhood development are demonstrated and practiced, including one picture-based, early literacy activity, in each session • Feed and protect: Three to four specific topics covering: nutrition; water, sanitation, and hygiene practices; accident prevention; and well-baby care (e.g., immunizations, vitamin A, use of oral rehydration salts) **Taking Care of Yourself** • Self-care activities: Recognizing postpartum depression; postpartum exercise/pelvic floor rehabilitation; getting rest; asking for help • PPFP: Sharing experiences and knowledge; partner challenges; management of side effects
**Meeting 2**	**6 months after** **delivery**	
**Meeting 3**	**9 months after** **delivery**	
**Meeting 4**	**12 months after** **delivery**	

At the first meeting, women agreed on what day and time they would like subsequent meetings to be and whether husbands should be invited to Meeting 3 ( Table 4). Women who have or develop complications requiring additional individual care are encouraged to continue attending group meetings.

Longitudinal group care registers were introduced in the intervention facilities and used for each cohort. They serve a dual purpose of data collection and reinforcement of key actions by providers and women, such as taking IFA supplements, sleeping under a LLIN, birth planning, and making plans regarding healthy timing and spacing of pregnancy.

***Phase 2: HMHB.*** Healthy Mother, Healthy Baby meetings extend group care into the first year after birth ( Table 4). The same cohorts continue to meet, with their babies. Meetings are scheduled every three months (total of four HMHB meetings). Because of the staggered timing of births for women in a cohort, HMHB meetings are not designed to replace standard PNC visits (i.e., four visits in the first six weeks) as recommended by WHO and included in national guidelines of both countries ^51^. Women in both arms are encouraged to follow country guidelines regarding immediate PNC, as well as national immunization schedules (when they do not correspond to scheduled group meetings).

In 2017, the G-ANC facilitators were trained to facilitate HMHB meetings and remained attached to the same cohorts in the intervention arm. Prior to the first cohort delivering, two to three providers from each facility in both arms will receive clinical training in PPFP methods.

***Meeting frameworks and key content.*** The meeting frameworks for G-ANC and HMHB were designed to incorporate three key components of group care: 1) individual clinical assessment; 2) participatory, facilitated learning, and; 3) peer support. The frameworks were informed by evidence-based learning principles, social behavior change and communication principles, and social support theories. They were designed to include highly facilitative, nonhierarchical, patient-centered participatory approaches with attention to education and literacy levels and community cultural norms.

Clinical assessments include self-assessments (e.g., blood pressure, weight, and danger signs), and brief, individual, private assessments with the provider, behind screens within the group meeting space. To address the main topic of each meeting, large illustrations cards are used to facilitate discussion around key actions or danger signs. Questions are posed to the group while viewing each illustration to: promote sharing of knowledge, examine cause and effect, agree on actions to take, discuss barriers and solutions, and agree to share information with others. The women receive take-home booklets with small versions of the same illustrations to use as memory aids and resource guides for sharing information with family and friends. Other meeting components are designed to specifically support group identity and cohesion; promote empowerment, self-efficacy, and agency; and promote self-reflection and planned action.

### Training and mentoring

Prior to introducing G-ANC in the intervention sites, 30 facilitators in each country were trained together by study staff in a five-day workshop offsite using the G-ANC framework and materials developed for this study. They were also trained to use self-reflective quality assurance and improvement tools, which included a scorecard for facilitation skills, a fidelity checklist, and prompts for identifying strengths and weaknesses and things to change or try at the next meeting.

Program officers (POs) were employed by the study to mentor facilitators, oversee research assistants, and support daily implementation of the study at all study facilities in their defined geographic area. POs are nurse-midwives who speak the local languages. POs aimed to attend 80% of the first three cohorts at each facility to provide mentoring on the G-ANC model and feedback on facilitation skills. Debriefs immediately after group meetings followed a structured coaching outline, incorporating reflections from the G-ANC facilitators.

### Outcomes

Different outcomes are targeted for the different phases of the trial. The primary outcome for Phase 1 is the proportion of facility-based delivery among women enrolled in intervention (G-ANC) arm versus control (standard individual care) arm. For Phase 2, the main outcome is the proportion of women using a modern FP method (defined for study purposes as condoms, oral contraceptives, injectables, implants, IUDs, and sterilization) at 12 months postpartum, comparing the two study arms.

Secondary outcomes include additional measures of: service utilization; provision of quality ANC; experience of care by women and providers (measures of satisfaction and positive experience); MNCH/FP-related health literacy and uptake of healthy behaviors (e.g., sleeping under a LLINs, taking IFA supplements, early initiation of breastfeeding, knowledge of danger signs, knowledge of lactational amenorrhea method criteria); and improvement in agency, self-efficacy, and empowerment, specifically related to birth planning and PPFP ( Table 5).

**Table 5.** Research questions and outcomes by study phase. Study research questions, outcomes and data collection tools stratified by study phase (1&2).

Research Questions	Secondary Research Questions
**Outcomes are cross-referenced with the data collection tools used to answer the question (in parentheses)**	
**PHASE 1: G-ANC**	
1. Are women in G-ANC arm more likely to deliver in a health facility, compared to women who are allocated to standard individual ANC? ( 4, 5) **Phase 1 primary outcome:** Health facility delivery	Are women allocated to G-ANC more likely than those allocated to standard individual care to: 1. Attend 4 or more ANC visits ( 4, 5) 2. Receive better quality provision of care as measured by: a. Total average doses of IPTp ( 5) b. Frequency of danger sign assessment at every visit ( 4) c. Total percentage of ANC visits with blood pressure recorded ( 5) d. Frequency of patients who received counseling on 12 key topics ( 4) 3. Report greater satisfaction and improved experience of care ( 4, 7) 4. Increase health literacy and practice of healthy behaviors ( 4, 7) 5. Report greater agency, self-efficacy, and empowerment, as measured by: a. Completion of birth plans ( 4, 5)) b. Choice of PPFP method prior to delivery ( 4, 5) Do providers report improved satisfaction and enjoyment and prefer providing G-ANC to standard individual care? ( 1, 3, 6)
**PHASE 2: HMHB**	
2. Are women in G-ANC more likely to be using a modern FP method at one year postpartum, compared to women allocated to standard individual care? ( 9) **Phase 2 primary outcome:** Use of a modern FP method at 12 months postpartum	At one year postpartum, are women allocated to G-ANC more likely than those allocated to standard individual care to: 1. Have attended more clinic visits, resulting in a. Higher average number of growth monitoring measurements ( 9, 10) b. Fully vaccinated infants ( 10) 2. Exhibit increased health literacy and practice of healthy behaviors, including: ( 9) a. Creation and use of hand washing station b. Exclusive breastfeeding until 6 months c. Earlier introduction of animal-based proteins d. Practice of select early childhood development activities by multiple members of household 3. Report increased couple communication and agreement in relation to healthy timing and spacing of pregnancy ( 9, 11, 12) 4. Report greater satisfaction and improved experience of care ( 9, 11, 12) 5. Report greater agency, self-efficacy, and empowerment in part measured by: a. PPFP self-efficacy scale ( 9) Do health care providers report improved satisfaction and enjoyment and prefer providing HMHB meetings to standard individual care in the first year postpartum? ( 11)

**Numbered data collection tools:** 1: ANC baseline survey; 2: survey of recently delivered women; 3: data extraction tool (from G-ANC registers and health facility records); 4: focus group discussion (FGD) guide for pregnant women in the intervention group at completion of G-ANC meetings; 5: in-depth interview (IDI) guide for pregnant women, at completion of group care before delivery; 6: IDI guide for health care providers (after completion of group care before delivery); 7: IDI guide for sub-national health managers (after completion of group care before delivery); 8: women's survey at one year after birth; 9: data extraction tool (from HMHB registers and health facility records); 10: IDI guide for women, at completion of group care, approximately 12 months after delivery; 11: IDI guide for health care providers (after completion of group care, approximately 12 months after delivery); 12: IDI guide for sub-national health managers (after completion of group care)

### Recruitment and consent

All ANC service providers at all study sites were oriented to the study prior to initiation of enrollment. Enrollment ended January 2017 in Nigeria and June 2017 in Kenya. One research assistant (RA) was hired and assigned to each health facility in both study arms. Standardization of study procedures was facilitated by a study manual ( Supplementary File 1), which was provided to all study staff.

***Health care providers.*** Study staff approached eligible ANC and PNC health care providers in participating health facilities, explained the purpose of the study, and obtained written consent prior to starting study enrollment. Health care providers were not blinded to group assignment. In intervention sites, two health care providers and one CHEW were recruited. In control sites, all health care providers who provide ANC services were included.

***Pregnant women.*** Research assistants were stationed at their assigned site during ANC service provision. ANC providers documented GA, methods used to calculate GA, and estimated date of delivery for every new ANC client. Those < 24 weeks GA were referred by the ANC provider to speak to the RA for further eligibility screening. No personal identifiers were retained for clients > 24 weeks GA, or those who did not ultimately consent.

Each RA conducted screening and consent in a location in the facility that allowed for audio and visual privacy. Women were accorded an opportunity to hear more about the study through use of a client study information sheet and screening was done in the most appropriate of five languages (English; Kiswahili, Kamba, and Luo in Kenya; English and Hausa in Nigeria). After confirming eligibility, the RA then collected basic demographic data. No personal identifier information was collected prior to the consent process.

In the intervention arm, assignment to a cohort was done based on GA at the time of the pre-determined cohort start date (i.e., Meeting 1). Eligible women unable to attend the first meeting were not consented or assigned to a group; they were advised to continue with standard individual ANC. If a woman was eligible for the study, but there were no open cohorts (e.g., the appropriate cohort was full), she was advised to continue with standard individual ANC. In the control arm, all eligible women who were willing to participate were consented. Written consents were obtained for all study participants in their language of choice (from the five options). A baseline survey was administered immediately after the consent process.

If it was not possible to recruit a minimum of eight women at least three days before the scheduled cohort start date, the cohort was cancelled, and the assigned women re-evaluated for eligibility into another cohort. Women who become ineligible to participate due to cohort cancellation were advised to continue with standard individual ANC. Their data were then coded to reflect cohort cancellation and retained for reporting purposes. However, they were removed from the study sample, and no additional follow-up was done for these women.

***Sub-national health managers and health facility in-charge.*** Sub-national health managers (i.e., county-level in Kenya; state-level in Nigeria) responsible for overseeing reproductive health programs were recruited by study staff via phone at the end of Phase 1 to participate in the in-depth interviews (IDIs). Priority was given to managers known to have had exposure to G-ANC at the end of Phase 1. An appointment for a face-to-face interview was scheduled with each respondent.

### Sample size

Sample size calculations were done separately for the two countries based on the assumptions that were relevant to the local situation. In each country, the goal was to ensure that the study was adequately powered and each cluster could recruit at least four cohorts.

**Quantitative:** The study is powered to detect a 15 percentage point difference in the primary outcome of interest—proportion of deliveries in a health facility—between the intervention and control arms at the end of Phase 1. For the purpose of sample size calculations, DHS data were used to estimate the average proportions of facility-based delivery in the study regions: 61% for Kenya and 40% for Nasarawa State in Nigeria respectively ^35, 36^.

Intra-class correlation was estimated at 0.03 for both countries, which will lead to a design effect of 2.45, assuming an average of 30 new ANC clients per month in Kenya and 20 new ANC clients per month in Nigeria for each of the 20 health facilities in each country (i.e., 10 facilities per study arm). After factoring an attrition rate of 20%, sample sizes of 1,026 and 1,076 women were determined for Kenya and Nigeria, respectively. The study is powered at 85% for Kenya and 80% for Nigeria to maintain enrollment sufficient for four cohorts per health facility. Three health care providers were recruited per facility (120 total), including only those who routinely provide ANC services.

For Phase 2, we calculated the change in proportion of women who are using a modern FP method at 12 months after birth that would be detectable given the sample size enrolled for Phase 1. Current estimates of PPFP use from the most recent DHS were not available for Kenya or Nigeria. For Kenya, the rate therefore was estimated at 65%, based on rates of PPFP one year after birth in the 2014 endline results from the Kenya Urban Reproductive Health Initiative ^52^. For Nigeria, sample size estimation used the contraceptive prevalence rate (modern methods) of 16% for Nasarawa State from the 2013 Nigeria DHS. Based on assumed 20% attrition in Phase 1 and an additional 20% attrition by the end of Phase 2, the study will be able to detect a 10-percentage point difference in PPFP use at 12 months in Kenya and nine percentage point difference in Nigeria between study arms.

**Qualitative:** Approximately 15 IDIs and six focus group discussions (FGDs) will be conducted per phase in each country. In sampling for both IDIs and FGDs, the aim is to draw a sample reflective of the full range of responses to the G-ANC intervention model. For Phase 1, the IDIs and FGDs were conducted with women and health care providers both near and far from district referral hospitals to capture variation in challenges related to training, logistics, supervision, transport, and care seeking based on location.

### Data collection

The study uses a mixed methods data collection approach (quantitative and qualitative). The data sources are client and service provider surveys; data extraction from facility records, G-ANC registers, patient held records; and FGDs and IDIs with providers, women, and sub-national health managers. Phase 1 data collection ended January 2018 in both countries. Phase 2 data collection concludes in October 2018. The two phases of the study use similar data collection approaches. Table 6 summarizes data collection by phase. Each participant was assigned a unique study ID that is used to link all the data from the same individual in the database. Qualitative data will be used to complement the quantitative data through more in-depth exploration of selected thematic areas.

**Table 6.** Data collection timing and tools, by trial phase. Summary of the timing of when each data collection tools stratified by study phase (1&2).

DATA COLLECTION: TIMING	Intervention health facilities		Control health facilities		Intervention only	
	Tool #		Tool #		Tool #	
	Health care providers	Pregnant women	Health care providers	Pregnant women	Sub-national health managers
**BASELINE**					
**Baseline survey**: at enrollment	1	2	1	2	**-**
**PHASE 1: G-ANC**					
**Phase 1 surveys**: providers after last cohort finishes; women 3–6 weeks postpartum (PP)	3	4	-	4	-
**Data extraction** from G-ANC registers, women-held records and facility records: extracted concurrently with surveys	-	5	-	5	-
**FGDs and IDIs**: toward completion of Phase 1, subset of PP women	6	7	-	-	6
**PHASE 2: HMHB**					
**Phase 2 surveys**: providers after last cohort finishes; women at 12 months PP or after their last HMHB meeting	8	9	-	9	-
**Data extraction** from HMHB registers, women-held records and facility records: extracted concurrently with surveys	-	10	-	10	-
**FGDs and IDIs** at completion of group care during year following birth (n=subset, to saturation)	11	12	-	-	11

**Numbered data collection tools:** 1: ANC baseline survey; 2: survey of recently delivered women; 3: data extraction tool (from G-ANC registers and health facility records); 4: FGD guide for pregnant women in the intervention group at completion of G-ANC meetings; 5: IDI guide for pregnant women, at completion of group care before delivery; 6: IDI guide for health care providers (after completion of group care before delivery); 7: IDI guide for sub-national health managers (after completion of group care before delivery); 8: women's survey at one year after birth; 9: data extraction tool (from HMHB registers and health facility records); 10: IDI guide for women, at completion of group care, approximately 12 months after delivery; 11: IDI guide for health care providers (after completion of group care, approximately 12 months after delivery); 12: IDI guide for sub-national health managers and health facility in-charges (after completion of group care)

Study data were collected and managed using electronic data capture tools hosted at Jhpiego through the REDCap ^TM^ (Research Electronic Data Capture) platform. REDCap is a secure, web-based application designed to support data capture for research studies.

***Quantitative data***

**Surveys ( Supplementary File 1)**: Baseline surveys were completed at the time of enrollment, during the woman’s first ANC visit. Face-to-face interviews with recently delivered women (RDW) were conducted at the end of Phase 1 (3–6 weeks post-delivery) to gather information regarding the women’s ANC and delivery experience. For the RDW survey, RAs contacted women by mobile phone to schedule a time to visit them at home to conduct the survey, based on the expected day of delivery, from REDCap ^TM^. RAs made up to five attempts to contact the women on different days of the week at different times of the day over a two-week period to schedule an interview. If phone numbers were not functional or women were not successfully contacted after 5 attempts, physical tracing was done using the information provided at enrollment. Detailed locator information was obtained and authority for home visits at consent stage for all participants who were enrolled.

Women are also scheduled to be surveyed after Phase 2 to collect data on MNCH/FP care received in the 12 months after birth. Data are captured electronically using tablets loaded with REDCap ^TM^ software. During both phases, a participant is determined to be lost to follow-up if both phone and physical tracing efforts are unsuccessful.

**Data extraction:** Per the study protocol RAs extracted data from different sources including: longitudinal group care registers; women-held records (Kenya only); and other relevant facility-based health records. The data were captured electronically through REDCap.

***Qualitative data.*** Focus group discussions and IDIs were scheduled and conducted in 2017 toward the end of Phase 1 and will be conducted toward the end of Phase 2 in 2018. An international qualitative research team lead, trained, and oversaw local qualitative researchers familiar with MNCH/FP-related issues. The FGDs and IDIs were audio-recorded and then transcribed after obtaining consent from the study participants.

**Focus group discussions**: FGDs for health care providers explored attitudes toward providing group care; perceived changes in their communication and relationship with patients; changes in empowerment, self-efficacy, and satisfaction related to their perceived ability to do their job well; sustainability of group care; suggested changes to the model and logistics required to offer group care; and perceived effects of group care on colleagues and clients. For the women attending group care, FGDs explored: satisfaction with care; perceived changes in self-efficacy or empowerment; health literacy; improved communication and trust with providers and health system as a whole; factors in uptake and continuation of PPFP; and overall impression of the group care model. Phase 2 FGDs will focus on similar topics; with a further focus on the ways HMHB influenced beliefs and actions around PPFP, nutrition and early childhood development practices, couple communication, and development of social capital.

**In-depth interviews:** IDIs were conducted with a select group of women on pre-determined topics that might have been difficult to explore through FGDs. To explore how problem recognition and care seeking was influenced by G-ANC, criteria for selection included: non-responders, (i.e., women who only came to one or two meetings [those whose barriers we don’t understand]); women who attended G-ANC meetings but didn’t deliver in a facility; and women who experienced complications during pregnancy. The IDIs are conducted until saturation of new information is reached. In addition, sub-national health managers were also selected for IDIs. Phase 2 IDIs will follow a similar pattern, including inclusion of those who came to meetings but did not accept PPFP at one year postpartum and exploration of care seeking for sick infants.

### Data quality assurance

Data collection tools were pretested and revised as necessary before use. A data management plan for the study is in place to assure data quality at all stages. The key components of the plan include the use of logic checks and skip patterns; verification of completeness of each record by POs prior to server upload; verification by POs of a random 20% of study subjects with submitted survey data that surveys were in fact administered to them; and real-time data review and monitoring by study investigators based in Kenya, Nigeria, and USA on the REDCap ^TM^ cloud platform. In Nigeria, where women tend to deliver at the same facility where they receive ANC, POs also cross-checked 20% of self-reported facility-based deliveries with facility records. This verification was not possible in Kenya where women commonly seek ANC and delivery care at different facilities, including the private sector.

### Anticipated and unanticipated events

We have no reason to believe that the intervention will cause harm. However, there might be some risk of embarrassment to women who share their personal information with group members; such information might leak outside the group. Additionally, the study team is tracking occurrence of anticipated and unanticipated events as outlined in the research plan. These include miscarriages, stillbirths, neonatal deaths, and maternal deaths.

### Data analysis

Data analysis and the reporting of results for this study will be conducted in accordance with norms for analyzing cluster randomized trials as described in the Consolidated Standards of Reporting Trials guidelines ^53^.

***Quantitative data analysis.*** Descriptive analysis of quantitative variables will be done using measures of central tendency (mean, median), measures of dispersion (range, standard deviation), and proportions (frequencies, percentages) as appropriate. Next, differences between the two study arms will be assessed using chi square test. Potential confounding factors, such as the women’s age will be assessed.

The primary outcome analysis will be done based on the intention-to-treat analysis. Participant’s data will be analyzed based on the groups to which they were randomized to compare the proportions of facility-based deliveries between the two study arms. The endpoints will be analyzed using a generalized estimating equations multivariable logistic regression model. The model will adjust the estimates for imbalances in the baseline characteristics.

Secondary analysis will be conducted to explore the uptake of services offered at ANC and during the 12-month post-delivery period, such as FP and child immunization. All statistical analysis will be performed using the R statistical software ^54^. Subgroup analysis will be conducted for specific subgroups to determine if there was a differential effect of the interventions. The amount of missing data will be assessed for each variable and overall for the sample. If more than 5% of data are missing, multiple imputation methods will be used, assuming data are missing at random ^55^.

***Qualitative data analysis.*** Following each audio recording, either the moderator or an official transcriber familiar with local language and English will transcribe from listening to the audio recording. The transcription will be done, and personal identifiers redacted. The transcribed data will be checked for quality by study staff conversant in both English and local languages used. Transcriptions will be in English. Analysis will be ongoing to inform decisions on saturation and thus there is no need to wait until all qualitative data are collected. Coding of textual passages will be done in Atlas-ti or other similar qualitative data analysis software. Two to three qualitative data analysts will code the textual information on health topics. The emerging themes will be summarized in tables and other appropriate formats. Audio recordings will be retained until when transcriptions analysis are complete, then destroyed. The electronic versions will be stored by Jhpiego in secure servers along with the electronic quantitative data files.

### Dissemination of study results

The study team plans to disseminate findings among national and sub-national stakeholders through in-country dissemination events and globally through peer-reviewed journal manuscripts and international conference presentations. About 10 publications, including conference abstracts and journal manuscripts, are planned. Study findings will be presented to national and sub-national health officials to determine if and how group care will be integrated into policy and included as a strategy for service delivery. The study will contribute to the body of knowledge that will inform decision makers locally and globally on whether G-ANC is a feasible service delivery model that is more acceptable and effective than individual ANC.

### Ethical approval

Ethical approval was obtained from the Kenya Medical and Research Institute, Nairobi, Kenya; Nasarawa State MOH Research Ethics Committee, in Nigeria; and the Johns Hopkins Bloomberg School of Public Health Institutional Review Board, Baltimore, Maryland, United States of America (IRB000007078). The study is registered with the Pan African Clinical Trials Registry, PACTR201706002254227 ( www.pactr.org).

### Study status

**Figure 2.**
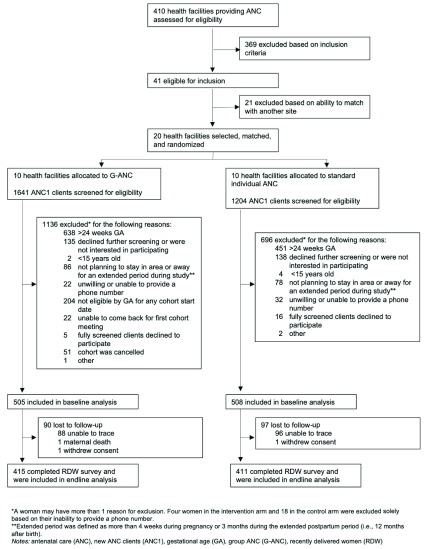
Enrollment of study participants in Kenya. CONSORT flowchart of the eligibility screening, consent, and enrolment of study participants in Kenya.

**Figure 3.**
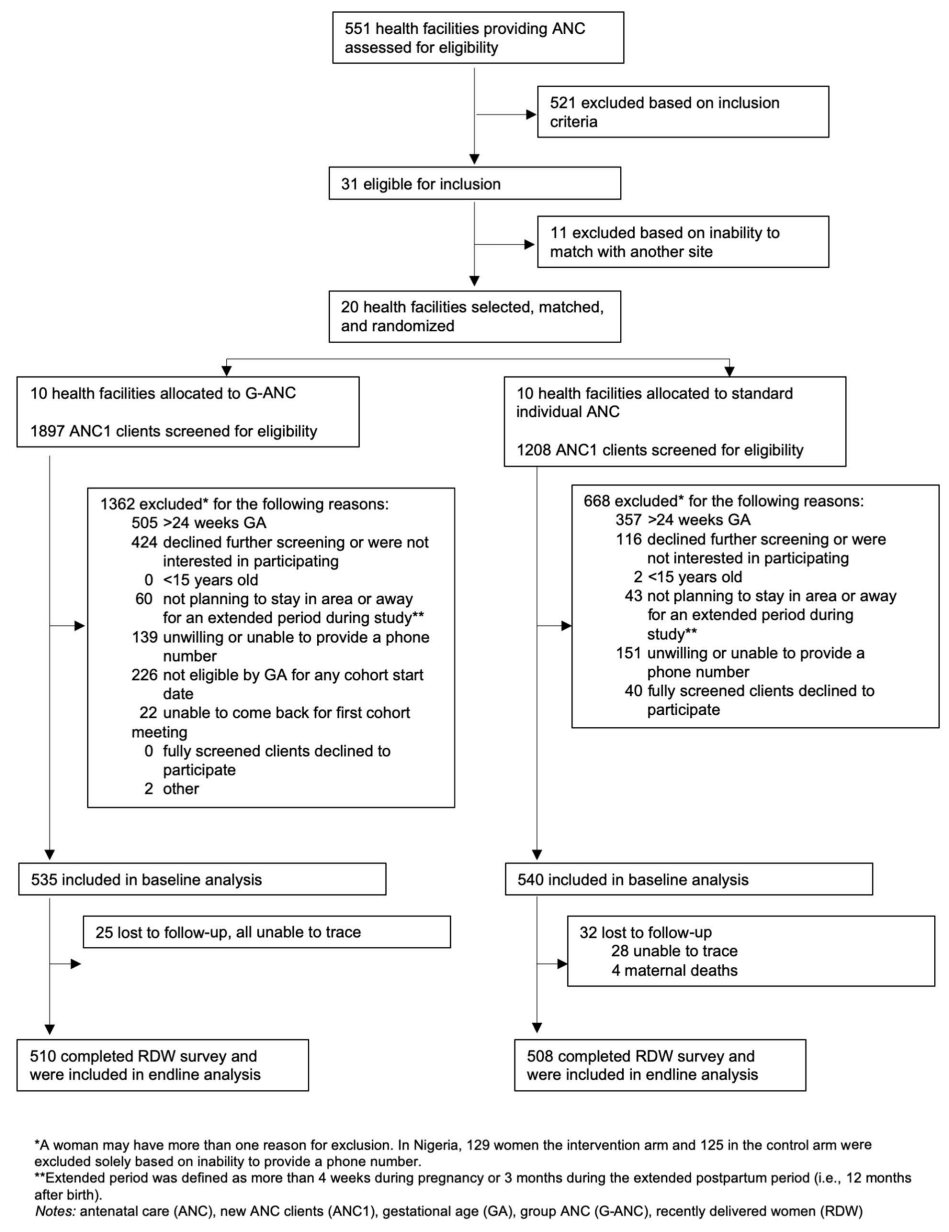
Enrollment of study participants in Nigeria. CONSORT flowchart of the eligibility screening, consent, and enrolment of study participants in Nigeria.

Enrollment of study participants and collection of baseline data began in October 2016, with the first group meetings occurring at the end of the month. Enrollment was completed in January 2017 in Nigeria and June 2017 in Kenya. Overall 1,075 and 1,013 pregnant women were enrolled in Nigeria and Kenya, respectively. Figure 2 and Figure 3 show the enrollment cascade for the two countries.

In Nigeria, G-ANC meetings for Phase 1 were completed in May 2017 with data collection for Phase 1 completed in September 2017. Phase 2 HMHB meetings were completed in May 2018, and data collection in July 2018.

In Kenya, Phase 1 G-ANC meetings were completed in October 2017 and data collection in January 2018. Phase 2 HMHB meetings and data collection will be completed in November 2018. Final study results will be available in February 2019.

## Discussion

The authors are not aware of any previous cRCTs on G-ANC or postpartum facility-based group care in Africa. The study will contribute to the body of evidence that will inform decision makers locally and globally on whether G-ANC is a feasible service delivery model that is more acceptable and effective than individual ANC. It will similarly inform stakeholders if continuing those cohorts is feasible and of sufficient value to implement. The findings of this study are expected to inform decision-making at different levels on whether to adopt the model as matter of policy, and how group care can be integrated into routine service delivery.

## Data availability

No data is associated with this article.

### Trial registration

The study was registered retrospectively on May 2, 2017, by the Pan African Clinical Trials Registry **PACTR201706002254227**

## Supplementary material

Supplementary File 1: Zip folder containing study manual, survey and consent forms.
