# Supplementary material for: Group versus individual antenatal and first year postpartum care: Study protocol for a multi-country cluster randomized controlled trial in Kenya and Nigeria
Source: Gates Open Res. 2019 Jul 4;2:56. Originally published 2018 Nov 5. [Version 2] doi: 10.12688/gatesopenres.12867.2 (PMC6350506; doi:10.12688/gatesopenres.12867.2)
Supplement: Supplementary file 1 [file gatesopenres-2-14160-s0000.tgz › 72242879-e244-48c8-a71c-b86050fb7704_Supplemental_files-_Study_manual,_Data_tools,_and_recruitment_,materials.zip!/Supplemental files- Study manual, Data tools, and recruitment ,materials/1. GANC Study manual_v2_Feb09, 2017.pdf]

GROUP ANC
STUDY MANUAL

Version 2

February 09, 2017

Table of Contents

Section I: Introduction **1**

Overview 1

Background and Description of Study 1

Purpose of manual 2

Inventory of study tools and documents 3

Data Collection Matrix 6

Contact Information for Study-Related Inquiries 7

Expectations for Research Assistants 9

Section II: Standard operating procedures (SOPs) for research assistants **15**

SOP 01 REDCap Mobile data collection 17

SOP 02:REDCap Data submission 23

SOP 03-05: First encounter with study participants 25

SOP 03:Screening and Recruitment 26

SOP 04: Managing Cohort Assignments 29

SOP 05: Consenting Pregnant Women 31

SOP 06:Enrolling Service Providers 34

SOP 07: Administering surveys 35

SOP 08: Contacting study participants for follow up 37

SOP 09: Determining Baseline Facility Based Birth Rate 38

SOP 10: RDW follow up, survey and ANC Data Extraction 39

Section III: Standard operating procedures (SOPs) for project officers and other study staff **42**

SOP 11: REDCap Access and Privileges 43

SOP 12: Data Management 44

SOP 13: Data Monitoring and Quality Assurance, data verification and submission by POs 47

SOP 14: Facility audits prior to enrollment (all sites) and commencement of group meetings

(intervention sites) 55

SOPs 15-16: Site visits and supportive supervision 56

SOP 15: Providing supportive supervision to Control sites 57

SOP 16: Providing supportive supervision to Intervention sites 59

Section IV: Standard operating procedures (SOPs) for group ANC facilitators **63**

SOP 17: Conducting Group ANC 64

Section 1: Introduction

Overview

- Background and description of study
- Purpose of study manual
- Inventory of study tools and documents
- Data collection matrix

Background and Description of Study

Introduction

In Kenya and Nigeria many women do not complete the recommended number of antenatal care (ANC) visits, and subsequent utilization of health care services such as facility based delivery, postnatal care, and family planning services remain below targets. Within ANC, the quality of care varies, resulting in women not receiving all the necessary evidence-based interventions or sufficient counseling and education. In developed countries, studies of group ANC have demonstrated increased attendance, knowledge, and patient satisfaction, as well as improved health practices and outcomes including reduced preterm birth and increased use of post partum family planning. Based on these findings, there is growing interest in exploring how group based care may influence health literacy and use of services in low-resource settings.

Jhpiego will conduct a study to evaluate the effectiveness, acceptability and feasibility of Group ANC as compared to traditional ANC. Group visits will then continue into the first year after birth. In group care women of the same gestational age come for clinical visits at the same time and receive care together, in a group. It includes 1) self-assessment 2) private consultation with a provider 3) time for group learning and socializing.

Study results will inform whether group care is a viable strategy to improve the quality and acceptability of ANC and increase retention in care and the use of key services through pregnancy, childbirth, and the postnatal period.

Study Design

This is a two-phase randomized control study. Phase I: ANC, covers enrollment through delivery (5 group visits). Phase II: Healthy Moms Healthy Babies, covers 3-12 months postpartum (4 group visits). There will be 10 intervention sites and 10 control sites in each country that have been matched on various criteria and then randomized. In the intervention sites, a group care model will be led by pairs of providers and community health extension workers, trained as facilitators who will lead a series of structured meetings that integrate the usual health assessments with facilitated discussion providing education and peer support for a group of 8-15 pregnant women of approximately the same gestational age. A total of 9meetings will be conducted: five during

pregnancy and four in the year after birth. The total study sample size in Kenya will be 1,026 women and 1076 in Nigeria. There will be a total of four-five cohorts per facility.

Project Location

In Kenya: Kisumu county (8 facilities), Machakos county (6 facilities), and Tharaka Nithi county (6 facilities).

In Nigeria: Nasawara State (20 facilities)

Project Duration

November 2015–November 2018 (3 years).

Outcomes of interest

Do women who receive ANC through a group ANC model, compared to women who receive traditional individual ANC, have higher rates of birth with a skilled birth attendant (SBA)?

Do women who participate in group care through 1 year after birth, compared to women who receive traditional care in the year following birth, have higher rates of family planning use at one year following birth?

Additional secondary outcomes will assess:

- Health literacy
- Satisfaction of both women and providers
- Service utilization (ANC, PNC, FP)
- Self-efficacy as related to health behaviors.

Purpose of manual

The purpose of this manual is to avail a collection of Standard Operating Procedures (SOPs) for implementing the group-ANC study. It should be used by all study staff and those involved with the study to ensure consistency in protocol implementation and data collection across participants and in all study sites.

In preparing the manual, the Investigators have taken into consideration the terms of award with respect to required reporting as well as Institutional Review Board (IRB) approvals.

The manual details the study's conduct and operations. It is a guideline that describes the study's organization; data collection methods - recruitment, screening, enrollment, follow-up and quality control procedures. It facilitates adherence to study procedures.

The manual will be updated throughout the study as needed to reflect any changes in the protocol or study procedures.

Inventory of study tools and documents

IRB approved tools:

01 Eligibility Screening form o To be used with every new ANC client during the enrolment period o Screens for eligibility and collects basic demographic data

02 Study script o Used to introduce study to potential study participants

03 Consent of pregnant women o Consent form used with pregnant women who agree to participate in study

04 Cohort log o Record of study participants assigned to each cohort, used by RA and health care providers

05 Consent health care worker o Consent form used with CHEW s and ANC providers in intervention sites

06 Baseline survey – pregnant women o Administered immediately after consent obtained

06a facility based delivery survey – women who began ANC prior to the start of the study

o Administered via phone to ANC I clients recorded in ANC register up to 6 months prior to the start of the study who were <24 weeks GA at the time of ANC I visit

07 Baseline survey – trained group ANC facilitators at intervention sites o Administered immediately after consent obtained

08 Phase I survey – recently delivered women o Administered in person 3-6 weeks after delivery for both control and intervention

09 Phase I survey – health care workers

o Administered to each group ANC facilitator (intervention sites only) after all cohorts at facility have finished the last group ANC meeting

10 Phase I FGD guide – pregnant women o Guide to use in conducting focus groups with subset of study participants from intervention sites

11 Phase I FGD guide – health care workers o Guide to use in conducting focus groups with subset of group ANC facilitators at the end of Phase I

12 Phase II survey – women 12 months postpartum o Administered to all study participants at 12 months postpartum

13 Phase II survey – health care workers o Administered to each Phase II group facilitator (intervention sites only) after all cohorts at facility have finished the last Healthy Moms Healthy Babies group meeting

14 Phase II FGD guide – pregnant women o Guide to use in conducting focus groups with subset of study participants from intervention sites after they have completed all meetings

15 Phase II FGD guide – health care workers o Guide to use in conducting focus groups with subset of Phase II group facilitators at the end of Phase II

16 a. Phase I Group ANC register – intervention sites only o Study specific register to be used during private consultations during group meetings. One register per group cohort

a. Kenya ANC data extraction – all sites, from normal ANC register and/or mother child booklet
b. Nigeria ANC data extraction – all sites, from normal ANC register and/or patient record

17 Phase II Group register – intervention sites only o Study specific register to be used during private consultations during group meetings. One register per group cohort

Additional documentation (Checklists, logs, reports, diary entries, etc):

101 Facility baseline assessment prior to randomization

102 Facility audit prior to commencement of study enrolment

103 Facility audit prior to commencement of group ANC meetings

104 Facilitator quality assurance tools (for use by facilitators)

105 Baseline facility delivery rate log

106 PO Site visit and RA Supervision log

107 Enrolment log: control sites

108 Enrolment log: intervention sites

109 PO weekly report: to enter in REDCap

110 Site visit and supportive supervision checklist: intervention site

111 Site visit and supportive supervision checklist: control site

112 PO group meeting attendance log

113 Fidelity checklist

114 PO observation of facilitators

115 PO debrief with facilitators

116 RA ANC follow up diary: intervention 117 RA ANC follow up diary: control site

Data Collection Matrix

Both quantitative and qualitative data collection approaches will be used in this study. Quantitative data includes surveys of pregnant women and ANC providers at three points in time as well as data extraction from group ANC registers and standard ANC registers. Qualitative data will be collected primarily through focus group discussions. The table below shows a summary of the data collection schedules for the various study participants.

		INTERVENTION FACILITIES		CONTROL FACIILITIES
**PHASE**	TOOL AND DATA COLLECTION METHOD	Health care worker at Intervention Sites	Clients participating in Group Care	Clients in traditional individual care
Screening clients	01 Screening form Verification of inclusion criteria and basic demographic data		X	X
Phase 1	06, 07 Baseline questionnaire, n=all who are enrolled	X	X	X
Phase 1	10, 11 Focus groups at completion of GANC meetings, n=subset, to saturation	X	X	
Phase 1	08, 09 Questionnaire after completion of ANC, n=all	X (after last cohort finishes ANC)	X (3-6 weeks after delivery)	X (3-6 weeks after delivery)
Phase 2	14, 15 Focus groups at completion of group care during year following birth n=subset, to saturation	X	X	
Phase 2	12, 13 Questionnaire at completion of 1st year postpartum	X (after last cohort finishes)	X (after 12 month group meeting)	X (at 12 months PP)
Phases 1&2	16 a-c Review facility records (group registers and normal registers) and MCH booklet		X	X

Phases 1&2	Model fidelity checklist assessing implementation of group process	Completed during meetings	N/A

Contact Information for Study-Related Inquiries

In Kenya, questions regarding the study protocol or this manual should be directed to the G-ANC Project Manager, George Avosa, email:george.avosa@jhpiego.org.

Adverse event reports should be sent to the in-country PI, Antony Gichangi, email: anthony.gichangi@jhpiego.org.

In Nigeria, questions regarding the study protocol or this manual should be directed to the G-ANC Project Manager, Jaiyeola Oyetunji, email: Jaiyeola.Oyetunji@jhpiego.org

Adverse event reports should be sent to the in-country PI, Emmanuel Ugwa, email: Emmanuel.Ugwa@jhpiego.org.

**Jhpiego, Baltimore**
Address:<br>1615 Thames Street<br>Baltimore, MD USA 21231
Lindsay Grenier, Project director and co-investigator<br>(lindsay.grenier@jhpiego.org)

Jhpiego, Kenya

Address:

14 Riverside, Off Riverside drive,

Arlington Block, 1st floor. PO

BOX 66119 – 00800, Nairobi

Kenya.

Anthony Gichangi, Site PI	George Avosa, Project Manager
Anthony gichangi, Site PI	George Avosa, Project Manager
(Anthony.gichangi@jhpiego.org)	(george.avosa@jhpiego.org)

Jhpiego, Nigeria

Address: Plot 971 Reuben Okoya
Crescent, off Okonjo-Iweala
Way, Wuye, Abuja.

Emmanuel Ugwa **,** Site PI	Jaiyeola Oyetunji, Project Manager
(emmanuel.ugwa@jhpiego.org)	(jaiyeola.oyetunji@jhpiego.org)

Expectations for Research Assistants

The Research Assistant will work closely with Monitoring Evaluation and Research team and Project officer in each country to ensure that the Study results inform whether group care is a viable strategy to improve the quality and acceptability of ANC and increase retention in care through pregnancy, childbirth, and the postnatal period.

Successful data collection is an art and should not be treated as a mechanical process. Each interaction with a study participant is an opportunity to collect very valuable data that will be used to answer the research questions; it should be kept pleasant and conducted in professional manner.

The main role of a research assistant is to collect AND document accurately all the necessary data as described in this manual to the best of his/her ability. The study procedures and instructions in the study tools / documents should be adhered to at all times.

Scope of work for RAs

- Be present in assigned heath facility during **all hours antenatal care is provided** for four months to screen, recruit and obtain consent from ANC mothers on whom the research is conducted
- Transfer initial screening information from providers to REDCap (a mature, secure web application for building and managing online surveys and databases) via project provided tablet
- Create group cohort logs; share group cohort logs with providers; update project officers weekly with enrollment numbers
- Administer baseline survey questions to all consented study participants at the time of consent via REDcap
- Using facility records, conduct phone based facility birth rate baseline assessment
- Track study participants in order to administer surveys at appropriate times; record if subjects move out of study area and/or are lost to follow up
- Arrange meeting time and place and administer survey questions to recently delivered study participants within 6 weeks of delivery via REDCap
- Support Focused group discussion in their respective areas as requested by the research team
- Review collected data and make corrections before submission via redcap
- Review and bring into force varied research quality and control procedures.
- Ensure safe custody of equipment supplied for the study
- Keep accurate records of consent forms and other study related documents.

Handling the tablets

Each research assistant will be provided an electronic tablet for study use only. The tablets include a program called REDCap which includes all study tools. The program will prompt you through administration of each study tool, and all data will be recorded directly in the tablets. Data collected should be uploaded to the server on a regular basis, via internet or sim card.

Research assistants are responsible at all times for their tablet, which should remain surpervised and/or locked at all times.

The electronic tablets are for research assistant to use strictly for the purposes of this study and not to be used for personal purposes, which include games, social networking, downloading music or photos, or internet browsing.

The electronic tablets are the property of Jhpiego and not the property of the person/persons using them. They must be returned to Jhpiego after data collection is complete. In the event that a tablet is damaged or stolen, immediately contact the Project Officer. Non-adherence to these guidelines may result in disciplinary action and where necessary termination of the contract.

- *Ensure safe custody of tablets supplied for the study*
- *Do not use tablet for personal use*

Enrollment of study participants

The research assistant must be present in the assigned health facility during all hours antenatal care is provided during the entire enrollment period to screen, recruit, and consent study participants.

Pre-screening – 01 screening form, Part 1

ANC service providers will use Part I of tool 01 to screen all pregnant women presenting for their first ANC visit to determine their eligibility based on gestational age (<24 weeks). The service provider will provide the RA with all screening forms, regardless of eligibility. Women who meet the gestational age criteria will be referred by the provider to the RA on-site for additional study screening immediately after their ANC visit.

- *Enter data from Part I of the screening form (01) for every ANC I client (even those who are ineligible) into REDCap by the end of each day.*

Screening – 01, Parts II-IV

When a client is referred to the RA, he/she will first introduce themselves and then read the study information sheet to the potential study participant. Group ANC will be described ONLY to women in the intervention sites. They will then administer the remainder of the screening tool using the study tablet.

For each client referred to the RA, the RA will immediately:

- *Begin new record in REDCap*
- *Enter pre-enrollment data*
- *Read study information sheet*

- *Administer survey questions to determine inclusion criteria and collect basic demographic data* ***If intervention site:***
- *RA will check with group ANC facilitators at the start of every day to receive updates on Group ANC meeting scheduling. RA will confirm the date and time of the next G-ANC meeting 1*
- *Identify eligible group cohort*

Informed Consent and placement in group cohort

A research assistant will obtain written consent from all eligible participants who are willing to join the study, via signature or thumbprint (for the illiterate), after explaining the content in the appropriate language which the participant understands. Update project officers on a weekly basis regarding enrollment numbers. Retain all signed consents and give to the study project officer on a monthly basis.

For intervention sites, once consent is obtained the participant must be assigned to a specific group cohort. Add the participant to the paper cohort log. This log will be used to track all the participants enrolled in that cohort, and later, to contact women for subsequent surveys. Notify PO immediately once 13 women have been enrolled in a cohort (See SOP 04 for more details on next steps).One week prior to the first meeting of a new cohort, confirm AND inform the PO the total number women enrolled (Refer to SoP 04 for more details, especially on next steps if <8 women are enrolled by then). A single log will be completed for each cohort.

- *Obtain consent, retain records and give to project officer on monthly basis*

- *Update POs regarding enrollment numbers weekly (in intervention sites, alert when cohort reaches 13)*

In intervention sites:

- *Add subject to hand written cohort log, alert PO when reach 13 subjects in cohort*
- *When cohort is closed to new additions, photocopy cohort log and share with group ANC providers*

Facility based delivery baseline survey

The purpose of this survey is to determine the percentage of women receiving ANC care at the study sites who went on to deliver at a facility prior to the start of the study. This is so we can compare it with the number of women participating in the study who go on to deliver at a facility. You will use the facility ANC register to ID and extract phone numbers of all women who had

their first ANC visit at <= 24 weeks gestational age in the 6 months preceding the study. This information will be logged in form 105, Baseline facility delivery rate log. You will then call those women after they are likely to have delivered based on their EDD (estimated date of delivery). After obtaining consent via phone, record the data collected in the SBA tool, 006a (consent script is included in the tool). This should be done while at the facility, sometime when RA is not screening and consenting study participants during the enrolment period. See SOP 09 for detailed instructions.

- *Review ANC register and ID clients eligible for facility based delivery baseline survey*
- *Create a record in REDCap for every identified eligible client, note if contact information available*
- *Obtain verbal consent via phone*
- *Administer survey via phone*

Participant Surveys

There are 3 surveys for enrolled pregnant women (from both control and intervention sites) and 3 surveys for health care workers (group facilitators at the intervention sites only):

Baseline: administered at the time of consent (enrolled pregnant women and health care workers)

Phase I: administered at 3-6 weeks postpartum for recently delivered women and at the end of Phase I for group facilitators

Phase II: administered 12 months postpartum for women and at the end of Phase II for group facilitators

The research assistant will be responsible for tracking each cohort and knowing when each survey should be administered. For phase I & II surveys the RA will contact women to set up times and locations to meet for survey administration and will carefully track which women need continued follow up.

First choose which of the available languages will be easiest for the subject to understand and respond in (English, Kiswahili, Hausa). The items on the surveys must be read as written to avoid introducing bias. Do not try to re-word to make them easier to understand.

Listen attentively in order to record the truest and closest answer to the options listed in the questionnaire. If the answer is different from what is listed, manually type it into the 'other' field. This involves asking questions, listening, observing and recording the answer accurately. This does not involve teaching, critiquing or telling the answer.

- *Track study participants in order to administer surveys at appropriate times; record if subjects move out of study area and/or are lost to follow up*
- *Administer baseline survey via REDCap at the time of consent for all study participants*
- *Schedule the meeting time and place and administer phase I survey questions to recently delivered study participants within 6 weeks of delivery via REDCap*
- *Schedule meeting time and place and administer phase I survey questions to group facilitators after they have completed their final ANC meeting*
- *Review collected data and make corrections before submission via redcap*
- *Meet with Project officer on a regular basis*

Administration duties

- Be at the assigned facility during all ANC clinic hours, 8:00 am to 5:00 pm every weekday (Monday-Friday) and if required, on weekends/public holidays – in consultation with the PO.
  NOTE: *RA to notify PO in advance if s/he will not be able to report to work due to an illness or other emergency.*
- Take care study of documents in their possession during the day and keep them in a locked cabinet at the end of each work day.
- Ensure they have enough supplies to last three days and alert project officer when running low on copies of study related docs, at least a week before they run out.

Section II: Standard operating procedures (SOPs) for research assistants

SOP 01: REDCap Mobile data collection

Version	Effective Date	Supersedes	Approved by	Date
1.0		None: New		

Purpose

This SOP describes the basic steps the Research assistant needs to follow while collecting data using the REDCap application.

Scope

This SOP applies to all study staff and research assistants who have direct involvement or oversight in the Group ANC study on how to collect data using the REDCap mobile application.

Overview

Project Administrator will enable the mobile data collection option to be used by Research Assistants in collecting data. The PA will make sure each RA has a user account in REDCap with a defined role for data collection and submission.

Assigning Participant ID

Participant ID will be pre-assigned; the pre-screening forms to be completed by service providers will have pre-printed Participant ID comprising of the facility MFL code and a three-digit No. (e.g. 12004/001).

Procedure

This procedure must be carried out in full and in a standardized manner to ensure the data is collected in a consistent manner. The procedure is applicable to users who have access to the REDCap system.

1. Switch the power button and check the battery status (battery should be charged the previous night till its full the previous night)

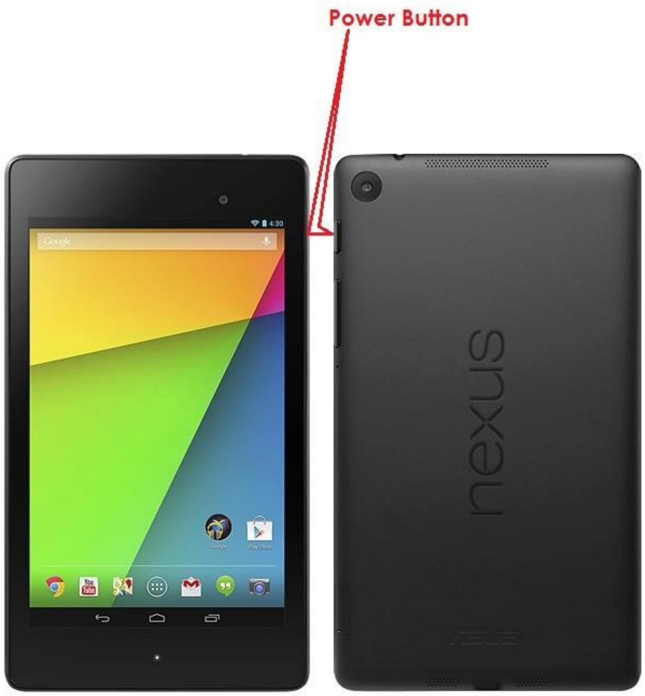

2. Once the power button is on, tap on the REDCap application to load/ open it

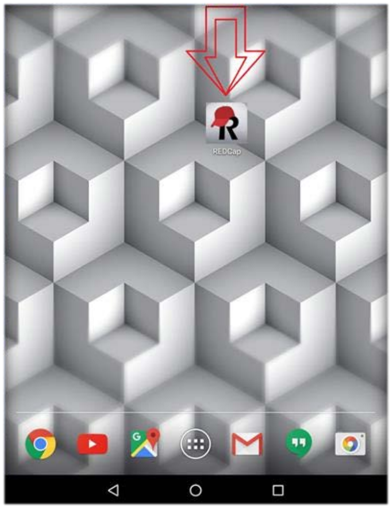

3. Enter the pin and click on login to open the ***offline data collection*** page

4. In case you have forgotten the Login Pin, dial phone number **0725276189 / 0715555875**

5. Once on the login page, click on ***My Projects***, then ***Group ANC: Women Survey, Collect data, Create New Record***

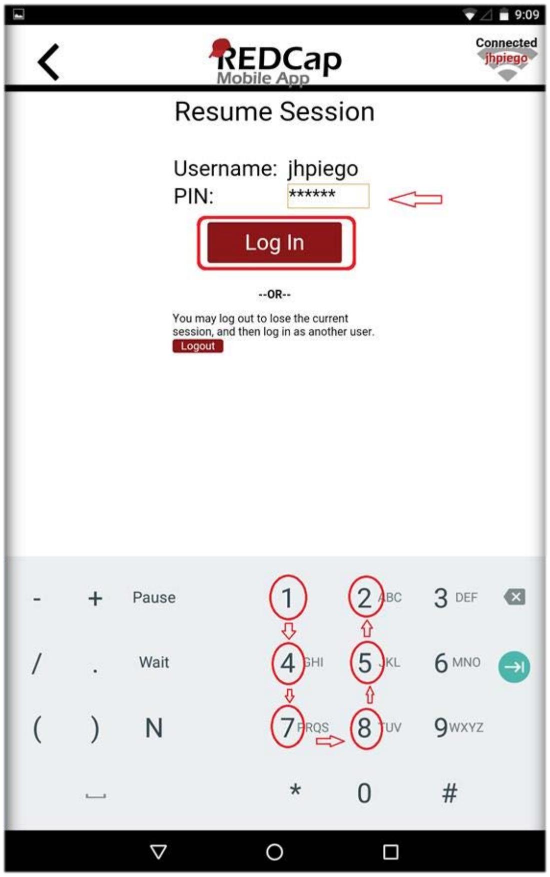

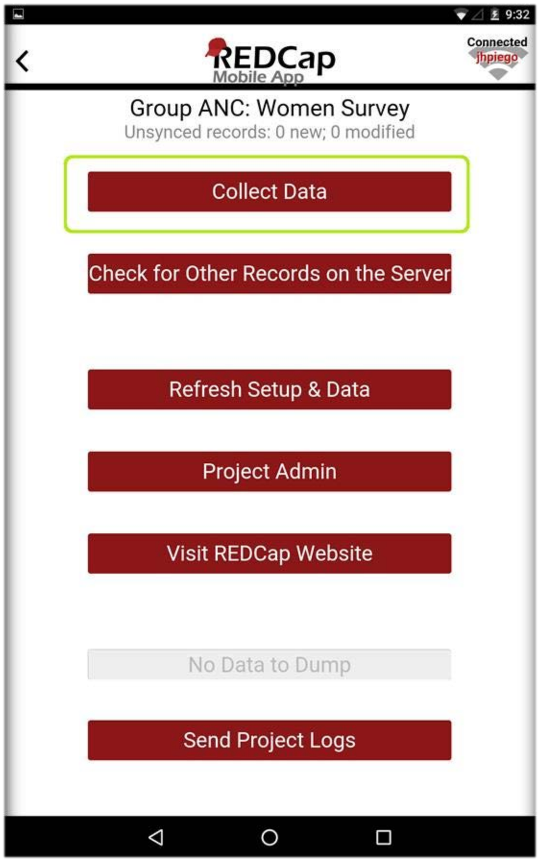

6. Enter the new record name (In the format stipulated here(MFL Code / 3-digit code). This is the Participant ID printed on the prescreening form.

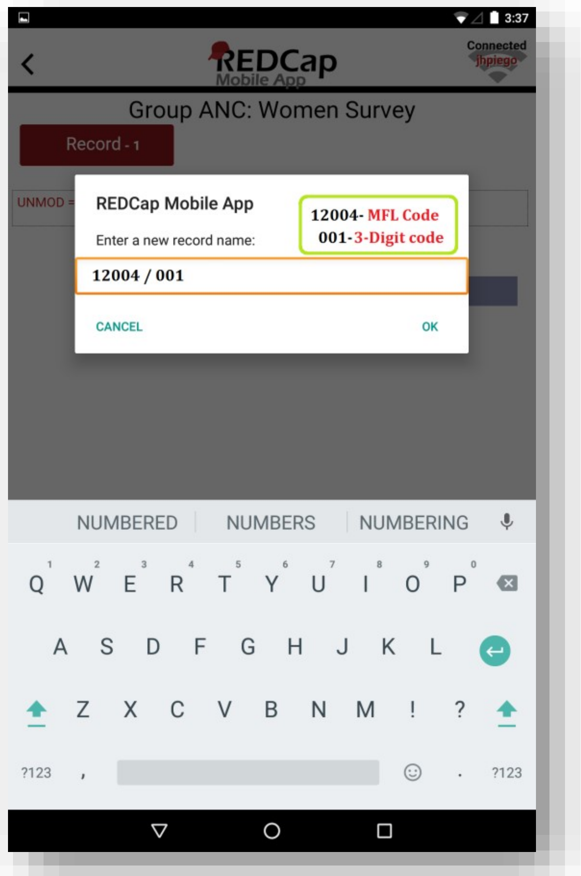

7. Click on the Screening and Consent form (See SOP 3-5 for details in administering this tool)

8. Once the screening and consenting is completed, check for completeness, select "unverified" as the form status, save and continue. Immediately return to the **Baseline Survey: Pregnant Women**

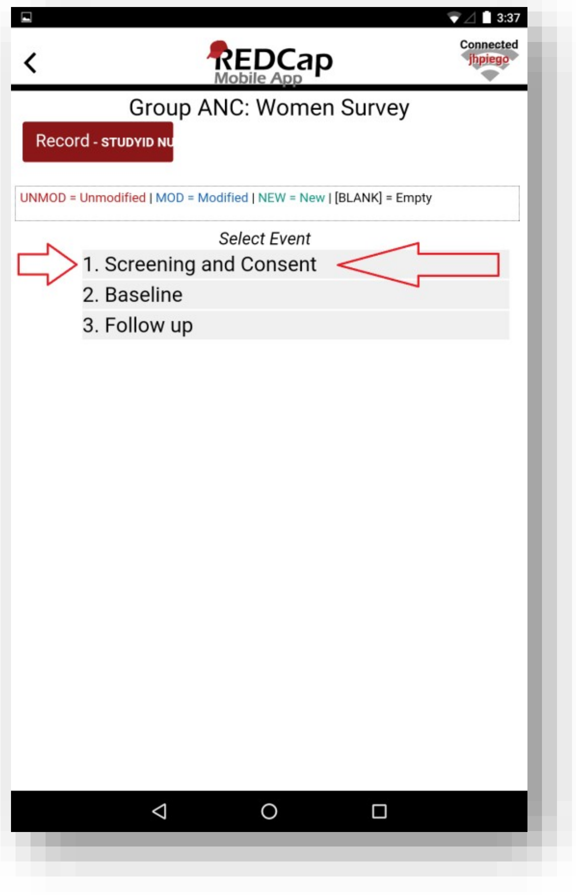

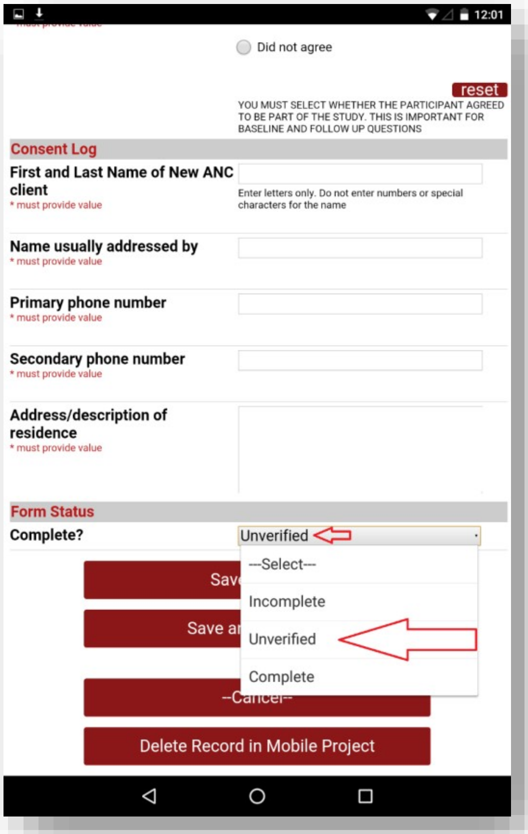

Data quality check and saving records

For each interview form, once complete review the form in the presence of the client to ensure all the information has been recorded. For any field where data is not recorded, the research assistant will note separately, in the comment section at the end of the form, the item number of the missing field and the reason for omission (e.g. client refusal; client unable to understand etc).

After reviewing the form for any missing data, mark the form as unverified and save and continue to return to the home page. All data will be verified and noted as incomplete or complete by project officers.

To enter another record, click on the return arrow in the upper left hand corner and select *'Create New Record'*.

Retrieving Record ID during follow up data collection

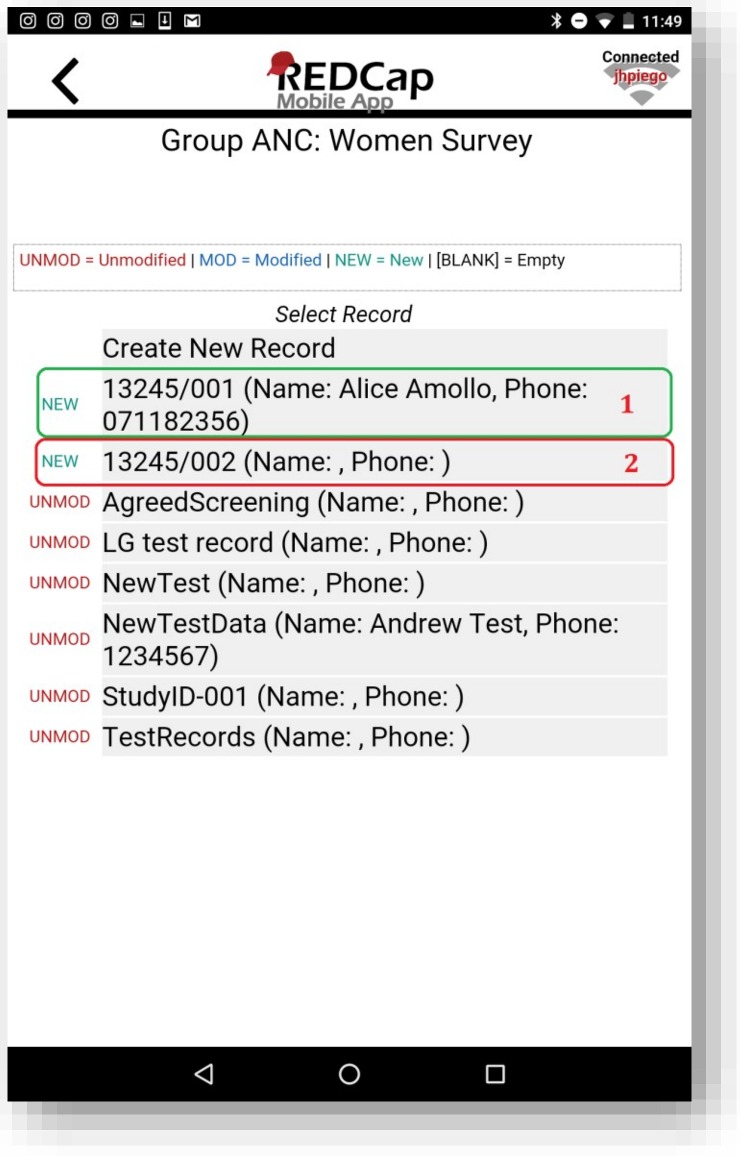

When you log on to REDCap mobile app, go to ***my projects>> G-ANC>> collect data.***

All record IDs in the tablet will be displayed as shown here. For follow up data collection, match the record ID on the tablet with the one on the diary. Click on it to open the follow up questionnaire.

SOP 02: REDCap Data submission

Version	Effective Date	Supersedes	Approved by	Date
I.0		None: New		

Purpose

To define data submission responsibilities and procedures by RAs and POs

Scope

This SOP applies to study staff and administrators who have direct involvement or oversight in the Group ANC study on how to submit data using the REDCap mobile application.

Overview:

Project Administrator will enable the mobile data submission option to be used by Research Team in submitting data. The Administrator will make sure each user has an account in REDCap with a defined role for viewing data and its submission. The Research assistant shall submit data after which the Supervisors will verify the data for completeness.

Procedure

Data should be uploaded via the internet daily.

This procedure must be carried out in full and in a standardized manner to ensure the data is submitted in a consistent manner. The procedure is applicable to users who have access to the REDCap system.

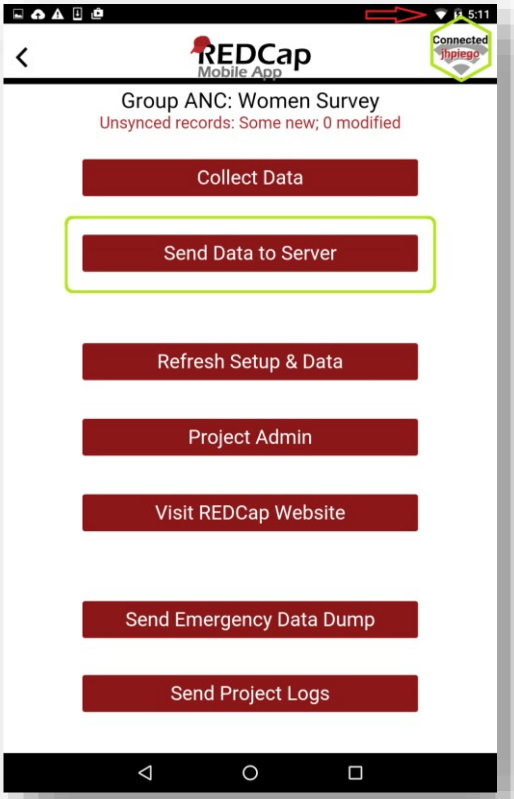

1. After connecting your tablet to the internet through a secure Wi-Fi network or tethering your smartphone to act as a Wi-Fi Hotspot, Go to the Redcap mobile App in your tablet, log in and click on **send data to server**

2. It will prompt you, click on **Begin send of all data.**
3. It will indicate on the **progress bar** whether the data is being submitted or

not. Please be patient as this may take a few minutes.

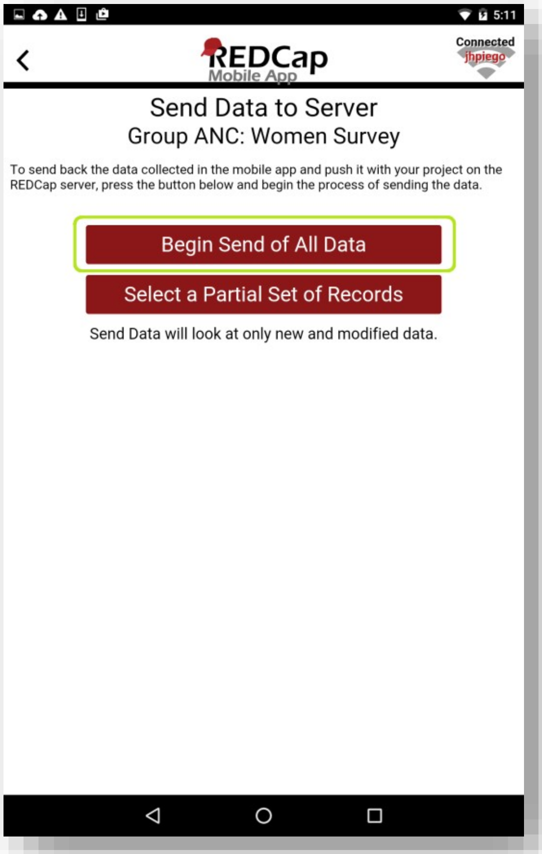

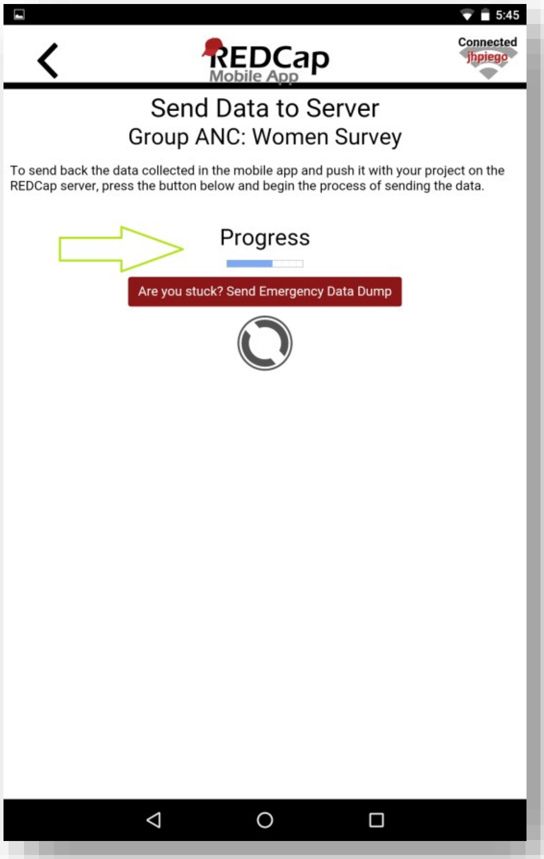

4. When done, click on the Back to Project button and **do not** click on refresh setup and data button as recommended by the system

5. **NOTE:** If the RA notices they made an error after they have submitted the data and would like to make changes, they should first notify the PO. The RA should not at any time overwrite any existing data without the permission of the PO. The PO will confirm whether the changes need to be made and submit the data ***after*** reviewing both the existing record and the new record.

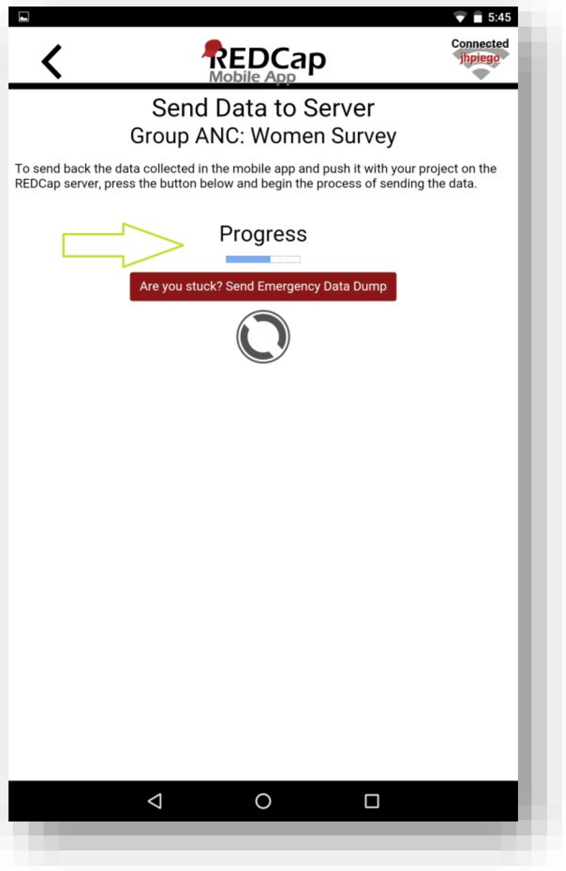

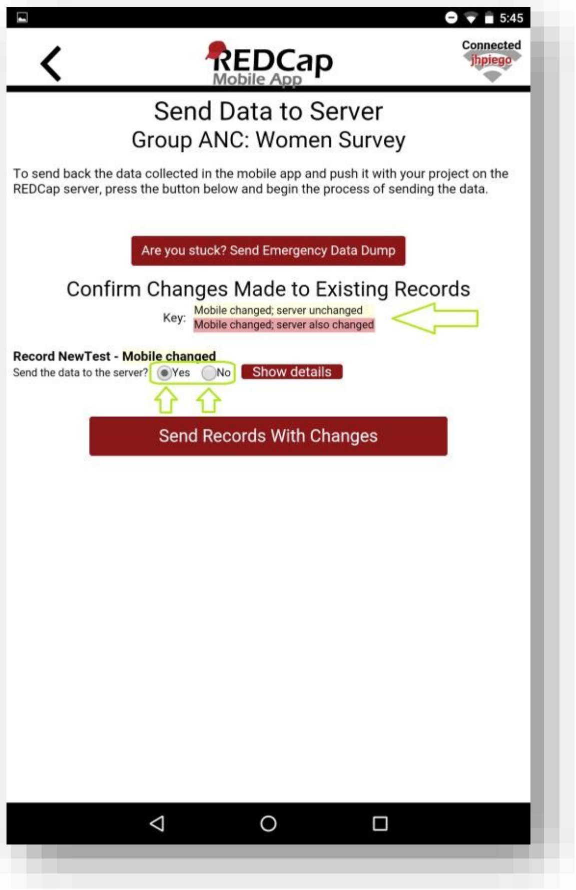

SOP 03–05: First encounter with study participants

<u>Purpose</u>: These SOPs (03–05) describe procedures for all activities which occur during the initial interaction with a study participant: screening and recruitment; Consent; and administration of baseline questionnaire.

<u>Scope</u>: These SOP's apply to all study and facility staff who have direct involvement or oversight in screening and/or consenting participants into the G-ANC sessions. SOP 03 and 05 apply to both intervention and comparison study sties. SOP 4 applies only to intervention sites.

<u>Overview</u>: Service providers will be responsible for pre-screening all first time ANC clients based on gestational age and referring them to Research Assistants (RA) who will then read the study script, and complete the eligibility screening. Those found to be eligible and who agree to participate will be consented. In intervention sites, they will be assigned to group ANC cohorts. The baseline survey will then be administered to those who consent. Health facility staff chosen as group facilitators are also study participants and will be consented prior to having the baseline survey administered to them but are covered under SOP 06: Enrolling Health care providers

Ensure following materials are in hand:

- ☐ A charged tablet and battery backup
- ☐ Pens
- ☐ Ink pad
- ☐ Clipboard
- ☐ Copies of tools
    - o 01 Eligibility screening form, part A, with prefilled sequential #s (45 at start of study)
    - o 02 Study script In English/Kiswahili and/or Hausa (45 at start of study in native language+ 10 in English)
    - o 03 Consent of pregnant women (45 at start of study in native language + 10 in English) o 04 Cohort log (1 copy)
    - o 06 baseline survey pregnant women (2 back up copies in each language in case tablet fails)

SOP 03: Screening and Recruitment

Version	Effective Date	Supersedes	Approved by	Date
1.0		None: New		

Procedures

001 Part I: Gestational Age-Screening by Service Providers

The service providers will fill out Part 1 of the– Eligibility Screening Form (Tool 001) for every single ANC 1 client to filter and refer clients to Research Assistants (RAs) stationed at the health facility.

The service providers will:

1. Document the gestational age on the paper-based screening form for every new ANC client whose GA is 20 – 24 weeks. The screening form will have a pre-assigned participant ID comprising of the facility MFL code and a three-digit no (e.g. 12004/001)
    a. If women do not meet gestational age criteria: continue with routine ANC care and give the screening form to the RA before the end of the day.
    b. If women do meet gestational age criteria: introduce the study and ask if the client can be referred to the research assistant (RA) on-site for additional screening

001 Part II-IV: Eligibility screening, collection of demographic data, placement in cohort - Research Assistant

The RA must adhere to the following steps to ensure only eligible participants are enrolled into the study:

1. Prior to meeting the potential client, ensure the tablet is charged and functional. Have printed copies of the study script and consent forms. Have printed copies of screening form and baseline assessment in case of tablet problems.
2. RA introduce him/herself to the potential participant and review the screening sheet completed by the service provider. Ensure client is eligible by gestational age (GA) prior to proceeding.
3. Enter data completed by the clinician (part I) into tablet and assign client a study PID as per SOP 01: *REDCap Data Collection SOP.*
4. Enter date of the first group meeting for the next cohort in tablet to confirm eligibility by GA
    a. If the woman is not eligible, terminate, thank her for her time and direct her to proceed with routine ANC care. Tell her to check with a provider if she is not sure when to return. To close survey in REDCap, follow the steps in SOP 01.
5. Assess if client is comfortable speaking in English, Kiswahili or Hausa.
    a. If the client is not able to communicate easily in any of these language, thank her, terminate the screening and refer her back to routine ANC care.
6. Read study script and obtain oral consent from her to ask her the screening questions using the following script:
   My name is __________________ (Name of RA). I am part of a research study supported by Jhpiego and the Ministry of Health to compare group antenatal care with usual care of one patient at a time. Let me first tell you about the study. [RA reads the study information sheet to client, only describe group ANC to women in sites]
   "I would like to ask you a few questions to see if you are eligible to participate in the project. We will collect your name and phone number for the purpose of reaching you. Your answers will only be seen by study staff. This will take less than 5 minutes. May I ask you a few questions?"
7. If she agrees, ask the screening questions, one at a time and determine if the woman is eligible for the study (see eligibility requirements below).

a. If the woman is not eligible, terminate, thank her for her time and direct her to proceed with routine ANC care. Tell her to check with a provider if she is not sure when to return. To close survey in REDCap, REDCap, follow the steps in SOP 01

b. If the woman is eligible, proceed to the next step of collecting basic demographic information from her (collected from all clients who meet eligibility criteria prior to consent)

8. Collect demographic information

Eligibility requirements:

- *Gestational age at time of first Group ANC meeting <=24 weeks GA*
- *Age ≥ 15 years*
- *Planning to stay in the area for the next 1 ½ years and continue her antenatal care at the facility*
- *Not planning to be away from home (near the facility) for more than 4 weeks in a row at any time during her pregnancy or more than 3 months in a row in the year after delivery.*
- *Able and willing to provide phone number where we she can be reached and reminded of upcoming ANC visits and/or to set up appointments for data collection.*

Note:If the response to any of the above is NO, client is not eligible to join the study.

The next steps will depend on whether the facility is an intervention or control site.

Intervention Facilities

1. Verify what cohort the client should be placed in based on her gestational age (GA)– she should be 20-24 weeks at the time of the first group visit.

    a. Keep in mind, if the next cohort to start is #2 (for example), and she will not qualify because she won't be ≥20 weeks GA on that start date, her eligibility should be assessed for cohort #3

Note: The G-ANC date will have been discussed and agreed on between study staff (PO) and the facility in charge.

2. Share the date and time with the subject and ask her if she is interested and will be able to attend at that time.

    a. If she responds yes/probably or unsure, assign to cohort (SOP 4) and proceed with consent (SOP 05).

a. If she responds No/probably not, terminate, thank her for her time and direct her to proceed with routine ANC care. She will not be included in any further study related activities. Tell her to check with a provider if she is not sure when to return. To close survey in REDCap, follow the steps in SOP 01

Control Facilities

1. ALL eligible clients who are willing to participate will be consented after screening and collection of demographic information.
2. Proceed to consent participant as per SOP 05.

SOP 04: Managing Cohort Assignments

Version	Effective Date	Supersedes	Approved by	Date
1.0		None: New		

Purpose

To outline practices surrounding management of group ANC cohorts to ensure final cohort sizes are 8-15 women, all of whom are between 20-24 weeks GA at the time of their first meeting. This includes description of regular communication which must happen between various parties involved in the study

Scope

This SOP applies only to study staff and Group ANC facilitators working in intervention sites

Procedures

1. Group ANC facilitators, in conjunction with other facility staff as needed, and the relevant project officer, will schedule group meetings for the 1st cohort prior to the study initiation (screening of women).
    a. Five meetings should be scheduled 4 weeks apart for each cohort
    b. If possible, hold meetings for each cohort on the same day and time (e.g. cohort I always meets Tuesdays 10:00-12:00; cohort II always meets Thursdays 12:00-2:00 etc)
    c. For most facilities, plan to start a new cohort every 4 weeks.

i. In a very busy facility, it may be decided to start new cohorts (hold group ANC meeting 1) every 2-3 weeks rather than every 4 weeks. This decision should be based on caseload of ANC1 clients <24 weeks. As a rule of thumb, if > 30 are seen a month, plan to start a new cohort every 3 weeks. If >40 are seen a month, plan to start a new cohort every 2 weeks. If >60 are seen a month, plan to start a new cohort every week.

2. The RA will check with facility staff every morning to assure they have the most updated schedule.
3. REDCap is programmed to lead you through the process of assigning women to cohorts. Women should be 20-24 weeks GA at the time of the first group meeting for the cohort in which they are enrolled. For women who enter care very early (e.g. 15 weeks GA), they

   may not be ≥ 20 weeks by the next cohort start. If not they would, however, qualify for the following cohort.
4. Once women have consented, enter their details in the paper based cohort log
   a. Keep this log for your records, you will use it to contact women for subsequent surveys
   b. When enrollment for the cohort is closed, provide a copy to both the project officer and group ANC facilitators at the facility
5. Write down the date and time of the first group meeting for their cohort on their ANC card, mother child booklet and/or in their phone or anywhere else the mother feels would be a helpful reminder
6. When 13 women have been enrolled in a cohort
   a. The RA will alert the project officer and facility staff
   b. The facility staff and project officer will examine the start date for the next cohort (group meeting 1) and determine if adjustments should be made (i.e. move up start date).
7. When 15 women have been enrolled in a cohort
   a. The cohort should be considered full and enrollment should begin in the next cohort (this means screening should consider eligibility based on the next cohort's start date)
8. One week before every cohort start date the RA will
   a. Give a copy of the cohort log to the group ANC facility staff
   b. If enrollment is <8 women alert facility staff and project officer immediately

      i. The project officer will examine the GA data of women enrolled thus far and determine together with facility staff whether and when to reschedule cohort start date(s). Each cohort must have a minimum of 8 women enrolled to commence.

         1. Project officers should contact the in country PI for assistance ii.

      Once the cohort start date is re-scheduled

1. All women should be notified that original start date is cancelled
2. Women who will be >24 weeks at that time should be counseled to return to the clinic for routine individual ANC
3. Women who will be 20-24 weeks GA at that time should be told the new start date and time

The RA must alert facility staff and the project officer when:

- *Fewer than 8 women are enrolled in a cohort 7 days before it's start date*
- *Once 13 women are enrolled in a cohort*
- *Once a cohort is closed to enrolment*

SOP 05: Consenting Pregnant Women

Version	Effective Date	Supersedes	Approved by	Date
1.0		None: New		

Purpose

This SOP describes how to obtain written informed consent from eligible pregnant women in both control and intervention sites

Scope

This SOP applies to all study staff who have direct involvement or oversight in consenting participants into the G-ANC sessions.

Overview:

Informed consent is a process by which an individual voluntarily expresses his/her willingness to participate in research, after having been informed of all aspects of the research that are relevant to her decision. Informed consent is rooted in the ethical principle of respect for persons. For the research project, oral and written informed consent must be obtained from all participants.

U.S. and site-specific regulations specify the elements of informed consent that must be conveyed to research participants through the informed consent process. It is the responsibility of the Principal Investigator, and his/her assigned staff, to deliver all required information to potential research participants. The responsibility for informed consent does not end with preparation of an adequate

informed consent form. It is also the responsibility of the site Principal Investigator and designated study staff to deliver all required information in a manner that is understandable to potential participants, assure that informed consent is obtained in a setting free of coercion and undue influence, ensure that the participant understands the information, and document the process.

Research Assistants (RA) will be responsible for informing all potential study participants about the research to obtain their written (i.e., signed) consent for participation. This informed consent MUST be obtained prior to participation in the group ANC. Consent forms will be available in English, Kiswahili and Hausa. The appropriate language to use during consent will be the one the potential study participant understands and communicates in best, eg. Hausa in Nigeria and Kiswahili in Kenya.

Procedure

These procedures must be carried out in full (to ensure that participants are sufficiently informed) and in a standardized manner (to ensure that all participants have the same information when making their decision about whether to participate in the G-ANC or not). The RA should:

1. Make sure s/he has adequate informed consent forms (ICFs) – at least 2 copies in English and 4 in Kiswahili or Hausa
2. Give the potential participant a chance to choose her preferred language (English, Kiswahili) or Hausa
3. Do not enroll if the participant cannot be questioned due to language barrier or hearing impairment, mental limitation, and physical limitation.
4. Read the consent form aloud and give a copy to the participant so she can read along if literate.
5. Continuously assess comprehension of participant in the course of consent administration and answer any questions she may have.
6. At the end, ask the potential participant again if she is willing to be in the study; anyone declining consent will be referred back to individual ANC care.
7. If she says YES, Obtain signed (written)consent:
    - Present 2 copies of the informed consent form to those who agree to be in the study, asking them to write their names, sign and date the form to signify her acknowledgement of having been informed about the procedures, risks and benefits and agreement to participate.
    - If participant is non-literate, obtain thumbprint in lieu of signature. The RA can print the participant's name and the date of consent.
8. RA to write his/her name,sign and date both consent forms as verification that the participant gave informed consent.

9. Present a signed copy of the consent form to the participant for their retention.

   NOTE:*If for some reason the participant does not wish to take her copy with her, file both together and make a note of this and give her a copy of contact information for the Study Coordinator, the PI and KEMRI-ERC in case she needs to contact any of them.*

   NB: RA to confirm that the consent form has been duly signed and dated.

10. Inform the newly enrolled study participant that, for confidentiality, the signed copy of their consent form will be stored in a locked file cabinet and stored separately from the rest of the information that they provide for the study.

11. Enter the participant details in the Consent section of REDCap. When you select "agree to participate" you will be prompted to enter contact information for the client.

12. Administer Baseline questionnaire as per SOP 07:*Administering surveys*

13. Handling signed hard copies of consent forms

    - Keep all signed copies of consent form in the folder provided for the same as you continue enrolling other participants
    - At the end of each day, hand over all signed consent forms to the PO or if provided lock them up in the cabinet at the facility.
    - At the end of each week, the PO will transfer all the consent form stored at the facility to the regional study office.

14. After a woman has consented, you will need to enter her name, contact information, and estimated date of delivery (EDD) in your study follow up diary (study document 116 for intervention sites and 117 for control sites). You will need this information in order to track appropriate follow up intervals for subsequent surveys.

SOP 06: Enrolling Service Providers

Version	Effective Date	Supersedes	Approved by	Date
1.0		None: New		

Purpose

This SOP describes how to enroll health providers in the G-ANC study.

Scope

This SOP applies to all study staff who have direct involvement or oversight in enrolling and administering the baseline questionnaire to group ANC facilitators in the intervention sites, namely research assistants.

Overview:

In control sites, all ANC providers should be consented and the baseline survey should be administered immediately afterward (5 questions). This is the only data that will be collected from them. In the intervention sites only those who have been (or will be) trained as group ANC facilitators should be consented.

Procedures

These procedures must be carried out in full (to ensure that participants are sufficiently informed) and in a standardized manner (to ensure that all participants have the same information when making their decision about whether to participate in the G-ANC or not). The RA should:

1. Make a list of all providers at your study site who need to be consented
    - In control sites, all ANC providers (verify currently providing ANC care at study site)
    - In intervention sites, all trained G-ANC facilitators
2. Provider consent forms (005) are available in English only. Ensure you have enough hard copies for the number of providers who need to be consented at your study site and number them serially.
3. Within the first 2 weeks of enrolling women on site, obtain consent from relevant health care providers. Cross off list as consent obtained and baseline survey complete
4. Follow the general procedures for consent similar to SOP 05, give one copy to provider and retain one copy with other consent forms. If any providers decline, contact the project officer immediately.

5. Administer Baseline questionnaire. Record ID = facility code _serial number from consent form. You will only be prompted to add contact information for providers in the intervention sites.

SOP 07: Administering surveys

Version	Effective Date	Supersedes	Approved by	Date
1.1	2/9/17	1.0	**Lindsay grenier**	**2/9/2017**

Purpose

This SOP describes when and with whom each survey is to be administered. It also outlines general principles to follow when administering standardized interviews.

Scope

This SOP applies to all study staff who have direct involvement or oversight in administering any questionnaires to study participants, namely research assistants.

Overview:

There are 2 sets of surveys, 1 for women who enter the study pregnant, and one for providers. Each set contains 3 surveys:baseline; Phase I (ANC); and Phase II (year after birth). Woman surveys are available in English, Kiswahili and Hausa. Provider surveys are available in English only. All surveys should be administered via REDCap using a study provided tablet. The software will automatically guide the administrator through any logic branching. Have copies of the questionnaire available for backup purposes only in the case that the tablet malfunctions or runs out of power.

Procedures

General principles for administering surveys

All baseline questionnaires will be administered one-on-one (i.e. one interviewer and one participant) by trained study RAs using REDCap on study provided tablet computers, in the language most comfortably spoken by the participant (English/Kiswahili/Hausa). Only use a paper questionnaire if the tablet has no battery power or you experience technical problems; once that is resolved, transfer the information into the tablet computer for upload to REDCap as soon as possible.

Always try to appear friendly, unhurried and calm. Before beginning, exchange pleasantries with the subject to put them at ease. Re-assure them that you are not there to judge them and that all your communication is 100% confidential, meaning under no circumstances will you share what they say with anyone. Let them know that you are happy to repeat any parts that are unclear or which they'd

like to hear again, or hear more slowly. However, do not try to interpret the questions for them, read them only as they are written. Pay close attention to instructions provided on the tablet/survey form. Some questions require you to read answer options for the subject to choose from while others are open ended questions but require you to match their answers to pre-determined categories (e.g. categories are shown but do not read them to subjects). In such cases, if they mention something that is not presented as an option, select “other” and type in their response.

Some of the surveys will require that you make arrangements to meet some women in their homes or providers in their clinics. Be aware of other distractions and offer to pause the interview if their attention is drawn elsewhere or they are needed by children/patients. If needed you can even return on another day to complete the survey. If this happens, choose “incomplete” for form status at the bottom of the survey form and click save. Provide the PO with timely updates on when an interview is rescheduled (incomplete).

Once you reach the end of the survey, review to make sure all questions have been adequately responded to, edit if necessary, and save the record as “complete” for the PO to review.

If a tablet is not sending surveys properly or if it is having other technical problems, the RA should contact the Project Officer/Project manager/Data manager immediately

Women surveys

All women’s surveys are administered to all enrolled women, in both control and intervention sites.

Baseline Survey – pregnant women

Baseline questionnaires are to be administered immediately (and only) after consent is obtained. Once you save and close the screening and consent form in REDCap, select “baseline” from the event grid.

Phase I Survey – recently delivered women

This survey is administered to each woman 3-6 weeks after delivery. This means that you will need to use the study follow up diary, tool 116, which include the estimated date of delivery for each woman. Some women deliver early and some late. Contact each woman the week of her estimated due date to see if she has delivered. See SOP 08 for additional details. Schedule a time and place for administering the survey which is convenient for the woman – remember, she’s a new mom!

Phase II Survey – 12 months after delivery

This survey should be administered one year after the actual delivery plus or minus 3 weeks (which will be known from the previous survey) and after the last group meeting.

Provider Surveys

Providers in control sites are ONLY asked the first 5 questions of the baseline survey. All remaining surveys are for ANC group facilitators only. Providers should be interviewed in a private location where their co-workers can not hear them. Remind them that their answers are completely confidential.

Baseline Survey – health care providers [Intervention and control groups]

Baseline questionnaires are to be administered immediately (and only) after consent is obtained. A new record ID will need to be generated for each provider when opening the baseline survey.

Phase I Survey – health care providers [Intervention group ONLY]

To be administered within a week after the last group ANC meeting for the last cohort has been held

Phase II Survey – health care providers [Intervention group ONLY]

To be administered within a week after the last group meeting in the year after birth has been held for the last cohort.

SOP 08: Contacting study participants for follow up

Version	Effective Date	Supersedes	Approved by	Date
1.1	2/9/2017	1.0	**Lindsay grenier**	**2/9/2017**

Purpose

This SOP describes the process for contacting study participants for follow up

Scope

This SOP applies to all study staff who have direct involvement or oversight in administering any questionnaires to study participants, namely research assistants.

Procedures

Make the first attempt to contact study participants the week of their due date. This means that you will need to know:

- When pregnant women delivered
- When the last group ANC meeting is being held at your study site (to interview G-ANC facilitators)
- When the last group PNC meeting is being held at your study site (to interview G-ANC facilitators)

For the recently delivered women survey, plan to try to interview women at 3 weeks postpartum to allow time in case it is difficult to connect with them.

Keep a log of your attempts to contact.

- Try to contact via phone over a period of two weeks, at least, but not limited to 5 times, at different times of the day each attempt. Log each attempt in tool 116

- If you are still unable to reach the subject, physically go to the address that was provided during enrolment which can be found in her screening and consent form in REDCap.

When calling and someone answers, first ask if you are speaking to the study participant (confirm the name). If not, explain who you are and why you are trying to reach that person (disclose the name). Also let the person who picks the phone know that the enrolled study participant had agreed to be contacted later. When you have the study participant on the phone, remind them of the upcoming interview and arrange a convenient time and place to meet. It is best not to do the women's surveys in the facility so that they do not feel obligated to answer questions positively. Offer to come to their home, or if that is not acceptable another place that is comfortable for the women.

As addresses and descriptions are sometimes imprecise, once there, if you still can't locate the subject ask neighbors or others who are around if they know the subject, or someone who lives nearby who recently had a baby [or has a 1 year old]. Ask if they know where she is and/or if she has moved. If you are able to locate the subject, offer to do the interview then, or another time in the near future, whichever works best for the woman.

SOP 09: Determining Baseline Facility Based Birth Rate

Version	Effective Date	Supersedes	Approved by	Date
1.0		None: New		

Purpose

This SOP details the process of data collection in order to determine the percentage of women starting ANC care <=24 weeks gestational age in the six months previous to the start of the study at each study site who went on to deliver at **any** facility.

Scope

The SOP applies to the research assistant and the project officers who will be extracting this information at the facility.

Procedures

1) Using the facility delivery rate log (tool 105), Extract data from ANC registry for the 6 months preceding enrollment in the study, starting with the oldest registry (from 6 months ago)
2) Keep the log in a secure location, such as locked with the consent forms.
3) Record information for every women who was <=24 weeks gestational age (GA) at the time of ANC1 (first ANC visit)
4) If EDD not recorded, use a GA wheel to calculate using GA and date from first ANC visit

5) Contact women with phone numbers 2-4 weeks after their EDD, If not able to reach, attempt to contact women at least 3 times at least a day apart and log attempts; try different times of day. Place an X in the column each time an attempt is made to contact which does not result in speaking to the woman
6) Once contact is made, administer survey 006a
7) Record place of delivery in the log
8) Enter every entry from log in REDCap (including those who did not have contact information or were not able to be contacted). Cross off # on the log as the data is entered in REDCap.

SOP 10: RDW follow up, survey and ANC Data Extraction

Version	Effective Date	Supersedes	Approved by	Date
1.1	**2/9/2017**	1.0	**Lindsay grenier**	**2/9/2017**

Purpose

This SOP defines the procedures for determining when to contact study subjects to set up a time and place to administer the recently delivered women survey (RDW, 3-6 weeks postpartum); administering the survey, and extracting ANC data for all recently delivered women (RDW) enrolled in the study.

Scope

This SOP applies to RAs who will administer the RDW survey and extract ANC data from records and the POs who support them.

Overview:

RAs will use tool 116 to create a follow up diary and log all contact attempts. They will aim to meet with each subject 3-6 weeks postpartum to administer the RDW survey and extract data. For all study subjects, data from ANC records will be collected in conjunction with administration of the RDW survey (3–6 weeks after delivery) from the following sources

- G-ANC registers: for intervention arm only, to be extracted prior to administration of RDW survey
- Mother-child booklets/ANC cards: all study participants, to be extracted at the time of RDW survey administration (and left with woman)
- ANC health facility register: all study participants if/as necessary when information is missing from mother-child booklet or ANC card

***Note**: For the Intervention sites, The RA will fill out data available in the G-ANC register prior to administering the survey.*

Procedures: preparing for follow-up

A report will be run for each facility detailing the following information for all subjects enrolled there:

- Study ID #
- Name, phone number, description of residence
- Estimated date of delivery (EDD) – from earliest to latest
- Gestational age on EDD based on GA recorded on date of enrollment

The RA will copy this information (minus study ID and description of residence) to the follow up diary, tool 116, from earliest to latest EDD, marking those records where the GA at EDD is <39 weeks or >41 weeks and providing an alternate EDD.

If enrollment has not been completed, the RA will subsequently check the report and add new subjects every 2 weeks.

RAs will contact enrolled participants for follow up interviews as per '**SOP 08: Contacting study participants for follow up'** and schedule an interview at a venue convenient to the participant (safe and accessible for the RA).

- If the GA at EDD is between 39-41 weeks, the first call attempt should be on the EDD to see if the woman has delivered.
  - If she has not yet delivered, call back in 3 weeks. Continue this every 3 weeks until she has delivered and you can schedule a time 3-6 weeks after delivery to meet with her
- Provide an alternate EDD when the GA at EDD is <39 or >41 weeks
  - For each week less than 40, add one week to the EDD (For example, if original EDD is Feb 1st, and the GA at EDD is 38 weeks, write down Feb 14th as the alternative EDD
  - For each week more than 40, subtract one week from the EDD.
  - For these women, follow the rules for BOTH EDDs. This means begin call attempts on the earlier EDD, and end attempts 1 month after the later EDD.
- If your call is not answered, record the attempt in the follow up diary. Attempts to reach via call should continue until 1 month past the EDD – each attempt should be recorded. Once the date is 1 month past the EDD and at least 5 call attempts have been made, attempt to contact the woman in her place of residence based on the description provided during consent and record the outcome in the diary.
  - If after at least 5 call attempts and a home visit the subject remains untraceable, open the RDW survey and complete the questions referring to contacts.

For intervention sites only

Data from the G-ANC register should be entered prior to meeting with the woman. Once the cohort has had their last meeting, get the group register from the provider and enter all data at

once. For each subject, open their data extraction record and complete section 1. In the case that women are known to have delivered prior to the last meeting, enter just their data from the register prior to following up with them.

Procedures: Meeting with the woman for RDW follow up

All participants:

- <u>Take your follow up diary with you</u>
- On meeting with the participant, the RA will introduce her/himself and remind the participant that during enrolment, she agreed to be interviewed 3-6 weeks after delivery.
- The RA will then:
  - Confirm that the participant is willing and ready to proceed with the interview.
  - If she says YES, the RA will administer the RDW survey
  - When the survey is complete, the RA will open the data extraction tool and begin with section 2 (5 questions).
    - If the Mother child book/ANC card is available, continue with data extraction from the source. Let the mother know that this may take a while and she is free to attend to other things if she wishes. When complete, proceed to submit the data as unverified
    - If the book is not available, thank the woman, and note the need to extract data from the ANC register

- When extracting data from the ANC register
  - Communicate your need to see the register with the facility and agree on a time you may have access to it
  - Open the data extraction tool for the subject in REDCap
  - Carefully review the register for records of the patient from the date of enrollment to the date of their delivery
  - Record information for each visit, including ANC1, as outlined in REDCap. When complete, proceed to submit the data as unverified

Section III: Standard operating procedures (SOPs) for project officers and other study staff

SOP 11: REDCap Access and Privileges

Version	Effective Date	Supersedes	Approved by	Date
1.0		None: New		

Purpose

To describe how to gain access to the REDCap system.

Scope

This SOP applies to all study staff and research assistants who have direct involvement or oversight in the Group ANC study on how to request for and gain access to the REDCap system.

Overview:

Each program manager (PM) will be responsible in informing the REDCap administrator, Charles Waka on the project team members who need to be added to the system. Roles for team members are described below. If you feel you need access to additional REDCap functions, contact the project director.

The roles must be defined before the project team members are added to the system.

Procedure

This procedure must be carried out in full and in a standardized manner to ensure the relevant project team members have access to the application based on their defined roles.

1. The PM to have a list of all project team members who need access to the REDCap system.
2. The PI to detail the roles which will be created in the project.
3. The PM to list which roles the users will be assigned as detailed above in the project.
4. The PM to send an email to the REDCap administrator requesting access for the users. The e-mail body should contain:
    - First Name of the users.
    - Last name of the users.
    - Email address of the users.
    - Roles in the project. User role.
5. The REDCap administrator to send a form which will be used to register the new users.
6. Roles and rights within the project is as follows:
    - Project Administrator (Charles Waka) o Add new users to project

- o Assign user roles o Update project with new data collection instruments o Update data dictionary of the project
  - o Access to logs to troubleshoot any error in submission
- Project Managers o Create and run data reports o Execute data quality rules o Export full data set
- Project Officers o Verify data submission by RAs o Edit collected data in conjunction with the RAs
  - o Add reports of data o View calendar to keep track of upcoming interviews for participants o Create, edit and execute data quality rules o Export de-identified data o Enter data on project weekly report
- Research Assistants o Collect data using the mobile app
  - o Save pdf of report linking study participants to study ID#s

7. Verification and review of the user roles to be done by REDCap administrator.
8. New users to receive e-mail notification within 2 working days granting them access to the system.
9. All users once granted access will not be deleted from the system for proper audit.

Relevant documents

REDCap definitive guide

SOP 12: Data Management

Version	Effective Date	Supersedes	Approved by	Date
1.0		None: New		

Purpose

The purpose of this SOP is to describe the process involved in managing and analyzing the G-ANC data and the persons involved in all statistical related aspects of the data.

Roles and Responsibilities

Principal investigator

The principal investigator is responsible for the overall conduct of the study and may delegate some responsibilities to other staff including the data manager and statistician.

Study statistician

The statistician assumes all the statistical aspects of the G-ANC study. S/he will be responsible for planning, monitoring, analysis and preparing of all statistical reports for the study. The statistician will work closely with the data manager to ensure data cleaning and validation is performed in a timely manner.

Data manager

The data manager is responsible for creating the electronic versions of the data collection tools and ensuring data collection is in accordance to the research plan. He will work to ensure incoming data are reliable and complete, cleaning and validation of data, exporting data to statistical software that will be used for analyses, and preparing data sets for analysis under the direction of the PI, coinvestigators or the study statistician.

Procedure

Electronic data capture

Data capture and entry shall be performed by research assistants who will undergo a 5 day training prior to the start of data collection. The training will entail familiarization with the study procedures and data collection instruments. They will also be trained on ethical issues involving human participants.

- The data capture software (REDCap) shall be programmed so that it reflects the layout and format of the hard copy questionnaires.
- The electronic version of the questionnaire will have validation, range and consistency checks to minimize data entry errors and provide high quality data.
- The project officers who will be supervising the research assistants will be on the lookout for errors and anomalies before submitting the data. No data will be submitted in case of any errors observed.

Data security and protection

- Data stored electronically on tablets or computers shall be backed up in a secure server daily.
- All equipment used to enter or access study data shall be password protected and will have security software installed. In addition, each REDCap user will be required to login using their personal ID to access the data.
- Data sets that will be analyzed will not contain personal identifiers and will only be accessed through the study data manager with the permission of the PI.
- All person-identifiable data must be treated in a confidential manner and shall be stored or transmitted on media with encryption.
- The contact information of the clients which will have been recorded in the tablets will be converted into pdf format and stored in the tablets.

- Paper or computer records shall only be accessible to the minimum and necessary number of people. Other than the study manager, all other persons must seek authorization from the study PI before they can gain access to the data
- At the end of the data collection, data shall be locked to prevent alterations by unauthorized persons. Only the study manager will have full access to the database

Data cleaning

- Data cleaning will be carried out progressively during the study implementation period as the data is streamed from the field. This will help to flag out inconsistencies and corrections will be made where necessary.
- Concerted efforts will be made to minimize missing data during data collection and cleaning. In cases where the outcome of interest is missing, the study supervisors will make efforts to contact the ANC mothers to verify the missing information.
- Data cleaning annotated programs will be prepared using either STATA or R statistical software and will be stored for future reference. An audit trail back to the master data set will be kept.
- The raw and master dataset (before manipulation) will be preserved and all manipulations will be carried out on a copy of this dataset. The raw data sets will be archived before cleaning and any subsequent versions after cleaning and during exportation for analysis will also be archived.

Data analysis

Creation of data analysis files

During the time of analysis, the data manager will work with the statistician to export the data from the database in a format that can be analyzed with a standard statistical software (STATA or R). All data will be kept in a password protected directory accessible to the statistician and the data manager. In case of queries, the data manager will be conducted for resolution and a corrected database will be availed if need be. The statistician will develop annotated programs for data analysis and manipulation that will be provided to the PI at the end of the analysis. The program will also include details about recoding, labeling and scoring of data.

Analysis programs

All analysis will be implemented according to the analysis plan outlined in the research plan and any changes will have to be justified. For all the analysis, adequate annotation will have to be provided by the statistician.

Statistical reporting

The statistician will usually draft the results section and tables in the study reports and manuscripts for publications. He will also provide support in drafting the methodology, discussion and conclusions sections.

SOP 13: Data Monitoring and Quality Assurance, data verification and submission by POs

Version	Effective Date	Supersedes	Approved by	Date
1.0		None: New		

Purpose

This SOP describes the data monitoring plan for the G-ANC data from the data collection point to ensure data is complete, accurate and submitted in a timely manner.

Scope

This SOP applies to all study management staff who have direct involvement or oversight on data collection in the G-ANC study.

Procedures

Specification of the data to be collected

Data for the G-ANC study will be collected from the recruited study participants using the REDCap application which will be pre-installed on tablets. Data collection will take place at various points in time as described in SOP 7: Administering surveys, and SOP 10: data extraction. The point of data collection will be at the health facilities where recruitment and consenting will take place and at the location of subjects choosing for follow up surveys.

<u>Plans for secure storage of data with limited data access</u>

The tablets to be used for data collection will be password protected. The password will be known by the database administrator and the research assistant only and, will not be shared with anyone else not authorized by the principal investigator. In addition, each REDCap user will be required to login using their personal ID to access the data.

Data transmission will be via a secured network to the central database which is located in JHPIEGO Nairobi office. The central database is also password protected and only the database administrator will have the permission to download the data.

Verification of data

The project officers will be responsible for reviewing all the submitted data under their jurisdiction from their laptops on a daily basis. The purpose of reviewing the data is to check for data completeness.

Data completeness: data completeness entails all fields where a response is required being duly filled. If the response was not obtained, the reason should be provided why it is missing.

Checking for completeness of submitted data

The project officers will be trained on how run data quality checks on REDCap. They will check for the following during these data quality checks:-

- That participant has been assigned a study ID.
- All fields have been recorded with relevant information as required o In case of missing data, the supervisor will work with the data collector and complete the missing information. If the missing information is due to refusal by the participant to answer the question, the supervisor will mark the questionnaire as complete and write a note to that effect to be kept on file. If getting the missing record is not feasible, the PO will mark it as complete and make a note also.
- In case a respondent discontinues an interview, the record will be marked as incomplete.
- The completeness of a record is to be understood as where the PO has established all records have the necessary information correctly filled and all missing information can be accounted for with notes are provided.

When the data has been marked complete and is received at the JHPIEGO Nairobi office, the study data manager will perform some consistency checks to identify any potential problems. For example the data manager will review each question individually and establish its completeness status. In case inconsistencies are found, the following actions will be taken.

- The PI of his designee will be notified and remedial measures taken to eliminate similar errors in the future. This may include:

  o Retraining of the staff involved o Retraining of all field staff undertaking a similar assignment o Revision of the applicable SOP or drafting of a new SOP

Contact information of clients and scheduling of follow up appointments

The research assistants will generate pdf reports from the tablets with all the follow up contact information. The POs will also be able to generate the same information from their laptops and they will use this information together with the recorded EDD to schedule appointments when the clients are supposed to be contacted by the RAs in an event calendar.

A few days prior to the appointment, they will remind the research assistant of the scheduled follow up.

Performing data quality checks on REDCap

The project officer will follow these steps in generating data quality reports from the report.

1. On the REDCap G- ANC page, scroll down on your screen (bottom left side), you'll see the Reports Module Labelled 1below. Under the Reports Module, there are pre-generated reports listed.
2. To create a new report, click on 'Edit reports' labelled 2 below.

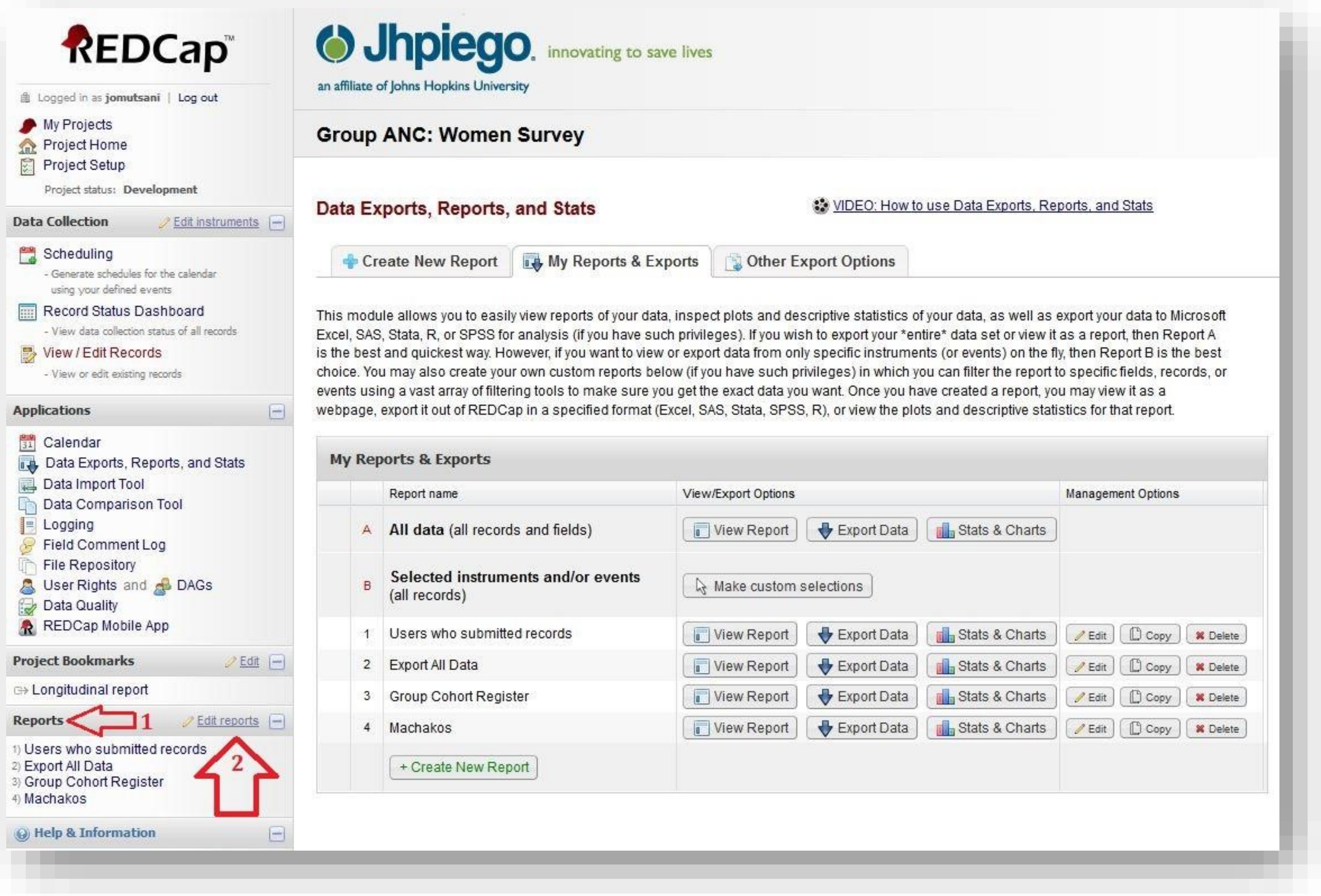

3. Then click on 'Create Report' labelled 3 and Name it as shown on the arrow labelled 4below.

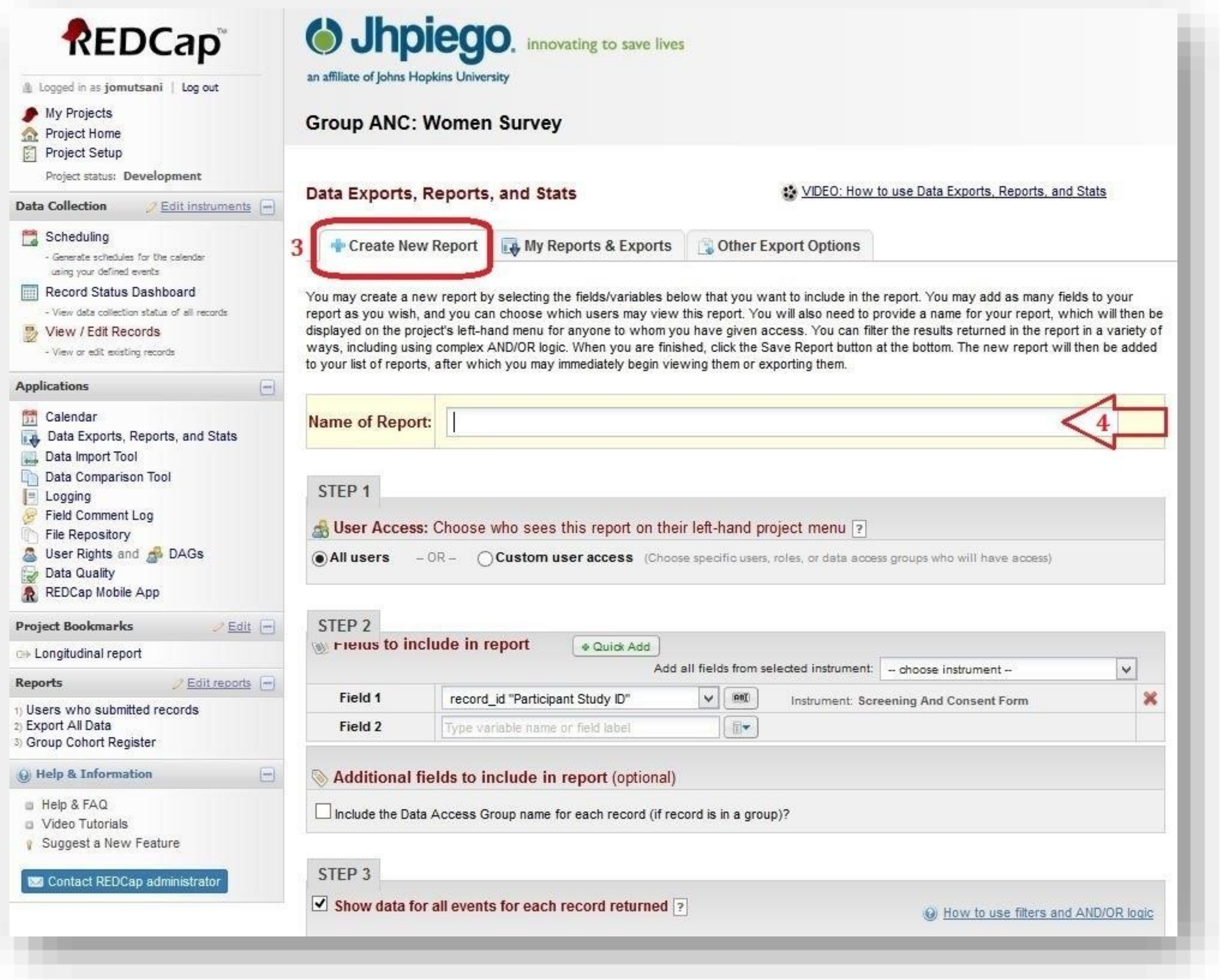

4. Under Name of report, there are steps included to customize your selection. Skip step 1 and go to step 2 and step 3.

5. Click on 5 to activate the drop down list of fields, which will present a downward arrow as shown in 6, whereby you can choose the fields to be displayed in your report. Click on 'Save Report' labelled 7.

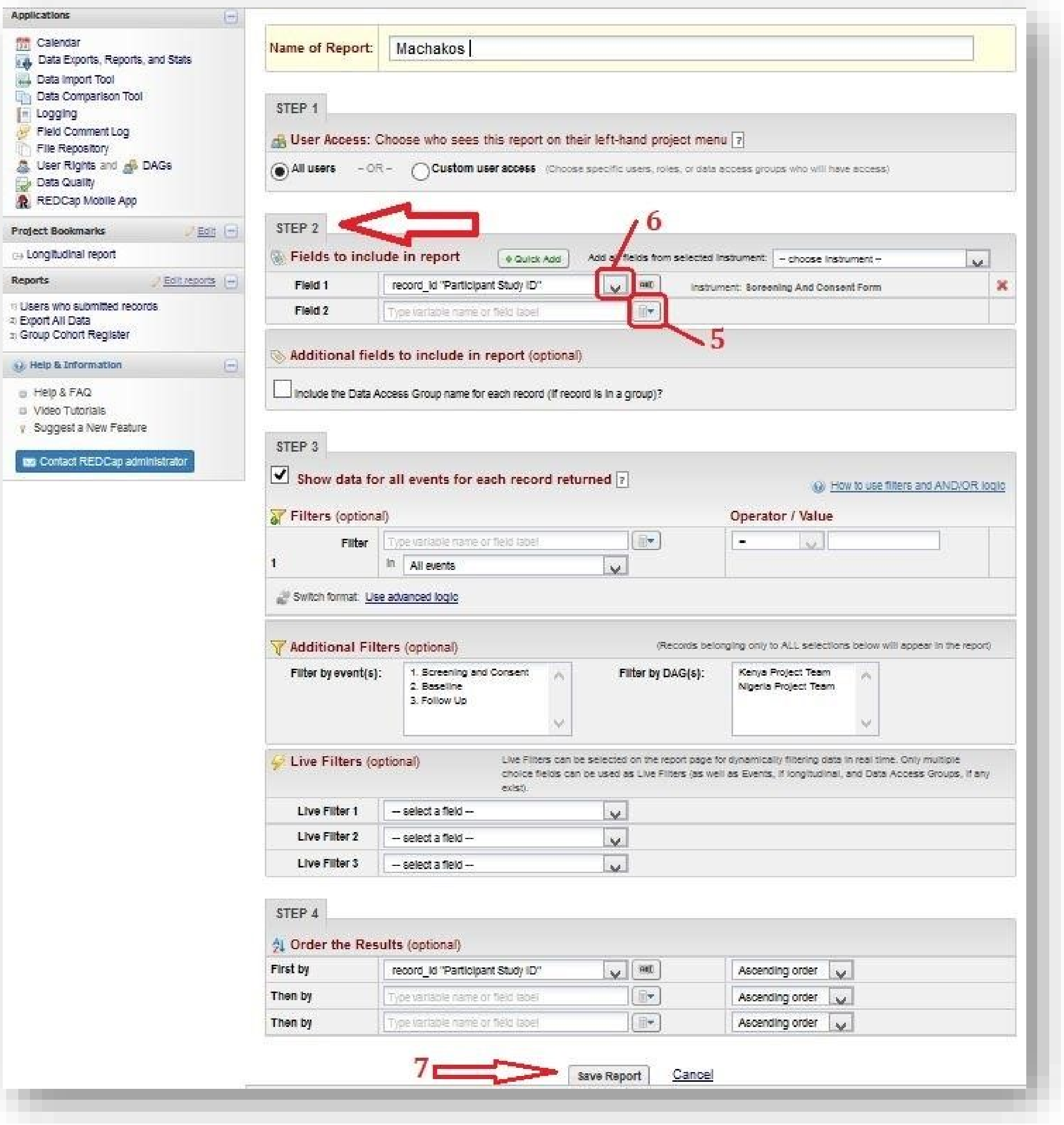

6. The below notification will be displayed. Click on the 'View Report' button labelled 8 to see the list of fields you chose for your whole data set.

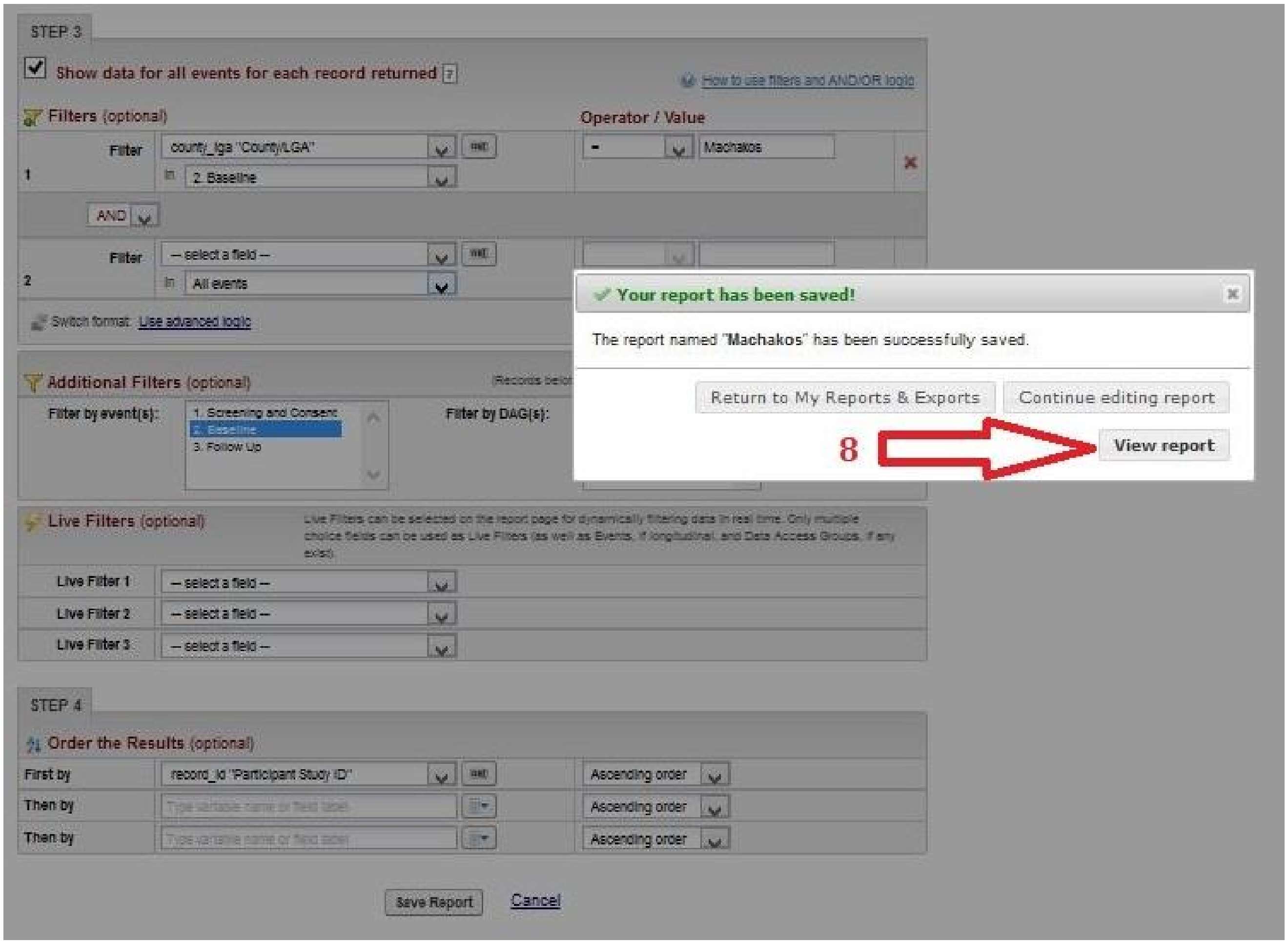

7. The date is now displayed for the records according to the fields you chose. To easily view missing data on your records, click on the 'stats & chart' button pointed below.

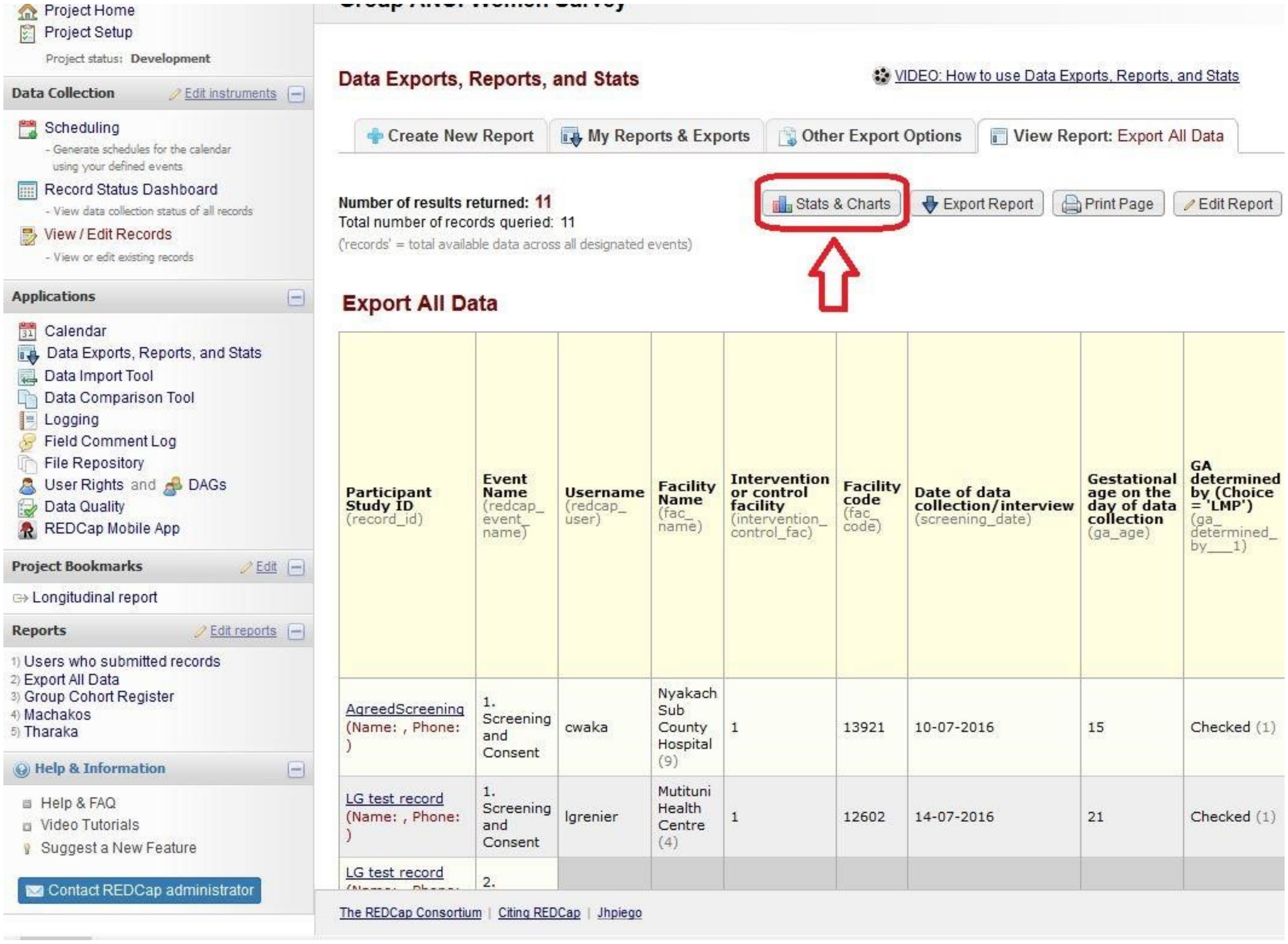

8. The data will be displayed in tables and Missing fields will also be indicated as shown below

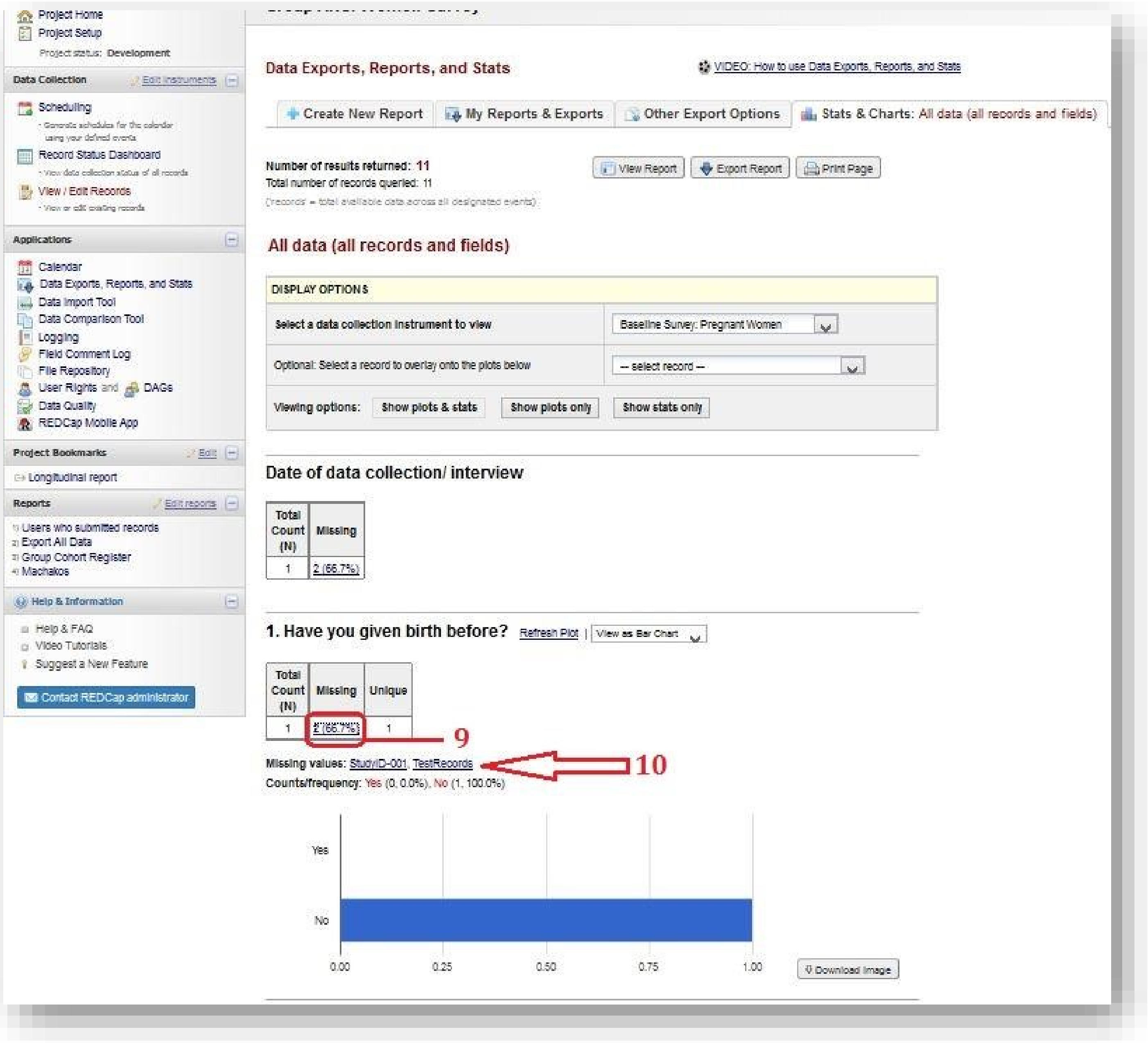

(9). Click on the figures in the table labelled 9 and you will be able to see the list of records with missing values as shown in 10. Clicking on the record ID takes you to the record itself, where you can be able to make edits if necessary.

SOP 14: Facility audits prior to enrollment (all sites) and commencement of group meetings (intervention sites)

Version	Effective Date	Supersedes	Approved by	Date
1.0		None: New		

Purpose

This SOP defines the procedures for carrying out facility audit prior to enrolment and also prior to holding the first G-ANC session at the G-ANC study facilities.

Scope

This SOP applies to all study staff who will have responsibility of carrying out facility audit at study facilities, namely the project officers.

Overview:

Facility audits will be carried out at two levels; (i) before the actual study implementation kicks off and in intervention facilities, prior to holding the first group ANC. It is important that these audits take place well in advance of planned start dates so that remediation, if needed, can be carried out.

Procedures

Facility audit prior to commencement of enrollment (all sites)

1. The PO will generate a schedule for completing all facility audits prior to commencement of the study and will liaise with the facility in-charge to carry out audits according to the schedule.
2. The PO will use checklist102: facility audit prior to commencement of enrollment, to assess facility readiness. If any areas are found lacking, the PO will alert the PM immediately and make a remediation plan. This exercise should be completed at least two weeks before the anticipated enrolment start date.
3. Once the facility is found to have met all criteria, the PO will sign off and proceed to commence enrollment of study participants.
    a. During this process the PO will update the RA, PM, and facility administration of intended start date as well as any alterations to that date
    b. The PO will ensure that all providers are clear re: start date and begin filling out Part I of screening form for all new ANC clients

Facility audit prior to commencement of group ANC meetings (intervention only)

1. The PO will liaise with the facility in-charge at each facility and make an appointment for a visit to check each intervention study facilities' readiness for the G-ANC sessions no later than two weeks into enrollment.
2. The PO will use checklist 103: facility audit prior to commencement of group meetings, to assess facility readiness.
    a. If any areas are found lacking, the PO will alert the PM and PI immediately and make a remediation plan. The PI will ultimately make the final decision as to whether the first cohort can commence as planned.
3. If it is confirmed that the facility is to be ready for group ANC, the PO will confirm G-ANC cohort 1 start date as scheduled.

This assessment will be repeated on a monthly basis as part ongoing supportive supervision to intervention sites

SOPs 15–16: Site visits and supportive supervision

Purpose

These SOPs describe how often site visits should be undertaken for control versus intervention sites as various points in the study as well as what activities are to be undertaken at those times including supportive supervision of RAs, ANC providers, and G-ANC facilitators. **Scope**

This SOP applies to project officers.

Overview:

At all sites POs will be expected to monitor RAs in 3 ways:

- By verifying data quality and follow up via REDCap (daily)
- By visiting on site (weekly for intervention sites; every 2 weeks for control sites)
- Via phone (the weeks control sites are not visited in person + as needed to resolve data quality issues etc)

All supportive supervision duties should be done at every on-site visit. Those that can, should be done via phone for the control sites the week's they are not visited. Site visit schedules and supportive supervision activities vary both by control or intervention, and by point in the study.

During Enrollment:

- Intervention sites should be visited weekly
- Control sites should be visited every other week
- All RAs should have the same supportive supervision
- All ANC providers should have the same limited supportive supervision related to GA assessment, screening of clients, and data quality in registers

During Follow up:

- Once enrollment ends, visit or call control sites monthly to assess SP and dipstick availability
- Once data collection of Phase I - recently delivered women survey commences, see each RA at least once every two weeks, and speak with via phone weekly.

In intervention sites:

- Attend majority of group meetings
- Use checklist 103: facility audit prior to commencement of group ANC meetings at least once a month
- Provide supportive supervision using provided tools before, during, and after meetings

SOP 15: Providing supportive supervision to Control sites

Version	Effective Date	Supersedes	Approved by	Date
1.0		None: New		

Procedures

Enrollment Period

Perform site visits every two weeks during enrollment

Check supplies

- ☐ Check that copies of all necessary forms, in appropriate language(s) are available (always have extra with you when doing site visits)
- ☐ Mother Child books and/or ANC cards available for ANC 1 clients
- ☐ 001 Screening form; Part 1, pre numbered (1 per expected ANC1 client)

- ☐ 002 Study script (1 per expected ANC1 client <24 weeks)
- ☐ 003 Consent (2 per expected eligible client)
- ☐ 001 screening form in it's entirety as backup (a few copies)
- ☐ 006 baseline survey as backup (a few copies)
- ☐ Verify RAs have adequate supply of low dose folic acid IFA and LLIN to distribute to study participants; verify HIV test kits available in ANC
- ☐ Verify SP and limited dipsticks available in ANC
- ☐ Verify functioning of tablet and backup battery

Check Enrollment

- ☐ Assess enrollment numbers for the week, and in total
- ☐ Fill out enrollment log and submit to PIs
- ☐ Alert site PI if numbers below or above expected (approx. 14/month; 54 total over enrollment period)

Provide supportive supervision to RAs

- ☐ Review requirements of informed consent; collect signed consent sheets and keep in locked cabinet
- ☐ Verify from facility staff that RA has attended during all ANC clinic hours
- ☐ If possible, observe RA during client interaction: screening; consent; baseline survey
- ☐ Ensure scripts are followed; RA is professional and friendly; RA assesses for understanding before consent finalized
- ☐ Verify correct procedure is being used to assign subject ID #s in REDCap
- ☐ Inquire as to any difficulties or questions
- ☐ Carry out spot checks (unannounced) to ascertain RAs are on site as expected.

Provide supportive supervision to ANC providers

Do NOT mentor ANC providers in FANC or engage in quality improvement activities around antenatal care. This will confound study results. Only support providers in the following (in addition to support specific to group care)

- ☐ Accurate estimation of gestational age
- ☐ Consistent and accurate completion of Part 1 of screening form
- ☐ Consistent and accurate completion of ANC registry data

Follow-up period

Once enrollment ends, visit or call control sites monthly to assess SP and dipstick availability

Once data collection of Phase I - recently delivered women survey commences, see each RA at least once every two weeks, and speak with via phone weekly.

☐ Pro-actively ensure that follow-up surveys are being administered at the appropriate times o Using REDCap and the cohort logs, for each cohort, have a record of every subject's expected EDD. Start reminding RAs of upcoming RDW surveys around the time the first woman in a cohort is expected to deliver.
    - o Once women begin delivering, run weekly reports in REDCap to see if subjects who are expected to have delivered 3 weeks ago have had the RDW survey administered yet.

☐ Observe each RA administer each follow-up survey at least once to ensure basic principles are understood and followed.

☐ Ensure adequate follow-up attempts (subject contact) are being made and logged appropriately.
    - o Examine their contact attempt logs and discuss any subjects who have been lost to follow-up. Decide if any more attempts should be made to find them, and if so, what.

SOP 16: Providing supportive supervision to Intervention sites

Version	Effective Date	Supersedes	Approved by	Date
1.0		None: New		

Procedures

Project officers should remain in close contact with group facilitators at intervention sites. Their purpose in the intervention sites is to assist the facility staff both in maintaining study standards and in gaining competency in providing group based care. Key supportive tasks include supervising and assisting in:

- Scheduling group meetings
- Identifying where and how to set up group space
- Ensuring all necessary (study supported) materials are available for ANC1 and group meetings
- Promoting good facilitation skills during group meetings
- Ensuring fidelity to the model, including continuity of facilitators for each cohort

During all site visits

Use checklist 103: Facility Audit prior to commencement of group ANC meetings to:

- ☐ Ensure all necessary (study supported) materials and supplies are available for group meetings
- ☐ Confirm continuity of facilitators within each cohort
- ☐ Confirm scheduling of group meetings is complete, clear to all and follows 1 month intervals for each cohort
- ☐ Confirm patients are being reminded of group meeting times and facility staff has needed talk time (provided by study)

During Enrollment Period

Perform site visits every week during enrollment

Check Enrollment

- ☐ Assess enrollment numbers for the week, and in total
- ☐ Fill out enrollment log and submit to PI or his designee
- ☐ Alert site PI if numbers below or above expected (approx. 14/month; 54 total over enrollment period)
- ☐ Assess current cohort enrollment and scheduling
- ☐ Spot check cohort enrollment in REDCap with paper log to ensure subjects are being recorded on cohort log
- ☐ If <8 have been enrolled and cohort start date is within a week, meet with facility staff to discuss cancelling and determine plans for next cohort start date. Contact site PI for assistance.
- ☐ If >=13 have been enrolled, assess current start date for the following cohort and assess need to move up
- ☐ If a cohort has closed (15 members is the max.), ensure RA has communicated this with facility staff and given them a copy of the cohort log
- ☐ Ensure scheduling is in place for current cohort's meetings and next cohort start date

Provide supportive supervision to RAs

- ☐ Review requirements of informed consent; collect signed consent sheets and keep in locked cabinet
- ☐ Verify from facility staff that RA has attended during all ANC clinic hours
- ☐ If possible, observe RA during client interaction: screening; consent; baseline survey
- ☐ Ensure scripts are followed; RA is professional and friendly; RA assesses for understanding before consent finalized
- ☐ Verify correct procedure is being used to assign subject ID #s in REDCap
- ☐ Inquire as to any difficulties or questions
- ☐ Carry out spot checks (unannounced) to ascertain RAs are on site as expected.

Provide supportive supervision to ANC providers

Do NOT mentor ANC providers in FANC or engage in quality improvement activities around antenatal care. This will confound study results. Only support providers in the following (in addition to support specific to group care)

- ☐ Accurate estimation of gestational age
- ☐ Consistent and accurate completion of Part 1 of screening form
- ☐ Consistent and accurate completion of ANC registry data

During Follow-up period

Once data collection of Phase I - recently delivered women survey commences, see each RA at least once every two weeks, and speak with via phone weekly.

- ☐ Pro-actively ensure that follow-up surveys are being administered at the appropriate times
  - o Using REDCap and the cohort logs, for each cohort, have a record of every subject's expected EDD. Start reminding RAs of upcoming RDW surveys around the time the first woman in a cohort is expected to deliver.
  - o Once women begin delivering, run weekly reports in REDCap to see if subjects who are expected to have delivered 3 weeks ago have had the RDW survey administered yet.
- ☐ Observe each RA administer each follow-up survey at least once to ensure basic principles are understood and followed.
- ☐ Ensure adequate follow-up attempts (subject contact) are being made and logged appropriately.
  - o Examine their contact attempt logs and discuss any subjects who have been lost to follow-up. Decide if any more attempts should be made to find them, and if so, what.

During group meetings

POs should attend as many meetings as possible, a majority for each cohort. Remember to bring blank copies of the supportive supervision tools to each meeting: debrief, fidelity checklist, and individual observation.

<u>Before group meetings</u>

- ☐ Watch facilitators prepare and prompt if necessary to remind them of anything they've forgotten.
- ☐ Make sure co-facilitators have planned who is doing what parts.

<u>During group meetings</u>:

- ☐ Explain to the group who you are and why you are there. Sit in the circle and participate, but do not take over facilitation.
- ☐ Use the "debrief with facilitators" template to take notes on what goes well and what could be improved.

- ☐ Also complete the fidelity checklist and individual observation (one for each facilitator)

<u>After group meetings:</u>

- ☐ First ask the facilitators to fill out the “individual observation”.
- ☐ While they are doing that, review the group register and normal ANC register for completeness, and remember to discuss as part of the debrief.
- ☐ When they are done (before discussing), use the “debrief with facilitators” tool to debrief the session.
  - o First ask them what they thought went well, then reiterate which of the things they named you also felt went well and add any additional comments.
  - o Next asked them what they learned and what they want to work on or try differently next time. Again, let them answer first and then add your comments.
  - o Use information from the fidelity checklist and individual observation to inform your comments.
  - o At the end ask if they’d like to discuss their self-reflection.

Section IV: Standard operating procedures (SOPs) for group ANC facilitators

SOP 17: Conducting Group ANC

Version	Effective Date	Supersedes	Approved by	Date
1.0		None: New		

Purpose

This SOP defines the procedures for conducting group ANC meetings as part of the Group ANC study

Scope

Facility staff who have undergone the study provided 5 day training to be group ANC facilitators at study sites

Overview:

ANC providers in intervention sites will be trained to facilitate group ANC meetings. Their primary responsibilities are to adhere to the model as defined during training and fill out group registers in addition to normal record keeping.

Procedures

Safe keeping of group ANC materials

Materials provided by the study, for use with the groups will be kept in a separate, safe location. POs will work with group facilitators to ensure they have all needed materials. SP and urine dipsticks provided for the study should remain with other group materials and used only for study participants.

Stability of cohorts

Group ANC facilitators will ensure that no more than 15 women are included in any one cohort (group) and that new people do not join in the middle of a group. Only study participants are to be admitted to study cohorts. Each cohort should have a minimum of two co-facilitators who are present at every group meeting to promote trust and group cohesiveness. Group facilitators will ensure that reminder calls are made to women for upcoming group meetings.

Conducting meetings

Meetings are to be conducted by the trained facilitators as outlined in each meeting guide with attention to items on the fidelity checklist. Facilitators may choose to do different activities as long as they serve the same purpose and are participatory. Meetings are to be conducted in circles, with everyone seated including all facilitators. Where possible, clinical assessments should take place

within the group space. Every meeting should include: self-assessments, provider assessments, covering key topics; making plans and time to socialize.

Record keeping

During private consultations providers will records assessments and care in the mother child book; ANC card; normal ANC register; and group ANC register as appropriate. There will be one group ANC register for each cohort. This will be given to the project officer after the last meeting of the cohort.

Quality assurance

Providers, together with project officers will use 3 tools for quality assurance: debriefing form; fidelity checklist; and self-reflection checklist. Only the fidelity checklists will be added to the study database.
