# Supplementary material for: Group versus individual antenatal and first year postpartum care: Study protocol for a multi-country cluster randomized controlled trial in Kenya and Nigeria
Source: Gates Open Res. 2019 Jul 4;2:56. Originally published 2018 Nov 5. [Version 2] doi: 10.12688/gatesopenres.12867.2 (PMC6350506; doi:10.12688/gatesopenres.12867.2)
Supplement: Supplementary file 1 [file gatesopenres-2-14160-s0000.tgz › 72242879-e244-48c8-a71c-b86050fb7704_Supplemental_files-_Study_manual,_Data_tools,_and_recruitment_,materials.zip!/Supplemental files- Study manual, Data tools, and recruitment ,materials/Baseline survey_ANC provider.pdf]

Baseline Survey Facility Staff

Enter the PARTICIPANT STUDY ID ________________________________

Data Collector ________________________________

Date and Time of record creation - Data quality check ________________________________

Baseline Survey - Latitude ________________________________

Baseline Survey - Longitude ________________________________

Health Facility Name

- ◯ ------MACHAKOS FACILITIES----------------
- ◯ Mitaboni Health Centre
- ◯ Kaviani Health Centre
- ◯ Muumandu Health Centre
- ◯ Mutituni Health Centre
- ◯ Nguluni Health Centre
- ◯ Matungulu Health Centre
- ◯ Matuu District Hospital
- ◯ Kangundo District Hospital
- ◯ Mlolongo Health Centre
- ◯ Athi River Health Centre
- ◯ Ekalakala Health Centre
- ◯ ------KISUMU FACILITIES---------------------
- ◯ Migosi Sub County Hospital
- ◯ Rabuor Sub County Hospital
- ◯ Nyakach Sub County Hospital
- ◯ Kombewa Sub County Hospital
- ◯ Sondu Health Centre
- ◯ Nyangande Health Centre
- ◯ Masogo Sub County Hospital
- ◯ Katito Sub County Hospital
- ◯ Ahero County Hospital
- ◯ Nyahera Sub County Hospital
- ◯ ------THARAKA NITHI FACILITIES--------------
- ◯ Chaikariga Health Centre
- ◯ Tunyai Health Centre
- ◯ Kibunga Health Centre
- ◯ Gatunga Health Centre
- ◯ Kibugua Health Centre
- ◯ Mpukoni Health Centre
- ◯ ------NASARAWA FACILITIES----------------
- ◯ PHC Nasarawa Eggon
- ◯ PHC New Karu
- ◯ PHC Tsonhon Kasuwa
- ◯ PHC Karshi I
- ◯ PHC Agyaragu Yakubu
- ◯ MPHC Baknyo
- ◯ PHC Akruba
- ◯ PHC Masaka
- ◯ PHC Lafia East
- ◯ PHC Doma Road, Lafia
- ◯ PHC I Garaku
- ◯ PHC Barkin Abdullahi (B.A.D)
- ◯ PHC Tundungwadara
- ◯ GH Akwanga
- ◯ Medical Centre, Mararaba
- ◯ PHC Mararaba
- ◯ PHC Kofar Pada
- ◯ PHC Mana, Emir's Palace, Lafia
- ◯ PHC Angwan Waje
- ◯ PHC Azara

County/LGA

____
(1 - Machakos County; 2 - Kisumu County; 3 - Tharaka Nithi County; 4 - Nasarwa (LGA))

Health Facility code

____

Intervention or control facility?

____
(1 - Intervention site; 2 - Control site)

Date of data collection/ interview

____
(DD-MM-YYYY)

First and Last Name of data collector ________________________________

Name of service provider ________________________________
(For Intervention sites only)

Phone number of service provider ________________________________
(For Intervention sites only)

1. Age? ________________________________

2. Sex?
- ◯ Male
- ◯ Female

3. Clinical/professional designation
- ☐ Midwife
- ☐ Nurse
- ☐ Physician
- ☐ Clinical officer or medical officer
- ☐ Community health extension worker (CHEW)
- ☐ Community health volunteer/worker (CHW/CHV)

(Check all that apply)

4. How many years have you had this designation? ________________________________

5. How many years have you been providing ANC care? ________________________________

THANK THE PARTICIPANT AND END THE INTERVIEW

Intervention Only: ANC providers

6. Do you currently provide ANC care?
- ◯ Yes
- ◯ No

(IF THE PARTICIPANT SAYS NO, END SURVEY AND THANK PARTICIPANT)

THANK THE PARTICIPANT AND END THE INTERVIEW

7. Please give me a few words to describe your feelings towards providing ANC care ________________________________

8. Thinking specifically about when you provide antenatal care, do you agree, disagree or neither agree nor disagree with each of these statements?

a. I am able to provide the level of quality in antenatal care that I would like to provide.
- ◯ Agree
- ◯ Disagree
- ◯ Neither Agree or Disagree

b. During antenatal care I have enough time with my patients
- ◯ Agree
- ◯ Disagree
- ◯ Neither Agree or Disagree

c. During antenatal care I am able to answer questions in a way my patients understand
- ◯ Agree
- ◯ Disagree
- ◯ Neither Agree or Disagree

d. I feel respected by my ANC patients
- ◯ Agree
- ◯ Disagree
- ◯ Neither Agree or Disagree

e. During antenatal care there is time to teach important information about how women can keep themselves and their babies healthy

- ◯ Agree
- ◯ Disagree
- ◯ Neither Agree or Disagree

f. I would recommend to a friend to go to my clinic for antenatal care

- ◯ Agree
- ◯ Disagree
- ◯ Neither Agree or Disagree

g. Sometimes I worry that patients leave their visits with unanswered questions or confused about some things

- ◯ Agree
- ◯ Disagree
- ◯ Neither Agree or Disagree

h. I am always able to be respectful towards my patients

- ◯ Agree
- ◯ Disagree
- ◯ Neither Agree or Disagree

i. I find providing antenatal care highly satisfying

- ◯ Agree
- ◯ Disagree
- ◯ Neither Agree or Disagree

9. On a scale of 1 - 5, with 1 being extremely dissatisfied and 5 being extremely satisfied, how would you rate your current job satisfaction, specifically in relation to providing ANC care?

◯ 1 ◯ 2 ◯ 3 ◯ 4 ◯ 5

10. On a scale of 1 - 5, with 1 being not at all and 5 being a lot, how much do you enjoy providing antenatal care?

◯ 1 ◯ 2 ◯ 3 ◯ 4 ◯ 5

11. What are your key frustrations providing ANC care?

- ☐ Too many patients
- ☐ Not enough time
- ☐ Not enough equipment or supplies
- ☐ Don't know the patients
- ☐ Women don't come early enough
- ☐ Women don't return for visits
- ☐ Don't feel prepared/trained
- ☐ Other

(Check all that apply. Ask "any other frustrations?". DO NOT PROMPT)

Other specify __________________________________

Feasibility

12. Do you think it's feasible to implement a group based care model in your facility?

- ◯ Yes
- ◯ No
- ◯ Not sure

For all responses: Why? ____

13. Do you personally want to provide group based care?

- ◯ Yes
- ◯ No
- ◯ Not sure

For all responses: Why? ____

Any other comments?

____

THANK THE PARTICIPANT AND END THE INTERVIEW
