# Supplementary material for: Group versus individual antenatal and first year postpartum care: Study protocol for a multi-country cluster randomized controlled trial in Kenya and Nigeria
Source: Gates Open Res. 2019 Jul 4;2:56. Originally published 2018 Nov 5. [Version 2] doi: 10.12688/gatesopenres.12867.2 (PMC6350506; doi:10.12688/gatesopenres.12867.2)
Supplement: Supplementary file 1 [file gatesopenres-2-14160-s0000.tgz › 72242879-e244-48c8-a71c-b86050fb7704_Supplemental_files-_Study_manual,_Data_tools,_and_recruitment_,materials.zip!/Supplemental files- Study manual, Data tools, and recruitment ,materials/Baseline Survey-PregnantWomen.pdf]

Baseline Survey: Pregnant Women

PARTICIPANT STUDY ID ______________________

Date and Time of record creation - Data quality check ______________________

Baseline Survey - Latitude ______________________

Baseline Survey - Longitude ______________________

Date of data collection/ interview ______________________ (DD-MM-YYYY)

1. Have you given birth before?

[Kin taba haifuwa?]

○ Yes [Eh]
○ No [A'a]

WOMEN WITH PREVIOUS BIRTH

2. What was the date of your last birth? ______________________ (DD-MM-YYYY)

[Wace rana kika yi haifuwarki ta karshe?]

3. Did you attend antenatal care during your last pregnancy?

[Kin je awon ciki lokacin da kina da cikinki na baya?]

○ Yes [Eh]
○ No [A'a]

SATISFACTION

4. Thinking about the antenatal care you received during your last pregnancy and as best as you can remember, do you agree, disagree or neither agree nor disagree with each of these statements.

[Bisa ga taimakon da kika samu lokacin won cikinki na baya ki nuna amincewarki ko rashin amincewarki da ra'ayoyin nan]

a. I was greeted warmly

[An karbe ni da fara'a]

○ Agree ○ Disagree ○ Neither agree nor disagree

b. I was able to ask questions and they were answered in a way I understood

[Na iya yin tambayoyi kuma na fahimci amsoshin da aka ba ni]

○ Agree ○ Disagree ○ Neither agree nor disagree

c. I learned important information about how to keep myself and my baby healthy

[Na koyi muhimman hanyoyin da zan bi don kare lafiyata da lafiyar jaririna]

◯ Agree ◯ Disagree ◯ Neither agree nor disagree

d. I left antenatal care visits with questions or confused about some things

[Na bar zuwa awon ciki don abubuwa da yawa ban fahimce su ba]

◯ Agree ◯ Disagree ◯ Neither agree nor disagree

e. The provider did not show me respect or act in respectful ways

[Mai ba da renon ciki bai mutunta ni ba kuma bai nuna mutunci ba]

◯ Agree ◯ Disagree ◯ Neither agree nor disagree

f. During this pregnancy I would be happy to receive care similar to what I received during my last pregnancy

[A wannan cikin zan yi murna idan zan sami kulawa kamar yadda na samu lokacin cikina na baya]

◯ Agree ◯ Disagree ◯ Neither agree nor disagree

g. I would recommend to a friend to go to the same place for antenatal care

[Zan ba abukiyata shawara ta je wannan wurin domin awo da renon cikinta]

◯ Agree ◯ Disagree ◯ Neither agree nor disagree

h. I felt respected

[Na ji an mutunta ni]

◯ Agree ◯ Disagree ◯ Neither agree nor disagree

i. I had enough time with my provider

[Na sami isassehen lokaci da mai renon cikina]

◯ Agree ◯ Disagree ◯ Neither agree nor disagree

j. I am very satisfied with the antenatal care I received

[Na gamsu sosai da renon ciki da na samu]

◯ Agree ◯ Disagree ◯ Neither agree nor disagree

COMPLICATIONS

5. Did you experience any problems during previous pregnancies or deliveries?

[Kin sami wata matsala lokacin ciki ko haifuwa a baya?]

- ◯ Yes [Eh]
- ◯ No [A'a]
- ◯ Not sure/Don't remember [Ban iya tunawa]

Specify
[A yi bayani]

____

PLACE OF DELIVERY

6. Where did you deliver your last baby?

[Ina kika yi haifuwarki ta baya?]

- ◯ At home [A gida]
- ◯ At a health facility [A asibiti]
- ◯ In transit to a facility [A kan hanyar zuwa asibiti]
- ◯ Other [In wani daban]

(Location)

Other, Specify

[In wani daban, a yi bayani]

____

ALL WOMEN: INTENDED PLACE OF DELIVERY AND FP BASELINE

7. Where do you intend to deliver this baby?

[Ina kike marmarin haifuwar wannan cikin?]

- ◯ At home [A gida]
- ◯ At a health facility [A asibiti]
- ◯ Not sure/Undecided [Ban sani ba tukuna]
- ◯ Other [In wani da ban]

Other, Specify

[In wani daban, a yi bayani]

____

8. Before this pregnancy, did you use any of the following methods to delay or prevent getting pregnant?

[Kafin wannan cikin ke ko mijinki waninku ya dauki matakin hana shi ko kuwa nisanta shi?]

- ☐ Lactational Amenorrhea Method (LAM) [Tsarin bayar da mamar uwa don hana ciki]
- ☐ Standard days or rhythm method (cycle beads/calendar/billing's method) [Kebabbun ranakku ko tsarin daki-daki bisa ga sanin lokacin al'adar wata-wata]
- ☐ Withdrawal [Cirewa kafin fitar maniyi]
- ☐ Condoms [Kwaroron roba]
- ☐ Pills/Oral contraceptives [Kwayoyin hadiya]
- ☐ Injectable [Allura]
- ☐ Implants [Wanda ake sawa karkashin fata]
- ☐ IUD [Na'urar sakawa a mahaifa]
- ☐ Emergency contraception [Matakin gaggawa na hana haifuwa]
- ☐ Other [In da wata hanya]
- ☐ None [Babu]

(Read one by one and mark all that apply)

Other, Specify

[In wani daban, a yi bayani]

____

9. Would you say that in the past, using contraception is mainly your decision, mainly your (husband's/partner's) decision, or did you both decide together

[Kina iya cewa a inda aka fito amfani da hanyar hana haifuwa shawararki ce ke kadai ko kuwa ta mijinki ce shi kadai ko ta dukanku ce?]

- ◯ Mainly respondent [Tawa ce ni kadai]
- ◯ Mainly husband/partner [Ta mijina ce shi kadai]
- ◯ Joint decision [Tare muka dauki niyar]
- ◯ Other [In da wata hanya]

Other, Specify

[In wani daban, a yi bayani]

____

10. If it was up to you, after you have this baby, would you want to delay getting pregnant again right away?

[Idan zai zama ra'ayinki za ki so ki yi tazarar daukar ciki bayan kin haifi wannan jariri?]

- ◯ Yes [Eh]
- ◯ No [A'a]
- ◯ Not sure/undecided [Ban sani ba tukuna]

11. Do you think there are advantages to spacing pregnancies apart?

[Kin yarda da cewa akwai amfani ga sa tazara tsakanin haifuwa da daukar wani ciki?]

- ◯ Yes [Eh]
- ◯ No [A'a]
- ◯ Not sure [Ban sani ba tukuna]

12. If yes, what are some advantages?

[Idan Eh ne, mine ne amfanin]

- ☐ Mom will be healthier/ less likely to have problems [Uwa za ta zama da lafiya/ da kuma rashin matsaloli]
- ☐ Baby will be healthier/ less likely to have problems [Wannan jariri da ma wadanda za su biyo baya za su sami lafiya/ba za a sami yawan matsaloli ba/ da wuya a sami matsalar haifuwa kafin lokaci]
- ☐ Easier to feed and/or take care of other children [Za a sami sau?in ciyarwa da lura da yara]
- ☐ Saves money [Za a iya tsimin ku?i]
- ☐ Other [In da wani]

(Do not prompt. Probe, any more? Select all items mentioned.)

Other, Specify

[In wani daban, a yi bayani]

____

ALL WOMEN: KNOWLEDGE BASELINE

13. How long should pregnancies be spaced apart for the best health of the mother and baby?

[Mine ne nisan tazarar da ya dace tsakanin ciki da ciki domin kyakkyawar lafiyar uwa da jariri?]

- ◯ Knows the time
- ◯ Don't know
- ◯ No response

Enter the time in months here

____
(Enter in Months (1 yr=12 months; 2 yrs = 24 months; 3 yrs = 36 months))

14. When do you think it is best to first breastfeed your baby?

[Yaushe kike tunanin ya dace ki fara ba jaririnki nono?]

- ◯ Immediately or within 1 hour of delivery [Nan take ko kada a wuce awa ?aya bayan haifuwa]
- ◯ After the first milk has gone (when "true" milk comes in) [Bayan nonon farko ya kau (insa'adda nonon kwarai ya samu)]
- ◯ Other [In da wata hanya]

([read response options?])

Other, Specify

[In wani daban, a yi bayani]

____

15. At what age do you think it is best to start giving your baby fluids or food other than breastmilk?

[A wane shekaru ne kike ganin ya kamata ki fara ba jaririnki kunu ko wani abinci daban da nonon uwa?]

- ◯ Knows the age
- ◯ Don't know
- ◯ No response

15.1. Weeks

[Mako]

____

15.2. Months

[Wata]

____

15.3. Other descriptors/comments

[in da wani a yi bayani]

____

16. Have you ever heard of using the lactational amenorrhea method, LAM, as a way to delay pregnancy after you have a baby? This method includes breastfeeding your baby.

[Kin taba jin ana amfani shayar da nonon uwa (LAM) a matsayin hanyar jinkirta ciki bayan haifuwa? Wannan ka'ida ta hada da shayar da jaririnki.]

- ◯ Yes [Eh]
- ◯ No [A'a]
- ◯ Not sure [Ban tuna na sani ba]

17. Can you describe how to use the lactational amenorrhea method (LAM)? What criteria or conditions need to be met for it to be an effective way to prevent or delay pregnancy?

[Kina iya bayana yadda ake amfani da shayar da nonon uwa a matsayin hanyar jinkirta daukar ciki? Wadanne ka'idodi ne ake bi don hana ko jinkirta daukar ciki
]

- ☐ Menstrual bleeding has not returned [Zubar jinin al'ada ba ta dawo ba tukuna]
- ☐ Baby is only breastfed/breastfed exclusively (no other food or liquids given) [Jariri yana shan nonon uwa kadai]
- ☐ Baby is less than six months old [Jariri bai kai wata shida ba tukuna]
- ☐ Other [In da wata hanya]
- ☐ Don't know [Ban sani ba tukuna]

Other, Specify

[In wani daban, a yi bayani]

____

18. After the birth of a child, can a woman become pregnant before her menstrual period has returned?

[Bayan haifuwar jariri mace tana iya daukar ciki kafin dawowar al'adar zubar jinin wata-wata?]

- ◯ Yes [Eh]
- ◯ No [A'a]
- ◯ Don't know [Ban sani ba]

19. In addition to the lactational amenorrhea method (LAM), what other forms of birth spacing or family planning can you use immediately after delivery?

[Ban da hanyar shayar da nonon uwa a matsayin jinkirta daukar ciki akwai wasu hanyoyi da za ki iya amfani da su?]

- ☐ Standard days or rhythm method (cycle beads/calendar/billing's method) [Kebabbun ranakku ko tsarin daki-daki]
- ☐ Withdrawal [Cire zakari kafin fitar maniyi]
- ☐ Condoms [Kwaroron roba]
- ☐ Pill/oral contraceptives [Kwayoyin hadiya]
- ☐ Injectable [Allura]
- ☐ Implants [Wadanda ake sawa karkashin fata]
- ☐ IUD [Na'urar sakawa a mahaifa]
- ☐ Emergency contraception [Matakin gaggawa na hana haifuwa]
- ☐ Sterilization [Daddaka]
- ☐ Other [In akwai wata hanya]
- ☐ None [Babu]
- ☐ Don't know [Ban sani ba tukuna]

(Check all that are mentioned. Prompt with any others. Do not read options.)

Other, Specify

[In wani daban, a yi bayani]

____

20. What are danger/warning signs during pregnancy? Things that mean there might be a problem with you or the baby?

[Wadanne ne alamun hadurra lokacin juna biyu? Abubuwa da ke nuna cewa ana iya samun matsaloli gare ki ko ga jaririnki?]

- ☐ Pain (not otherwise specified) [Ciwo]
- ☐ Headache [Ciwon kai]
- ☐ Vision changes/blurred vision [Sauyawar gani/ gani bushi-bushi]
- ☐ Nausea & vomiting [Tashin zuciya da amai]
- ☐ Pain in abdomen [Ciwon ciki]
- ☐ Shortness of breath [Wahalar numfashi]
- ☐ Fever [Masassara]
- ☐ Vaginal bleeding [Zubar jini]
- ☐ Leaking of fluids [Yoyon ruwa-ruwa]
- ☐ Change in colour or smell of vaginal discharge [Canja launi ko warin rowan]
- ☐ Painful urination [Jin zafi lokacin fitsari]
- ☐ Signs of Labor before it's time for the baby to come [Alamun nakuda kafin lokacin haifuwa (nakuda kafin lokacin haifuwa): zubar jini, tsamukar ciki]
- ☐ Reduced or No fetal movement [Ragewa ko daukewar motsin jariri]
- ☐ Other [Wata alama]
- ☐ Don't know [Ba a sani ba]

(Check all that are mentioned. Prompt with any others. Do not read options.)

Other, Specify

[In wani daban, a yi bayani]

____

21. What are danger/warning signs for you, the mother, after the baby is born? These are things that mean there might be a problem and that you should see a health care worker.

[Wadanne ne alamun hadurra gareki bayan kin haifi jariri? Wadannan abubuwa na nufin za yiwu a sami matsaloli kuma dole ki ga ma'aikacin lafiya]

- ☐ Heavy bleeding [Zubar jini da yawa]
- ☐ Severe abdominal (stomach) pain [Matsanancin ciwon ciki (tumbi)]
- ☐ Severe pain and/or read and hot breasts [Nono ya yi jawur yana zafi tare da matsanancin ciwo]
- ☐ Severe headache [Matsanancin ciwon kai]
- ☐ Visual disturbances [Rashin gani da kyau]
- ☐ Fever [Masassara]
- ☐ Unexplained pain in chest or legs [Dimewar ciwon kirji ko kafafu]
- ☐ Breathing difficulties [Matsalar Sheda]
- ☐ Difficult or painful urination [Jin zafi lokacin fitsari]
- ☐ Other [In da wani]
- ☐ Don't know [Ba a sani ba]

(Check all that are mentioned. Prompt with any others. Do not read options.)

Other, Specify

[In wani daban, a yi bayani]

____

22. What are danger/warning signs for your newborn? These are signs that there may be a problem and you need to seek care from a health care worker.

[Wadanne ne alamun hadurra ga sabon jaririnki? Wadannan abubuwa na nufin zai yiwu a sami matsaloli kuma dole ki ga ma'aikacin lafiya]

- ☐ Too hot or too cold [Jikinsa ya yi zafi sosai ko ya yi sanyi kwarai]
- ☐ Convulsions [Ya rinka kwankwangewa]
- ☐ Little or no movement [Ya zama ba ya motsawa ko kuma kadan yake motsi]
- ☐ Feeding poorly or not at all [Shan nono kadan-kadan ko rashin shan nono gaba daya]
- ☐ Fast breathing or chest indrawing [Sheda da sauri ko fadawar kirji]
- ☐ Not passing urine and/or stool [Rashin yin fitsari ko kashi]
- ☐ Umbilical cord stump is bleeding; has foul odor; or redness around it [Cibiya tana jini ko tana wari ko kuwa ta yi ja]
- ☐ Very small baby born at home [Dan mitsitsin jariri kuma an haife a gida]
- ☐ Yellow palms or soles of feet [Tafin hannuwa ko kafafu su yi kore-kore]
- ☐ Other [In da wani]
- ☐ Don't know [Ba a sani ba]

(Check all that are mentioned. Prompt with any others. Do not read options.)

Other, Specify

[In wani daban, a yi bayani]

____

23. What are the most important things you can do to help keep your newborn healthy?

[Wadanne hanyoyi ne za ki bi don kare lafiyar jaririnki?]

- ☐ Keep warm (keep head and body covered; skin to skin with mom) [A kare shi daga sanyi (a rufe kansa da jikinsa; uwa ta rungume shi)]
- ☐ Feed only on breastmilk [A ba shi nonon uwa kadai]
- ☐ Feed often/whenever baby wants [A ba shi nono a kai-a kai/ duk lokacin da yake so]
- ☐ Do not put anything on cord stump [Kada a sa kome a kan cibiyarsa]
- ☐ Keep cord stump clean [A tsabtace cibiyarsa koyaushe]
- ☐ Wash hands [A wanke hannuwa koyaushe]
- ☐ Have baby immunized [A yi wa jariri riga-kafi]
- ☐ Wait atleast 24 hours before bathing baby [A jira har awa 24 tukuna kafin a yi wa jariri wanka bayan haifuwa]
- ☐ Other [In da wani]
- ☐ Don't know [Ba a sani ba]

(Check all that are mentioned. Prompt with any others. Do not read options.)

Other, Specify

[In wani daban, a yi bayani]

____

24. Could you ask your husband/partner to use a condom if you wanted him to?

[Idan kina son mijinki ya yi amfani da kororon roba kina iya ce masa ya yi amfani da shi]

- ◯ Yes [Eh]
- ◯ No [A'a]
- ◯ Not sure/Depends [Ban sani ba / ya danganta]

25. Who will usually make decisions about health care for yourself and your newborn?

[Wa ke yanke hukunci game da kiwon lafiyarki da kiwon lafiyar jaririnki ?]

◯ You [Ke]
◯ Your husband/partner [Mijinki]
◯ You and your husband/partner jointly [Ke da mijinki gaba daya]
◯ Someone else, specify [Idan wani ne a yi bayani]

Someone else Specify

[Idan wani ne a yi bayani]

____

26. Thinking about this pregnancy, do you agree, disagree or neither agree nor disagree with the following statements?

[Bisa ga yanayin wannan ciki za ki yarda da wadannan ra'ayoyi?]

a. If I don't understand something a provider is telling me I will tell them and ask them to explain a different way

[Idan ban gane abinda ma'aikacin asibiti ke cewa ba zan fada masa kuma in nemi ya yi mani bayani yadda zan gane]

◯ Agree ◯ Disagree ◯ Neither agree nor disagree

b. There are things I can do to help prevent problems and keep myself and my baby healthy

[Akwai abubuwan da zan yi don taimakawa ga riga-kafin matsaloli in kare lafiyata da ta jaririna]

◯ Agree ◯ Disagree ◯ Neither agree nor disagree

c. I will talk with my husband/family about how to keep myself and our baby healthy

[Zan yi magana da mijina da iyalina game da yadda zan tsare lafiyata da ta jaririnmu]

◯ Agree ◯ Disagree ◯ Neither agree nor disagree

d. I know how to recognize a problem with my pregnancy

[Ina iya ganewa idan akwai matsala ga cikina]

◯ Agree ◯ Disagree ◯ Neither agree nor disagree

e. I know what actions I will take if I think there is a problem with my pregnancy

[Na san matakan da zan dauka idan na fahimci cewa akwai matsala ga cikina]

◯ Agree ◯ Disagree ◯ Neither agree nor disagree

f. I know how to recognize a problem with my newborn

[Na san yadda zan gane idan akwai matsala ga jaririna]

◯ Agree ◯ Disagree ◯ Neither agree nor disagree

g. I know what actions I will take if I think there is a problem with my newborn

[Na san matakan da zan dauka idan na fahimci cewa akwai matsala ga jaririna]

◯ Agree ◯ Disagree ◯ Neither agree nor disagree

h. I am good at making decisions related to the health of myself and my family

[Na iya daukar matakai game da lafiyata da lafiyar iyalina]

◯ Agree ◯ Disagree ◯ Neither agree nor disagree

27. Thinking about life generally, do you agree or disagree with the following statements?

[Bisa ga rayuwa da zamantakewa za ki amince da wadannan ra'ayoyi?]

a. I can talk to others about difficult subjects

[Ina iya yin magana da mutane a kan matsaloli masu sarkakiya]

◯ Agree ◯ Disagree

b. I can let others know what I really think, even if it is different from them

[Ina iya fada ma mutane ra'ayina ko da ya saba nasu]

◯ Agree ◯ Disagree

c.I like to make decisions for myself

[Ina so in dauki mataki da kaina game da abinda ya shafe ni]

◯ Agree ◯ Disagree

d. I am able to make decisions for myself

[Na isa in dauki mataki don kaina]

◯ Agree ◯ Disagree

Any other Comments

[Akwai wani bayani ko ra'ayi] ______________________________

THANK THE PARTICIPANT AND END THE INTERVIEW
