# Supplementary material for: Group versus individual antenatal and first year postpartum care: Study protocol for a multi-country cluster randomized controlled trial in Kenya and Nigeria
Source: Gates Open Res. 2019 Jul 4;2:56. Originally published 2018 Nov 5. [Version 2] doi: 10.12688/gatesopenres.12867.2 (PMC6350506; doi:10.12688/gatesopenres.12867.2)
Supplement: Supplementary file 1 [file gatesopenres-2-14160-s0000.tgz › 72242879-e244-48c8-a71c-b86050fb7704_Supplemental_files-_Study_manual,_Data_tools,_and_recruitment_,materials.zip!/Supplemental files- Study manual, Data tools, and recruitment ,materials/Demographic SES Survey - Kenya.pdf]

Demographic SES Survey

PARTICIPANT STUDY ID ______________________________

1. What is the main source of drinking water for members of your family
Chanzo kikuu cha maji ya kunywa kwa wananyumba wako ni kipi

- ◯ Piped water - Maji ya mfareji
- ◯ Dug well - Kisima kilichochimbwa
- ◯ Water from spring - Maji kutoka mtoni
- ◯ Rainwater - Maji ya mvua
- ◯ Tanker truck - Lori ya maji
- ◯ Cart with small tank - Mkokoteni ulio na tenki ndogo
- ◯ Surface water (river/dam/lake/pond/stream/canal) - Maji ya chini (Mto/Bwawa/ Ziwa/Dimbwi/Mfereji/Unyunyuzi)
- ◯ Irrigation channel - Njia ya unyunyuzi
- ◯ Bottled water - Maji ya chupa
- ◯ Sachet water - Maji ya sacheti
- ◯ Other - Nyingine

Other specify ______________________________

2. Where is that water source located
Chanzo hicho cha maji kiko wapi?

- ◯ In own dwelling - Kwenye makazi yake binafsi
- ◯ In own yard/plot - Kwenye ploti yake kibinafsi
- ◯ Elsewhere - Kwingine

3. What kind of toilet facility do members of your household usually use
Kwa kawaida, wanafamilia wako hutumia choo cha aina gani?

- ◯ Flush or pour toilet - Choo cha kubumburusha/kumwaga ili kubumburusha
- ◯ Pit latrine - Choo cha shimo
- ◯ Other - Nyingine

Other specify ______________________________

4. Do you share this toilet facility with other households
Mnatumia choo hiki na wananyumba wa nyumba ingine?

- ◯ Yes - Ndio
- ◯ No - La

5. Does your household have
Nyumba yako ina

	Yes	No
5a. Electricity<br>Umeme	◯	◯
5b. A radio	◯	◯
5c. A television<br>Televisheni	◯	◯
5d. A mobile telephone<br>Simu ya rununu	◯	◯
5e. A refrigerator<br>Friji	◯	◯
5f. A cable TV<br>Televisheni ya kebo	◯	◯

5g. A generating set	◯	◯
5h. Air conditioner Kiyoyozi	◯	◯
5i. A computer Tarakilishi	◯	◯
5j. Electric iron Pasi ya umeme	◯	◯
5k. A fan Feni	◯	◯

6. What type of fuel does your household mainly use for cooking
Wananyumba wako wanatumia aina ipi ya kuni kupika?

- ◯ Electricity - Stima
- ◯ LPG - Gesi ipatikanayo kwenye mtungi.
- ◯ Natural gas - Gesi ya asilia
- ◯ Biogas - Gesi itokayo kwa kinyesi
- ◯ Kerosene - Mafuta taa
- ◯ Coal, lignite - Makaa ya mawe
- ◯ Charcoal - Makaa
- ◯ Wood - Kuni
- ◯ Straw/shrubs/grass - Nyasi/vijiti
- ◯ Agricultural crop - Mimea ya kilimo
- ◯ Animal dung - Samadi ya mnyama

7. Main material of the floor
Maghafi kuu ya sakafu

- ◯ Natural floor - Sakafu asilia
- ◯ Rudimentary floor - Sakafu ya kawaida
- ◯ Finished floor - Sakafu iliyokamilika

7a. Natural floor
Sakafu asilia

- ◯ Earth/sand - Udongo/mchanga
- ◯ Dung - Samadi

7b. Rudimentary floor
Sakafu ya kawaida

- ◯ Wood planks - Mbao
- ◯ Palm/bamboo -

7c. Finished floor
Sakafu iliyokamilika

- ◯ Partquet or polished wood - Mbao
- ◯ Vinyl or asphalt strips - Vipande vya vinylau asphalt
- ◯ Ceramic tiles - Vigae vilivyofinywanga
- ◯ Cement - Simiti
- ◯ Carpet/rug - Zulia
- ◯ Other - Nyingine

Other specify

____

8. How many rooms in total are in your household including rooms for sleeping and all other rooms
Nyumba yako ina jumla ya vyumba vingapi ikiwemo vyumba ambavyo vinatumika kama vya kulala

____

9. How many rooms are used for sleeping in your household
Ni vyumba vingapi kwenye nyumba yako ambavyo vinatumika kama vyumba vya kulala?

____

10. Does any member of this household own
Kuna mtu wa nyumba hii ambaye anamiliki

	Yes	No
10a. A watch<br>Saa	○	○
10b. A bicycle<br>Baiskeli	○	○
10c. A motorcycle or motor scooter<br>Pikipiki	○	○
10d. An animal-drawn cart<br>Mkokoteni unaovutwa na mnyama	○	○
10e. A car or truck<br>Gari au lori	○	○
10f. A boat with a motor<br>Mashua iliyo na mota	○	○
10g. A canoe<br>Mtumbwi	○	○

That is the end of the survey. Do you have any other comments
Huu ndio mwisho wa Aua. Una maoni mengine? ______________________________
