# Supplementary material for: Group versus individual antenatal and first year postpartum care: Study protocol for a multi-country cluster randomized controlled trial in Kenya and Nigeria
Source: Gates Open Res. 2019 Jul 4;2:56. Originally published 2018 Nov 5. [Version 2] doi: 10.12688/gatesopenres.12867.2 (PMC6350506; doi:10.12688/gatesopenres.12867.2)
Supplement: Supplementary file 1 [file gatesopenres-2-14160-s0000.tgz › 72242879-e244-48c8-a71c-b86050fb7704_Supplemental_files-_Study_manual,_Data_tools,_and_recruitment_,materials.zip!/Supplemental files- Study manual, Data tools, and recruitment ,materials/Demographic SES Survey - Nigeria.pdf]

Demographic SES Survey

PARTICIPANT STUDY ID ________________________________

1. What is the main source of drinking water for members of your family
Wacce hanyar samun ruwa kuke da ita ga iyalan ku?

- ◯ Piped water - Ruwan fanfo
- ◯ Dug well - Ruwan rijiya
- ◯ Water from spring - Ruwan korama
- ◯ Rainwater - Ruwan sama
- ◯ Tanker truck - Ruwan daga motar ruwa
- ◯ Cart with small tank - Ruwan wilbaro
- ◯ Surface water (river/dam/lake/pond/stream/canal) - Kwanceccan ruwa (Rafi/Ruwan Dam/ Kogi/Kudiddifi/Korama)
- ◯ Irrigation channel - Ruwan noman rani
- ◯ Bottled water - Ruwan roba
- ◯ Sachet water - Ruwan leda
- ◯ Other - Wani

Other specify ________________________________

2. Where is that water source located
Daga ina ake samo ruwan?

- ◯ In own dwelling - A nan unguwar
- ◯ In own yard/plot - A cikin Gidana/Filina
- ◯ Elsewhere - A wani wuri daban

3. What kind of toilet facility do members of your household usually use
Wane irin bayan gida ko ban daki iyalinki suke amfani dashi?

- ◯ Flush or pour toilet - Wurin Yin Bayan gida na tangaran ko zamani
- ◯ Pit latrine - Ramin Shadda
- ◯ Other - Wani

Other specify ________________________________

4. Do you share this toilet facility with other households
Kuna amfani da shi tare da mutan nan gidan?

- ◯ Yes - Eh
- ◯ No - Aa

5. Does your household have
Gidan ku akwai

	Yes	No
5a. Electricity<br>Wutar lantarki?	◯	◯
5b. A radio<br>Akwai radio?	◯	◯
5c. A television<br>Akwai talabijin?	◯	◯
5d. A mobile telephone<br>Akwai wayar hannu?	◯	◯
5e. A refrigerator<br>Akwai firij ?	◯	◯
5f. A cable TV	◯	◯
5g. A generating set<br>Jenareta	◯	◯

5h. Air conditioner<br>Na'urar sanyaya 'daki?	○	○
5i. A computer<br>Na'ura mai 'kwa'kwalwa	○	○
5j. Electric iron<br>Akwai dutsen guga	○	○
5k. A fan<br>Akwai fanka	○	○

---

6. What type of fuel does your household mainly use for cooking
Wane irin mai kuka fi amfani da shi a wajen girki ?

○ Electricity - Lantarki
○ LPG - Iskar Gas
○ Natural gas - Gas na mai
○ Biogas
○ Kerosene - Kanazir
○ Coal, lignite
○ Charcoal - Gawayi
○ Wood - Itace
○ Straw/shrubs/grass - Yayi/Karmami/Ciyawa.
○ Agricultural crop - Itacen Gona
○ Animal dung - Kashin dabbobi

---

7. Main material of the floor
Abinda aka yi daben kasa da shi.

○ Natural floor - Kasa yadda take
○ Rudimentary floor - Saukakyakykyan dabe
○ Finished floor - Gyararran Dabe

7a. Natural floor

○ Earth/sand - Kasa/Rairayi
○ Dung - Yayi

7b. Rudimentary floor

○ Wood planks - Falankin Katako
○ Palm/bamboo - Icen Bishiyar Dabino/Ciyawar Bunu

7c. Finished floor

○ Partquet or polished wood - Daben Kyakykyawan Katako
○ Vinyl or asphalt strips
○ Ceramic tiles - Shinfidar tayil
○ Cement - Siminti
○ Carpet/rug - Kafet/Karamin Kafet
○ Other - Akwai wani

Other specify

____

---

8. How many rooms in total are in your household including rooms for sleeping and all other rooms
Daki nawa kuke amfani da su a gidan nan harda da dakunnan bacci da sauran dakuna?

____

---

9. How many rooms are used for sleeping in your household
Daki nawane na kwana a gidan nan?

____

---

10. Does any member of this household own
A cikin mutan gidan nan akwai wanda ya mallaki

	Yes	No
10a. A watch/clock<br>Agogo	◯	◯
10b. A bicycle<br>Keke	◯	◯
10c. A motorcycle or motor scooter<br>Babir	◯	◯
10d. An animal-drawn cart<br>Amalanke ko keken shanu	◯	◯
10e. A car or truck<br>Mota	◯	◯
10f. A boat with a motor<br>Kwalekwale mai inji	◯	◯
10g. A canoe<br>Jirgin Ruwa na katako	◯	◯

That is the end of the survey. Do you have any other comments
Wanna shene karshen tamboyin . Kina da wani karin bayani? ______________________________
