# Supplementary material for: Group versus individual antenatal and first year postpartum care: Study protocol for a multi-country cluster randomized controlled trial in Kenya and Nigeria
Source: Gates Open Res. 2019 Jul 4;2:56. Originally published 2018 Nov 5. [Version 2] doi: 10.12688/gatesopenres.12867.2 (PMC6350506; doi:10.12688/gatesopenres.12867.2)
Supplement: Supplementary file 1 [file gatesopenres-2-14160-s0000.tgz › 72242879-e244-48c8-a71c-b86050fb7704_Supplemental_files-_Study_manual,_Data_tools,_and_recruitment_,materials.zip!/Supplemental files- Study manual, Data tools, and recruitment ,materials/Phase I Data Extraction_G-ANC register.pdf]

Data Extraction

PARTICIPANT STUDY ID ________________

Date and Time of data collection - Data Quality Check ________________

Date of data collection/ interview ________________
(DD-MM-YYYY)

First and Last Name of data collector ________________

How many attempts were made by phone to reach this participant ________________
(ENTER A NUMBER e.g. 1)

Were additional strategies used to trace this participant?
- ◯ Yes
- ◯ No

Describe the additional strategies ________________

Is this person lost to follow up?
- ◯ Yes
- ◯ No

FOR INTERVENTION SITES ONLY - EXTRACT DATA FROM GROUP ANC REGISTER

1. Did woman attend any group meetings?
   - ◯ Yes
   - ◯ No

2. HIV status known
   - ◯ Yes
   - ◯ No

3. Partner HIV tested
   - ◯ Yes
   - ◯ No

4. Which group meetings did the woman attend?
   - ☐ M1
   - ☐ M2
   - ☐ M3
   - ☐ M4
   - ☐ M5

   (Select all that apply)

5. Woman has LLIN?
   - ◯ Yes
   - ◯ No

6. Given IFAS? (ever)
   - ◯ Yes
   - ◯ No

7. For meeting 1, select all that apply
   - ☐ Has IFA
   - ☐ Took IFA
   - ☐ Bed net
   - ☐ Gave SP
   - ☐ N/A, missed meeting

8. For meeting 2, select all that apply
   - ☐ Has IFA
   - ☐ Took IFA
   - ☐ Bed net
   - ☐ Gave SP
   - ☐ N/A, missed meeting

9. For meeting 3, select all that apply

- ☐ Has IFA
- ☐ Took IFA
- ☐ Bed net
- ☐ Gave SP
- ☐ N/A, missed meeting

10. For meeting 4, select all that apply

- ☐ Has IFA
- ☐ Took IFA
- ☐ Bed net
- ☐ Gave SP
- ☐ N/A, missed meeting

11. For meeting 5, select all that apply

- ☐ Has IFA
- ☐ Took IFA
- ☐ Bed net
- ☐ Gave SP
- ☐ N/A, missed meeting

12. Meeting 1 blood pressure

12. Meeting 1: Systolic ________________________________

12. Meeting 1: Diastolic ________________________________

13. Meeting 2 blood pressure

13. Meeting 2: Systolic ________________________________

13. Meeting 2: Diastolic ________________________________

14. Meeting 3 blood pressure

14. Meeting 3: Systolic ________________________________

14. Meeting 3: Diastolic ________________________________

15. Meeting 4 blood pressure

15. Meeting 4: Systolic ________________________________

15. Meeting 4: Diastolic ________________________________

16. Meeting 5 blood pressure

16. Meeting 5: Systolic ________________________________

16. Meeting 5: Diastolic ________________________________

17. Other danger sign identified from self-assessment card?

____

18. BP counseling

- ◯ Yes
- ◯ No

19. Place of delivery specified?

- ◯ Yes
- ◯ No

20. Place of delivery ________________________________

21. Plan: transport

- ◯ Yes
- ◯ No
- ◯ N/A

22. Plan: emergency cash
- ◯ Yes
- ◯ No
- ◯ N/A

23. Plan: companion
- ◯ Yes
- ◯ No
- ◯ N/A

24. Plan: decision maker
- ◯ Yes
- ◯ No
- ◯ N/A

25. FP desired? Meeting 4
- ◯ Yes
- ◯ No
- ◯ N/A

26. FP desired? Meeting 5
- ◯ Yes
- ◯ No
- ◯ N/A

27. Chosen desired method? Meeting 4 (G4)
- ◯ Yes
- ◯ No

27i. Specify method
- ◯ Injectable
- ◯ Implants
- ◯ Withdrawal
- ◯ Condoms
- ◯ Pill/Oral contraceptives
- ◯ IUD
- ◯ Emergency contraception
- ◯ Sterilisation
- ◯ Standard days or rhythm method
- ◯ LAM
- ◯ Other

Other G4 method ________________________________

28. Chosen desired method? Meeting 5 (G5)
- ◯ Yes
- ◯ No

28i. Specify method
- ◯ Injectable
- ◯ Implants
- ◯ Withdrawal
- ◯ Condoms
- ◯ Pill/Oral contraceptives
- ◯ IUD
- ◯ Emergency contraception
- ◯ Sterilisation
- ◯ Standard days or rhythm method
- ◯ LAM
- ◯ Other

Other G5 method ________________________________

29. If LAM select method, transition method chosen?
- ◯ Yes
- ◯ No
- ◯ N/A

29i. Specify method

- ◯ Injectable
- ◯ Implants
- ◯ Withdrawal
- ◯ Condoms
- ◯ Pill/Oral contraceptives
- ◯ IUD
- ◯ Emergency contraception
- ◯ Sterilisation
- ◯ Standard days or rhythm method
- ◯ Other

Other transition method ________________________________
