# Supplementary material for: Group versus individual antenatal and first year postpartum care: Study protocol for a multi-country cluster randomized controlled trial in Kenya and Nigeria
Source: Gates Open Res. 2019 Jul 4;2:56. Originally published 2018 Nov 5. [Version 2] doi: 10.12688/gatesopenres.12867.2 (PMC6350506; doi:10.12688/gatesopenres.12867.2)
Supplement: Supplementary file 1 [file gatesopenres-2-14160-s0000.tgz › 72242879-e244-48c8-a71c-b86050fb7704_Supplemental_files-_Study_manual,_Data_tools,_and_recruitment_,materials.zip!/Supplemental files- Study manual, Data tools, and recruitment ,materials/Phase I DataExtraction_all women.pdf]

Section 2 And 3 Data

PARTICIPANT STUDY ID ______________________________

SECTION 2: ALL WOMEN

1. When you enrolled in the study you were receiving ANC care at [state facility]. Have you gone to any other facilities for ANC care since then?
   - ◯ Yes
   - ◯ No

2. How many ANC visits did you go to in total at any facility? ______________________________ (ENTER A NUMBER e.g. 3 NOT three)

3. Did you take the same mother and child health booklet (Kenya)/ ANC card (Nigeria) to all your ANC visits?
   - ◯ Yes
   - ◯ No
   - ◯ Unsure

4. Is your mother and child health booklet (Kenya) / ANC card (Nigeria) available?
   - ◯ Yes
   - ◯ No

5. May I please see your mother and child health booklet (Kenya) / ANC card (Nigeria) to extract some information for the study? I am looking to see what services were provided and will not record the results on any tests.
   - ◯ Permission granted
   - ◯ Permission denied

6. After ANC 1, did you attend any individual ANC visits, any that were not part of the group?
   - ◯ Yes
   - ◯ No

SECTION 3: DATA EXTRACTION FROM MCB/ANC CARD OR ANC REGISTER

For INTERVENTION SITES, only add information for ANC1 plus any other visits that were made in addition to group visits - DON'T ADD GROUP VISITS HERE.

Date of ANC visit ______________________________

Blood pressure

Systolic ______________________________

Diastolic ______________________________

Given IFA
- ◯ Yes
- ◯ No
- ◯ N/A

Given IpTP
- ◯ Yes
- ◯ No
- ◯ N/A

Is there another visit to record?
- ◯ Yes
- ◯ No

Date of ANC visit ______________________________

Blood pressure

Systolic ______________________________

Diastolic	________________
Given IFA	◯ Yes<br>◯ No<br>◯ N/A
Given IpTP	◯ Yes<br>◯ No<br>◯ N/A
Is there another visit to record?	◯ Yes<br>◯ No
Date of ANC visit	________________
Blood pressure	
Systolic	________________
Diastolic	________________
Given IFA	◯ Yes<br>◯ No<br>◯ N/A
Given IpTP	◯ Yes<br>◯ No<br>◯ N/A
Is there another visit to record?	◯ Yes<br>◯ No
Date of ANC visit	________________
Blood pressure	
Systolic	________________
Diastolic	________________
Given IFA	◯ Yes<br>◯ No<br>◯ N/A
Given IpTP	◯ Yes<br>◯ No<br>◯ N/A
Is there another visit to record?	◯ Yes<br>◯ No
Date of ANC visit	________________
Blood pressure	
Systolic	________________
Diastolic	________________
Given IFA	◯ Yes<br>◯ No<br>◯ N/A

Given IpTP
◯ Yes
◯ No
◯ N/A

Is there another visit to record?
◯ Yes
◯ No

Date of ANC visit ________________________________

Blood pressure

Systolic ________________________________

Diastolic ________________________________

Given IFA
◯ Yes
◯ No
◯ N/A

Given IpTP
◯ Yes
◯ No
◯ N/A

Is there another visit to record?
◯ Yes
◯ No

Date of ANC visit ________________________________

Blood pressure

Systolic ________________________________

Diastolic ________________________________

Given IFA
◯ Yes
◯ No
◯ N/A

Given IpTP
◯ Yes
◯ No
◯ N/A

Is there another visit to record?
◯ Yes
◯ No

Date of ANC visit ________________________________

Blood pressure

Systolic ________________________________

Diastolic ________________________________

Given IFA
◯ Yes
◯ No
◯ N/A

Given IpTP
◯ Yes
◯ No
◯ N/A

Total number of ANC visits ________________________________

Was client given LLIN during ANC care
- ◯ Yes
- ◯ No
- ◯ N/A

HIV status known?
- ◯ Yes
- ◯ No
- ◯ N/A

HIV partner testing completed and status known?
- ◯ Yes
- ◯ No
- ◯ N/A

Syphilis testing completed (VDRL/RPR)
- ◯ Yes
- ◯ No
- ◯ N/A

DATA EXTRACTION: DIAGNOSIS MADE

Were any of the following conditions detected during examination? [in mother child booklet look at "clinical notes" on pg. 6 & 10]
- ☐ Hypertension
- ☐ Diabetes
- ☐ Malaria
- ☐ STIs/RTIs
- ☐ Other
- ☐ None

Other conditions detected ______________________________

Were any of the following conditions treated during the visit? [in mother child booklet look at "clinical notes" on pg. 6 & 10]
- ☐ Hypertension
- ☐ Diabetes
- ☐ Malaria
- ☐ STIs/RTIs
- ☐ Other
- ☐ None

Other condition treated ______________________________

Please select all data sources consulted for this record
- ☐ ANC group register
- ☐ Kenya mother child book
- ☐ Nigerian ANC card
- ☐ ANC registry

(select all that apply)
