# Supplementary material for: Group versus individual antenatal and first year postpartum care: Study protocol for a multi-country cluster randomized controlled trial in Kenya and Nigeria
Source: Gates Open Res. 2019 Jul 4;2:56. Originally published 2018 Nov 5. [Version 2] doi: 10.12688/gatesopenres.12867.2 (PMC6350506; doi:10.12688/gatesopenres.12867.2)
Supplement: Supplementary file 1 [file gatesopenres-2-14160-s0000.tgz › 72242879-e244-48c8-a71c-b86050fb7704_Supplemental_files-_Study_manual,_Data_tools,_and_recruitment_,materials.zip!/Supplemental files- Study manual, Data tools, and recruitment ,materials/Phase I RDW survey_Women.pdf]

Anc Survey Recently Delivered Women

PARTICIPANT STUDY ID ________________

1. Tell me what happened to your last pregnancy

[Yadda haifuwa ta zo]

- ◯ Live birth [An haifi yaro lafiya]
- ◯ Still birth [Yaro ya zo ba rai]
- ◯ Miscarriage [An barin ciki]

Live birth, date baby born

[An haifi yaro lafiya, ranar]

________________ (DD-MM-YYYY)

Still birth, date baby born

[Yaro ya zo ba rai, ranar]

________________ (DD-MM-YYYY)

Miscarriage, Week of pregnancy

[An barin ciki - Makonnin ciki (GA)]

________________ (Enter a number)

Miscarriage, Date

[An barin ciki - ranar]

________________ (DD-MM-YYYY)

2. Did you know your due date, the time around when the baby was suppose to be born?

[Kin san ranar da za ki haifu, watau lokacin da ake sa ran za a haifi yaronki?]

- ◯ Yes [Eh]
- ◯ No [A'a]
- ◯ Not sure [Ban tabbata ba]

3. If yes, how?

[Idan Eh, yaya kika sani ?]

- ◯ Provider told me [Mai awo da renon ciki ya fada mani]
- ◯ Other [In da wata hanya a fada]

Other

[In da wata hanya a fada]

____

4. Do you think your baby was premature, that is born too soon?

[Kina tsammani jaririnki bai isa haifuwa ba ?]

- ◯ Yes [Eh]
- ◯ No [A'a]
- ◯ Not sure [Ban tabbata ba]

5. Is your baby alive today?

- ◯ Yes
- ◯ No

5a. I'm sorry for your loss. Is it OK if I ask you questions about the antenatal care you received? If it will be too upsetting we can stop now.

- ◯ Yes
- ◯ No

Improved quality of Antenatal care

6. What are danger/warning signs during pregnancy? Things that mean there might be a problem with you or the baby?

[Wadanne ne alamun hadurra a lokacin juna biyu? Abubuwa da ke nuna akwai matsala gare ki ko ga jariri?]

- ☐ Pain (not otherwise specified) [Ciwo]
- ☐ Headache [Ciwon kai]
- ☐ Vision changes/blurred vision [Canjin gani ko gani buji-buji]
- ☐ Nausea and vomiting [Tashin zuciya & amai]
- ☐ Pain in abdomen[Ciwon ciki]
- ☐ Shortness of breath [Rashin numfashi da kyau]
- ☐ Fever [Masassara]
- ☐ Vaginal bleeding [Zubar jini]
- ☐ Leaking of fluids [Yoyon ruwa-ruwa]
- ☐ Change in color or smell of vaginal discharge [Sauya launi da wari ga yoyon]
- ☐ Painful urination [Jin ciwo lokacin fitsari]
- ☐ Signs of labour before its time for the baby to come (preterm labour): bloody show, cramping/painful tightening of belly/contractions [Alamun nakuda kafin lokacin haifuwa (nakuda kafin lokacin haifuwa): zubar jini, tsamukar ciki (sa alama ga wanda aka ambata)]
- ☐ Reduced or no fetal movement [Ragewa ko daukewar motsin jariri (cikin ciki)]
- ☐ Other [In da wani a yi bayanir]
- ☐ Don't know [Ba a sani ba]

(Check all that are mentioned. Prompt with "any others". Do not read options. DON'T KNOW should NOT be checked if other options are selected.)

Other

[In da wani a yi bayanir]

____

7. At every antenatal visit, did your provider ask if you had:

[Mai renon cikinki yakan tambaye ki a duk lokacin awo ko kina jin]

a. Pain

[Ciwo]

- ◯ Yes [Eh]
- ◯ No [A'a]
- ◯ Not sure [Ban tuna ba]

b. Fever

[Masassara]

- ◯ Yes [Eh]
- ◯ No [A'a]
- ◯ Not sure [Ban tuna ba]

c. Bleeding

[Zubar jini]

- ◯ Yes [Eh]
- ◯ No [A'a]
- ◯ Not sure [Ban tuna ba]

d. leakage of fluids

[Yoyon ruwa-rwa]

- ◯ Yes [Eh]
- ◯ No [A'a]
- ◯ Not sure [Ban tuna ba]

e. Reduced or no fetal movement

[Regewa ko daukewar motsin jariri cikin ciki]

- ◯ Yes [Eh]
- ◯ No [A'a]
- ◯ Not sure [Ban tuna ba]

8. Have you ever been told you had high blood pressure?

[An taba fada maki cewa kina da hawan jini?]

- ◯ Yes [Eh]
- ◯ No [A'a]
- ◯ Not sure [Ban tuna ba]

9. Did you have high blood pressure before pregnancy?

[Kin taba samun hawan jini kafin wannan ciki?]

- ◯ Yes [Eh]
- ◯ No [A'a]
- ◯ Not sure

10. Did you attend additional ANC visits because of it?

[Kin taba zuwa awon ciki saboda hawan jininki?]

- ◯ Yes [Eh]
- ◯ No [A'a]
- ◯ Not sure [Ban tuna ba]

11. Did you take any medication for it?

[Kin taba shan wani magani saboda saboda hawan jininki lokacin da kina da ciki?]

- ◯ Yes [Eh]
- ◯ No [A'a]
- ◯ Not sure [Ban tuna ba]

12. Did you receive any other special care related to your blood pressure?

[Kin taba samun wani taimako na musamman saboda hawan jininki?]

- ◯ Yes [Eh]
- ◯ No [A'a]

Yes, Specify

[Idan Eh a yi bayani]

____

For questions 13 - 27 , mark the first response mentioned. If sought help in the community, or respondent gives a person's name, probe to discern appropriate category

13. During your pregnancy did you ever have a severe headache or vision changes?

[Lokacin juna biyunki kin taba samun matsanancin ciwon kai ko mtsalar gani?]

- ◯ Yes [Eh]
- ◯ No [A'a]

14. Did you seek help?

[Kin nemi taimako?]

- ◯ Yes [Eh]
- ◯ No [A'a]

15. Where/from who did you first seek help?

[Aina /ko a wurin wa kika fara neman taimakon?]

- ◯ Dispensary/health center/clinic [N-primary] [Dakin jinya /karamin asibit]
- ◯ Hospital [N-secondary/tertiary] [Asibiti]
- ◯ Chemist/Pharmacy [Dakin sayar da magani]
- ◯ Community health worker/volunteer [Ma'aikacin lafiya na sa-kai]
- ◯ Non CHW TBA [if TBA & CHW, categorize as CHW]
- ◯ Friend/family/neighbor [Aboki/abukiya/iyali/makwabci/makwabciya]
- ◯ Traditional healer/faith healer [Mai maganin gargajiya /mai addu'a]
- ◯ Other [In da wata hanya a yi bayani]

Other

[In da wata hanya a yi bayani]

____

16. During your pregnancy did you ever have any vaginal bleeding or leakage of fluids?

[Lokacin cikinki kin taba samun zubar jini ko yoyon ruwa-ruwa?]

- ◯ Yes [Eh]
- ◯ No [A'a]

17. Did you seek help?

[Kin nemi taimako?]

- ◯ Yes [Eh]
- ◯ No [A'a]

18. Where/from who did you first seek help?

[Aina /ko a wurin wa kika fara neman taimakon?]

- ◯ Dispensary/health center/clinic [N-primary] [Dakin jinya /karamin asibit]
- ◯ Hospital [N-secondary/tertiary] [Asibiti]
- ◯ Chemist/Pharmacy [Dakin sayar da magani]
- ◯ Community health worker/volunteer [Ma'aikacin lafiya na sa-kai]
- ◯ Non CHW TBA [if TBA & CHW, categorize as CHW]
- ◯ Friend/family/neighbor [Aboki/abukiya/iyali/makwabci/makwabciya]
- ◯ Traditional healer/faith healer [Mai maganin gargajiya /mai addu'a]
- ◯ Other [In da wata hanya a yi bayani]

Other

[In da wata hanya a yi bayani]

____

19. During your pregnancy did you ever have painful contractions or cramping when it was too early for the baby to come?

[Loakacin cikinki kin taba samun tsamuka ko mirdewar ciki lokacin da jariri bai isa haifuwa?]

- ◯ Yes [Eh]
- ◯ No [A'a]

20. Did you seek help?

[Kin nemi taimako?]

- ◯ Yes [Eh]
- ◯ No [A'a]

21. Where/from who did you first seek help?

[A ina/wurin wa kika fara neman taimako?]

- ◯ Dispensary/health center/clinic [N-primary] [Dakin jinya /karamin asibit]
- ◯ Hospital [N-secondary/tertiary] [Asibiti]
- ◯ Chemist/Pharmacy [Dakin sayar da magani]
- ◯ Community health worker/volunteer [Ma'aikacin lafiya na sa-kai]
- ◯ Non CHW TBA [if TBA & CHW, categorize as CHW]
- ◯ Friend/family/neighbor [Aboki/abukiya/iyali/makwabci/makwabciya]
- ◯ Traditional healer/faith healer [Mai maganin gargajiya /mai addu'a]
- ◯ Other [In da wata hanya a yi bayani]

Other

[In da wata hanya a yi bayani]

____

22. During your pregnancy or since you had your baby have you had a fever?

[Lokacin cikinki ko tunda kika haifu kin taba samun masassara?]

- ◯ Yes [Eh]
- ◯ No [A'a]

23. Did you seek help?

[Kin nemi taimako?]

- ◯ Yes [Eh]
- ◯ No [A'a]

24. Where/from who did you first seek help?

[A ina/wurin wa kika fara neman taimako?]

- ◯ Dispensary/health center/clinic [N-primary] [Dakin jinya /karamin asibit]
- ◯ Hospital [N-secondary/tertiary] [Asibiti]
- ◯ Chemist/Pharmacy [Dakin sayar da magani]
- ◯ Community health worker/volunteer [Ma'aikacin lafiya na sa-kai]
- ◯ Non CHW TBA [if TBA & CHW, categorize as CHW]
- ◯ Friend/family/neighbor [Aboki/abukiya/iyali/makwabci/makwabciya]
- ◯ Traditional healer/faith healer [Mai maganin gargajiya /mai addu'a]
- ◯ Other [In da wata hanya a yi bayani]

Other

[In da wata hanya a yi bayani]

____

25. Did you seek help for any other problems during your pregnancy?

[Kin taba neman wani taimako saboda wata matsala wadda bat a shafi cikinki ba?]

- ◯ Yes [Eh]
- ◯ No [A'a]

26. Did you seek help?

[Kin nemi taimako?]

- ◯ Yes [Eh]
- ◯ No [A'a]

27. Where/from who did you first seek help?

[A ina/wurin wa kika fara neman taimako?]

- ◯ Dispensary/health center/clinic [N-primary] [Dakin jinya /karamin asibit]
- ◯ Hospital [N-secondary/tertiary] [Asibiti]
- ◯ Chemist/Pharmacy [Dakin sayar da magani]
- ◯ Community health worker/volunteer [Ma'aikacin lafiya na sa-kai]
- ◯ Non CHW TBA [if TBA & CHW, categorize as CHW]
- ◯ Friend/family/neighbor [Aboki/abukiya/iyali/makwabci/makwabciya]
- ◯ Traditional healer/faith healer [Mai maganin gargajiya /mai addu'a]
- ◯ Other [In da wata hanya a yi bayani]

Other

[In da wata hanya a yi bayani]

____

ANC Mothers have increased knowledge of maternal and newborn health

28. When you went for antenatal care, did a health care provider (or group leader) talk with you about

[Da kina zuwa awo da renon ciki ko shi ma'aikacin lafiya (ko shigaban kungiya) ya taba yi maki magana game da:]

a. Options for family planning/child spacing

[Hanyoyin tsara iyali/sa tazara tsakanin haifuwa]

- ◯ Yes [Eh]
- ◯ No [A'a]
- ◯ Not sure [Ban tuna ba]

b. How to use breastfeeding to prevent another pregnancy right away

[Hanyar shayar da nonon uwa a matsayin hana daukar ciki]

- ◯ Yes [Eh]
- ◯ No [A'a]
- ◯ Not sure [Ban tuna ba]

c. When to begin breastfeeding

[Yaushe ya kamata a fara ba yaro nonon uwa bayan haifuwa]

- ◯ Yes [Eh]
- ◯ No [A'a]
- ◯ Not sure [Ban tuna ba]

d. When to introduce other fluids and foods to your baby?

[Lokacin da za fara ba jariri wani abinci ko abin sha bandaa nonon uwa?]

- ◯ Yes [Eh]
- ◯ No [A'a]
- ◯ Not sure [Ban tuna ba]

e. Danger signs or warning signs to watch for during pregnancy?

[Alamun hadurra da ya kamata a lura da su lokacin juna biyu?]

- ◯ Yes [Eh]
- ◯ No [A'a]
- ◯ Not sure [Ban tuna ba]

f. Returning to the clinic if you noticed any danger signs, warning signs, or had other problems?

[Ya kamata ki dawo asibiti da zarar kin ji wasu alamun hadari ko kuma kin sami wata matsala?]

◯ Yes [Eh]
◯ No [A'a]
◯ Not sure [Ban tuna ba]

g. Eating extra food while pregnant and breastfeeding

[Ya kamata ki rinka cin abinci a kai-a kai lokacin juna-biyu da lokacin bad a mama]

◯ Yes [Eh]
◯ No [A'a]
◯ Not sure [Ban tuna ba]

h. Danger signs or problems to watch for in your newborn?

[La'akari alamun hadari ko wasu matsaloli da ke iya samun jaririnki?]

◯ Yes [Eh]
◯ No [A'a]
◯ Not sure [Ban tuna ba]

i. How to prevent transmission of sexually transmitted infections

[Hanyar kare kai daga kamuwa da cututtuka da ake dauka ta wurin jima'i]

◯ Yes [Eh]
◯ No [A'a]
◯ Not sure [Ban tuna ba]

j. The importance of sleeping under a bed net

[Muhimmancin kwana karkashin gidan sauro]

◯ Yes [Eh]
◯ No [A'a]
◯ Not sure [Ban tuna ba]

k. The importance of taking iron and folic acid tablets (blood tonic)

[Muhimmancin shan magunguna masu gina kasha da bargo]

◯ Yes [Eh]
◯ No [A'a]
◯ Not sure [Ban tuna ba]

l. The importance of making a birth plan and what to plan for

[Muhimmancin tsara haifuwa da kuma shirya matakan dauka]

◯ Yes [Eh]
◯ No [A'a]
◯ Not sure [Ban tuna ba]

29. Do you think there are advantages to spacing pregnancies apart?

[Kina ganin akwai alfano gas a tazara tsakanin haifuwa da daukar ciki na gaba?]

◯ Yes [Eh]
◯ No [A'a]
◯ Not sure [Ban sani ba]

30. What are some advantages?

[Mine ne alfanon?]

- ☐ Mom will be healthier/ less likely to have problems [Uwa za ta sami isashiyar lafiya kuma da wuya ta smi matsaloli lokacin juna biyu]
- ☐ Baby will be healthier/ less likely to have problems [Wannan jariri da ma masu biyowa bayan shi za su sami koshin lafiya kuma da wuya a sami haifuwar dan da bai isa haifuwa ba]
- ☐ Easier to feed and/or take care of other children [Za a sami saukin ciyarwa da luraa da yara]
- ☐ Saves money [Za a iya tsimin kudi]
- ☐ Other [In da wani a yi bayani]

(Do not prompt. Probe, any more? Select all items mentioned)

Other specify

[In da wani a yi bayani]

____

31. How long should pregnancies be spaced apart for the best health of the mother and baby

[Mine ne tsawon lokacin dauka tsakanin haifuwa da ciki don samun koshin lafiya ga uwa da jariri?]

____
(Enter in Months (1 yr=12 months; 2 yrs = 24 months; 3 yrs = 36 months))

32. When do you think it is best to first breastfeed your baby?

[Yaushe kike tsammani ya yi daidai ki fara ba jaririnki nonon uwwa?]

- ○ Immediately or within 1 hour of delivery [Nan take ko kuma kada a wuce awa daya bayan haifuwa]
- ○ After the first milk has gone - when the true milk comes in [Bayan an tatse nonon farko (llokacin da nonon kkwarai ya hadu)]
- ○ Other [In da wani a bayana]

([read response options?])

Other

[In da wani a bayana]

____

33. At what age do you think it is best to start giving your baby fluids or food other than breastmilk?

[Awane lokaci ne kike ganin ya dace ki fara ba jaririnki wani abinci ko kunu, watau wani daban da nonon uwa?]

Weeks [Sati] ______________________________

Months [Wata] ______________________________

33a. Other descriptors/comments [Wani bayani/ra'ayi] ______________________________

34. Have you ever heard of using the lactational amenorrhea method, LAM, as a way to delay pregnancy after you have a baby? This method includes breastfeeding your baby.

[Kin taba sanin ana amfani da shayar da nonon uwa a matsayin hanyar jinkirta daukar ciki bayan haifuwa?]

- ○ Yes [Eh]
- ○ No [A'a]
- ○ Not sure [Ban tuna ba]

35. Can you describe how to use lactational amenorrhea method (LAM)? What criteria or conditions need to be met for it to be an effective way to prevent or delay pregnancy?

[Kina iya bayana yadda ake amfani da shayar da nonon uwa a matsayin hanyar jinkirta daukar ciki (LAM)? Wadanne kaidodi ne za a bi don wannan hanya ta yi nasarar jinkirta ko hana daukar ciki?]

- ☐ Menstrual bleeding has not returned [Jinin al'ada bai dawo ba tukuna bayan haifuwa]
- ☐ Baby is only breastfed/breastfed exclusively (no other food or liquids given) [Ana ba jariri nonon uwa kadai (ba wani nau'in abinci ko mai ruwa-ruwa)]
- ☐ Baby is less than six months old [Jariri bai kai wata shida ba tukuna]
- ☐ Other [In da wata hanya a bayana]

(Check all that are mentioned. Prompt with 'anything else'.)

Other specify

[In da wata hanya a bayana]

____

36. After the birth of a child, can a woman become pregnant before her menstrual period has returned?

[Bayan haifuwa, mace tana iya daukar ciki kafin jinin al'adarta na wata-wata ya dawo?]

- ◯ Yes [Eh]
- ◯ No [A'a]
- ◯ Don't know [Ban sani ba]

37. In addition to the lactational amenorrhea method (LAM) what other forms of birth spacing or family planning can you use immediately after delivery?

[Banda hanyar shayar da nonon uwa wadanne hanyoyi ne kuma kike iya amfani da su nan take bayan haifuwa don tsara iyali ko sa tazara tsakanin haifuwa da daukar ciki?]

- ☐ Standard days or rhythm method (cycle beads/calendar/billing's method) [Kebabbun ranakku bias ga sanin lokacin al'adar mace ta wata-wata]
- ☐ Withdrawal [Cire zakari kafin fitar maniyi]
- ☐ Condoms [Kwaroron roba]
- ☐ Pill/oral contraceptives [Kwayoyin hadiya]
- ☐ Injectable [Allura]
- ☐ Implants [Abubuwan da ake sawa karkashin fata]
- ☐ IUD [Na'urar sakawa a mahaifa]
- ☐ Emergency contraception [Matakin hana ciki na gaggawa]
- ☐ Sterilization [Daddaka]
- ☐ Other [In da wani a bayanaer]
- ☐ None [Babu]
- ☐ Don't Know [Ban sani ba]

Other

[In da wani a bayanaer]

____

38. What are danger/warning signs for you, the mother, after the baby is born? These are things that mean there might be a problem and that you should see a health care worker.

[Wadanne alamu ne na hadurra gareki ke uwa bayan kin haifi jaririnki? Wannan yana nufin ana iya samun matsala kuma ya kamata ki ga ma'aikacin lafiya.]

- ☐ Heavy bleeding [Zubar jinni da yawa]
- ☐ Severe abdominal (stomach) pain [Matsanancin ciwon ciki (tumbi)]
- ☐ Severe pain and/or read and hot breasts [Nono ya yi ciwo mai tsanani, ya yi jay a kuma yi zafi]
- ☐ Severe headache [Ciwon kai mai tsanani]
- ☐ Visual disturbances [Rashin gani da kyau]
- ☐ Fever [Masassara]
- ☐ Unexplained pain in chest or legs [Dimewar ciwon kirji da kafafu]
- ☐ Breathing difficulties [Sheda da wuya]
- ☐ Difficult or painful urination [Ciwo lokacin fitsari]
- ☐ Other [In da wani a bayana]
- ☐ Don't know [Ban sani ba]

(Check all that are mentioned. Prompt with "any others". Do not read options.)

Other specify ____________________

[In da wani a bayana]

39. What are danger/warning signs for your newborn? These are things that mean there might be a problem and that you should see a health care worker.

[Wadanne alamu ne na hadurra ga jaririnki? Wadannan alamu suna nufin ana iya samun matsala kuma ya kamata ki ga ma'aikacin lafiya.]

- ☐ Too hot or too cold [Jikinsa yana zafi kwarai ko kuma yana sanyi kwarai]
- ☐ Convulsions [Jijjiga]
- ☐ Little or no movement [Jariri yana motsi kadan-kadan ko kuma bai motsi sam-sam]
- ☐ Feeding poorly or not at all [Ba ya marmarin abinci ko ba ya cin abinci sam-sam]
- ☐ Fast breathing or chest indrawing [Sheda da sauri ko fadawar kirji]
- ☐ Not passing urine and/or stool [Kasa kasha ko fitsari]
- ☐ Umbilical cord stump is bleeding; has foul odor; or redness around it [Cibiya tana jini ko tana wari ko kuwa ta yi ja]
- ☐ Very small baby born at home [Dan mitsitsin jariri kuma an haife a gida]
- ☐ Yellow palms or soles of feet [Tafin hannuwa ko kafafu su yi kore-kore]
- ☐ Other [In da wani a yi bayani]
- ☐ Don't know [Ban sani ba]

(Check all that are mentioned. Prompt with "any others". Do not read options.)

Other specify ____________________

[In da wani a yi bayani]

40. What are the most important things you can do to help keep your newborn healthy?

[Wane muhimman abubuwa ne za ki don bayar da kyakkyawar lafiya ga jaririnki?]

- ☐ Keep warm (keep head and body covered; skin to skin with mom) [A kare jariri daga sanyi (a rufe kansa da jikinsa kuma uwarsa ta rungume shi)]
- ☐ Feed only on breastmilk [A shayar das hi da nonon uwa kadai]
- ☐ Feed often/whenever baby wants [A shayar da jariri a kai-a kai/ a duk lokacin day a so]
- ☐ Do not put anything on cord stump [Kada a sa kome bisa cibiyarsa]
- ☐ Keep cord stump clean [Cibiyarsa ta zama da tsabta ko da yaushe]
- ☐ Wash hands [A wanke hann ko da yaushe]
- ☐ Have baby immunized [A yi wa jariri riga-kafi]
- ☐ Wait atleast 24 hours before bathing baby [A jira sai bayan awa 24 kafin a yi wa jariri wanka]
- ☐ Other [In da wani a yi bayani]
- ☐ Don't know [Ban sani ba]

(Check all that are mentioned. Prompt with "any others". Do not read options.)

Other specify ____________________

[In da wani a yi bayani]

Increased uptake of entire ANC package

41. Were you given a mosquito net during ANC care for this pregnancy?

[Ko an ba ki gidan sauro lokacin awo da renon wannan ciki?]

◯ Yes [Eh]
◯ No [A'a]
◯ Not sure [Ban tabbata ba]

42. Did you sleep under a bed net last night?

[Kin kwana cikin gidan sauro a daren jiya?]

◯ Yes [Eh]
◯ No [A'a]
◯ Not sure [Ban tabbata ba]

43. Did your baby sleep under a bed net last night?

[Ko jaririnki ya kwana cikin gidan sauro a daren jiya?]

◯ Yes [Eh]
◯ No [A'a]
◯ Not sure [Ban tabbata ba]

44. Did you have an HIV test and receive the results during antenatal care?

[Kin yi gwajin cutar kanjamau kuma kin karbi sakamako lokacin awo da renon ciki]

◯ Yes [Eh]
◯ No [A'a]
◯ Not sure [Ban tabbata ba]

45. Did you know your HIV status before antenatal care?

[Kin san matsayinki game da cutar kanjamau kafin awo da renon ciki?]

◯ Yes [Eh]
◯ No [A'a]
◯ Not sure [Ban tabbata ba]

46. Were you given iron and folic acid tablets (blood tonic) during ANC care?

[An ba ki kwayoyin magungunan gina kashi da bargo lokacin awo da renon ciki (blood tonic)?]

◯ Yes [Eh]
◯ No [A'a]
◯ Not sure [Ban tabbata ba]

47. Did you run out of tablets at any time during your pregnancy

[Magungunan sun taba kasa maki lokacin juna biyunki]

◯ Yes [Eh]
◯ No [A'a]
◯ Not sure [Ban tabbata ba]

48. Were you given additional iron and folic acid tablets (blood tonic) prior to delivery?

[An kara maki wadannan magunguna (blood tonic)kafin ki haifu?]

◯ Yes [Eh]
◯ No [A'a]
◯ Not sure [Ban tabbata ba]

49. Did you take an iron and folic acid tablet (blood tonic) yesterday?

[Kin sha magungunan gina kasha da bargo(blood tonic) jiya?]

◯ Yes [Eh]
◯ No [A'a]
◯ Not sure [Ban tabbata ba]

50. How long after birth did you first put your baby to the breast?

[Wane tsawon lokaci kika dauka bayan haifuwa kafin ki ba jaririnki nonon uwa?]

◯ Not applicable, did not breastfeed [Bai shafe ni ba, ba na shayarwa]
◯ Less than one hour [Kafin awa daya bayan haifuwa]
◯ Less than 24 hours [Kafin awa 24]
◯ >24 hours [Bayan awa 24]

Specify hours for 'less than 24 hours choice'

[Kafin awa 24 , yi bayani awa]

____

Specify number of days for '> than 24 hours choice'

[Bayan awa 24 , yi bayanin yawan kwanaki]

____

Increased number of clients reporting satisfaction with ANC services provided

51. Thinking about the antenatal care you received during this pregnancy and as best as you can remember, do you agree, disagree or neither agree nor disagree with each of these statements

[Bisa ga awo da renon ciki da kika samu lokacin wannan juna biyu da kuma yadda kike iya tunawa a baya, za ki amince da wadannan ra'ayoyi:]

a. I was greeted warmly

[An karbe ni da fara'a]

◯ Agree ◯ Disagree ◯ Neither Agree or Disagree

b. I was able to ask questions and they were answered in a way I understood

[Na samu zarafin yi tambayoyi kuma an ba ni amsa yadda na fahimta]

◯ Agree ◯ Disagree ◯ Neither Agree or Disagree

c. I learned important information about how to keep myself and my baby healthy

[Na koyi muhimman hanyoyin kiyaye lafiyata da lafiyar jaririna]

◯ Agree ◯ Disagree ◯ Neither Agree or Disagree

d. I left antenatal care visits with questions or confused about some things

[Na bar zuwa awon ciki don abubuwa da yawa ban fahimce su ba]

◯ Agree ◯ Disagree ◯ Neither Agree or Disagree

e. The provider did not show me respect or act in respectful ways

[Mai ba da renon ciki bai mutunta ni ba kuma bai nuna mutunci ba]

◯ Agree ◯ Disagree ◯ Neither Agree or Disagree

f. During my next pregnancy I would be happy to receive care similar to what I received during this past pregnancy

[A wannan cikin zan yi murna idan zan sami kulawa kamar yadda na samu lokacin cikina na baya]

◯ Agree ◯ Disagree ◯ Neither Agree or Disagree

g. I would recommend to a friend to go to the same place for antenatal care

[Zan ba abukiyata shawara ta je wannan wurin domin awo da renon cikinta]

◯ Agree ◯ Disagree ◯ Neither Agree or Disagree

h. I felt respected

[Na ji an mutunta ni]

◯ Agree ◯ Disagree ◯ Neither Agree or Disagree

i. I had enough time with my provider

[Na sami isassehen lokaci da mai renon cikina]

◯ Agree ◯ Disagree ◯ Neither Agree or Disagree

j. I am very satisfied with the antenatal care I received

[Na gamsu sosai da renon ciki da na samu]

◯ Agree ◯ Disagree ◯ Neither Agree or Disagree

52. Imagine you were to get pregnant again. If you could choose, would you prefer individual one on one antenatal visits or would you prefer group visits where you receive your care with other women who are at a similar time in their pregnancy?

[A ce za ki sake daukar ciki, za ki zabi awo na daga ke sai mai renon ciki (awon daidaiku) ko kuwa za ki zabi awo cikin taro tare da mata masu ciki daidai lokaci da ke?]

◯ Individual care [Awo da renon ciki daidaiku] ◯ Group care [Awo da renon ciki cikin taro]

Intervention arm only

53. Thinking about the antenatal care you received in a group, do you agree, disagree or neither agree nor disagree with each of these statements ?

[Bisa ga awo da renon ciki da kika samu cikin taro, za ki amince da wadannan ra'ayoyi ?]

a. I was comfortable receiving antenatal care in a group

[Na ji dadi da walwala zuwa awo da renon ciki cikin taro]

◯ Agree ◯ Disagree ◯ Neither Agree or Disagree

b. I was worried about privacy during group visits

[Na damu da rashin sirri lokacin awo da renon ciki cikin taro]

◯ Agree ◯ Disagree ◯ Neither Agree or Disagree

c. I felt like I was part of the group

[Na ji kamar dukanmu daya muke cikin taron]

◯ Agree ◯ Disagree ◯ Neither Agree or Disagree

d. I was comfortable talking and sharing thoughts with the group

[Na sami sakewa da walwalar musayar ra'ayi da sauran mas awo]

◯ Agree ◯ Disagree ◯ Neither Agree or Disagree

e. Being in a group made it difficult to ask some questions I had

[Da yake cikin taro ne ban iya in yi wasu tambayoyi da na so yi ba]

◯ Agree ◯ Disagree ◯ Neither Agree or Disagree

f. Women in the group listened to me

[Mataye sun saurare ni a cikin taron]

◯ Agree ◯ Disagree ◯ Neither Agree or Disagree

g. The group leader talked at us or instructed us most of the time

[Shugaban taron ya dauki dogon lokaci yana bayani]

◯ Agree ◯ Disagree ◯ Neither Agree or Disagree

h. The group leader talked too much

[Shugaban taro ya yi magana da yawa]

◯ Agree ◯ Disagree ◯ Neither Agree or Disagree

i. All women in the group had the chance to talk most visits

[Dukan mataye sukan sami zarafin yin magana lokacin awo da renon ciki]

◯ Agree ◯ Disagree ◯ Neither Agree or Disagree

j. Mostly we, the pregnant women answered each others questions

[Yawancinmu, mata masu ciki mukan amsa tambayoyin junanmu]

◯ Agree ◯ Disagree ◯ Neither Agree or Disagree

k. Mostly the group leader, not other members of the group answered our questions.

[Yawancin lokaci shugaban taro ne ke amsa tambayoyinmu, ba wasu daga cikin taro ba]

◯ Agree ◯ Disagree ◯ Neither Agree or Disagree

l. I learned a lot from other women in the group

[Na koyi abu da yawa daga wasu mataye cikin taro]

◯ Agree ◯ Disagree ◯ Neither Agree or Disagree

m. The group changed the way I think about some things

[Zuwa cikin taron ya canja tunanina game da wasu abubuwa]

◯ Agree ◯ Disagree ◯ Neither Agree or Disagree

All women: Increased self-efficacy/empowerment of pregnant women

54. Who will usually make decisions about health care for yourself and your newborn?

[Wake daukar matakan kiwon lafiyarki da ta jaririnki?]

◯ You [Ke]
◯ Your husband/partner [Mijinki]
◯ You and your husband/partner jointly [Ke tare da mijinki]
◯ Someone else [Wani daban]
(read responses select one)

Someone else specify

[Wani daban, a yi bayani]

____

55. Could you ask your husband/partner to use a condom if you wanted him to?

[Idan kina son mijinki ya yi amfani da kwaroron roba kina iya ce masa ya yi amfani da shi?]

◯ Yes [Eh]
◯ No [A'a]
◯ Not sure/Depends [Wani daban/Ya danganta]

56. Would you say that using (or not using) contraception is mainly your decision, mainly your husband's/partner's decision, or did you both decide together?

[Kina iya cewa amfani da hanyoyin hana daukar ciki ko rashin amfani da su niyarki ce ko niyar mijinki ko kuwa niyarku gaba daya?]

◯ Your decision [Niyarki]
◯ Your husband/partners decision [Niyar mijinki]
◯ Joint decision [Niyarku tare]
◯ Other [In ba haka ba a yi bayani]

Other Specify

[In ba haka ba a yi bayani]

____

57. Thinking about your next pregnancy, do you agree, disagree or neither agree nor disagree with the following statements?

[Game da cikinki na gaba za ki amince da wadannan ra'ayoyi?]

a. If I don't understand something a provider is telling me I will tell them and ask them to explain a different way

[Idan ban gane abinda ma'aikacin asibiti ke cewa ba zan fada masa kuma in nemi ya yi mani bayani yadda zan gane]

◯ Agree ◯ Disagree ◯ Neither Agree or Disagree

b. There are things I can do to help prevent problems and keep myself and my baby healthy

[Akwai abubuwan da zan yi don taimakawa ga riga-kafin matsaloli in kare lafiyata da ta jaririna]

◯ Agree ◯ Disagree ◯ Neither Agree or Disagree

c. I will talk with my husband/family about how to keep myself and our baby healthy

[Zan yi magana da mijina da iyalina game da yadda zan tsare lafiyata da ta jaririnmu]

◯ Agree ◯ Disagree ◯ Neither Agree or Disagree

d. I know how to recognize a problem with my pregnancy

[Ina iya ganewa idan akwai matsala ga cikina]

◯ Agree ◯ Disagree ◯ Neither Agree or Disagree

e. I know what actions I will take if I think there is a problem with my pregnancy

[Na san matakan da zan dauka idan na fahimci cewa akwai matsala ga cikina]

◯ Agree ◯ Disagree ◯ Neither Agree or Disagree

If yes, I will be able to take those actions if there is a problem

◯ Agree ◯ Disagree ◯ Neither Agree or Disagree

f. I know how to recognize a problem with my newborn

[Idan Eh, zan iya daukar wadannan matakan idan akwai matsala]

◯ Agree ◯ Disagree ◯ Neither Agree or Disagree

g. I know what actions I will take if I think there is a problem with my newborn

[Na san yadda zan gane idan akwai matsala ga jaririna]

◯ Agree ◯ Disagree ◯ Neither Agree or Disagree

h. I am good at making decisions related to the health of myself and my family

[Na iya daukar matakai game da lafiyata da lafiyar iyalina]

◯ Agree ◯ Disagree ◯ Neither Agree or Disagree

58. Thinking about life generally, do you agree or disagree with the following statements?

[Bisa ga rayuwa da zamantakewa za ki amince da wadannan ra'ayoyi?]

a. I am comfortable talking about difficult subjects

[Ina iya yin magana da mutane a kan matsaloli masu sarkakiya]

◯ Agree ◯ Disagree

b. I am comfortable letting others know what I really think, even if it is different from them

[Ina iya fada ma mutane ra'ayina ko da ya saba nasu]

◯ Agree ◯ Disagree

c. I like to make decisions for myself

[Ina so in dauki mataki da kaina game da abinda ya shafe ni]

◯ Agree ◯ Disagree

d. I am able to make decisions for myself

[Na isa in dauki mataki don kaina]

◯ Agree ◯ Disagree

Increased uptake of skilled birth attendance/delivery by SBA

59. Before your baby was born did you

[Kafin haifuwar jaririnki kin]

a. Identify a facility where you planned to give birth?

[Zabi wurin samun taimako inda kin shirya za ki haifu?]

◯ Yes [Eh] ◯ No [A'a]

b.Plan how you were going to get there?

[Shirya yadda za ki isa wurin?]

◯ Yes [Eh] ◯ No [A'a]

c. Plan who was going to go with you/help you?

[Shirya wanda zai tafi tare da ke don ya taimake ki?]

◯ Yes [Eh] ◯ No [A'a]

d. Save money in case of an emergency?

[Tanadi kudi don yanayi gaggawa?]

◯ Yes [Eh] ◯ No [A'a]

e. Decide who could make decisions in the case of emergency?

[Shirya wanda zai dauki shugabanci idan akwai yanayi gaggawa?]

◯ Yes [Eh] ◯ No [A'a]

e_i. Decide who else could make decisions if they weren't around?

[Shirya wani da zai iya taimakawa idan wancan ba ya nan?]

◯ Yes [Eh] ◯ No [A'a]

f. Prepare a birth kit

[Shirya dan akwatin kayan karban haifuwa]

◯ Yes [Eh] ◯ No [A'a]

60. Where was your baby born?

[A ina aka haifi jaririnki?]

◯ At home [A gida]
◯ At a health Facility [A asibiti]
◯ In transit [Kan hanyar zuwa asibiti]
◯ Other [In wani a bayana]

Other, specify

[In wani a bayana]

____

61. Did antenatal care influence your place of delivery?

[Ko kin zabi wurin haifuwa bisa zuwa awo da renon ciki da kika samu?]

◯ Yes [Eh]
◯ No [A'a]
◯ Not sure [Ban tuna ba]

62. Have you already gone to a clinic/hospital for postnatal care (a check up for you and the baby?)

[Kin riga kin je asibiti bayan kin haifu don lura da jaririnki (binciken lafiyarki da lafiyar jaririnki?)]

◯ Yes [Eh]
◯ No [A'a]

63. If yes, how many days after you delivered did you go?

[Idan Eh, kwana nawa bayan haifuwa kika je?]

____
(Enter a number)

64. Did you go for postnatal care a second time?

[Kin sake zuwa ganin likita bayan haifuwa zuwa na biyu?]

◯ Yes [Eh]
◯ No [A'a]

65. If yes, how many days after you delivered did you go?

[Idan Eh, kwana bayan haifuwa kika je zuwa na biyu?]

____

Knowledge/counseling/plans: PPFP

66. Do you agree, disagree or neither agree nor disagree with each of these statements?

[Kin amince da wadannan ra'ayoyi?]

a. During ANC care I was told about different methods I could use to space or prevent my next pregnancy

[Lokacin awo da renon ciki an koya mani hanyoyi daban daban zan yi amfani da su in jinkirta ko in hana shigar ciki na gaba]

◯ Agree ◯ Disagree ◯ Neither Agree or Disagree

b. During ANC care I received all the information I needed to make a decision about if and how to space my next pregnancy

[Lokacin awo da renon ciki na sami dukan bayanai da nake bukata don sa tazara tsakanin haifuwa da daukar ciki na gaba]

◯ Agree ◯ Disagree ◯ Neither Agree or Disagree

c. During ANC care I was given the opportunity to ask questions about different ways to space pregnancies

[Lokacin awo da renon ciki na sami zarafin yi dukan tambayoyi game da yadda sa tazara tsakanin haifuwa da ciki na gaba]

◯ Agree ◯ Disagree ◯ Neither Agree or Disagree

d. All of my questions were answered in a way I understood

[Dukan tambayoyina an amsa mani kuma na fahimta]

◯ Agree ◯ Disagree ◯ Neither Agree or Disagree

e. I can have some control over if and when I get pregnant again

[Zan iya lura da kaina gwargwado idan na sami ciki nan gaaba]

◯ Agree ◯ Disagree ◯ Neither Agree or Disagree

67. If it was up to you, would you like to have another child?

[Idan zabinki ne, za ki so ki sake haifuwa?]

◯ Yes [Eh]
◯ No [A'a]
◯ Undecided [Ban sani ba]

68. If it was up to you, would you want to delay getting pregnant again right away?

[Idan zabinki ne za ki so ki fara jinkirta daukar ciki tun yanzu?]

◯ Yes [Eh]
◯ No [A'a]
◯ Undecided [Ban sani ba]

69. During antenatal care, did you select a form of family planning to use after your baby was born?

[Lokacin da kike awo da renon ciki kin zabi irin hanyar da za ki bi don tsarin iyali bayan haifuwar jaririnki?]

◯ Yes [Eh]
◯ No [A'a]

70. What form of family planning did you choose during antenatal care?

[Wane iri hutun haihuwa kika zaba lokacin da kikaje awon ciki?]

◯ LAM/breastfeeding [Tsarin bayar da mamar uwa don hana ciki]
◯ Standard days or rhythm method (Calendar/rhythm/cycle beads/natural/Billing's method) [Kebabbun ranakku ko tsarin daki-daki bisa ga sanin lokacin al'adar wata-wata]
◯ Withdrawal [Cirewa kafin fitar maniyi]
◯ Condoms [Kwaroron roba]
◯ Pill/Oral contraceptives [Kwayoyin hadiya]
◯ Injectable [Allura]
◯ Implants [Abubuwan da ake sawa karkashin fata]
◯ IUD [Na'urar sakawa a mahaifa]
◯ Emergency contraception [Matakin hana ciki na gaggawa]
◯ Sterilization [Daddaka]
◯ Other [Ib da wani a bayanaer]

Other specify

[Ib da wani a bayanaer]

____

71. Are you currently using it?

[Kina amfani da wannan hanyar a yanzu?]

◯ Yes [Eh]
◯ No [A'a]

72. Are you using another method?

[Kina amfani da wani tsari na daban?]

◯ Yes [Eh]
◯ No [A'a]

73. What method are you currently using?

[Wace hanya kike amfani da ita a yanzu?]

◯ LAM/breastfeeding [Tsarin bayar da mamar uwa don hana ciki]
◯ Standard days or rhythm method (Calendar/rhythm/cycle beads/natural/Billing's method) [Kebabbun ranakku ko tsarin daki-daki bisa ga sanin lokacin al'adar wata-wata]
◯ Withdrawal [Cirewa kafin fitar maniyi]
◯ Condoms [Kwaroron roba]
◯ Pill/Oral contraceptives [Kwayoyin hadiya]
◯ Injectable [Allura]
◯ Implants [Abubuwan da ake sawa karkashin fata]
◯ IUD [Na'urar sakawa a mahaifa]
◯ Emergency contraception [Matakin hana ciki na gaggawa]
◯ Sterilization [Daddaka]
◯ Other [Ib da wani a bayanaer]

Other specify

[Ib da wani a bayanaer]

____

74. Why didn't you select a method of birth spacing during antenatal care?

[Me ya sa ba ki zabi hanyar sa tazara tsakanin haifuwa ba tun lokacin da kike awo da renon ciki ba?]

- ◯ I don't want to space my next pregnancy/want to get pregnant again right away [Ba na son jinkirta cikina na gaba /Ina son in dauki ciki yanzu haka]
- ◯ I didn't know what the options were/wasn't counseled/my provider didn't talk to me about it [Ba ni da masaniya kan hanyoyin zabi/ ba a ba ni shawara ba/ mai awo da renon cikina bai yi mani bayani ba game da su]
- ◯ I couldn't decide [Ban iya na dauki mataki ba]
- ◯ Other [In da wata hanya a yi bayani]

(Don't read options, select most appropriate.)

Other

[In da wata hanya a yi bayani] ______________________________

Any other comments?

[A kwai wani bayani ko ra'ayoyi?] ______________________________
