# Supplementary material for: Group versus individual antenatal and first year postpartum care: Study protocol for a multi-country cluster randomized controlled trial in Kenya and Nigeria
Source: Gates Open Res. 2019 Jul 4;2:56. Originally published 2018 Nov 5. [Version 2] doi: 10.12688/gatesopenres.12867.2 (PMC6350506; doi:10.12688/gatesopenres.12867.2)
Supplement: Supplementary file 1 [file gatesopenres-2-14160-s0000.tgz › 72242879-e244-48c8-a71c-b86050fb7704_Supplemental_files-_Study_manual,_Data_tools,_and_recruitment_,materials.zip!/Supplemental files- Study manual, Data tools, and recruitment ,materials/Phase I Survey_ANC Provider.pdf]

Anc Provider Survey

Date and Time of record creation - Data quality check ________________

ANC Survey - Latitude ________________

ANC Survey - Longitude ________________

Date of data collection/ interview ________________

First and Last Name of data collector ________________

Participant is:
- ◯ ANC Provider (midwife, nurse, physician, clinical officer)
- ◯ CHEW or CHW

Health care workers reporting job satisfaction

1. Please give me a few words to describe your feelings towards providing group ANC care ________________

2. Thinking specifically about the group ANC you have been providing, do you agree, disagree or neither agree nor disagree with each of theses statements.

a. I am able to provide the level of quality in antenatal care that I would like to provide.

◯ Agree ◯ Disagree ◯ Neither Agree or Disagree

b. During antenatal care I have enough time with my patients

◯ Agree ◯ Disagree ◯ Neither Agree or Disagree

c. In group visits I am able to answer questions in a way the women understand

◯ Agree ◯ Disagree ◯ Neither Agree or Disagree

d. I feel respected by the women in my groups

◯ Agree ◯ Disagree ◯ Neither Agree or Disagree

e. During antenatal care I have the time to teach important information about how women can keep themselves and their babies healthy

◯ Agree ◯ Disagree ◯ Neither Agree or Disagree

f. I would recommend to a friend to go to my clinic for group antenatal care

◯ Agree ◯ Disagree ◯ Neither Agree or Disagree

g. Sometimes I worry that patients leave their group visits with unanswered questions or confused about some things

◯ Agree ◯ Disagree ◯ Neither Agree or Disagree

h. I am always able to be respectful towards my patients during group visits

◯ Agree ◯ Disagree ◯ Neither Agree or Disagree

i. I find providing group antenatal care highly satisfying

◯ Agree ◯ Disagree ◯ Neither Agree or Disagree

3. On a scale of 1 - 5, with 1 being extremely dissatisfied and 5 being extremely satisfied, how would you rate your current job satisfaction specifically in relation to providing group ANC care?
(The rating is between 1 - 5.)

◯ 1 ◯ 2 ◯ 3 ◯ 4 ◯ 5

4. On a scale of 1 - 5, with 1 being not at all and 5 being a lot, how much do you enjoy providing antenatal care with the group model?
(The rating is between 1 - 5.)

◯ 1 ◯ 2 ◯ 3 ◯ 4 ◯ 5

5. How do you prefer providing antenatal care?

◯ Through the group based model
◯ Through standard individual care
◯ Both equally
◯ Not applicable, I don't provide individual care

Feasibility

6. Were you able to attend and facilitate all the group visits for the cohorts (groups) you were assigned to?

◯ Yes
◯ No

7. Did you have a co-facilitator?

◯ Yes
◯ No

8. Why not? Specify

____

9. What designation did your co-facilitator have?

◯ CHEW
◯ CHW
◯ Doctor
◯ Midwife
◯ Nurse
◯ Clinical officer
◯ Other

Other specify

____

10. Would you recommend that other facilities use CHEWs as co-facilitators for groyp based antenatal care?

◯ Yes
◯ No
◯ Unsure

11. Do you think its feasible to implement a group based care model in your facility?

◯ Yes
◯ No
◯ Not sure

For all responses: why?

____

12. Do you personally want to provide group based care?

◯ Yes
◯ No
◯ Not sure

For all responses: why?

____

Any other comments?

____

THANK THE PARTICIPANT AND END THE INTERVIEW
