# Supplementary material for: Group versus individual antenatal and first year postpartum care: Study protocol for a multi-country cluster randomized controlled trial in Kenya and Nigeria
Source: Gates Open Res. 2019 Jul 4;2:56. Originally published 2018 Nov 5. [Version 2] doi: 10.12688/gatesopenres.12867.2 (PMC6350506; doi:10.12688/gatesopenres.12867.2)
Supplement: Supplementary file 1 [file gatesopenres-2-14160-s0000.tgz › 72242879-e244-48c8-a71c-b86050fb7704_Supplemental_files-_Study_manual,_Data_tools,_and_recruitment_,materials.zip!/Supplemental files- Study manual, Data tools, and recruitment ,materials/Phase II Data Extraction.pdf]

HMHB Data Extraction Form

PARTICIPANT STUDY ID ______________________

Date of data extraction ______________________

Data Source
- ◯ Mother baby booklet
- ◯ Child health card
- ◯ Individual facility based records
- ◯ Facility register

SECTION 1: VACCINES

Record total number doses of each:

1. BCG ______________________

2. Polio/OPV ______________________

3. DPT ______________________

4. HBV ______________________

5. Measles ______________________

6. Yellow Fever ______________________

7. Vitamin A ______________________

8. Deworming ______________________

9. Maternal TT ______________________

10. PENTA ______________________

SECTION 2: GROWTH MONITORING

1. Total Number of clinic visits between birth and 1 year of age where the child's growth was monitored. (This includes weight, height/length, and/or MUAC)	______________________
VISIT 1	
Age of child	______________________<br>(Age in MONTHS)
Indicate what was measured at each visit	☐ Weight<br>☐ Length/Height<br>☐ MUAC<br>☐ No growth monitoring data available
Weight	______________________<br>(in KGs)
Length/Height	______________________<br>(Length/Height in CM)
MUAC	______________________<br>(MUAC in CM)
Is there another growth monitoring visit to record?	◯ Yes<br>◯ No
VISIT 2	
Age of child	______________________<br>(Age in MONTHS)
Indicate what was measured at each visit	☐ Weight<br>☐ Length/Height<br>☐ MUAC<br>☐ No growth monitoring data available
Weight	______________________<br>(in KGs)
Length/Height	______________________<br>(Length/Height in CM)
MUAC	______________________<br>(MUAC in CM)
Is there another growth monitoring visit to record?	◯ Yes<br>◯ No

VISIT 3

Age of child

____
(Age in MONTHS)

Indicate what was measured at each visit

- ☐ Weight
- ☐ Length/Height
- ☐ MUAC
- ☐ No growth monitoring data available

Weight

____
(in KGs)

Length/Height

____
(Length/Height in CM)

MUAC

____
(MUAC in CM)

Is there another growth monitoring visit to record?

- ◯ Yes
- ◯ No

VISIT 4

Age of child

____
(Age in MONTHS)

Indicate what was measured at each visit

- ☐ Weight
- ☐ Length/Height
- ☐ MUAC
- ☐ No growth monitoring data available

Weight

____
(in KGs)

Length/Height

____
(Length/Height in CM)

MUAC

____
(MUAC in CM)

Is there another growth monitoring visit to record?

- ◯ Yes
- ◯ No

VISIT 5

Age of child	______________________ (Age in MONTHS)
Indicate what was measured at each visit	☐ Weight<br>☐ Length/Height<br>☐ MUAC<br>☐ No growth monitoring data available
Weight	______________________ (in KGs)
Length/Height	______________________ (Length/Height in CM)
MUAC	______________________ (MUAC in CM)
Is there another growth monitoring visit to record?	◯ Yes<br>◯ No
VISIT 6	
Age of child	______________________ (Age in MONTHS)
Indicate what was measured at each visit	☐ Weight<br>☐ Length/Height<br>☐ MUAC<br>☐ No growth monitoring data available
Weight	______________________ (in KGs)
Length/Height	______________________ (Length/Height in CM)
MUAC	______________________ (MUAC in CM)
Is there another growth monitoring visit to record?	◯ Yes<br>◯ No
VISIT 7	
Age of child	______________________ (Age in MONTHS)

Indicate what was measured at each visit	☐ Weight<br>☐ Length/Height<br>☐ MUAC<br>☐ No growth monitoring data available
Weight	______________________<br>(in KGs)
Length/Height	______________________<br>(Length/Height in CM)
MUAC	______________________<br>(MUAC in CM)
Is there another growth monitoring visit to record?	◯ Yes<br>◯ No
VISIT 8	
Age of child	______________________<br>(Age in MONTHS)
Indicate what was measured at each visit	☐ Weight<br>☐ Length/Height<br>☐ MUAC<br>☐ No growth monitoring data available
Weight	______________________<br>(in KGs)
Length/Height	______________________<br>(Length/Height in CM)
MUAC	______________________<br>(MUAC in CM)
Is there another growth monitoring visit to record?	◯ Yes<br>◯ No
VISIT 9	
Age of child	______________________<br>(Age in MONTHS)
Indicate what was measured at each visit	☐ Weight<br>☐ Length/Height<br>☐ MUAC<br>☐ No growth monitoring data available

Weight	_______________ (in KGs)
Length/Height	_______________ (Length/Height in CM)
MUAC	_______________ (MUAC in CM)
Is there another growth monitoring visit to record?	◯ Yes<br>◯ No
VISIT 10	
Age of child	_______________ (Age in MONTHS)
Indicate what was measured at each visit	☐ Weight<br>☐ Length/Height<br>☐ MUAC<br>☐ No growth monitoring data available
Weight	_______________ (in KGs)
Length/Height	_______________ (Length/Height in CM)
MUAC	_______________ (MUAC in CM)
3. Was malnutrition ever diagnosed or included in a clinical problem list during any visit?:	◯ Yes<br>◯ No
