# Supplementary material for: Group versus individual antenatal and first year postpartum care: Study protocol for a multi-country cluster randomized controlled trial in Kenya and Nigeria
Source: Gates Open Res. 2019 Jul 4;2:56. Originally published 2018 Nov 5. [Version 2] doi: 10.12688/gatesopenres.12867.2 (PMC6350506; doi:10.12688/gatesopenres.12867.2)
Supplement: Supplementary file 1 [file gatesopenres-2-14160-s0000.tgz › 72242879-e244-48c8-a71c-b86050fb7704_Supplemental_files-_Study_manual,_Data_tools,_and_recruitment_,materials.zip!/Supplemental files- Study manual, Data tools, and recruitment ,materials/Phase II Survey 1 yr PP_women.pdf]

PNC Survey

PARTICIPANT STUDY ID	__________________________________
First and Last Name of data collector	__________________________________
HMHB PNC Register: Data Extraction	
PNC Attendance	☐ 1<br>☐ 2<br>☐ 3<br>☐ 4
Total PNC	__________________________________
Baby slept uder bed net last night	☐ 1<br>☐ 2<br>☐ 3<br>☐ 4
Total nights under net	__________________________________
1 - Would you like to delay another pregnancy?	◯ Yes<br>◯ No
1 - Currently using FP method	◯ Yes<br>◯ No
1 - Specify FP method	◯ LAM/breastfeeding<br>◯ Standard days or rhythm method (Calendar/rhythm/cycle beads/natural/Billing's method)<br>◯ Withdrawal<br>◯ Condoms<br>◯ Pill/Oral contraceptives<br>◯ Injectable<br>◯ Implants<br>◯ IUD<br>◯ Emergency contraception<br>◯ Sterilization<br>◯ Other
Other specify	__________________________________
2 - Would you like to delay another pregnancy?	◯ Yes<br>◯ No
2 - Currently using FP method	◯ Yes<br>◯ No

2 - Specify FP method	◯ LAM/breastfeeding<br>◯ Standard days or rhythm method (Calendar/rhythm/cycle beads/natural/Billing's method)<br>◯ Withdrawal<br>◯ Condoms<br>◯ Pill/Oral contraceptives<br>◯ Injectable<br>◯ Implants<br>◯ IUD<br>◯ Emergency contraception<br>◯ Sterilization<br>◯ Other
Other specify	__________________________
3 - Would you like to delay another pregnancy?	◯ Yes<br>◯ No
3 - Currently using FP method	◯ Yes<br>◯ No
3 - Specify FP method	◯ LAM/breastfeeding<br>◯ Standard days or rhythm method (Calendar/rhythm/cycle beads/natural/Billing's method)<br>◯ Withdrawal<br>◯ Condoms<br>◯ Pill/Oral contraceptives<br>◯ Injectable<br>◯ Implants<br>◯ IUD<br>◯ Emergency contraception<br>◯ Sterilization<br>◯ Other
Other specify	__________________________
4 - Would you like to delay another pregnancy?	◯ Yes<br>◯ No
4 - Currently using FP method	◯ Yes<br>◯ No
4 - Specify FP method	◯ LAM/breastfeeding<br>◯ Standard days or rhythm method (Calendar/rhythm/cycle beads/natural/Billing's method)<br>◯ Withdrawal<br>◯ Condoms<br>◯ Pill/Oral contraceptives<br>◯ Injectable<br>◯ Implants<br>◯ IUD<br>◯ Emergency contraception<br>◯ Sterilization<br>◯ Other

Other specify	__________________________________
GROUP ANC PHASE II: PNC Survey	
How many attempts were made by phone to reach this participant	__________________________________<br>(ENTER A NUMBER e.g. 1)
Physically traced	◯ Yes<br>◯ No
Outcome of tracing	◯ Located subject<br>◯ Did not locate subject
Were additional strategies used to trace this participant?	◯ Yes<br>◯ No
Describe the additional strategies	____
Is this person lost to follow up?	◯ Yes<br>◯ No
Why did you classify the participant as lost to follow up? Please add any additional information you may have uncovered related to women who were lost to follow up, such as whether they moved away; died; or withdrew participation in study	____
Do you agree to participate	◯ Yes - Eh<br>◯ No - Aa
Date of data collection/interview	__________________________________
Location of the interview	◯ Home<br>◯ Facility<br>◯ Other
Other location	__________________________________
1. What day was your baby born (the one who is close to 1 year old now)?<br>Wacce rana kika haihu	__________________________________
Age of baby in months	__________________________________
1a. Where was your baby born?<br>A ina aka haifi jaririnki?	◯ Facility - Asibiti<br>◯ Home - Gida<br>◯ Transit/Other - Kan hanyar zuwa asibiti/wani

i. Specify facility name

____

ii. Was this the same facility where you received ANC?
Shin wannan makaman ne wanda aka samu ANC?

◯ Yes - Eh
◯ No - Aa

2. Is the baby still alive?
Yaron yana raye?

◯ Yes - Eh
◯ No - Aa

2(i). When did your baby die?
Yaushe yaron ya rasu?

____

Age of baby in months

____

2(ii). What do you think caused your baby to die?
Menene sanadiyyar rasuwarsa?

◯ Diarrheal disease - Gudawa/Kashi ruwa-ruwa
◯ Respiratory infection, including pneumonia or flu - Ciwon numfashi ko ciwon sanyi da mura
◯ Malaria - Zazzabin cizon sauro
◯ Other fever/infection - Wani dalili daban
◯ Congenital problem (problem they were born with) - Ciwon da aka haifeshi da shi
◯ Born too early/too small - Haihuwa kafin lokaci /Yaro karami too small
◯ Don't know - Ban sani ba
◯ Other - Wani daban

Other specify

____

2(iii). This survey has questions about your health and care and your baby's health and care before they died. Is it OK if we ask you these questions?
Wannan tambayoyi sun 'kunshi bayanai akan abin da ya shafi kulawa da lafiyarki da na yaronki kafin rasuwarsa. Babu damuwa mu ci gaba da tambayoyi?

◯ Yes - Eh
◯ No - Aa

3. What is (was) your baby's name?
Menene sunan yaron/yarinyarki?

____

CARE SEEKING AND PROVISION OF CARE

4. Since [pnc_q3]'s birth, how many Healthy Mother Healthy Baby meetings have you attended?
Bayan haihuwar [pnc_q3]'s, sau nawa kika halarci taron 'Uwa Lafiya, 'Da Lafiya?

◯ 0
◯ 1
◯ 2
◯ 3
◯ 4
(Specify 0, 1, 2, 3, 4)

4b. What was the biggest reason you did not attend any Healthy Mother Healthy Baby meetings?
Menene babban dalilin da ya hanaki halartar taron 'Uwa Lafiya, 'Da Lafiya?

- ☐ I did not know about them/didn't receive notification or phone calls - Bani da masaniya akai/ba wanda ya sanar da ni ko ya kira ni a wayar hannu
- ☐ I was not in the area/moved to a different area after baby was born - Bana nan/mun tashi bayan haihuwar yaron
- ☐ Husband or mother in law did not want me to attend - Mijina/sirikata bai/bata aminta in halarci taron ba
- ☐ It didn't sound appealing to me/I didn't want to go - Bani da sha'awar zuwa/Bana son zuwa taron
- ☐ It was too hard to get to the clinic - Wajen taron da wahalar zuwa
- ☐ Other - Wani Dalili

Other specify

____

5. I am going to read you a list of services. For each one, reply yes if you have received the service from a health care provider since [pnc_q3]'s birth, no if you have not, or not sure if you can't remember. These are services for you, not your baby
Zan karanto miki wasu tsare tsare. Ko wane daga cikinsu ina son ki bani amsar Eh indan kin amfana da shi daga ma'aikaciyar/ma'aikacin lafiya bayan haihuwar [pnc_q3]'s, Aa idan baki amfanaba, ko baza ki iya tunawaba. Ga tsare tsaren kamar haka, naki ko na yaron

5a. Family planning counseling
Bayani akan hutun lafiya

- ◯ Yes - Eh
- ◯ No - Aa

5b. A method of Family planning (for example, injectables, an implant, or oral contraceptives)
Hanyar Hutun haihuwa (Misali, Allurai, Dashen Hannu, or 'Kwayoyi na sha)

- ◯ Yes - Eh
- ◯ No - Aa

5c. Your blood pressure taken
Gwajin Hawan jini

- ◯ Yes - Eh
- ◯ No - Aa

5d. Any immunisations (for you ,not your baby)
Rigakafi (na ki, ba na yaro ba)

- ◯ Yes - Eh
- ◯ No - Aa

5e. Care related to depression or your mental health (that is, how you were thinking or feeling emotionally)
Larurar 'Kwa'kwalwa (Yadda kike tunani ko ji a tunaninki)

- ◯ Yes - Eh
- ◯ No - Aa

5f. Care for any other problem or concern with your health
Kulawa da wata matsala da ta shafi lafiyarki

- ◯ Yes - Eh
- ◯ No - Aa

6. Since [pnc_q3]'s birth, how many times have you taken [did you take] him/her to see a health care provider for
Tun haihuwar [pnc_q3], sau nawa kika kaishi/kaita warin ma'aikacin lafiya [An Kai] don matsalar

6. Not counting healthy mother, healthy baby meetings, since [pnc_q3]'s birth, how many times have you taken [did you take] him/her to see a health care provider for
Ban da taron 'Uwa Lafiya, 'Da Lafiya, Bayan haihuwa [pnc_q3], sau nawa kika kaishi/kaita [An Kai] wurin ma'aikacin lafiya domin

6a. Well-child care: that is, not because he/she was sick, but to receive immunizations, vitamin A, or growth monitoring? Include immunization days or campaigns<br>Kula da lafiyar yaro: ba don rashin lafiya ba, domin ya karbi rigakafi, vitamin A, ko kula da girman yaro? Harda ranakun rigakafi ko kulawa aka rigakafi	______________________
6b. A problem: that is, [pnc_q3] was sick or there was something worrying you about his/her health<br>Matsala: Wadda, [pnc_q3] yayi/tayi rashin lafiya ko akwai wata damuwa dangane da lafiyarsa/lafiyarta	______________________
7. Since birth, how many times has (did) [pnc_q3] experienced each of the following?<br>Bayan haihuwa Sau nawa [did] [pnc_q3] ya fuskanci daya daga cikin wadannan?	
7a. Diarrhea<br>Gudawa/Kashi mai ruwa-ruwa	______________________
7b. A fever<br>Zazzabi ko Mashashshara	______________________
7c. A cough<br>Tari	______________________
7d. Fast, short, rapid breaths or difficulty breathing, not caused by a blocked or runny nose<br>Hauhawar numfashi a dalilin toshewar hanci ko mura	______________________
8. Considering the last (most recent) time [pnc_q3] had diarrhea<br>La'akari da na 'karshe (mafi kusantuwa) lokaci [pnc_q3] aka yi gudawa	
8a. I would like to know how much [pnc_q3] was given to drink. This includes breastmilk, Oral Rehydration Salt solution (ORS) and other liquids given with medicine. During the last time [pnc_q3] had diarrhoea, was (he/she) given<br>Ina bukatar in san ko [pnc_q3] abin da aka bashi/bata ta/ya sha. Wannan ya hada da shayarwa, Ruwan Gishiri da Sikari (ruwan gishiri) da wani maganin da ake hadawa da ruwa. A lokaci na karshe [pnc_q3] yayi gudawa, aka ba(shi/ta)	◯ Less than usual to drink - Kasa da yadda aka saba bayarwa<br>◯ About the same amount - Kamar yadda aka saba bayarwa<br>◯ More than usual - Fiye da yadda aka saba bayarwa<br>◯ Don't know/Don't remember - Ban sani ba/B azan iya tunawa ba
8b. Was either home-made or store bought oral rehydration salt solution (ORS) given?<br>Ruwan gishiri da sukarin da aka bayar hadawa aka yi a gida ko kuma saye aka yi?	◯ Yes - Eh<br>◯ No - Aa<br>◯ Don't know/Don't remember - Ban sani ba/Na manta
8c. Were zinc tablets given?<br>An bada zinc?	◯ Yes - Eh<br>◯ No - Aa<br>◯ Don't know/Don't remember - Ban sani ba/Na manta

8d. During the time [pnc_q3] had diarrhea, was (he/she) given<br>Yayin da [pnc_q3] tayi/yayi gudawa, an ba(shi/ta)	◯ Less than usual to eat - Kasa da yadda aka saba bayarwa<br>◯ About the same amount to eat - Yadda aka saba bayarwa<br>◯ More than usual to eat - Sama da yadda aka saba bayarwa<br>◯ Nothing to eat - Babu abinda za'a ci<br>◯ Don't know/Don't remember - Ban sani ba/Na manta
8e. Did you seek any advice or treatment from a health center, clinic or hospital<br>An nemi shawara ko magani daga asibiti or 'dakin shan magani?	◯ Yes - Eh<br>◯ No - Aa<br>◯ Don't know/Don't remember - Ban sani ba/Na manta
9. Considering the last (most recent) time [pnc_q3] had a fever<br>Lokaci [pnc_q3] aka yi zazzabi	
9a. At any time when your child had a fever, did they have blood taken from his/her finger or heel for testing<br>A duk lokacin da danki/'yarki yake/take yin zazzabi, akan yi masa/mata gwajin jini wanda aka dauka ta 'danyatsar hannu ko na 'kafa?	◯ Yes - Eh<br>◯ No - Aa<br>◯ Don't know/Don't remember - Ban sani ba/Na manta
9b. Did you seek any advice or treatment from a health centre, clinic or hospital<br>An nemi shawara ko magani daga asibiti or 'dakin shan magani?	◯ Yes - Eh<br>◯ No - Aa
10. The last time [pnc_q3] had a cough or difficulty breathing, did you seek any advice or treatment from a health centre, clinic or hospital<br>Da [pnc_q3] tayi/yayi tari ko hauhawan numfashi, An nemi shawara ko magani daga asibiti or 'dakin shan magani?	◯ Yes - Eh<br>◯ No - Aa
11. To the best of your recollection, how many times has (did) [pnc_q3] receive each of the following<br>A iya saninki, sau nawa [pnc_q3] tayi/yayi amfani da 'daya daga cikin wadannan?	
11a. Medication for malaria<br>Maganin zazzabi	__________________________________<br>(explain that 1 time = the entire course of medication for illness episode, not each dose)
11b. An antibiotic<br>Maganin Kwayoyin	__________________________________<br>(explain that 1 time = the entire course of medication for illness episode, not each dose)
12. Did you sleep under a bed net last night<br>An kwana cikin shinge/gidan sauro?	◯ Yes - Eh<br>◯ No - Aa
13. Did [pnc_q3] baby sleep under a bed net last night<br>Shin [pnc_q3] ta/ya kwana cikin shinge/gidan sauro jiya da daddare?	◯ Yes - Eh<br>◯ No - Aa

14. How old was [pnc_q3] when you last took an iron folate tablet (blood tonic)
Shekarun [pnc_q3] nawa lokacin da tasha 'kwayar iron folate (Maganin Karin jini)?

____
(Provide answer in WEEKS, if answer given in MONTHS, multiply by 4)

15. In the year since [pnc_q3] was born, has a health care provider talk with you about
A shekarar da aka haifi [pnc_q3], ma'aikatan lafi sun yi Magana da ke akan

15a. Danger signs or problems to watch for in your baby
Alamomin hatsari ko matsaloli da za'a lura da su a tattare da yaron/yarinyar

- ◯ Yes - Eh
- ◯ No - Aa
- ◯ Don't know/Don't remember - Ban sani ba/Na manta

15b. The importance of sleeping under a bed net
Mahimmancin kwana cikin shinge ko gidan sauro

- ◯ Yes - Eh
- ◯ No - Aa
- ◯ Don't know/Don't remember - Ban sani ba/Na manta

15c. The importance of continuing to take iron and folic acid tablets after delivery (blood tonic)
Mahimmancin shan maganin iron da folic acid bayan haihuwa (Maganin 'karin jini)

- ◯ Yes - Eh
- ◯ No - Aa
- ◯ Don't know/Don't remember - Ban sani ba/Na manta

15d. Why immunisations are important and/how immunisations protect your child
Menene yasa rigakafi take da mahimmanci kuma/ko yaya rigakafi ke bawa yaro/yarinya kariya?

- ◯ Yes - Eh
- ◯ No - Aa
- ◯ Don't know/Don't remember - Ban sani ba/Na manta

15e. Postpartum depression, that is if you don't take joy in anything or are feeling sad or tired all the time since [pnc_q3] was born
Kunci bayan haihuwa, kamar idan kina jin fushi ko rahin farin ciki tun daga haihuwa [pnc_q3]

- ◯ Yes - Eh
- ◯ No - Aa
- ◯ Don't know/Don't remember - Ban sani ba/Na manta

EARLY CHILDHOOD DEVELOPMENT

16. In the past 3 days, did you or any household member age 15 or over engage in any of the following activities with [pnc_q3]? If yes, who engaged in this activity with [pnc_q3]?
A 'kwana uku da suka wuce, kin yi ko wani daga cikin iyalanki mai shekaru 15 ko fiye da haka yi 'daya daga cikin wa'dannan abubuwan da [pnc_q3]? Idan Eh, wanene yayi wa'dannan abubuwan da [pnc_q3]?

16a. Read books or looked at picture books with [pnc_q3]
Karanta litattafai ko littafin kallon hoto tare da [pnc_q3]

- ☐ Mother - Uwa
- ☐ Father - Uba
- ☐ Other - Wasu
- ☐ No one - Ba kowa

Other specify

____

16b. Told stories to [pnc_q3]
Fadin labarai [pnc_q3]

- ☐ Mother - Uwa
- ☐ Father - Uba
- ☐ Other - Wasu
- ☐ No one - Ba kowa

Other specify

____

16c. Sang songs to or with [pnc_q3] including lullabies
Rera waka ko tare da [pnc_q3] hadda tatsuniya?

☐ Mother - Uwa
☐ Father - Uba
☐ Other - Wasu
☐ No one - Ba kowa

Other specify

____

16d. Played with [pnc_q3]
Wasa da [pnc_q3]

☐ Mother - Uwa
☐ Father - Uba
☐ Other - Wasu
☐ No one - Ba kowa

Other specify

____

16e. Named, counted, or drew things for or with [pnc_q3]
Sunaye, 'kirge, zanwa ko zane tare da [pnc_q3]?

☐ Mother - Uwa
☐ Father - Uba
☐ Other - Wasu
☐ No one - Ba kowa

Other specify

____

17. For the following statements,please rate each statement on a scale according to how true the statement is. (Show participant the visual analogue scale/visual aide and use to describe the response options).
Daga cikin wadannan bayanai, auna kowane 'daya bisa ma'aunin daidai. (Nunawa mai bada amsa hotunan kati/ko hoton dake bayyana ma'anar bayanan).

17a. In order to bring up, raise, or educate a child properly, the child needs to be physically punished
Domin rainon ko ilimantar da yaro/yarinya akwai bukatar huntarwa mai tsanani

◯ Not at all true - Duka ba daidai ba ne
◯ Slightly true - Kusan daidai
◯ Somewhat true - Kamar daidai
◯ Mostly true - Mafiya daidai
◯ Completely true - Duka daidai ne
(Follow the SOP for introducing the visual analog scale)

17b. There are things I can do to make my baby smarter/sharper/more intelligent
Akwai abubuwa da zanyi domin yarona/yarinyata ya/ta zama mai 'kokari/kaifin tunani/wayo

◯ Not at all true - Duka ba daidai ba ne
◯ Slightly true - Kusan daidai
◯ Somewhat true - Kamar daidai
◯ Mostly true - Mafiya daidai
◯ Completely true - Duka daidai ne
(Follow the SOP for introducing the visual analog scale)

17c. Showing my baby I love them will help them develop and be healthier
Nunawa 'ya'ya soyayya zai taimaka musu wajen girma cikin 'koshin lafiya

◯ Not at all true - Duka ba daidai ba ne
◯ Slightly true - Kusan daidai
◯ Somewhat true - Kamar daidai
◯ Mostly true - Mafiya daidai
◯ Completely true - Duka daidai ne
(Follow the SOP for introducing the visual analog scale)

17d. It is important for fathers to talk and play with their children<br>Yana da matu'kar mahimmanci ga Iyaye maza su na yin wasa da yiwa 'ya'yansu magana	◯ Not at all true - Duka ba daidai ba ne<br>◯ Slightly true - Kusan daidai<br>◯ Somewhat true - Kamar daidai<br>◯ Mostly true - Mafiya daidai<br>◯ Completely true - Duka daidai ne<br>(Follow the SOP for introducing the visual analog scale)
18. In the year since your baby's birth, has a health care provider talked with you about<br>A shekarar da aka haifi suna, ma'aikatan lafi sun yi magana da ke akan	
18a. The importance of talking with your baby<br>Mahimmancin Magana da 'ya'ya	◯ Yes - Eh<br>◯ No - Aa<br>◯ Don't know/Don't remember - Ban sani ba/Na manta
18b. The importance of playing with your baby<br>Mahimmancin wasa da 'ya'ya	◯ Yes - Eh<br>◯ No - Aa<br>◯ Don't know/Don't remember - Ban sani ba/Na manta
18c. What activities and behaviours you can expect your baby to be doing by age 1<br>Wanne irin halayya kike tsammanin ya nuna yayin ya ciki shekara 1	◯ Yes - Eh<br>◯ No - Aa<br>◯ Don't know/Don't remember - Ban sani ba/Na manta
18d. Ways you can teach your child right from wrong without hitting or yelling<br>Hanyoyin da za'a iya nunawa 'ya'ya daidai ko kuskure ba tare da duka ko hantara ba	◯ Yes - Eh<br>◯ No - Aa<br>◯ Don't know/Don't remember - Ban sani ba/Na manta

WASH AND SAFETY

19. Do you have a place to wash your hands in your home or yard<br>Ko kina da wajen wanke hannu a gidanki ko 'bangarenki?	◯ Yes - Eh<br>◯ No - Aa
19(i). May I see it<br>Na iya gani?	◯ Piped water/tap/sink observed - Ruwan famfo/famfo/wajen wanke-wanke aka gani<br>◯ Other fixed station that allows one person to wash hands independently observed - Wani killaceccan wuri na musamman dake bawa mutum daya damar wan hannu aka gani<br>◯ Mobile object observed (bucket/jug/pitcher/kettle) - Mazubin ruwa da akan iya mtsarwa aka gani (bokati/babban kofi/kwanan ruwa/Buta)<br>◯ No handwashing place observed - Babu abin wanke hannu da aka gani (Tsallake zuwa tambaya ta 2)
19(i). Describe	__________________________________
19(ii). Observe presence of water at the place for handwashing<br>Duba ko akwai ruwa a wajen wanke hannu	◯ Water is available - Akwai ruwa<br>◯ Water is not available - Babu ruwa

19(iii). Observe - is soap, detergent or ash/mud/sand present at the place for handwashing
Duba - akwai sabulu, toka/toka/rairayi a wurin da ake wanke hannu?

- ◯ Yes, soap/detergent present - Eh, akwai sabulu/garin sabulu
- ◯ Yes, ash/mud/sand present - Eh, akwai toka/'kasa/rairayi
- ◯ No, nothing present - Aa, babu komai

20. For the following statements,please rate each statement on a scale according to how true the statement is. (Show participant the visual analogue scale/visual aide and use to describe the response options).
Daga cikin wadannan bayanai, auna kowane 'daya bisa ma'aunin daidai. (Nunawa mai bada amsa hotunan kati/ko hoton dake bayyana ma'anar bayanan).

20a. Clean, safe water is always available in the house for drinking
Ko da yaushe akwai ruwa tsaftacacce mai lafiya domin asha

- ◯ Not at all true - Duka ba daidai ba ne
- ◯ Slightly true - Kusan daidai
- ◯ Somewhat true - Kamar daidai
- ◯ Mostly true - Mafiya daidai
- ◯ Completely true - Duka daidai ne

20b. [pnc_q3] has not been fed with a bottle or cup with a teat
[pnc_q3] ba a ciyar da kwalban ko kwalba ba tare da kara

- ◯ Not at all true - Duka ba daidai ba ne
- ◯ Slightly true - Kusan daidai
- ◯ Somewhat true - Kamar daidai
- ◯ Mostly true - Mafiya daidai
- ◯ Completely true - Duka daidai ne

20c. My yard is kept clean and free of chicken or other feces
Tsakar gidana a tsaftace yake kuma babu kashin kaji ko na dabbobi

- ◯ Not at all true - Duka ba daidai ba ne
- ◯ Slightly true - Kusan daidai
- ◯ Somewhat true - Kamar daidai
- ◯ Mostly true - Mafiya daidai
- ◯ Completely true - Duka daidai ne

20d. All animal and human feces, including baby's , and nappy water is disposed of in latrine, compost or trash bin
Ana zubar da dukkanin kashin dabbobi da na mutane, harda da na jariri ko ruwan wanke kasha da fitsari a bayan gida ko kwandon shara

- ◯ Not at all true - Duka ba daidai ba ne
- ◯ Slightly true - Kusan daidai
- ◯ Somewhat true - Kamar daidai
- ◯ Mostly true - Mafiya daidai
- ◯ Completely true - Duka daidai ne

20e. [pnc_q3] is successfully preventted from eating soil or chicken feces
[pnc_q3] a killace yake/take daga cin 'kasa ko kashin dabbobi

- ◯ Not at all true - Duka ba daidai ba ne
- ◯ Slightly true - Kusan daidai
- ◯ Somewhat true - Kamar daidai
- ◯ Mostly true - Mafiya daidai
- ◯ Completely true - Duka daidai ne

20f. [pnc_q3] is always supervised
[pnc_q3] ana kulawa da shi/ita

- ◯ Not at all true - Duka ba daidai ba ne
- ◯ Slightly true - Kusan daidai
- ◯ Somewhat true - Kamar daidai
- ◯ Mostly true - Mafiya daidai
- ◯ Completely true - Duka daidai ne

NUTRITION (MATERNAL AND INFANT, INCLUDING BF)

21. Is [pnc_q3] still breastfeeding
[pnc_q3] yana shan nono har yanzu?

- ◯ Yes - Eh
- ◯ No - Aa

21(i). At what age did you stop breastfeeding [pnc_q3]
A wacce shekar ne ki ka dakata shayar da [pnc_q3] ?

- ◯ Never breastfed - Ban taba shayar da nono ba
- ◯ Age in months - Shekaru a watanni
- ◯ Don't know/Don't remember - Ban sani ba/Ba zan iya tunawa ba

Age in months<br>Shekaru a watanni	________________________
22. How old was [pnc_q3] when he/she was first given<br>Shekarun [pnc_q3] nawa sanda aka bata/bashi na farko	
22a. A non-breastmilk liquid, such as water, formula, cow/goat milk, sugar water, tea, or fruit juice<br>Madara ta ruwa, kamar irin ruwa, madarar, nonon shanu ko akuya, ruwa sukari, shayi, ko lemon 'ya'yan itatuwa	◯ Months - Watanni<br>◯ Don't know/Don't remember - Ban sani ba/Ba zan iya tunawa ba<br>◯ Not given yet - Har yanzu ba'a bata<br>◯ Not given prior to death - Ba'a bata/bashi ba kafin ta/ya rasu
Months	________________________
22b. Thin porridge (from maize, rice etc.)<br>Fate (na masara, shinkafa, da sauransu)	◯ Months - Watanni<br>◯ Don't know/Don't remember - Ban sani ba/Ba zan iya tunawa ba<br>◯ Not given yet - Har yanzu ba'a bata<br>◯ Not given prior to death - Ba'a bata/bashi ba kafin ta/ya rasu
Months	________________________
22c. Fish or meat (chicken, beef, goat etc.)<br>Kifi ko Nama (kaza, Jan Nama, naman akuya, da suaransu)	◯ Months - Watanni<br>◯ Don't know/Don't remember - Ban sani ba/Ba zan iya tunawa ba<br>◯ Not given yet - Har yanzu ba'a bata<br>◯ Not given prior to death - Ba'a bata/bashi ba kafin ta/ya rasu
Months	________________________
22d. Sugar sweetened drinks like soda, juice or sweetened milk<br>Abin na zaki kamar soda, ruwan lemo, ko madara mai zaki	◯ Months - Watanni<br>◯ Don't know/Don't remember - Ban sani ba/Ba zan iya tunawa ba<br>◯ Not given yet - Har yanzu ba'a bata<br>◯ Not given prior to death - Ba'a bata/bashi ba kafin ta/ya rasu
Months	________________________
22e. Any food or drink other than breastmilk<br>Kowane irin abinci/abinsha banda ruwan nono	◯ Months - Watanni<br>◯ Don't know/Don't remember - Ban sani ba/Ba zan iya tunawa ba<br>◯ Not given yet - Har yanzu ba'a bata<br>◯ Not given prior to death - Ba'a bata/bashi ba kafin ta/ya rasu
Months	________________________

23. At what age should a baby first start to receive liquid or food other than breastmilk
A wane shekaru ne ya kamata yaron ya fara shan wani abu bayan nonon uwa?

____
(Months)

24. At what age should a baby first start to receive foods from animal sources, such as fish, meat, eggs or diary
A wane shekaru ne yaro zai fara cin abinci da aka samar daga dabbobikamar Kifi, Nama, lemukan madara?

____
(Months)

25. What are some reasons it is best not to give babies less than 6 months old anything but breastmilk?
Wadanne dalilaine suke hana bawa yara 'yan kasa da wata 6 ko wane irin abinci face ruwan nonon uwa?

- ☐ Breast milk provides the best nutrition/more likely to become malnourished if fed other drinks/foods - Ruwan nonon uwa na bawa yara ingattaccen abinci/sukan iya kamuwa da tamowa idan suka ci/sha wani abu
- ☐ Breast milk prevents infections like diarrhea, respiratory and ear infections/more likely to get sick if fed other drinks/foods - Ruwan nonon uwa na bada kariya daga cututtuka kamar gudawa, ciwon numfashi da kunne (har da mura, ciwon nimfashi)/ sukan iya kamuwa da tamowa idan suka ci/sha wani abu
- ☐ Before 6 months anything other than breast milk can damange baby's stomach - Wani nau'in abincin na iya 'lalata cikin yara 'yan 'kasa da wattanni shida
- ☐ Feeding other drinks/foods brefore 6 months can increase risk of HIV transmission - Cin abinci/abinsha kafin yaro ya cika wata 6 na cikin hadarin kamuwa da ciwo mai karya karkuwar jiki
- ☐ Feeding only breast milk will make baby smarter - Shan nono uwa kadai zai sa yaro basira
- ☐ Feeding only breast milk allows mother to use LAM as FP method - Shayar da nonon uwa ga yaro zalla zai taimakawa uwa wajen bada hutun haihuwa
- ☐ Other - Wani

(select as many as mentioned, probe "any other reasons we shouldn't give babies less than six months old food or drink other than breastmilk?"))

Other specify

____

26. If a mother thinks her baby is not getting enough breastmilk, what should she do?<br>Idan uwa tana tuaninin yaron ta baya samun isheshen ruwan, me zata yi?	☐ Breastfeed more often/more frequently - Shayar da nonon uwa mafi yawa/akai akai<br>☐ Breastfeed for a longer period at each feeding - Shayar da yaro nonon uwa mai tsawo duk lokacin da za'a shayar<br>☐ Empty each breast at each feeding/empty one breast before switching to the next - Karar da ruwan nonon uwa duk lokacin shayarwa/Karar da nonon 'bari guda kafin a shayar daga 'bari na biyu<br>☐ Mother needs to drink more water/fluids - Uwa ta nabukatar shan ruwa da yawa<br>☐ Mother needs to eat more food - Uwa tana bukatar ta ci abinci da yawa<br>☐ Give baby other liquids/foods - Abawa yaro wani abinci ko abin sha give<br>☐ Other - Wani<br>(do not read, prompt with "anything else she should do if she thinks her baby is not getting enough breastmilk?"))
Other specify	______________________
27. From the time [pnc_q3] woke up yesterday, to the time [pnc_q3] woke up today<br>Tun daga lokacin da [pnc_q3] ta/ya tashi jiya zuwa lokacin [pnc_q3] da ta/ya tashi yau	
27a. How many times did he/she breastfeed<br>Sau nawa aka shayar da ita/shi?	______________________<br>(If not BF, record 0)
27b. How many times did they eat solid, semi-solid, or soft foods, including snacks<br>Sau nawa suka ci abinci mai nauyi, dan nauyi-nauyi, ko mai laushi, har da chinchin?	______________________
27c. Did they eat any foods from an animal source, like meat, fish, eggs, or dairy<br>Sun ci wani abinci da aka samar daga dabbobi, kamar Nama, Kifi, Kwai ko Ruwa Madara?	◯ Yes - Eh<br>◯ No - Aa<br>◯ Not sure - Ban tabbatar ba
27d. Did they eat any grains, roots, or tubers<br>Sun ci wani abinci na tsaba, saiwu, ko doya?	◯ Yes - Eh<br>◯ No - Aa<br>◯ Not sure - Ban tabbatar ba
27e. Did they eat any legumes (beans, peas), seeds, nuts, or lentils<br>Sun ci wani abuncin wake, (wake), 'ya'ya itace, gwada?	◯ Yes - Eh<br>◯ No - Aa<br>◯ Not sure - Ban tabbatar ba
27f. Did they eat any fruits and vegetables<br>Sun ci 'ya'yan itatuwa ko kayan lambu?	◯ Yes - Eh<br>◯ No - Aa<br>◯ Not sure - Ban tabbatar ba
27g. Did they eat any sugary drinks or sweets<br>Sun ci wani abinsha mai zaki ko alawa?	◯ Yes - Eh<br>◯ No - Aa<br>◯ Not sure - Ban tabbatar ba
28. In the year since your baby's birth, has a health care provider talked with you about<br>Tun daga shekarar haihuwar yaron, wata malamar asibiti tayi miki Magana akan wadannan	

28a. How to use breastfeeding to prevent another pregnancy right away, a method called lactational amenorrhea method, or LAM<br>Yadda za kiyi amfani da shayarwa wajen jinkirta samun juna biyu kai tsaye, hanyar da ake kiranta Shayar da nonon uwa zalla, (LAM)	◯ Yes - Eh<br>◯ No - Aa<br>◯ Don't know/Don't remember - Ban sani ba/Na manta
28b. How to increase your milk supply if needed<br>Yadda za ki kara ruwan nononki idan kin bukaci hakan	◯ Yes - Eh<br>◯ No - Aa<br>◯ Don't know/Don't remember - Ban sani ba/Na manta
28c. Eating extra food while breastfeeding<br>Yawaita cin abinci yayin shayarwa	◯ Yes - Eh<br>◯ No - Aa<br>◯ Don't know/Don't remember - Ban sani ba/Na manta
28d. When to introduce fluids and foods other than breastmilk to your baby<br>Lokacin fara bawa yaro ruwa ko abinci bayan ruwan nonon uwa	◯ Yes - Eh<br>◯ No - Aa<br>◯ Don't know/Don't remember - Ban sani ba/Na manta
28e. Feeding your baby from their own, separate bowl/plate<br>Baiwa yaro abinci a kwanonsa daban/faranti	◯ Yes - Eh<br>◯ No - Aa<br>◯ Don't know/Don't remember - Ban sani ba/Na manta
28f. Feeding your baby a variety of foods everyday<br>Ciyar da yaro abinci kala-kala a kullum	◯ Yes - Eh<br>◯ No - Aa<br>◯ Don't know/Don't remember - Ban sani ba/Na manta
28g. Feeding your baby foods from animal sources<br>Ciyar da yaro abinci da aka samar daga dabbobi	◯ Yes - Eh<br>◯ No - Aa<br>◯ Don't know/Don't remember - Ban sani ba/Na manta
28h. Feeding your baby colorful fruits and vegetables<br><br>Ciyar da yaro 'ya'yan itatuwa na marmari ko ganyayyaki	◯ Yes - Eh<br>◯ No - Aa<br>◯ Don't know/Don't remember - Ban sani ba/Na manta
28i. Avoiding sugary drinks and sweets<br>Kauracewa abinsa mai za'ki da alawoyi	◯ Yes - Eh<br>◯ No - Aa<br>◯ Don't know/Don't remember - Ban sani ba/Na manta

INCREASED SELF-EFFICACY/EMPOWERMENT OF DELIVERED WOMEN

29. Who will usually make decisions about health care for yourself and your newborn<br>Wa nene yake yanke hukuncin kan abin da ya shafi lafiyarki da ta jaririn?	◯ You - Ke ce<br>◯ Your husband/partner - Mijinki/Abokin zama<br>◯ You and your husband/partner jointly - Tare da Ke da Mijinki/Abokin zama<br>◯ Someone else - Wani daban
Someone else, specify	__________________________
30. Would you say that using contraception is mainly<br><br>Kina jin amfani da Hanyoyin samar da hutun haihuwa	◯ Your decision - Ra'ayinki ne<br>◯ Your husband/partners decision - Ra'ayin Mijinki/Abokin zama<br>◯ Joint decision - Ra'ayinku tare<br>◯ Other - Wani

Other specify

____

31. Please rate how certain you are that you can do each of the things described by using this scale (Show participant the visual analogue scale/visual aide and use to describe the response options - 0-10 cannot do at all to highly certain can do)
Ina son ki auna tabbatar da wadannan bayanai ta yin amfani da ma'auni (Nunawa mai bada amsa hoton ma'auni/hotunan kwatance da ake yin amfani da su wajen ma'auni - 0-10 daga baza su iya ba zuwa za su iya sosai)

31a. Feed my baby in a way that helps prevent problems and keeps my baby healthy
Ciyar da yaro/yarinya ta hanyar taimaka masa/mata wajen kariya da zama cikin 'koshin lafiya

○ 0 ○ 1 ○ 2 ○ 3 ○ 4 ○ 5 ○ 6 ○ 7 ○ 8 ○ 9 ○ 10

31b. Help my baby's brain grow and stronger/smarter
Taimakawa kwakwalwar yaro/yarina ta girma cikin kaifi da kwazo

○ 0 ○ 1 ○ 2 ○ 3 ○ 4 ○ 5 ○ 6 ○ 7 ○ 8 ○ 9 ○ 10

31c. Prevent serious accidents from happening to my baby
Kare yarona/yarinyata daga mummunan ha'dari

○ 0 ○ 1 ○ 2 ○ 3 ○ 4 ○ 5 ○ 6 ○ 7 ○ 8 ○ 9 ○ 10

31d. Recognise a health problem with an infant or small child
Kokarin kane matsalolin lafiya na jariri ko yara

○ 0 ○ 1 ○ 2 ○ 3 ○ 4 ○ 5 ○ 6 ○ 7 ○ 8 ○ 9 ○ 10

31e. Take action to help an infant or small child with a health problem
Daukar matakin taimakawa yaro akan matsalar lafiya

○ 0 ○ 1 ○ 2 ○ 3 ○ 4 ○ 5 ○ 6 ○ 7 ○ 8 ○ 9 ○ 10

31f. Make good decisions related to the health of myself and my family
Yanke kyakykyawan hukunci akan abinda da ya shafi lafiyata da ta iyalina

○ 0 ○ 1 ○ 2 ○ 3 ○ 4 ○ 5 ○ 6 ○ 7 ○ 8 ○ 9 ○ 10

31g. Bring up difficult subjects with my husband
Tattauna al'amura masu tsauri da mijina

○ 0 ○ 1 ○ 2 ○ 3 ○ 4 ○ 5 ○ 6 ○ 7 ○ 8 ○ 9 ○ 10

31h. Bring up difficult subjects with my mother in law
Tattauna al'amura masu tsauri da uwar mijina

○ 0 ○ 1 ○ 2 ○ 3 ○ 4 ○ 5 ○ 6 ○ 7 ○ 8 ○ 9 ○ 10

31i. Share my knowledge and let others know what I really think, even if it is different from what they know or think
Tattauna ra'ayina tare da wasu mutane don sanar da su fahimta ta ko da ra'ayinsu ya bambanta da nawa

○ 0 ○ 1 ○ 2 ○ 3 ○ 4 ○ 5 ○ 6 ○ 7 ○ 8 ○ 9 ○ 10

31j. Ask a provider to explain something in a different way if I don't understand what they are telling me
Tambayar ma'aikacin lafiya don neman 'karin bayani aka abinda suke fada bangane ba

○ 0 ○ 1 ○ 2 ○ 3 ○ 4 ○ 5 ○ 6 ○ 7 ○ 8 ○ 9 ○ 10

31k. Make decisions for myself
Yanke wa kaina hukunci

○ 0 ○ 1 ○ 2 ○ 3 ○ 4 ○ 5 ○ 6 ○ 7 ○ 8 ○ 9 ○ 10

32. For these statements, regarding your husband or current partner, please rate each statement on a scale according to how true the statement is. (Show participant the visual analogue scale/visual aide and use to describe the response options).
Daga cikin wadannan bayanan dangance da mijinki/abokin zamanki na yanzu, auna ko wanne bayani akan ma'aunin yanda yayi miki daidai. (Nunawa mai bada amsa hoton ma'auni/hotunan kwatance da ake yin amfani da su wajen ma'auni)

32a. [pnc_q3]'s father helps(ed) to take care of him/her, such as by bathing/feeding/playing (not just providing money)<br>Baban [pnc_q3]'s yana taimaka wajen kula da ita/shi, kamar su wanka/ciyarwa/wasa (ba bukatar kudi kadai ba)	○ Not at all true - Duka ba daidai ba ne<br>○ Slightly true - Kusan daidai<br>○ Somewhat true - Kamar daidai<br>○ Mostly true - Mafiya daidai<br>○ Completely true - Duka daidai ne
32b. He supports(ed) you with breastfeeding<br>Yana taimaka mini wajen shayar da ita/shi nono	○ Not at all true - Duka ba daidai ba ne<br>○ Slightly true - Kusan daidai<br>○ Somewhat true - Kamar daidai<br>○ Mostly true - Mafiya daidai<br>○ Completely true - Duka daidai ne
32c. He let you rest while you were pregnant and when [pnc_q3] was small<br>Ya barki ki huta lokacin da kike da juna biyu da sanda [pnc_q3] take/yake karama	○ Not at all true - Duka ba daidai ba ne<br>○ Slightly true - Kusan daidai<br>○ Somewhat true - Kamar daidai<br>○ Mostly true - Mafiya daidai<br>○ Completely true - Duka daidai ne
32d. He helped you/allowed you extra food while pregnant and nursing<br>Ya taimaka miki/yabarki ki ci abinci sosai lokacin da kike da juna biyu da shayarwa	○ Not at all true - Duka ba daidai ba ne<br>○ Slightly true - Kusan daidai<br>○ Somewhat true - Kamar daidai<br>○ Mostly true - Mafiya daidai<br>○ Completely true - Duka daidai ne

33. How much have you discussed each of the following subjects with your husband/partner in the last year since your baby was born? (Show participant the visual analogue scale/visual aide and use to describe the response options).
Kamar sau nawa kika tattauna akan ko wanne daya daga cikin wadannan bayanai tare da mijinki/abonkin zamanki a shekarar da ta wuce tun bayan haihuwar 'danki? (Nunawa mai bada amsa hoton ma'auni/hotunan kwatance da ake yin amfani da su wajen ma'auni).

33a. How to help our baby grow smarter/sharper/more intelligent<br>Yadda zan taimaki yarona/yarinyata don ya girma cikin kokari/basira kuma mai kyakykyawan tunani	○ None - Babu<br>○ A little - Dan Kadan<br>○ Some - Kadan<br>○ Quite a bit - Dan kankani<br>○ A lot - Da yawa

33b. How to help our baby stay healthy and well nourished<br>Yadda zan taimaki jaririn mu don ya kasance cikin koshin lafiya da walwala	◯ None - Babu<br>◯ A little - Dan Kadan<br>◯ Some - Kadan<br>◯ Quite a bit - Dan kankani<br>◯ A lot - Da yawa
33c. How to keep our baby safe and prevent accidents<br><br>Tadda zan zaunar da yaronmu cikin tsaro da kare shi daga daharuka	◯ None - Babu<br>◯ A little - Dan Kadan<br>◯ Some - Kadan<br>◯ Quite a bit - Dan kankani<br>◯ A lot - Da yawa
33d. What family size we want<br>Irin yawan iyalin da muke so	◯ None - Babu<br>◯ A little - Dan Kadan<br>◯ Some - Kadan<br>◯ Quite a bit - Dan kankani<br>◯ A lot - Da yawa
33e. If and when we want to have another baby<br>Idan muna so ko lokacin da muke son haihuwar wani 'dan	◯ None - Babu<br>◯ A little - Dan Kadan<br>◯ Some - Kadan<br>◯ Quite a bit - Dan kankani<br>◯ A lot - Da yawa
33f. What family planning method we'd like to use (including none)<br>Irin Hanyar hutun haihuwar da muke so muyi amfani dashi (Harda babu)	◯ None - Babu<br>◯ A little - Dan Kadan<br>◯ Some - Kadan<br>◯ Quite a bit - Dan kankani<br>◯ A lot - Da yawa

34. For these statements, thinking of your husband/current partner, please rate each statement on a scale according to how true the statement is. (Show participant the visual analogue scale/visual aide and use to describe the response options).
A ko wane daya daga cikin wadannan bayanai, bisa la'akari da mijinki/abokin zamanki, auna ko wanne bayani akan ma'aunin yanda yayi miki daidai. (Nunawa mai bada amsa hoton ma'auni/hotunan kwatance da ake yin amfani da su wajen ma'auni).

34a. We agree on what size family we want<br>Mun amince akan yawan iyalin da muke so	◯ Not at all true - Duka ba daidai ba ne<br>◯ Slightly true - Kusan daidai<br>◯ Somewhat true - Kamar daidai<br>◯ Mostly true - Mafiya daidai<br>◯ Completely true - Duka daidai ne
34b. We agree on if and when we want to have another baby<br>Mun amince idan muna so ko yaushe muke son samun wani 'dan	◯ Not at all true - Duka ba daidai ba ne<br>◯ Slightly true - Kusan daidai<br>◯ Somewhat true - Kamar daidai<br>◯ Mostly true - Mafiya daidai<br>◯ Completely true - Duka daidai ne
34c. We agree on what form(s) of family planning we'd like to use (including none)<br>Mun amince akan wacce irin hanyar samun hutun haihuwa muke so muyi amfani da ita (Har da babu)	◯ Not at all true - Duka ba daidai ba ne<br>◯ Slightly true - Kusan daidai<br>◯ Somewhat true - Kamar daidai<br>◯ Mostly true - Mafiya daidai<br>◯ Completely true - Duka daidai ne

INCREASED # OF CLIENTS REPORTING SATISFACTION WITH SERVICES PROVIDED IN THE FIRST YEAR POSTNATALLY

35. Thinking about care you have received from health care providers since [pnc_q35] was born, please rate each statement on a scale according to how true the statement is. (Show participant the visual analogue scale/visual aide and use to describe the response options).
La'akari da kulawar da kika samu tun lokacin da kika haifi (name), auna ko wanne bayani akan ma'aunin yanda yayi miki daidai. (Nunawa mai bada amsa hoton ma'auni/hotunan kwatance da ake yin amfani da su wajen ma'auni).

35a. I was able to ask questions and they were answered in a way I understood<br>Nayi tambayoyi kuma an bani amsa yadda zan fahimta	◯ Not at all true - Duka ba daidai ba ne<br>◯ Slightly true - Kusan daidai<br>◯ Somewhat true - Kamar daidai<br>◯ Mostly true - Mafiya daidai<br>◯ Completely true - Duka daidai ne
35b. I learned important information about how to keep myself and my baby healthy<br>Na koyi mahimman abubuwa akan yadda zan kula da kaina da 'da na	◯ Not at all true - Duka ba daidai ba ne<br>◯ Slightly true - Kusan daidai<br>◯ Somewhat true - Kamar daidai<br>◯ Mostly true - Mafiya daidai<br>◯ Completely true - Duka daidai ne
35c. I never left visits with questions or confused about some things<br>Lokacin ziyarata, ban taba lafiya da wani abun da ban gane shi ba	◯ Not at all true - Duka ba daidai ba ne<br>◯ Slightly true - Kusan daidai<br>◯ Somewhat true - Kamar daidai<br>◯ Mostly true - Mafiya daidai<br>◯ Completely true - Duka daidai ne
35d. The provider always showed me respect and acted in respectful ways<br>Malaman lafiya sun girmamani kuma su saurare ni cikin girmamawa	◯ Not at all true - Duka ba daidai ba ne<br>◯ Slightly true - Kusan daidai<br>◯ Somewhat true - Kamar daidai<br>◯ Mostly true - Mafiya daidai<br>◯ Completely true - Duka daidai ne
35e. If I have another child I would be happy to receive care similar to what I received during this last year<br>Idan na samu wani yaron, zanyi matukar farin cikin samun kulawa irin wadda na samu a cikin shekarar baya.	◯ Not at all true - Duka ba daidai ba ne<br>◯ Slightly true - Kusan daidai<br>◯ Somewhat true - Kamar daidai<br>◯ Mostly true - Mafiya daidai<br>◯ Completely true - Duka daidai ne
35f. I would recommend to a friend to go to the same place for care for their child and/or themselves<br>Zan iya bawa wasu shawarar zuwa wurin domin kulawa da yaron ko dukkansu	◯ Not at all true - Duka ba daidai ba ne<br>◯ Slightly true - Kusan daidai<br>◯ Somewhat true - Kamar daidai<br>◯ Mostly true - Mafiya daidai<br>◯ Completely true - Duka daidai ne
35g. I always had enough time with the provider<br>Ina samun lokaci ishashshe tare da ma'iakaciyar lafiya	◯ Not at all true - Duka ba daidai ba ne<br>◯ Slightly true - Kusan daidai<br>◯ Somewhat true - Kamar daidai<br>◯ Mostly true - Mafiya daidai<br>◯ Completely true - Duka daidai ne
35h. I am very satisfied with the care my baby and I have received since birth<br>Na gamsu sosai da kulawar da aka bawa yaro/yarinya da ni kaina tun daka haihuwarsa	◯ Not at all true - Duka ba daidai ba ne<br>◯ Slightly true - Kusan daidai<br>◯ Somewhat true - Kamar daidai<br>◯ Mostly true - Mafiya daidai<br>◯ Completely true - Duka daidai ne

36. Imagine you were to have another baby. If you could choose, would you prefer individual one on one care in the year after birth or would you prefer group visits where you and your baby receive your care with other women who have babies the same age?<br><br>A misalin zaki sami wani yaron. Idan zaki zaba, za ki zabi kulawa ta daya bayan daya, a shekarar da kika haihu ko zuwa cikin taron matan da suka haihu da yaransu masu shekarun irin na 'danki/'yarki?	◯ Individual care - Kulawa daya bayan daya<br>◯ Group care - Kulawa cikin taro<br>◯ Unsure; doesn't matter which one; no preference - Ba tabbas; Koma wanne ne; bani da zabi

37. Thinking about the care you received in a group since your baby was born (healthy mother, healthy baby meetings), please rate each statement on a scale according to how true the statement is. (Show participant the visual analogue scale/visual aide and use to describe the response options).
La'akari da kulawar da kika samu tare da wasu matan tun daga lokacin da kika haifi yaronki (Taron Uwa Lafiya, 'Da Lafiya), auna ko wanne bayani akan ma'aunin yanda yayi miki daidai. (Nunawa mai bada amsa hoton ma'auni/hotunan kwatance da ake yin amfani da su wajen ma'auni).

37a. I was comfortable receiving care in a group<br>Hankalina kwance na karbi kulawar cikin taron mata	◯ Not at all true - Duka ba daidai ba ne<br>◯ Slightly true - Kusan daidai<br>◯ Somewhat true - Kamar daidai<br>◯ Mostly true - Mafiya daidai<br>◯ Completely true - Duka daidai ne
37b. I felt like I was part of the group<br>Sai na ji dama kamar tare muke da sauran taron matan	◯ Not at all true - Duka ba daidai ba ne<br>◯ Slightly true - Kusan daidai<br>◯ Somewhat true - Kamar daidai<br>◯ Mostly true - Mafiya daidai<br>◯ Completely true - Duka daidai ne
37c. I was comfortable talking and sharing thoughts with the group<br>Hankalina kwance nake Magana da misayar sani cikin taron matan	◯ Not at all true - Duka ba daidai ba ne<br>◯ Slightly true - Kusan daidai<br>◯ Somewhat true - Kamar daidai<br>◯ Mostly true - Mafiya daidai<br>◯ Completely true - Duka daidai ne
37d. I was comfortable asking questions in the group<br><br>Hankalina kwance nake tambaya a cikin taron matan	◯ Not at all true - Duka ba daidai ba ne<br>◯ Slightly true - Kusan daidai<br>◯ Somewhat true - Kamar daidai<br>◯ Mostly true - Mafiya daidai<br>◯ Completely true - Duka daidai ne
37e. I learned a lot from other women in the group<br>Na koyi abubuwa da yawa daga wurin taron matan	◯ Not at all true - Duka ba daidai ba ne<br>◯ Slightly true - Kusan daidai<br>◯ Somewhat true - Kamar daidai<br>◯ Mostly true - Mafiya daidai<br>◯ Completely true - Duka daidai ne
37f. Participating in the group changed the way I think about parenting<br>Kasancewa cikin taron mata ya canja min tunanina akan kulawar iyaye	◯ Not at all true - Duka ba daidai ba ne<br>◯ Slightly true - Kusan daidai<br>◯ Somewhat true - Kamar daidai<br>◯ Mostly true - Mafiya daidai<br>◯ Completely true - Duka daidai ne
37g. Participating in the group changed the way I think about family planning<br>Kasancewa cikin taron mata ya canja min tunanina akan hutun haihuwa	◯ Not at all true - Duka ba daidai ba ne<br>◯ Slightly true - Kusan daidai<br>◯ Somewhat true - Kamar daidai<br>◯ Mostly true - Mafiya daidai<br>◯ Completely true - Duka daidai ne

37h. Being part of the group helped make parenting less stressful<br>Kasancewa cikin taron mata ya taimaka min wajen rage damuwa	◯ Not at all true - Duka ba daidai ba ne<br>◯ Slightly true - Kusan daidai<br>◯ Somewhat true - Kamar daidai<br>◯ Mostly true - Mafiya daidai<br>◯ Completely true - Duka daidai ne

INCREASED SOCIAL CAPITAL/SOCIAL CONNECTIONS

38. Did you ever phone or send a text message to your provider
Shin kin taba' kiran waya ko aika sako ta wayar hannu zuwa ga ma'aikaciyar lafiyar da take baki kulawa

38a. During pregnancy<br>Lokacin Juna biyu?	◯ Yes - Eh<br>◯ No - Aa
38b. After pregnancy<br>Bayan haihuwa?	◯ Yes - Eh<br>◯ No - Aa
38c. If yes to either, did you phone or send a text message, or both<br>Idan Eh a daya daga ciki, kin buga waya ko aika sako ko duka?	◯ Phone - Buga waya<br>◯ Text - Aika sako<br>◯ Both - Duka biyun
39. Did you ever share a ride with another pregnant woman you knew to an ANC visit or to the clinic in the year since your baby was born?<br>Kin taba hada tafiya da wata mai juna biyu da kika sani zuwa awon juna biyu ko zuwa asibiti tun shekarar haihuwar yaronki/yarinyarki?	◯ Yes - Eh<br>◯ No - Aa
40. After being enrolled in this study, did you ever attend any group ANC or Healthy Mother Healthy Baby meetings at the facility?<br>Tun lokacin da aka zabe ki cikin irin wannan tattaunawa ta tattara bayanai da muke yi da ke, kin taba halartar tattaunawa cikin taro na masu juna biyu ko taron "Uwa Lafiya, Da Lafiya" a asibiti?	◯ Yes - Eh<br>◯ No - Aa
41. Did that group have a name<br>Idan Eh, taron naku yana da suna	◯ Yes, has a name - Eh, Yana da suna<br>◯ No, does not have a name - Aa, bashi da suna
Group name	__________________________
42. Either when you were pregnant or in the past year, how often did you communicate with women in your group outside of meeting time?<br>Ko lokacin da kike da juna biyu ko cikin shekarar da ta wuce, kamar sau nawa kika yi Magana da wasu daga cikin abokan taron ba cikin taron ba?	◯ Never - Bantaba ba<br>◯ Rarely/not very often - Ba sosai ba/Ba da yawa ba<br>◯ Sometimes/occasionally - Wane lokaci/lokaci-lokaci<br>◯ Regularly/on a monthly basis - Akai aki / a duk wata<br>◯ Regularly/on a weekly basis - Akai akai / duk sati ko mako<br>◯ More frequently/almost daily - Sau da yawa/Kusan kullum<br>(Read all and select one)

43. Which of the following did you use to communicate with other women in your group?<br>Wanne daga cikin wadannan kike amfani dashi wajen sadarwa?	☐ Phone - Waya<br>☐ Text - Tura sako<br>☐ WhatsApp - Hanyar Sada da zumunci ta "WhatsApp"<br>☐ Another social media app - Wata hanyar sada zumunci ta daban<br>☐ Social gatherings - Taron wayar da kai<br>☐ Any other ways - Akwai wata hanyar<br>(Read and select all that apply)
Any other way	__________________________________
44. Which of the following things did you communicate about<br>Wanne daga cikin wadannan abubuwa kuka tattauna ta hanyar sadarwa?	☐ Upcoming meetings, for example to remind each other of the meeting, see if they were planning to attend, or set up a shared ride - Taro mai zuwa, A misali don tutarta da juna, ki tabbatar ko sauran suna kokarin hada taron ko shirya hada tafiya wuri daya/guda<br>☐ A problem you or others in the group were having - Matsalar da ke ko wani acikin abonka taron yake fama da ita<br>☐ Help that you or others in the group needed - Wani taimako da ke ko wata daga cikin abokan taron yake da bukata<br>☐ Advice/information related to pregnancy - Shawara/Bayanai da suka danganci juna biyu<br>☐ Advice/information related to family planning - Shawara/Bayanai da suka danganci hutun haihuwa<br>☐ Advice/information related to parenting - Shawara/Bayanai da suka danganci rainon 'ya'ya<br>☐ Advice/information related to infant/child care practices or health - Shawara/Bayanai da suka danganci al'amuran jariri ko kulawa da yaro ko lafiyarsa<br>☐ Other - Wani<br>(Read and select all that apply)
Other specify	__________________________________
45. Do you think a woman would reach out to other members of the group if she had a problem or needed help?<br>Kina ganin wata mace zata iya ganawa da ma'abota taron idan tana bukar wani taimako ko tana da matsala?	◯ Yes - Eh<br>◯ No - Aa<br>◯ Unsure - Ban tabbatar ba
45a. If yes, do you know of specific examples when this has happened<br>Idan Eh, kina misalin na hakika wanda irin hakan ya faru?	◯ Yes - Eh<br>◯ No - Aa<br>◯ Unsure - Ban tabbatar ba

POSTPARTUM FAMILY PLANNING

46. I'm going to read some statements related to family planning (birth spacing). Please rate each statement on a scale according to how true the statement is. (Show participant the visual analogue scale/visual aide and use to describe the response options).
Zan karanto wasu bayanai da suka shafi/danganci Hutun haihuwa (hutun Haihuwa). Auna ko wanne bayani akan ma'aunin yanda yayi miki daidai. (Nunawa mai bada amsa hoton ma'auni/hotunan kwatance da ake yin amfani da su wajen ma'auni).

46a. I have heard people say things about family planning methods being harmful or dangerous<br>Na kan ji mutane suna fadin wasu abubuwa akan hanyoyin hutun haihuwa yana da hadari da kuma yana cutarwa.	○ Not at all true - Duka ba daidai ba ne<br>○ Slightly true - Kusan daidai<br>○ Somewhat true - Kamar daidai<br>○ Mostly true - Mafiya daidai<br>○ Completely true - Duka daidai ne
46b. In the last year, I have had questions or concerns about the safety of family planning methods<br>A shekarar da ta wuce, ina da tambayoyi ko damuwa akan ingancin hanyoyin hutun haihuwa	○ Not at all true - Duka ba daidai ba ne<br>○ Slightly true - Kusan daidai<br>○ Somewhat true - Kamar daidai<br>○ Mostly true - Mafiya daidai<br>○ Completely true - Duka daidai ne
46c. In the last year, I have discussed concerns or questions I had about the safety of family planning methods with a provider<br>A shekarar da ta wuce, na tattauna akan tambayoyi da damuwa akan ingancin hanyoyin hutun haihuwa tare da ma'aikaciyar lafiya	○ Not at all true - Duka ba daidai ba ne<br>○ Slightly true - Kusan daidai<br>○ Somewhat true - Kamar daidai<br>○ Mostly true - Mafiya daidai<br>○ Completely true - Duka daidai ne
46d. In the last year, I have discussed concerns or questions I had about the safety of family planning methods with other women<br>A shekarar da ta wuce, na tattauna akan tambayoyi da damuwa akan ingancin hanyoyin hutun haihuwa tare da wasu matan	○ Not at all true - Duka ba daidai ba ne<br>○ Slightly true - Kusan daidai<br>○ Somewhat true - Kamar daidai<br>○ Mostly true - Mafiya daidai<br>○ Completely true - Duka daidai ne
46e. In the last year, conversations with women who've had positive experiences with family planning methods have influenced my thinking about family planning<br>A shekarar da ta wuce, bayanai daga matan da suka amfana da aiki da hanyoyi hutun haihuwa ya canja mini tunanina	○ Not at all true - Duka ba daidai ba ne<br>○ Slightly true - Kusan daidai<br>○ Somewhat true - Kamar daidai<br>○ Mostly true - Mafiya daidai<br>○ Completely true - Duka daidai ne
46f. In the last year, I have received or gathered all the information I need to make a decision about if and how to space my next pregnancy<br>A shekarar da ta wuce, na samu ko tattara dukkan bayanan da nake bukata domin yanke shawara akan yadda zan huta haihuwa kafin juna biyu na gaba	○ Not at all true - Duka ba daidai ba ne<br>○ Slightly true - Kusan daidai<br>○ Somewhat true - Kamar daidai<br>○ Mostly true - Mafiya daidai<br>○ Completely true - Duka daidai ne
46g. In interactions with health care providers in the last year, I was given the opportunity to ask questions about family planning methods<br>A mu'amalata da ma'aikatan lafiya a shekarar da ta wuce, Na samu dama in yi tambayoyi akan hanyoyin hutun haihuwa	○ Not at all true - Duka ba daidai ba ne<br>○ Slightly true - Kusan daidai<br>○ Somewhat true - Kamar daidai<br>○ Mostly true - Mafiya daidai<br>○ Completely true - Duka daidai ne

46h. In interactions with health care providers in the last year, all of my questions about family planning were answered in a way I understood<br>A mu'amalata da ma'aikatan lafiya a shekarar da ta wuce, dukkan tambayoyina akan hanyoyin hutun haihuwa an amsa min kuma na gamsu	◯ Not at all true - Duka ba daidai ba ne<br>◯ Slightly true - Kusan daidai<br>◯ Somewhat true - Kamar daidai<br>◯ Mostly true - Mafiya daidai<br>◯ Completely true - Duka daidai ne

47. Different people think there are different advantages to spacing pregnancies apart. For each statement, tell me if you agree, disagree, or neither agree nor disagree that it is an advantage to spacing pregnancies.
Mutane daban-daban suna da ra'ayi daban-daban akan amfanin bada hutun samun juna biyu. A ko wane bayani ki gaya mini kin yarda ko baki yarda ba, ko babu ko daya daga ciki, cewar amfanin bada hutu tsaknin samun juna biyu. (karanta duka amsoshi a zabi dukan wanda aka yarda da shi)

47a. Mom will be healthier/ less likely to have problems<br>Uwa zata samu lafiya/ saukin samun matsaloli	◯ Agree - Yarda<br>◯ Disagree - Baki yarda ba<br>◯ Neither agree nor disagree - Babu ko daya daga ciki
47b. Current Baby will be healthier/ less likely to have problems<br>Yaro/yarinya za su samu lafiya /samun saukin samun matsolloli	◯ Agree - Yarda<br>◯ Disagree - Baki yarda ba<br>◯ Neither agree nor disagree - Babu ko daya daga ciki
47c. Next baby will be healthier/less likely to have problem<br>Yaron da za a haifa a gaba shima zai samu lafiya/ saukin matsololi	◯ Agree - Yarda<br>◯ Disagree - Baki yarda ba<br>◯ Neither agree nor disagree - Babu ko daya daga ciki
47d. Easier to feed and/or take care of other children<br>Saukin ciyarwa/ kula da sauran yara	◯ Agree - Yarda<br>◯ Disagree - Baki yarda ba<br>◯ Neither agree nor disagree - Babu ko daya daga ciki
47e. Saves money<br>Tanadin kudi	◯ Agree - Yarda<br>◯ Disagree - Baki yarda ba<br>◯ Neither agree nor disagree - Babu ko daya daga ciki
48. After delivering a baby, for the best health of the mother and baby, how long should a woman wait until getting pregnant again?<br>Bayan haihuwar yaronki, domin lafiyar uwa mai kyau da na yaron ta, tsahon wane lokaci ne mace ya kamata ta jira kafin ta sake samun juna biyu?	__________________________<br>((1 year = 12 months; 2 years = 24 months; 3 years = 36 months))
49. After the birth of a child, can a woman become pregnant before her menstrual period has returned?<br>Bayan haihuwar yaro, mace zata iya samun juna biyu kafin al'adarta ta dawo?	◯ Yes - Eh<br>◯ No - Aa<br>◯ Don't know/Unsure - Ban sani ba/ban tabbatar ba

50. I'm going to tell you a short story about a woman named Habiba and ask you a few questions about her. Habiba's baby is 2 months old. She does not want to get pregnant again yet. She would like to use a method called lactational amenorrhea method, or LAM, to prevent another pregnancy
Zan gaya miki wani dan gajeran labari a wata mata sunan ta Habiba zan miki tambayyi kadan game da ita. Yaro Habiba watan sa biyu. Bata samu ta sami wani juna biyun a yanzu. Ta naso zata yi amfani da wani tsari da ake kira bada Nonon uwa zalla, tana so tayi amfani da shi ta samu kariya daga samun juna biyu

50a. Habiba only gives her baby breastmilk but her mother in law also gives the baby water and thin porridge sometimes. To prevent pregnancy, should Habiba choose a different family planning method?<br>Habiba tana bawa yaron ta ruwan Nono kawai amma uwar mijinta/sirikarta tana bawa yaro ruwa da dan fate wani lokacin. Domin kare samun juna biyu, Habiba zata iya zabar wani hanyar hutun haihuwa?	◯ Yes - Eh<br>◯ No - Aa<br>◯ Not sure/Don't know - Ban tabbatar ba/Ban sani ba
50b. Can she continue to use LAM as a family planning method after she sees her first menses/monthly bleeding?<br>Zata ta iya ci gaba da amfanin da Bada Nono zalla a matsinyin hutun haihuwa, bayan ta ga al'adarta ta farko/jinin wata wata?	◯ Yes - Eh<br>◯ No - Aa<br>◯ Not sure/Don't know - Ban tabbatar ba/Ban sani ba
50c. What is the oldest her baby can be before LAM is no longer a good option for Habiba?<br>Wata nawa yaro zai kai kafin bada nono zalla ga yaro ya daina yiwa Habiba amfani?	______________________________<br>(Specify in Months)
51. During pregnancy, did you form a plan to deliver at a facility and start a contraceptive before leaving, for example, getting an IUD or implant after delivery and before leaving the facility?<br>Lokacin da kike da junu biyu, kinyi shirin haihuwa a asibiti ne kuma zaki fara hutun haihuwa kafin ki bar asibitin, misili, saka robar mahaifa ko dashen hannu bayan haihuwa kafin ki bar asibiti?	◯ Yes - Eh<br>◯ No - Aa
51a. If yes, what was your method choice<br>Idan Eh, wacce hanya ki ka zaba?	◯ IUCD insertion - Robar mahaifa<br>◯ Implant insertion - Dashen hannu<br>◯ Progesterone only pill<br>◯ Tubal ligation - Daurin mahaifa<br>◯ Other - Wani
Other specify	______________________________
51b. Did you receive this method prior to leaving the hospital after birth<br>Kin karbi wanan hanyar hutun kafin ki bar asibiti bayan kin haihu?	◯ Yes - Eh<br>◯ No - Aa
51b1. If No, why not<br>Idan Aa, Me yasa?	◯ I forgot - Na manta<br>◯ Changed my mind - Na chanja shawara<br>◯ The method wasn't available - Abin da na zaba<br>◯ Provider did not know how to insert - Ma'aikaciyar lafiya bata san yadda zata sa ba<br>◯ Provider thought I should wait - Ma'aikaciyar lafiya tace in jira<br>◯ Husband or mother in law disagreed - Miji ko uwar miji ba su yarda ba<br>◯ Didn't end up delivering in a facility - Ban haihu a asibiti ba<br>◯ Other - Wani
Other specify	______________________________

I would like to ask you some questions about the times you or your partner may have used a method to prevent pregnancy since [pnc_q3] was born. I will ask you to look at a timeline and tell me when certain events occurred. [Use timeline to probe for earlier periods of use and non-use, starting with most recent use, back to the first month postpartum. Using an erasable marker, mark on the laminated timeline according to women's responses to help orient her and to see the order of events. Start by writing the month of the baby's birth at the beginning of the timeline, and write each month on the timeline in order, ending with the current month at the end of the timeline (explain to the woman that you are writing the months because you will discuss events that occurred since the baby's birth, and the timeline is meant to aide in this discussion). In case the woman needs to correct a response later in the timeline activity, correct the associated mark on the timeline and go back to change the answer to the previous question. Confirm with the participant that you have the correct answer before proceeding.]

Ina so nayi miki wasu tambayoyi akan lokacin da ke ko mijinki ko kunyi amfani da hanyoyin kare juna biyu tun bayan haihuwar [pnc_q3]. Ina so in tambayeki ki duba lokacin ki gaya mini wani al'amari faru [Yi amfani da lokaci kiyi nuni da rukunin lokaci na farko na amfani ko ba na amfani ba, fara daga wata mafi kusa har zuwa watan farko bayan haihuwa. Ta hanyar yin amfani da abin rubutu da za'a iya gogewa, yi alama akan takardar nuni da lokaci, akan bisa amsoshin mata domin taimaka mata wajen gane tsarin da kuma ganin al'amarin. Fara daga watan da aka haifi yaro a farkon ma'aunin lokacin, sannan a rubuta ko wane wata a cikin tsari, a karkare da wata mafi kusa a karshen ma'aunin lokacin (Yi mata bayani cewar ana rubuta watannin saboda za'a bukaci bayani akan al'amuran da suka faru, kuma ma'aunin lokacin an yi shi ne domin ya taimakawa tattaunawar). Ko mace tana bukatar tayi gyaran amsarta daga baya akan ma'aunin lokacin, gyara alamar da ta dace akan ma'aunin lokacin sa'annan a koma, a gyara amsar zuwa daidai da tambayar baya. Tabbabatarwa mai bada amsa cewar amsar da ya/ta bayar an rubuta ta daidia kafin a ci gaba.]

54. Since the birth of [pnc_q3], have you experienced any other pregnancy<br>Tun bayan haihuwar [pnc_q3], kin fuskanci wani juna biyun?	◯ Yes - Eh<br>◯ No - Aa
54a. How old was your baby when you became pregnant<br>Watanin Yaronki/yarinyarki nawa kafin ki sami juna biyu?	________________________________<br>(Months)
54b. Are you currently pregnant<br>Kina da juna biyu yanzu	◯ Yes - Eh<br>◯ No - Aa<br>◯ Unsure -
54c. When did the pregnancy end?<br>Yaushe juna biyun ya kare?	◯ January<br>◯ February<br>◯ March<br>◯ April<br>◯ May<br>◯ June<br>◯ July<br>◯ August<br>◯ September<br>◯ October<br>◯ November<br>◯ December
Number of months after birth of baby	________________________________
54cii. How did the pregnancy end<br>Ta wacce hanya juna biyun ya kare?	◯ Miscarriage - Bari<br>◯ Termination - Cirewa<br>◯ Birth - Haihuwa

55. If it were up to you, which of the following would you prefer<br>In da zabinki ne, wane zaki zaba da ga cikin wayannan?	◯ Not ever getting pregnant again - Bakyan son ki sake samun juna biyu<br>◯ Having another child, but not getting pregnant right now - Kina son samun wani yaron, amma ba samun juna biyu a kusa ba<br>◯ Getting pregnant as soon as possible - Samun juna biyu da gaggawa<br>◯ Undecided - Ban shawarta haka tukuna<br>◯ NA: Currently pregnant - Ina da juna biyu yanzu
52. In what month did your menstrual period return after having your baby<br>Awane wata ne al'adarki ta dawo bayan haihuwar yaronki/yarinyarki?	◯ January<br>◯ February<br>◯ March<br>◯ April<br>◯ May<br>◯ June<br>◯ July<br>◯ August<br>◯ September<br>◯ October<br>◯ November<br>◯ December<br>◯ Menstrual period has not returned - Ko"al'ada bata dawo ba"
Number of months after birth of baby	______________________
53. In what month did you and your husband/partner first have sexual intercourse again after having your baby?<br>A wane wata ne ke da mijinki ku kayi saduwar farko bayan haihuwar yaronki/yarinyarki?	◯ January<br>◯ February<br>◯ March<br>◯ April<br>◯ May<br>◯ June<br>◯ July<br>◯ August<br>◯ September<br>◯ October<br>◯ November<br>◯ December<br>◯ We have not had sex since the baby was born - ba mu sadu bat un bayan haihuwa yarona/ yarinyata
Number of months after birth of baby	______________________
56. In the last 12 months since your baby was born, have you used any method of family planning to prevent pregnancy<br>Wata goma sha biyu da suka wuce bayar haihuwar yaronki/yarinyarki kin yi amfani da wata hanyar hutun haihuwa domin jinkirta samun juna biyu?	◯ Yes - Eh<br>◯ No - Aa

56a. If No, what factors contributed to you not using a form of family planning?
Idan Aa, me ya hanaki yin amfani da hanyoyin hutun haihuwa?

- ☐ Does not want to space next pregnancy/wants to get pregnant again right away - Ba na so na jinkirta samun juna biyu na gaba/ina so na sami juna biyu da wuri
- ☐ Infrequent sex/husband or partner away - Rashin saduwa akai akai/miji ko abokin zama bayana
- ☐ Not having sex - Bana saduwa da mijina
- ☐ Can't get pregnant - Bazan iya samun juna biyu ba
- ☐ Breastfeeding - Shayar da Nono
- ☐ Fatalistic/up to God - Kaddara/Yadda Allah ya so
- ☐ Has not menstruated since last birth - Bana yin al'ada tun bayan haihu
- ☐ Husband/partner opposed - Mijina/abokin zama bai yarda ba
- ☐ Respondent opposed - Bana so
- ☐ Others opposed - Wasu ba su so
- ☐ Religious prohibition - Addinin ya hana
- ☐ Knows no method - Ban san kowane hanya ba ta hutun hihuwa
- ☐ Knows no source (place to get FP) - Ban san wajen ba (wajen da zan samun huldar hutun haihuwa)
- ☐ Concerned about side effects/heard rumours about dangers/health concerns - Damuwa akan abinda zai iya haddasawa/na ji jita-jita akan hatsarin/al'amuran lafiya
- ☐ Lack of access/too far - Rashin sanin waje/Nisa
- ☐ The cost is too much - Kudin yayi yawa
- ☐ The preferred method not available - Babu hanyar da tafi dacewa
- ☐ No method available - Babu hanyoyin hutun haihuwa
- ☐ Inconvenient to use - Bana jin dadin amfani da su
- ☐ Interferes with body's normal processes - Shiga tsakanin yanayin jiki
- ☐ Health centre was not open - Ba a bude Asibitin ba
- ☐ Health provider was unfriendly - Ma'akaciyar lafiya bata da kirki
- ☐ Health provider dissuaded me from using - Ma'aikaciyar lafiya ta rinjayi ra'ayin na yin amfani
- ☐ Couldn't decide on a method - Bani da shawara kan wacce hanya
- ☐ Other - Wani

Explain why health provider dissuaded
Yi bayanin dalili

____

Other specify

____

---

57. Are you currently using a family planning method to prevent pregnancy
A yanzu kina amfani da hanyoyin hutun haihuwa domin ki kare kanki daga samu juna biyu?

- ◯ Yes - Eh
- ◯ No - Aa

57ia. What method(s) are you currently using
Wanne hanyoyi ne yanzu kike amfani dashi?

- ☐ LAM/breastfeeding - Shayar da nono zalla (LAM)/shayar da nono breastfeeding
- ☐ Calendar/rhythm/cycle beads/natural/Billing's method - Taswirar kwanan wata/rhythm/guru/haka nan/ hanyar Billing's
- ☐ Withdrawal - Azalu
- ☐ Condoms - Kwaroron roba
- ☐ Oral contraceptives - Kwayoyi na sha
- ☐ Injectables - Allurai
- ☐ Implants - Dashen hannu
- ☐ IUD - Robar mahaifa
- ☐ Emergency pill - Kwayoyin sha na gaggawa
- ☐ Sterilisation - Dandatsa
- ☐ Other - Wani dalilin

Other specify

____

57ib. Does your husband/partner know that you are currently using a method of family planning
Mijinki yasan kina amfani da hanyoyin hutun haihuwa yanzu?

- ◯ Yes - Eh
- ◯ No - Aa

57ib_a. Did you discuss together with your husband/partner before you started with this method
Kin tattautauna da maigidanki Kafin ki fara amfani da hanyoyin hutun haihuwa?

- ◯ Yes, we discussed family planning generally - Eh, mun tattauna gaba
- ◯ Yes, we discussed the specific family planning method - Eh, muntattauna akan wannan hutun hnayar haihuwar
- ◯ No, we did not discuss beforehand - Aa, bamu tattauna ba kafin nan

(Read aloud and select one)

LAM/Breastfeeding

58. When did you begin using your current/most recent method - LAM/Breastfeeding
Da yaushe kika fara amfani da hanya yanzu/mafi kusa da wannan hutun haihuwa - Shayar da nono zalla (LAM)/shayar da nono breastfeeding?

- ◯ January
- ◯ February
- ◯ March
- ◯ April
- ◯ May
- ◯ June
- ◯ July
- ◯ August
- ◯ September
- ◯ October
- ◯ November
- ◯ December

Number of months after birth of baby

____

62. What factors contributed to your current/most recent method choice - LAM/Breastfeeding
Wane dalilin ne ya taimaka wajen yin amfani da hutun haihuwa da kike amfani da shi yanzu - Shayar da nono zalla (LAM)/shayar da nono breastfeeding?

- ☐ Convenience of obtaining method - Naji dadin amfanin da shiConvenience of obtaining method
- ☐ Easy/convenient to use - Saukin amfani
- ☐ Availability - Akwai ako ina
- ☐ Cost is not too much/free - Ba tsada/kyauta
- ☐ Preferred method selected during pregnancy - Zabi na lokacin da nake da ciki
- ☐ Considered the most effective method - Ganin hanyar tana da inganci
- ☐ Unlikely to be bothered by side effects - Ba lallai in damu da larurar maganin ta daban
- ☐ Felt method was safe/few health concerns - Inajin hanyar bata da matsala/Ina jin kyan wannan hanyar/akwai darurar kan lafiya
- ☐ Health provider influenced choice - Zabin maakaciyar asibiti
- ☐ Friends/family influenced choice - Zabin abokai/'yan'uwa
- ☐ Husband influenced choice - Zabin miji
- ☐ Women in my ANC/PNC group influenced my choice - Zabin mata a lokacin awon cikina/taron bayan haihuwa
- ☐ Other - Wani

(Tick ALL options mentioned; DO NOT read the options. Probe: 'anything else?')

Other specify

____

Calendar/Rhythm/Cycle Beads

58. When did you begin using your current/most recent method - Calendar/Rhythm/Cycle Beads
Da yaushe kika fara amfani da hanya yanzu/mafi kusa da wannan hutun haihuwa - Taswirar kwanan wata/rhythm/guru/haka nan/ hanyar Billing's?

- ◯ January
- ◯ February
- ◯ March
- ◯ April
- ◯ May
- ◯ June
- ◯ July
- ◯ August
- ◯ September
- ◯ October
- ◯ November
- ◯ December

Number of months after birth of baby

____

62. What factors contributed to your current/most recent method choice - Calendar/Rhythm/Cycle Beads
Wane dalilin ne ya taimaka wajen yin amfani da hutun haihuwa da kike amfani da shi yanzu - Taswirar kwanan wata/rhythm/guru/haka nan/ hanyar Billing's?

- ☐ Convenience of obtaining method - Naji dadin amfanin da shiConvenience of obtaining method
- ☐ Easy/convenient to use - Saukin amfani
- ☐ Availability - Akwai ako ina
- ☐ Cost is not too much/free - Ba tsada/kyauta
- ☐ Preferred method selected during pregnancy - Zabi na lokacin da nake da ciki
- ☐ Considered the most effective method - Ganin hanyar tana da inganci
- ☐ Unlikely to be bothered by side effects - Ba lallai in damu da larurar maganin ta daban
- ☐ Felt method was safe/few health concerns - Inajin hanyar bata da matsala/Ina jin kyan wannan hanyar/akwai darurar kan lafiya
- ☐ Health provider influenced choice - Zabin maakaciyar asibiti
- ☐ Friends/family influenced choice - Zabin abokai/'yan'uwa
- ☐ Husband influenced choice - Zabin miji
- ☐ Women in my ANC/PNC group influenced my choice - Zabin mata a lokacin awon cikina/taron bayan haihuwa
- ☐ Other - Wani

(Tick ALL options mentioned; DO NOT read the options. Probe: 'anything else?')

Other specify

____

Withdrawal

58. When did you begin using your current/most recent method - Withdrawal
Da yaushe kika fara amfani da hanya yanzu/mafi kusa da wannan hutun haihuwa - Azalu?

- ◯ January
- ◯ February
- ◯ March
- ◯ April
- ◯ May
- ◯ June
- ◯ July
- ◯ August
- ◯ September
- ◯ October
- ◯ November
- ◯ December

Number of months after birth of baby

____

62. What factors contributed to your current/most recent method choice - Withdrawal
Wane dalilin ne ya taimaka wajen yin amfani da hutun haihuwa da kike amfani da shi yanzu - Azalu?

- [ ] Convenience of obtaining method - Naji dadin amfanin da shiConvenience of obtaining method
- [ ] Easy/convenient to use - Saukin amfani
- [ ] Availability - Akwai ako ina
- [ ] Cost is not too much/free - Ba tsada/kyauta
- [ ] Preferred method selected during pregnancy - Zabi na lokacin da nake da ciki
- [ ] Considered the most effective method - Ganin hanyar tana da inganci
- [ ] Unlikely to be bothered by side effects - Ba lallai in damu da larurar maganin ta daban
- [ ] Felt method was safe/few health concerns - Inajin hanyar bata da matsala/Ina jin kyan wannan hanyar/akwai darurar kan lafiya
- [ ] Health provider influenced choice - Zabin maakaciyar asibiti
- [ ] Friends/family influenced choice - Zabin abokai/'yan'uwa
- [ ] Husband influenced choice - Zabin miji
- [ ] Women in my ANC/PNC group influenced my choice - Zabin mata a lokacin awon cikina/taron bayan haihuwa
- [ ] Other - Wani

(Tick ALL options mentioned; DO NOT read the options. Probe: 'anything else?')

Other specify

____

Condoms

58. When did you begin using your current/most recent method - Condoms
Da yaushe kika fara amfani da hanya yanzu/mafi kusa da wannan hutun haihuwa - Kwaroron roba?

- ◯ January
- ◯ February
- ◯ March
- ◯ April
- ◯ May
- ◯ June
- ◯ July
- ◯ August
- ◯ September
- ◯ October
- ◯ November
- ◯ December

Number of months after birth of baby

____

61. From where did you obtain your current/most recent method - Condoms
Daga ina kika karbi hutun haihuwa da dakike amfani da shi yanzu - Kwaroron roba?

- ◯ Public sector health centre, doctor, or clinic - Asibiti, Likita or dakin magani na sha ka tafi
- ◯ Private or faith based sector health centre, doctor, or clinic - Wurin Sirri ko wurin addini, likita, or dakin magani na sha ka tafi
- ◯ Pharmacy - Babbab dakin shan magani na kudi
- ◯ PMS/Chemist - Dakin sayar da magani/kantin magani
- ◯ Community health worker - Ma'aikatan lafiya na unguwa
- ◯ Other - Wani

Other specify

____

62. What factors contributed to your current/most recent method choice -Condoms
Wane dalilin ne ya taimaka wajen yin amfani da hutun haihuwa da kike amfani da shi yanzu - Kwaroron roba?

- ☐ Convenience of obtaining method - Naji dadin amfanin da shiConvenience of obtaining method
- ☐ Easy/convenient to use - Saukin amfani
- ☐ Availability - Akwai ako ina
- ☐ Cost is not too much/free - Ba tsada/kyauta
- ☐ Preferred method selected during pregnancy - Zabi na lokacin da nake da ciki
- ☐ Considered the most effective method - Ganin hanyar tana da inganci
- ☐ Unlikely to be bothered by side effects - Ba lallai in damu da larurar maganin ta daban
- ☐ Felt method was safe/few health concerns - Inajin hanyar bata da matsala/Ina jin kyan wannan hanyar/akwai darurar kan lafiya
- ☐ Health provider influenced choice - Zabin maakaciyar asibiti
- ☐ Friends/family influenced choice - Zabin abokai/'yan'uwa
- ☐ Husband influenced choice - Zabin miji
- ☐ Women in my ANC/PNC group influenced my choice - Zabin mata a lokacin awon cikina/taron bayan haihuwa
- ☐ Other - Wani

(Tick ALL options mentioned; DO NOT read the options. Probe: 'anything else?')

Other specify

____

Oral Contraceptives

58. When did you begin using your current/most recent method - Oral Contraceptives
Da yaushe kika fara amfani da hanya yanzu/mafi kusa da wannan hutun haihuwa - Kwayoyi na sha?

- ◯ January
- ◯ February
- ◯ March
- ◯ April
- ◯ May
- ◯ June
- ◯ July
- ◯ August
- ◯ September
- ◯ October
- ◯ November
- ◯ December

Number of months after birth of baby

____

61. From where did you obtain your current/most recent method - Oral Contraceptives
Daga ina kika karbi hutun haihuwa da dakike amfani da shi yanzu - Kwayoyi na sha?

- ◯ Public sector health centre, doctor, or clinic - Asibiti, Likita or dakin magani na sha ka tafi
- ◯ Private or faith based sector health centre, doctor, or clinic - Wurin Sirri ko wurin addini, likita, or dakin magani na sha ka tafi
- ◯ Pharmacy - Babbab dakin shan magani na kudi
- ◯ PMS/Chemist - Dakin sayar da magani/kantin magani
- ◯ Community health worker - Ma'aikatan lafiya na unguwa
- ◯ Other - Wani

Other specify

____

62. What factors contributed to your current/most recent method choice - Oral Contraceptives
Wane dalilin ne ya taimaka wajen yin amfani da hutun haihuwa da kike amfani da shi yanzu - Kwayoyi na sha?

- ☐ Convenience of obtaining method - Naji dadin amfanin da shiConvenience of obtaining method
- ☐ Easy/convenient to use - Saukin amfani
- ☐ Availability - Akwai ako ina
- ☐ Cost is not too much/free - Ba tsada/kyauta
- ☐ Preferred method selected during pregnancy - Zabi na lokacin da nake da ciki
- ☐ Considered the most effective method - Ganin hanyar tana da inganci
- ☐ Unlikely to be bothered by side effects - Ba lallai in damu da larurar maganin ta daban
- ☐ Felt method was safe/few health concerns - Inajin hanyar bata da matsala/Ina jin kyan wannan hanyar/akwai darurar kan lafiya
- ☐ Health provider influenced choice - Zabin maakaciyar asibiti
- ☐ Friends/family influenced choice - Zabin abokai/'yan'uwa
- ☐ Husband influenced choice - Zabin miji
- ☐ Women in my ANC/PNC group influenced my choice - Zabin mata a lokacin awon cikina/taron bayan haihuwa
- ☐ Other - Wani

(Tick ALL options mentioned; DO NOT read the options. Probe: 'anything else?')

Other specify ______________________________

Injectables

58. When did you begin using your current/most recent method - Injectables
Da yaushe kika fara amfani da hanya yanzu/mafi kusa da wannan hutun haihuwa - Allurai?

- ◯ January
- ◯ February
- ◯ March
- ◯ April
- ◯ May
- ◯ June
- ◯ July
- ◯ August
- ◯ September
- ◯ October
- ◯ November
- ◯ December

Number of months after birth of baby ______________________________

61. From where did you obtain your current/most recent method - Injectables
Daga ina kika karbi hutun haihuwa da dakike amfani da shi yanzu - Allurai?

- ◯ Public sector health centre, doctor, or clinic - Asibiti, Likita or dakin magani na sha ka tafi
- ◯ Private or faith based sector health centre, doctor, or clinic - Wurin Sirri ko wurin addini, likita, or dakin magani na sha ka tafi
- ◯ Pharmacy - Babbab dakin shan magani na kudi
- ◯ PMS/Chemist - Dakin sayar da magani/kantin magani
- ◯ Community health worker - Ma'aikatan lafiya na unguwa
- ◯ Other - Wani

Other specify ______________________________

62. What factors contributed to your current/most recent method choice - Injectables<br>Wane dalilin ne ya taimaka wajen yin amfani da hutun haihuwa da kike amfani da shi yanzu - Allurai?	☐ Convenience of obtaining method - Naji dadin amfanin da shiConvenience of obtaining method<br>☐ Easy/convenient to use - Saukin amfani<br>☐ Availability - Akwai ako ina<br>☐ Cost is not too much/free - Ba tsada/kyauta<br>☐ Preferred method selected during pregnancy - Zabi na lokacin da nake da ciki<br>☐ Considered the most effective method - Ganin hanyar tana da inganci<br>☐ Unlikely to be bothered by side effects - Ba lallai in damu da larurar maganin ta daban<br>☐ Felt method was safe/few health concerns - Inajin hanyar bata da matsala/Ina jin kyan wannan hanyar/akwai darurar kan lafiya<br>☐ Health provider influenced choice - Zabin maakaciyar asibiti<br>☐ Friends/family influenced choice - Zabin abokai/'yan'uwa<br>☐ Husband influenced choice - Zabin miji<br>☐ Women in my ANC/PNC group influenced my choice - Zabin mata a lokacin awon cikina/taron bayan haihuwa<br>☐ Other - Wani<br>(Tick ALL options mentioned; DO NOT read the options. Probe: 'anything else?')
Other specify	__________________________________

Implants

58. When did you begin using your current/most recent method - Implants<br>Da yaushe kika fara amfani da hanya yanzu/mafi kusa da wannan hutun haihuwa - Dashen hannu?	◯ January<br>◯ February<br>◯ March<br>◯ April<br>◯ May<br>◯ June<br>◯ July<br>◯ August<br>◯ September<br>◯ October<br>◯ November<br>◯ December
Number of months after birth of baby	__________________________________
61. From where did you obtain your current/most recent method - Implants<br>Daga ina kika karbi hutun haihuwa da dakike amfani da shi yanzu - Dashen hannu?	◯ Public sector health centre, doctor, or clinic - Asibiti, Likita or dakin magani na sha ka tafi<br>◯ Private or faith based sector health centre, doctor, or clinic - Wurin Sirri ko wurin addini, likita, or dakin magani na sha ka tafi<br>◯ Pharmacy - Babbab dakin shan magani na kudi<br>◯ PMS/Chemist - Dakin sayar da magani/kantin magani<br>◯ Community health worker - Ma'aikatan lafiya na unguwa<br>◯ Other - Wani
Other specify	__________________________________

62. What factors contributed to your current/most recent method choice - Implants<br>Wane dalilin ne ya taimaka wajen yin amfani da hutun haihuwa da kike amfani da shi yanzu - Dashen hannu?	☐ Convenience of obtaining method - Naji dadin amfanin da shiConvenience of obtaining method<br>☐ Easy/convenient to use - Saukin amfani<br>☐ Availability - Akwai ako ina<br>☐ Cost is not too much/free - Ba tsada/kyauta<br>☐ Preferred method selected during pregnancy - Zabi na lokacin da nake da ciki<br>☐ Considered the most effective method - Ganin hanyar tana da inganci<br>☐ Unlikely to be bothered by side effects - Ba lallai in damu da larurar maganin ta daban<br>☐ Felt method was safe/few health concerns - Inajin hanyar bata da matsala/Ina jin kyan wannan hanyar/akwai darurar kan lafiya<br>☐ Health provider influenced choice - Zabin maakaciyar asibiti<br>☐ Friends/family influenced choice - Zabin abokai/'yan'uwa<br>☐ Husband influenced choice - Zabin miji<br>☐ Women in my ANC/PNC group influenced my choice - Zabin mata a lokacin awon cikina/taron bayan haihuwa<br>☐ Other - Wani<br>(Tick ALL options mentioned; DO NOT read the options. Probe: 'anything else?')
Other specify	__________________________

IUD

58. When did you begin using your current/most recent method - IUD<br>Da yaushe kika fara amfani da hanya yanzu/mafi kusa da wannan hutun haihuwa - Robar mahaifa?	◯ January<br>◯ February<br>◯ March<br>◯ April<br>◯ May<br>◯ June<br>◯ July<br>◯ August<br>◯ September<br>◯ October<br>◯ November<br>◯ December
Number of months after birth of baby	__________________________
61. From where did you obtain your current/most recent method - IUD<br>Daga ina kika karbi hutun haihuwa da dakike amfani da shi yanzu - Robar mahaifa?	◯ Public sector health centre, doctor, or clinic - Asibiti, Likita or dakin magani na sha ka tafi<br>◯ Private or faith based sector health centre, doctor, or clinic - Wurin Sirri ko wurin addini, likita, or dakin magani na sha ka tafi<br>◯ Pharmacy - Babbab dakin shan magani na kudi<br>◯ PMS/Chemist - Dakin sayar da magani/kantin magani<br>◯ Community health worker - Ma'aikatan lafiya na unguwa<br>◯ Other - Wani
Other specify	__________________________

62. What factors contributed to your current/most recent method choice - IUD
Wane dalilin ne ya taimaka wajen yin amfani da hutun haihuwa da kike amfani da shi yanzu - Robar mahaifa?

- ☐ Convenience of obtaining method - Naji dadin amfanin da shiConvenience of obtaining method
- ☐ Easy/convenient to use - Saukin amfani
- ☐ Availability - Akwai ako ina
- ☐ Cost is not too much/free - Ba tsada/kyauta
- ☐ Preferred method selected during pregnancy - Zabi na lokacin da nake da ciki
- ☐ Considered the most effective method - Ganin hanyar tana da inganci
- ☐ Unlikely to be bothered by side effects - Ba lallai in damu da larurar maganin ta daban
- ☐ Felt method was safe/few health concerns - Inajin hanyar bata da matsala/Ina jin kyan wannan hanyar/akwai darurar kan lafiya
- ☐ Health provider influenced choice - Zabin maakaciyar asibiti
- ☐ Friends/family influenced choice - Zabin abokai/'yan'uwa
- ☐ Husband influenced choice - Zabin miji
- ☐ Women in my ANC/PNC group influenced my choice - Zabin mata a lokacin awon cikina/taron bayan haihuwa
- ☐ Other - Wani

(Tick ALL options mentioned; DO NOT read the options. Probe: 'anything else?')

Other specify

____

Emergency Contraception Pill

58. When did you begin using your current/most recent method - Emergency pill
Da yaushe kika fara amfani da hanya yanzu/mafi kusa da wannan hutun haihuwa - Kwayoyin sha na gaggawa?

- ◯ January
- ◯ February
- ◯ March
- ◯ April
- ◯ May
- ◯ June
- ◯ July
- ◯ August
- ◯ September
- ◯ October
- ◯ November
- ◯ December

Number of months after birth of baby

____

61. From where did you obtain your current/most recent method - Emergency pill
Daga ina kika karbi hutun haihuwa da dakike amfani da shi yanzu - Kwayoyin sha na gaggawa?

- ◯ Public sector health centre, doctor, or clinic - Asibiti, Likita or dakin magani na sha ka tafi
- ◯ Private or faith based sector health centre, doctor, or clinic - Wurin Sirri ko wurin addini, likita, or dakin magani na sha ka tafi
- ◯ Pharmacy - Babbab dakin shan magani na kudi
- ◯ PMS/Chemist - Dakin sayar da magani/kantin magani
- ◯ Community health worker - Ma'aikatan lafiya na unguwa
- ◯ Other - Wani

Other specify

____

62. What factors contributed to your current/most recent method choice - Emergency pill<br>Wane dalilin ne ya taimaka wajen yin amfani da hutun haihuwa da kike amfani da shi yanzu - Kwayoyin sha na gaggawa?	☐ Convenience of obtaining method - Naji dadin amfanin da shiConvenience of obtaining method<br>☐ Easy/convenient to use - Saukin amfani<br>☐ Availability - Akwai ako ina<br>☐ Cost is not too much/free - Ba tsada/kyauta<br>☐ Preferred method selected during pregnancy - Zabi na lokacin da nake da ciki<br>☐ Considered the most effective method - Ganin hanyar tana da inganci<br>☐ Unlikely to be bothered by side effects - Ba lallai in damu da larurar maganin ta daban<br>☐ Felt method was safe/few health concerns - Inajin hanyar bata da matsala/Ina jin kyan wannan hanyar/akwai darurar kan lafiya<br>☐ Health provider influenced choice - Zabin maakaciyar asibiti<br>☐ Friends/family influenced choice - Zabin abokai/'yan'uwa<br>☐ Husband influenced choice - Zabin miji<br>☐ Women in my ANC/PNC group influenced my choice - Zabin mata a lokacin awon cikina/taron bayan haihuwa<br>☐ Other - Wani<br>(Tick ALL options mentioned; DO NOT read the options. Probe: 'anything else?')
Other specify	__________________________________

Sterilisation

58. When did you begin using your current/most recent method - Sterilisation<br>Da yaushe kika fara amfani da hanya yanzu/mafi kusa da wannan hutun haihuwa - Dandatsa ?	◯ January<br>◯ February<br>◯ March<br>◯ April<br>◯ May<br>◯ June<br>◯ July<br>◯ August<br>◯ September<br>◯ October<br>◯ November<br>◯ December
Number of months after birth of baby	__________________________________
61. From where did you obtain your current/most recent method - Sterilisation<br>Daga ina kika karbi hutun haihuwa da dakike amfani da shi yanzu - Dandatsa ?	◯ Public sector health centre, doctor, or clinic - Asibiti, Likita or dakin magani na sha ka tafi<br>◯ Private or faith based sector health centre, doctor, or clinic - Wurin Sirri ko wurin addini, likita, or dakin magani na sha ka tafi<br>◯ Pharmacy - Babbab dakin shan magani na kudi<br>◯ PMS/Chemist - Dakin sayar da magani/kantin magani<br>◯ Community health worker - Ma'aikatan lafiya na unguwa<br>◯ Other - Wani
Other specify	__________________________________

62. What factors contributed to your current/most recent method choice - Sterilisation<br>Wane dalilin ne ya taimaka wajen yin amfani da hutun haihuwa da kike amfani da shi yanzu - Dandatsa ?	☐ Convenience of obtaining method - Naji dadin amfanin da shiConvenience of obtaining method<br>☐ Easy/convenient to use - Saukin amfani<br>☐ Availability - Akwai ako ina<br>☐ Cost is not too much/free - Ba tsada/kyauta<br>☐ Preferred method selected during pregnancy - Zabi na lokacin da nake da ciki<br>☐ Considered the most effective method - Ganin hanyar tana da inganci<br>☐ Unlikely to be bothered by side effects - Ba lallai in damu da larurar maganin ta daban<br>☐ Felt method was safe/few health concerns - Inajin hanyar bata da matsala/Ina jin kyan wannan hanyar/akwai darurar kan lafiya<br>☐ Health provider influenced choice - Zabin maakaciyar asibiti<br>☐ Friends/family influenced choice - Zabin abokai/'yan'uwa<br>☐ Husband influenced choice - Zabin miji<br>☐ Women in my ANC/PNC group influenced my choice - Zabin mata a lokacin awon cikina/taron bayan haihuwa<br>☐ Other - Wani<br>(Tick ALL options mentioned; DO NOT read the options. Probe: 'anything else?')
Other specify	__________________________
Other FP ([pnc_q57i_a_other])	
58. When did you begin using your current/most recent method - [pnc_q57i_a_other]<br>Da yaushe kika fara amfani da hanya yanzu/mafi kusa da wannan hutun haihuwa - [pnc_q57i_a_other]?	○ January<br>○ February<br>○ March<br>○ April<br>○ May<br>○ June<br>○ July<br>○ August<br>○ September<br>○ October<br>○ November<br>○ December
Number of months after birth of baby	__________________________
61. From where did you obtain your current/most recent method - [pnc_q57i_a_other]<br>Daga ina kika karbi hutun haihuwa da dakike amfani da shi yanzu - [pnc_q57i_a_other]?	○ Public sector health centre, doctor, or clinic - Asibiti, Likita or dakin magani na sha ka tafi<br>○ Private or faith based sector health centre, doctor, or clinic - Wurin Sirri ko wurin addini, likita, or dakin magani na sha ka tafi<br>○ Pharmacy - Babbab dakin shan magani na kudi<br>○ PMS/Chemist - Dakin sayar da magani/kantin magani<br>○ Community health worker - Ma'aikatan lafiya na unguwa<br>○ Other - Wani
Other specify	__________________________

62. What factors contributed to your current/most recent method choice - [pnc_q57i_a_other]
Wane dalilin ne ya taimaka wajen yin amfani da hutun haihuwa da kike amfani da shi yanzu - [pnc_q57i_a_other]?

- ☐ Convenience of obtaining method - Naji dadin amfanin da shiConvenience of obtaining method
- ☐ Easy/convenient to use - Saukin amfani
- ☐ Availability - Akwai ako ina
- ☐ Cost is not too much/free - Ba tsada/kyauta
- ☐ Preferred method selected during pregnancy - Zabi na lokacin da nake da ciki
- ☐ Considered the most effective method - Ganin hanyar tana da inganci
- ☐ Unlikely to be bothered by side effects - Ba lallai in damu da larurar maganin ta daban
- ☐ Felt method was safe/few health concerns - Inajin hanyar bata da matsala/Ina jin kyan wannan hanyar/akwai darurar kan lafiya
- ☐ Health provider influenced choice - Zabin maakaciyar asibiti
- ☐ Friends/family influenced choice - Zabin abokai/'yan'uwa
- ☐ Husband influenced choice - Zabin miji
- ☐ Women in my ANC/PNC group influenced my choice - Zabin mata a lokacin awon cikina/taron bayan haihuwa
- ☐ Other - Wani

(Tick ALL options mentioned; DO NOT read the options. Probe: 'anything else?')

Other specify

____

57iia. If No, what is the most recent method of family planning you have used
Idan Aa, wane hanyar hutun haihuwa kike yi amfani dashi mafi kusa?

- ☐ LAM/breastfeeding - Shayar da nono zalla (LAM)/shayar da nono breastfeeding
- ☐ Calendar/rhythm/cycle beads/natural/Billing's method - Taswirar kwanan wata/rhythm/guru/haka nan/ hanyar Billing's
- ☐ Withdrawal - Azalu
- ☐ Condoms - Kwaroron roba
- ☐ Oral contraceptives - Kwayoyi na sha
- ☐ Injectables - Allurai
- ☐ Implants - Dashen hannu
- ☐ IUD - Robar mahaifa
- ☐ Emergency pill - Kwayoyin sha na gaggawa
- ☐ Sterilisation - Dandatsa
- ☐ Other - Wani dalilin
- ☐ None -

Other specify

____

57iib. Did your husband/partner know that you were using a method of family planning
Mijinki yasan kina amfani da hanyar hutun haihuwa?

- ◯ Yes - Eh
- ◯ No - Aa

57iib_a. If Yes, did you discuss together with your husband/partner before you started using this method
Idan Eh, kin tattatauna da mijinki kafin ki fara amfanin da wannan hanyar hutun haihuwa?

- ◯ Yes, we discussed family planning generally - Eh, mun tattauna gaba
- ◯ Yes, we discussed the specific family planning method - Eh, muntattauna akan wannan hutun hnayar haihuwar
- ◯ No, we did not discuss beforehand - Aa, bamu tattauna ba kafin nan

(Read aloud and select one)

59. When did you stop using this most recent method
Yaushe kika daina afani?

- ◯ January
- ◯ February
- ◯ March
- ◯ April
- ◯ May
- ◯ June
- ◯ July
- ◯ August
- ◯ September
- ◯ October
- ◯ November
- ◯ December

Number of months after birth of baby

____

60. Why did you stop using this most recent method
Me yasa ki ka dakata da amfani da hanyar hutun haihuwa yanzu?

- ☐ Infrequent sex/husband or partner away - Rashin saduwa/maigida baya gari
- ☐ Became pregnant while using - Samun juna lokacin da kae amfani da hutun haihuwa
- ☐ Wanted to become pregnant - Ina so in sami juna biyu
- ☐ Husband/partner opposed - Mijinki bai yarda ba
- ☐ Others opposed - Wasu basu yarda ba
- ☐ Wanted more effective method - Ina son ingantaccien hutun haihuwa
- ☐ Side effects/health concerns - Laruran maganin na daban/damuwa da lafiya
- ☐ Lack of access/too far - Rashin samu/waje yayi nisa
- ☐ Costs too much - Yayi tsada da yawa
- ☐ Inconvenient to use - Banji dadin amfani da shi ba
- ☐ Up to God/fatalistic - Yadda Allah ya so/Gaddara
- ☐ Difficult to get pregnant/menopausal - Da wuaya na samu juna biyu/Na daina haihuwa
- ☐ Marital dissolution/separation - Rabuwa da maigida
- ☐ Other - Wani

(Tick ALL options mentioned; DO NOT read the options. Probe: 'any other reasons?)

Other specify

____

63. After the birth of your baby but before using this (these) current/most recent method(s), did you or your partner do something or use a different method to avoid pregnancy
Bayan haihuwar yaronki/yarinyarki kafin ki fara amfani da wannan hanyar hutun haihuwa mafi kusa, mijinki yayi amfani da wata hanya domin kariya daga samun juna biyu?

- ◯ Yes - Eh
- ◯ No - Aa

64. What other methods have you used to avoid pregnancy since the birth of your last baby?
Wanne hanyoyin hutun haihuwa kika yi amfani da su domin samun kariya daga samun juna biyu tun bayan haihuwar yarokin/yarinyarki

- ☐ LAM/breastfeeding - Shayar da nono zalla (LAM)/shayar da nono breastfeeding
- ☐ Calendar/rhythm/cycle beads/natural/Billing's method - Taswirar kwanan wata/rhythm/guru/haka nan/ hanyar Billing's
- ☐ Withdrawal - Azalu
- ☐ Condoms - Kwaroron roba
- ☐ Oral contraceptives - Kwayoyi na sha
- ☐ Injectables - Allurai
- ☐ Implants - Dashen hannu
- ☐ IUD - Robar mahaifa
- ☐ Emergency pill - Kwayoyin sha na gaggawa
- ☐ Sterilisation - Dandatsa
- ☐ Other - Wani dalilin

Other FP method

____

LAM/Breastfeeding

65. When did you begin using LAM/Breastfeeding
Yaushe kika fara amfani Shayar da nono zalla (LAM)/shayar da nono breastfeeding

- ◯ January
- ◯ February
- ◯ March
- ◯ April
- ◯ May
- ◯ June
- ◯ July
- ◯ August
- ◯ September
- ◯ October
- ◯ November
- ◯ December

Number of months after birth of baby

____

66. When did you stop using LAM/breastfeeding
Yaushe kika dakata da amfani Shayar da nono zalla (LAM)/shayar da nono breastfeeding

- ◯ January
- ◯ February
- ◯ March
- ◯ April
- ◯ May
- ◯ June
- ◯ July
- ◯ August
- ◯ September
- ◯ October
- ◯ November
- ◯ December

Number of months after birth of baby

____

67. Why did you stop using LAM/breastfeeding
Me yasa kika daina amfani da Shayar da nono zalla (LAM)/shayar da nono breastfeeding

- ☐ Infrequent sex/husband or partner away - Rashin saduwa/maigida baya gari
- ☐ Became pregnant while using - Samun juna lokacin da kae amfani da hutun haihuwa
- ☐ Wanted to become pregnant - Ina so in sami juna biyu
- ☐ Husband/partner opposed - Mijinki bai yarda ba
- ☐ Others opposed - Wasu basu yarda ba
- ☐ Wanted more effective method - Ina son ingantaccien hutun haihuwa
- ☐ Side effects/health concerns - Laruran maganin na daban/damuwa da lafiya
- ☐ Lack of access/too far - Rashin samu/waje yayi nisa
- ☐ Costs too much - Yayi tsada da yawa
- ☐ Inconvenient to use - Banji dadin amfani da shi ba
- ☐ Up to God/fatalistic - Yadda Allah ya so/Gaddara
- ☐ Difficult to get pregnant/menopausal - Da wuaya na samu juna biyu/Na daina haihuwa
- ☐ Marital dissolution/separation - Rabuwa da maigida
- ☐ Other - Wani

Other specify

____

69. What factors contributed to your decision to use LAM/breastfeeding
Wane dalili ne ya taimaka miki wajen yanke shawara yin amfani da shi Shayar da nono zalla (LAM)/shayar da nono breastfeeding

- ☐ Convenience of obtaining method - Naji dadin amfanin da shiConvenience of obtaining method
- ☐ Easy/convenient to use - Saukin amfani
- ☐ Availability - Akwai ako ina
- ☐ Cost is not too much/free - Ba tsada/kyauta
- ☐ Preferred method selected during pregnancy - Zabi na lokacin da nake da ciki
- ☐ Considered the most effective method - Ganin hanyar tana da inganci
- ☐ Unlikely to be bothered by side effects - Ba lallai in damu da larurar maganin ta daban
- ☐ Felt method was safe/few health concerns - Inajin hanyar bata da matsala/Ina jin kyan wannan hanyar/akwai darurar kan lafiya
- ☐ Health provider influenced choice - Zabin maakaciyar asibiti
- ☐ Friends/family influenced choice - Zabin abokai/'yan'uwa
- ☐ Husband influenced choice - Zabin miji
- ☐ Women in my ANC/PNC group influenced my choice - Zabin mata a lokacin awon cikina/taron bayan haihuwa
- ☐ Other - Wani

Other specify

____

Calendar/Rhythm/Cycle Beads

65. When did you begin using Calendar/rhythm/cycle beads/natural method
Yaushe kika fara amfani Taswirar kwanan wata/rhythm/guru/haka nan

- ◯ January
- ◯ February
- ◯ March
- ◯ April
- ◯ May
- ◯ June
- ◯ July
- ◯ August
- ◯ September
- ◯ October
- ◯ November
- ◯ December

Number of months after birth of baby

____

66. When did you stop using Calendar/rhythm/cycle beads/natural method
Yaushe kika dakata da amfani Taswirar kwanan wata/rhythm/guru/haka nan

- ◯ January
- ◯ February
- ◯ March
- ◯ April
- ◯ May
- ◯ June
- ◯ July
- ◯ August
- ◯ September
- ◯ October
- ◯ November
- ◯ December

Number of months after birth of baby

____

67. Why did you stop using Calendar/rhythm/cycle beads/natural method
Me yasa kika daina amfani da Taswirar kwanan wata/rhythm/guru/haka nan

- ☐ Infrequent sex/husband or partner away - Rashin saduwa/maigida baya gari
- ☐ Became pregnant while using - Samun juna lokacin da kae amfani da hutun haihuwa
- ☐ Wanted to become pregnant - Ina so in sami juna biyu
- ☐ Husband/partner opposed - Mijinki bai yarda ba
- ☐ Others opposed - Wasu basu yarda ba
- ☐ Wanted more effective method - Ina son ingantaccien hutun haihuwa
- ☐ Side effects/health concerns - Laruran maganin na daban/damuwa da lafiya
- ☐ Lack of access/too far - Rashin samu/waje yayi nisa
- ☐ Costs too much - Yayi tsada da yawa
- ☐ Inconvenient to use - Banji dadin amfani da shi ba
- ☐ Up to God/fatalistic - Yadda Allah ya so/Gaddara
- ☐ Difficult to get pregnant/menopausal - Da wuaya na samu juna biyu/Na daina haihuwa
- ☐ Marital dissolution/separation - Rabuwa da maigida
- ☐ Other - Wani

Other specify

____

69. What factors contributed to your decision to use Calendar/rhythm/cycle beads/natural method
Wane dalili ne ya taimaka miki wajen yanke shawara yin amfani da shi Taswirar kwanan wata/rhythm/guru/haka nan

- ☐ Convenience of obtaining method - Naji dadin amfanin da shiConvenience of obtaining method
- ☐ Easy/convenient to use - Saukin amfani
- ☐ Availability - Akwai ako ina
- ☐ Cost is not too much/free - Ba tsada/kyauta
- ☐ Preferred method selected during pregnancy - Zabi na lokacin da nake da ciki
- ☐ Considered the most effective method - Ganin hanyar tana da inganci
- ☐ Unlikely to be bothered by side effects - Ba lallai in damu da larurar maganin ta daban
- ☐ Felt method was safe/few health concerns - Inajin hanyar bata da matsala/Ina jin kyan wannan hanyar/akwai darurar kan lafiya
- ☐ Health provider influenced choice - Zabin maakaciyar asibiti
- ☐ Friends/family influenced choice - Zabin abokai/'yan'uwa
- ☐ Husband influenced choice - Zabin miji
- ☐ Women in my ANC/PNC group influenced my choice - Zabin mata a lokacin awon cikina/taron bayan haihuwa
- ☐ Other - Wani

Other specify

____

Withdrawal

65. When did you begin using Withdrawal
Yaushe kika fara amfani Azalu

- ◯ January
- ◯ February
- ◯ March
- ◯ April
- ◯ May
- ◯ June
- ◯ July
- ◯ August
- ◯ September
- ◯ October
- ◯ November
- ◯ December

Number of months after birth of baby

____

66. When did you stop using Withdrawal
Yaushe kika dakata da amfani Azalu

- ◯ January
- ◯ February
- ◯ March
- ◯ April
- ◯ May
- ◯ June
- ◯ July
- ◯ August
- ◯ September
- ◯ October
- ◯ November
- ◯ December

Number of months after birth of baby

____

67. Why did you stop using Withdrawal method<br>Me yasa kika daina amfani da Azalu	☐ Infrequent sex/husband or partner away - Rashin saduwa/maigida baya gari<br>☐ Became pregnant while using - Samun juna lokacin da kae amfani da hutun haihuwa<br>☐ Wanted to become pregnant - Ina so in sami juna biyu<br>☐ Husband/partner opposed - Mijinki bai yarda ba<br>☐ Others opposed - Wasu basu yarda ba<br>☐ Wanted more effective method - Ina son ingantaccien hutun haihuwa<br>☐ Side effects/health concerns - Laruran maganin na daban/damuwa da lafiya<br>☐ Lack of access/too far - Rashin samu/waje yayi nisa<br>☐ Costs too much - Yayi tsada da yawa<br>☐ Inconvenient to use - Banji dadin amfani da shi ba<br>☐ Up to God/fatalistic - Yadda Allah ya so/Gaddara<br>☐ Difficult to get pregnant/menopausal - Da wuaya na samu juna biyu/Na daina haihuwa<br>☐ Marital dissolution/separation - Rabuwa da maigida<br>☐ Other - Wani
Other specify	__________________________________
69. What factors contributed to your decision to use Withdrawal<br>Wane dalili ne ya taimaka miki wajen yanke shawara yin amfani da shi Azalu	☐ Convenience of obtaining method - Naji dadin amfanin da shiConvenience of obtaining method<br>☐ Easy/convenient to use - Saukin amfani<br>☐ Availability - Akwai ako ina<br>☐ Cost is not too much/free - Ba tsada/kyauta<br>☐ Preferred method selected during pregnancy - Zabi na lokacin da nake da ciki<br>☐ Considered the most effective method - Ganin hanyar tana da inganci<br>☐ Unlikely to be bothered by side effects - Ba lallai in damu da larurar maganin ta daban<br>☐ Felt method was safe/few health concerns - Inajin hanyar bata da matsala/Ina jin kyan wannan hanyar/akwai darurar kan lafiya<br>☐ Health provider influenced choice - Zabin maakaciyar asibiti<br>☐ Friends/family influenced choice - Zabin abokai/'yan'uwa<br>☐ Husband influenced choice - Zabin miji<br>☐ Women in my ANC/PNC group influenced my choice - Zabin mata a lokacin awon cikina/taron bayan haihuwa<br>☐ Other - Wani
Other specify	__________________________________

Condoms

65. When did you begin using Condoms
Yaushe kika fara amfani Kwaroron roba

- ◯ January
- ◯ February
- ◯ March
- ◯ April
- ◯ May
- ◯ June
- ◯ July
- ◯ August
- ◯ September
- ◯ October
- ◯ November
- ◯ December

Number of months after birth of baby

____

66. When did you stop using Condoms
Yaushe kika dakata da amfani Kwaroron roba

- ◯ January
- ◯ February
- ◯ March
- ◯ April
- ◯ May
- ◯ June
- ◯ July
- ◯ August
- ◯ September
- ◯ October
- ◯ November
- ◯ December

Number of months after birth of baby

____

67. Why did you stop using Condoms
Me yasa kika daina amfani da Kwaroron roba

- ☐ Infrequent sex/husband or partner away - Rashin saduwa/maigida baya gari
- ☐ Became pregnant while using - Samun juna lokacin da kae amfani da hutun haihuwa
- ☐ Wanted to become pregnant - Ina so in sami juna biyu
- ☐ Husband/partner opposed - Mijinki bai yarda ba
- ☐ Others opposed - Wasu basu yarda ba
- ☐ Wanted more effective method - Ina son ingantaccien hutun haihuwa
- ☐ Side effects/health concerns - Laruran maganin na daban/damuwa da lafiya
- ☐ Lack of access/too far - Rashin samu/waje yayi nisa
- ☐ Costs too much - Yayi tsada da yawa
- ☐ Inconvenient to use - Banji dadin amfani da shi ba
- ☐ Up to God/fatalistic - Yadda Allah ya so/Gaddara
- ☐ Difficult to get pregnant/menopausal - Da wuaya na samu juna biyu/Na daina haihuwa
- ☐ Marital dissolution/separation - Rabuwa da maigida
- ☐ Other - Wani

Other specify

____

68. From where did you obtain Condoms
Daga ina kika samu Kwaroron roba

- ◯ The same facility where I received antenatal care (ANC)
- ◯ A different public sector health centre, doctor, or clinic from where you I antenatal care (ANC) - Asibiti, Likita or dakin magani na sha ka tafi
- ◯ Private or faith based sector health centre, doctor, or clinic - Wurin Sirri ko wurin addini, likita, or dakin magani na sha ka tafi
- ◯ Pharmacy - Babbab dakin shan magani na kudi
- ◯ PMS/Chemist - Dakin sayar da magani/kantin magani
- ◯ Community health worker - Ma'aikatan lafiya na unguwa
- ◯ Other - Wani

Other specify

____

69. What factors contributed to your decision to use Condoms
Wane dalili ne ya taimaka miki wajen yanke shawara yin amfani da shi Kwaroron roba

- ☐ Convenience of obtaining method - Naji dadin amfanin da shiConvenience of obtaining method
- ☐ Easy/convenient to use - Saukin amfani
- ☐ Availability - Akwai ako ina
- ☐ Cost is not too much/free - Ba tsada/kyauta
- ☐ Preferred method selected during pregnancy - Zabi na lokacin da nake da ciki
- ☐ Considered the most effective method - Ganin hanyar tana da inganci
- ☐ Unlikely to be bothered by side effects - Ba lallai in damu da larurar maganin ta daban
- ☐ Felt method was safe/few health concerns - Inajin hanyar bata da matsala/Ina jin kyan wannan hanyar/akwai darurar kan lafiya
- ☐ Health provider influenced choice - Zabin maakaciyar asibiti
- ☐ Friends/family influenced choice - Zabin abokai/'yan'uwa
- ☐ Husband influenced choice - Zabin miji
- ☐ Women in my ANC/PNC group influenced my choice - Zabin mata a lokacin awon cikina/taron bayan haihuwa
- ☐ Other - Wani

Other specify

____

Oral Contraceptives

65. When did you begin using Oral contraceptives
Yaushe kika fara amfani Kwayoyi na sha

- ◯ January
- ◯ February
- ◯ March
- ◯ April
- ◯ May
- ◯ June
- ◯ July
- ◯ August
- ◯ September
- ◯ October
- ◯ November
- ◯ December

Number of months after birth of baby

____

66. When did you stop using Oral contraceptives
Yaushe kika dakata da amfani Kwayoyi na sha

- ◯ January
- ◯ February
- ◯ March
- ◯ April
- ◯ May
- ◯ June
- ◯ July
- ◯ August
- ◯ September
- ◯ October
- ◯ November
- ◯ December

Number of months after birth of baby

____

67. Why did you stop using Oral contraceptives
Me yasa kika daina amfani da Kwayoyi na sha

- ☐ Infrequent sex/husband or partner away - Rashin saduwa/maigida baya gari
- ☐ Became pregnant while using - Samun juna lokacin da kae amfani da hutun haihuwa
- ☐ Wanted to become pregnant - Ina so in sami juna biyu
- ☐ Husband/partner opposed - Mijinki bai yarda ba
- ☐ Others opposed - Wasu basu yarda ba
- ☐ Wanted more effective method - Ina son ingantaccien hutun haihuwa
- ☐ Side effects/health concerns - Laruran maganin na daban/damuwa da lafiya
- ☐ Lack of access/too far - Rashin samu/waje yayi nisa
- ☐ Costs too much - Yayi tsada da yawa
- ☐ Inconvenient to use - Banji dadin amfani da shi ba
- ☐ Up to God/fatalistic - Yadda Allah ya so/Gaddara
- ☐ Difficult to get pregnant/menopausal - Da wuaya na samu juna biyu/Na daina haihuwa
- ☐ Marital dissolution/separation - Rabuwa da maigida
- ☐ Other - Wani

Other specify

____

68. From where did you obtain Oral contraceptives
Daga ina kika samu Kwayoyi na sha

- ◯ The same facility where I received antenatal care (ANC)
- ◯ A different public sector health centre, doctor, or clinic from where you I antenatal care (ANC) - Asibiti, Likita or dakin magani na sha ka tafi
- ◯ Private or faith based sector health centre, doctor, or clinic - Wurin Sirri ko wurin addini, likita, or dakin magani na sha ka tafi
- ◯ Pharmacy - Babbab dakin shan magani na kudi
- ◯ PMS/Chemist - Dakin sayar da magani/kantin magani
- ◯ Community health worker - Ma'aikatan lafiya na unguwa
- ◯ Other - Wani

Other specify

____

69. What factors contributed to your decision to use Oral contraceptives
Wane dalili ne ya taimaka miki wajen yanke shawara yin amfani da shi Kwayoyi na sha

- ☐ Convenience of obtaining method - Naji dadin amfanin da shiConvenience of obtaining method
- ☐ Easy/convenient to use - Saukin amfani
- ☐ Availability - Akwai ako ina
- ☐ Cost is not too much/free - Ba tsada/kyauta
- ☐ Preferred method selected during pregnancy - Zabi na lokacin da nake da ciki
- ☐ Considered the most effective method - Ganin hanyar tana da inganci
- ☐ Unlikely to be bothered by side effects - Ba lallai in damu da larurar maganin ta daban
- ☐ Felt method was safe/few health concerns - Inajin hanyar bata da matsala/Ina jin kyan wannan hanyar/akwai darurar kan lafiya
- ☐ Health provider influenced choice - Zabin maakaciyar asibiti
- ☐ Friends/family influenced choice - Zabin abokai/'yan'uwa
- ☐ Husband influenced choice - Zabin miji
- ☐ Women in my ANC/PNC group influenced my choice - Zabin mata a lokacin awon cikina/taron bayan haihuwa
- ☐ Other - Wani

Other specify

____

Injectable

65. When did you begin using Injectables
Yaushe kika fara amfani Allurai

- ◯ January
- ◯ February
- ◯ March
- ◯ April
- ◯ May
- ◯ June
- ◯ July
- ◯ August
- ◯ September
- ◯ October
- ◯ November
- ◯ December

Number of months after birth of baby

____

66. When did you stop using Injectables
Yaushe kika dakata da amfani Allurai

- ◯ January
- ◯ February
- ◯ March
- ◯ April
- ◯ May
- ◯ June
- ◯ July
- ◯ August
- ◯ September
- ◯ October
- ◯ November
- ◯ December

Number of months after birth of baby

____

67. Why did you stop using Injectables
Me yasa kika daina amfani da Allurai

- ☐ Infrequent sex/husband or partner away - Rashin saduwa/maigida baya gari
- ☐ Became pregnant while using - Samun juna lokacin da kae amfani da hutun haihuwa
- ☐ Wanted to become pregnant - Ina so in sami juna biyu
- ☐ Husband/partner opposed - Mijinki bai yarda ba
- ☐ Others opposed - Wasu basu yarda ba
- ☐ Wanted more effective method - Ina son ingantaccien hutun haihuwa
- ☐ Side effects/health concerns - Laruran maganin na daban/damuwa da lafiya
- ☐ Lack of access/too far - Rashin samu/waje yayi nisa
- ☐ Costs too much - Yayi tsada da yawa
- ☐ Inconvenient to use - Banji dadin amfani da shi ba
- ☐ Up to God/fatalistic - Yadda Allah ya so/Gaddara
- ☐ Difficult to get pregnant/menopausal - Da wuaya na samu juna biyu/Na daina haihuwa
- ☐ Marital dissolution/separation - Rabuwa da maigida
- ☐ Other - Wani

Other specify

____

68. From where did you obtain Injectables
Daga ina kika samu Allurai

- ○ The same facility where I received antenatal care (ANC)
- ○ A different public sector health centre, doctor, or clinic from where you I antenatal care (ANC) - Asibiti, Likita or dakin magani na sha ka tafi
- ○ Private or faith based sector health centre, doctor, or clinic - Wurin Sirri ko wurin addini, likita, or dakin magani na sha ka tafi
- ○ Pharmacy - Babbab dakin shan magani na kudi
- ○ PMS/Chemist - Dakin sayar da magani/kantin magani
- ○ Community health worker - Ma'aikatan lafiya na unguwa
- ○ Other - Wani

Other specify

____

69. What factors contributed to your decision to use Injectables
Wane dalili ne ya taimaka miki wajen yanke shawara yin amfani da shi Allurai

- [ ] Convenience of obtaining method - Naji dadin amfanin da shiConvenience of obtaining method
- [ ] Easy/convenient to use - Saukin amfani
- [ ] Availability - Akwai ako ina
- [ ] Cost is not too much/free - Ba tsada/kyauta
- [ ] Preferred method selected during pregnancy - Zabi na lokacin da nake da ciki
- [ ] Considered the most effective method - Ganin hanyar tana da inganci
- [ ] Unlikely to be bothered by side effects - Ba lallai in damu da larurar maganin ta daban
- [ ] Felt method was safe/few health concerns - Inajin hanyar bata da matsala/Ina jin kyan wannan hanyar/akwai darurar kan lafiya
- [ ] Health provider influenced choice - Zabin maakaciyar asibiti
- [ ] Friends/family influenced choice - Zabin abokai/'yan'uwa
- [ ] Husband influenced choice - Zabin miji
- [ ] Women in my ANC/PNC group influenced my choice - Zabin mata a lokacin awon cikina/taron bayan haihuwa
- [ ] Other - Wani

Other specify

____

Implants

65. When did you begin using Implants
Yaushe kika fara amfani Dashen hannu

- ○ January
- ○ February
- ○ March
- ○ April
- ○ May
- ○ June
- ○ July
- ○ August
- ○ September
- ○ October
- ○ November
- ○ December

Number of months after birth of baby

____

66. When did you stop using Implants
Yaushe kika dakata da amfani Dashen hannu

- ○ January
- ○ February
- ○ March
- ○ April
- ○ May
- ○ June
- ○ July
- ○ August
- ○ September
- ○ October
- ○ November
- ○ December

Number of months after birth of baby

____

67. Why did you stop using Implants
Me yasa kika daina amfani da Dashen hannu

- ☐ Infrequent sex/husband or partner away - Rashin saduwa/maigida baya gari
- ☐ Became pregnant while using - Samun juna lokacin da kae amfani da hutun haihuwa
- ☐ Wanted to become pregnant - Ina so in sami juna biyu
- ☐ Husband/partner opposed - Mijinki bai yarda ba
- ☐ Others opposed - Wasu basu yarda ba
- ☐ Wanted more effective method - Ina son ingantaccien hutun haihuwa
- ☐ Side effects/health concerns - Laruran maganin na daban/damuwa da lafiya
- ☐ Lack of access/too far - Rashin samu/waje yayi nisa
- ☐ Costs too much - Yayi tsada da yawa
- ☐ Inconvenient to use - Banji dadin amfani da shi ba
- ☐ Up to God/fatalistic - Yadda Allah ya so/Gaddara
- ☐ Difficult to get pregnant/menopausal - Da wuaya na samu juna biyu/Na daina haihuwa
- ☐ Marital dissolution/separation - Rabuwa da maigida
- ☐ Other - Wani

Other specify

____

68. From where did you obtain Implants
Daga ina kika samu Dashen hannu

- ◯ The same facility where I received antenatal care (ANC)
- ◯ A different public sector health centre, doctor, or clinic from where you I antenatal care (ANC) - Asibiti, Likita or dakin magani na sha ka tafi
- ◯ Private or faith based sector health centre, doctor, or clinic - Wurin Sirri ko wurin addini, likita, or dakin magani na sha ka tafi
- ◯ Pharmacy - Babbab dakin shan magani na kudi
- ◯ PMS/Chemist - Dakin sayar da magani/kantin magani
- ◯ Community health worker - Ma'aikatan lafiya na unguwa
- ◯ Other - Wani

Other specify

____

69. What factors contributed to your decision to use Implants
Wane dalili ne ya taimaka miki wajen yanke shawara yin amfani da shi Dashen hannu

- ☐ Convenience of obtaining method - Naji dadin amfanin da shiConvenience of obtaining method
- ☐ Easy/convenient to use - Saukin amfani
- ☐ Availability - Akwai ako ina
- ☐ Cost is not too much/free - Ba tsada/kyauta
- ☐ Preferred method selected during pregnancy - Zabi na lokacin da nake da ciki
- ☐ Considered the most effective method - Ganin hanyar tana da inganci
- ☐ Unlikely to be bothered by side effects - Ba lallai in damu da larurar maganin ta daban
- ☐ Felt method was safe/few health concerns - Inajin hanyar bata da matsala/Ina jin kyan wannan hanyar/akwai darurar kan lafiya
- ☐ Health provider influenced choice - Zabin maakaciyar asibiti
- ☐ Friends/family influenced choice - Zabin abokai/'yan'uwa
- ☐ Husband influenced choice - Zabin miji
- ☐ Women in my ANC/PNC group influenced my choice - Zabin mata a lokacin awon cikina/taron bayan haihuwa
- ☐ Other - Wani

Other specify ______________________________

IUD

65. When did you begin using IUD
Yaushe kika fara amfani Robar mahaifa

- ◯ January
- ◯ February
- ◯ March
- ◯ April
- ◯ May
- ◯ June
- ◯ July
- ◯ August
- ◯ September
- ◯ October
- ◯ November
- ◯ December

Number of months after birth of baby ______________________________

66. When did you stop using IUD
Yaushe kika dakata da amfani Robar mahaifa

- ◯ January
- ◯ February
- ◯ March
- ◯ April
- ◯ May
- ◯ June
- ◯ July
- ◯ August
- ◯ September
- ◯ October
- ◯ November
- ◯ December

Number of months after birth of baby ______________________________

67. Why did you stop using IUD
Me yasa kika daina amfani da Robar mahaifa

- ☐ Infrequent sex/husband or partner away - Rashin saduwa/maigida baya gari
- ☐ Became pregnant while using - Samun juna lokacin da kae amfani da hutun haihuwa
- ☐ Wanted to become pregnant - Ina so in sami juna biyu
- ☐ Husband/partner opposed - Mijinki bai yarda ba
- ☐ Others opposed - Wasu basu yarda ba
- ☐ Wanted more effective method - Ina son ingantaccien hutun haihuwa
- ☐ Side effects/health concerns - Laruran maganin na daban/damuwa da lafiya
- ☐ Lack of access/too far - Rashin samu/waje yayi nisa
- ☐ Costs too much - Yayi tsada da yawa
- ☐ Inconvenient to use - Banji dadin amfani da shi ba
- ☐ Up to God/fatalistic - Yadda Allah ya so/Gaddara
- ☐ Difficult to get pregnant/menopausal - Da wuaya na samu juna biyu/Na daina haihuwa
- ☐ Marital dissolution/separation - Rabuwa da maigida
- ☐ Other - Wani

Other specify

____

68. From where did you obtain IUD
Daga ina kika samu Robar mahaifa

- ○ The same facility where I received antenatal care (ANC)
- ○ A different public sector health centre, doctor, or clinic from where you I antenatal care (ANC) - Asibiti, Likita or dakin magani na sha ka tafi
- ○ Private or faith based sector health centre, doctor, or clinic - Wurin Sirri ko wurin addini, likita, or dakin magani na sha ka tafi
- ○ Pharmacy - Babbab dakin shan magani na kudi
- ○ PMS/Chemist - Dakin sayar da magani/kantin magani
- ○ Community health worker - Ma'aikatan lafiya na unguwa
- ○ Other - Wani

Other specify

____

69. What factors contributed to your decision to use IUD
Wane dalili ne ya taimaka miki wajen yanke shawara yin amfani da shi Robar mahaifa

- ☐ Convenience of obtaining method - Naji dadin amfanin da shiConvenience of obtaining method
- ☐ Easy/convenient to use - Saukin amfani
- ☐ Availability - Akwai ako ina
- ☐ Cost is not too much/free - Ba tsada/kyauta
- ☐ Preferred method selected during pregnancy - Zabi na lokacin da nake da ciki
- ☐ Considered the most effective method - Ganin hanyar tana da inganci
- ☐ Unlikely to be bothered by side effects - Ba lallai in damu da larurar maganin ta daban
- ☐ Felt method was safe/few health concerns - Inajin hanyar bata da matsala/Ina jin kyan wannan hanyar/akwai darurar kan lafiya
- ☐ Health provider influenced choice - Zabin maakaciyar asibiti
- ☐ Friends/family influenced choice - Zabin abokai/'yan'uwa
- ☐ Husband influenced choice - Zabin miji
- ☐ Women in my ANC/PNC group influenced my choice - Zabin mata a lokacin awon cikina/taron bayan haihuwa
- ☐ Other - Wani

Other specify

____

Emergency Contraception Pill

65. When did you begin using Emergency pill
Yaushe kika fara amfani Kwayoyin sha na gaggawa

- ◯ January
- ◯ February
- ◯ March
- ◯ April
- ◯ May
- ◯ June
- ◯ July
- ◯ August
- ◯ September
- ◯ October
- ◯ November
- ◯ December

Number of months after birth of baby

____

66. When did you stop using Emergency pill
Yaushe kika dakata da amfani Kwayoyin sha na gaggawa

- ◯ January
- ◯ February
- ◯ March
- ◯ April
- ◯ May
- ◯ June
- ◯ July
- ◯ August
- ◯ September
- ◯ October
- ◯ November
- ◯ December

Number of months after birth of baby

____

67. Why did you stop using Emergency pill
Me yasa kika daina amfani da Kwayoyin sha na gaggawa

- ☐ Infrequent sex/husband or partner away - Rashin saduwa/maigida baya gari
- ☐ Became pregnant while using - Samun juna lokacin da kae amfani da hutun haihuwa
- ☐ Wanted to become pregnant - Ina so in sami juna biyu
- ☐ Husband/partner opposed - Mijinki bai yarda ba
- ☐ Others opposed - Wasu basu yarda ba
- ☐ Wanted more effective method - Ina son ingantaccien hutun haihuwa
- ☐ Side effects/health concerns - Laruran maganin na daban/damuwa da lafiya
- ☐ Lack of access/too far - Rashin samu/waje yayi nisa
- ☐ Costs too much - Yayi tsada da yawa
- ☐ Inconvenient to use - Banji dadin amfani da shi ba
- ☐ Up to God/fatalistic - Yadda Allah ya so/Gaddara
- ☐ Difficult to get pregnant/menopausal - Da wuaya na samu juna biyu/Na daina haihuwa
- ☐ Marital dissolution/separation - Rabuwa da maigida
- ☐ Other - Wani

Other specify

____

68. From where did you obtain Emergency pill
Daga ina kika samu Kwayoyin sha na gaggawa

- ◯ The same facility where I received antenatal care (ANC)
- ◯ A different public sector health centre, doctor, or clinic from where you I antenatal care (ANC) - Asibiti, Likita or dakin magani na sha ka tafi
- ◯ Private or faith based sector health centre, doctor, or clinic - Wurin Sirri ko wurin addini, likita, or dakin magani na sha ka tafi
- ◯ Pharmacy - Babbab dakin shan magani na kudi
- ◯ PMS/Chemist - Dakin sayar da magani/kantin magani
- ◯ Community health worker - Ma'aikatan lafiya na unguwa
- ◯ Other - Wani

Other specify

____

69. What factors contributed to your decision to use Emergency pill
Wane dalili ne ya taimaka miki wajen yanke shawara yin amfani da shi Kwayoyin sha na gaggawa

- ☐ Convenience of obtaining method - Naji dadin amfanin da shiConvenience of obtaining method
- ☐ Easy/convenient to use - Saukin amfani
- ☐ Availability - Akwai ako ina
- ☐ Cost is not too much/free - Ba tsada/kyauta
- ☐ Preferred method selected during pregnancy - Zabi na lokacin da nake da ciki
- ☐ Considered the most effective method - Ganin hanyar tana da inganci
- ☐ Unlikely to be bothered by side effects - Ba lallai in damu da larurar maganin ta daban
- ☐ Felt method was safe/few health concerns - Inajin hanyar bata da matsala/Ina jin kyan wannan hanyar/akwai darurar kan lafiya
- ☐ Health provider influenced choice - Zabin maakaciyar asibiti
- ☐ Friends/family influenced choice - Zabin abokai/'yan'uwa
- ☐ Husband influenced choice - Zabin miji
- ☐ Women in my ANC/PNC group influenced my choice - Zabin mata a lokacin awon cikina/taron bayan haihuwa
- ☐ Other - Wani

Other specify

____

Sterilisation

65. When did you begin using Sterilisation
Yaushe kika fara amfani Dandatsa

- ○ January
- ○ February
- ○ March
- ○ April
- ○ May
- ○ June
- ○ July
- ○ August
- ○ September
- ○ October
- ○ November
- ○ December

Number of months after birth of baby

____

68. From where did you obtain Sterilisation
Daga ina kika samu Dandatsa

- ○ The same facility where I received antenatal care (ANC)
- ○ A different public sector health centre, doctor, or clinic from where you I antenatal care (ANC) - Asibiti, Likita or dakin magani na sha ka tafi
- ○ Private or faith based sector health centre, doctor, or clinic - Wurin Sirri ko wurin addini, likita, or dakin magani na sha ka tafi
- ○ Pharmacy - Babbab dakin shan magani na kudi
- ○ PMS/Chemist - Dakin sayar da magani/kantin magani
- ○ Community health worker - Ma'aikatan lafiya na unguwa
- ○ Other - Wani

Other specify

____

69. What factors contributed to your decision to use Sterilisation
Wane dalili ne ya taimaka miki wajen yanke shawara yin amfani da shi Dandatsa

- ☐ Convenience of obtaining method - Naji dadin amfanin da shiConvenience of obtaining method
- ☐ Easy/convenient to use - Saukin amfani
- ☐ Availability - Akwai ako ina
- ☐ Cost is not too much/free - Ba tsada/kyauta
- ☐ Preferred method selected during pregnancy - Zabi na lokacin da nake da ciki
- ☐ Considered the most effective method - Ganin hanyar tana da inganci
- ☐ Unlikely to be bothered by side effects - Ba lallai in damu da larurar maganin ta daban
- ☐ Felt method was safe/few health concerns - Inajin hanyar bata da matsala/Ina jin kyan wannan hanyar/akwai darurar kan lafiya
- ☐ Health provider influenced choice - Zabin maakaciyar asibiti
- ☐ Friends/family influenced choice - Zabin abokai/'yan'uwa
- ☐ Husband influenced choice - Zabin miji
- ☐ Women in my ANC/PNC group influenced my choice - Zabin mata a lokacin awon cikina/taron bayan haihuwa
- ☐ Other - Wani

Other specify

____

Other FP ([pnc_q64_other])

65. When did you begin using [pnc_q64_other]
Yaushe kika fara amfani [pnc_q64_other]

- ○ January
- ○ February
- ○ March
- ○ April
- ○ May
- ○ June
- ○ July
- ○ August
- ○ September
- ○ October
- ○ November
- ○ December

Number of months after birth of baby

____

66. When did you stop using [pnc_q64_other]
Yaushe kika dakata da amfani [pnc_q64_other]

- ○ January
- ○ February
- ○ March
- ○ April
- ○ May
- ○ June
- ○ July
- ○ August
- ○ September
- ○ October
- ○ November
- ○ December

Number of months after birth of baby

____

67. Why did you stop using [pnc_q64_other]
Me yasa kika daina amfani da [pnc_q64_other]

- ☐ Infrequent sex/husband or partner away - Rashin saduwa/maigida baya gari
- ☐ Became pregnant while using - Samun juna lokacin da kae amfani da hutun haihuwa
- ☐ Wanted to become pregnant - Ina so in sami juna biyu
- ☐ Husband/partner opposed - Mijinki bai yarda ba
- ☐ Others opposed - Wasu basu yarda ba
- ☐ Wanted more effective method - Ina son ingantaccien hutun haihuwa
- ☐ Side effects/health concerns - Laruran maganin na daban/damuwa da lafiya
- ☐ Lack of access/too far - Rashin samu/waje yayi nisa
- ☐ Costs too much - Yayi tsada da yawa
- ☐ Inconvenient to use - Banji dadin amfani da shi ba
- ☐ Up to God/fatalistic - Yadda Allah ya so/Gaddara
- ☐ Difficult to get pregnant/menopausal - Da wuaya na samu juna biyu/Na daina haihuwa
- ☐ Marital dissolution/separation - Rabuwa da maigida
- ☐ Other - Wani

Other specify

____

68. From where did you obtain [pnc_q64_other]
Daga ina kika samu [pnc_q64_other]

- ◯ The same facility where I received antenatal care (ANC)
- ◯ A different public sector health centre, doctor, or clinic from where you I antenatal care (ANC) - Asibiti, Likita or dakin magani na sha ka tafi
- ◯ Private or faith based sector health centre, doctor, or clinic - Wurin Sirri ko wurin addini, likita, or dakin magani na sha ka tafi
- ◯ Pharmacy - Babbab dakin shan magani na kudi
- ◯ PMS/Chemist - Dakin sayar da magani/kantin magani
- ◯ Community health worker - Ma'aikatan lafiya na unguwa
- ◯ Other - Wani

Other specify

____

69. What factors contributed to your decision to use [pnc_q64_other]
Wane dalili ne ya taimaka miki wajen yanke shawara yin amfani da shi [pnc_q64_other]

- ☐ Convenience of obtaining method - Naji dadin amfanin da shiConvenience of obtaining method
- ☐ Easy/convenient to use - Saukin amfani
- ☐ Availability - Akwai ako ina
- ☐ Cost is not too much/free - Ba tsada/kyauta
- ☐ Preferred method selected during pregnancy - Zabi na lokacin da nake da ciki
- ☐ Considered the most effective method - Ganin hanyar tana da inganci
- ☐ Unlikely to be bothered by side effects - Ba lallai in damu da larurar maganin ta daban
- ☐ Felt method was safe/few health concerns - Inajin hanyar bata da matsala/Ina jin kyan wannan hanyar/akwai darurar kan lafiya
- ☐ Health provider influenced choice - Zabin maakaciyar asibiti
- ☐ Friends/family influenced choice - Zabin abokai/'yan'uwa
- ☐ Husband influenced choice - Zabin miji
- ☐ Women in my ANC/PNC group influenced my choice - Zabin mata a lokacin awon cikina/taron bayan haihuwa
- ☐ Other - Wani

Other specify ______________________________

ADAPTED CONTRACEPTIVE SELF-EFFICACY SCALE

71. This set of questions is designed to help us get a better understanding of the kinds of things that are difficult for women who want family planning services. Please rate how certain you are that you can do each of the things described by using this scale
Wannan tambayoyin an an tsara sune su taimake mu Wannan jerin tambayoyin zasu taimaka wajen ganiya akan ire-irn abubuwan da suka yi wa matan da suke son yin amfani da hanyoyin hutun haihuwa tsauri. of things that are difficult for women who want family planning services. Ina son ki auna tabbatar da wadannan bayanai ta yin amfani da ma'auni

71a. Discuss family size with my husband/partner
Tattauna yawan iyalin da nake so tare da mijina na ko abokin zama

○ 0 ○ 1 ○ 2 ○ 3 ○ 4 ○ 5 ○ 6 ○ 7 ○ 8 ○ 9 ○ 10

71b. Discuss if and when I'd like to get pregnant again with my husband/partner
Tattaunawa da mijina akan yaushe zan sake samun juna biyu

○ 0 ○ 1 ○ 2 ○ 3 ○ 4 ○ 5 ○ 6 ○ 7 ○ 8 ○ 9 ○ 10

71c. Discuss specific family planning methods with my husband/partner.
Tattauna akan wata daya daga cikin hanyoyin hutun haihuwa.

○ 0 ○ 1 ○ 2 ○ 3 ○ 4 ○ 5 ○ 6 ○ 7 ○ 8 ○ 9 ○ 10

71d. Fully express my opinions about family size and family planning methods with my husband/partner
Bayyana ra'ayina game da yawan iyali da kuma hanyar hutun haihuwa

○ 0 ○ 1 ○ 2 ○ 3 ○ 4 ○ 5 ○ 6 ○ 7 ○ 8 ○ 9 ○ 10

71e. Ask my husband/partner to use a condom if I want him to
Nunawa mijina bukar amfani da kororon roba idan ina son haka

○ 0 ○ 1 ○ 2 ○ 3 ○ 4 ○ 5 ○ 6 ○ 7 ○ 8 ○ 9 ○ 10

71f. Reach an agreement with my husband/partner about use of family planning that takes my desires into account
Kaiwa matsaya tare da mijina/abokin zama dangane da hanyar hutun haihuwa day a ja ra'ayina.

○ 0 ○ 1 ○ 2 ○ 3 ○ 4 ○ 5 ○ 6 ○ 7 ○ 8 ○ 9 ○ 10

71g. Choose a family size, time between births, or family planning method that my mother in law does not agree with

Zabi yawan iyali, tazara tsakanin haihuwa, ko hanyar hutun haihuwa wanda uwar mijina ba ta amince dashi ba

○ 0 ○ 1 ○ 2 ○ 3 ○ 4 ○ 5 ○ 6 ○ 7 ○ 8 ○ 9 ○ 10

71h. Start a family planning method if my friends and family might find out
Fara amfani da hanyar hutun haihuwa idan 'yan uwa da abokai zasu gano

○ 0 ○ 1 ○ 2 ○ 3 ○ 4 ○ 5 ○ 6 ○ 7 ○ 8 ○ 9 ○ 10

71i. Continue a family planning method if my friends and family found out
Ci gaba da amfani da hanyar hutun haihuwa idan 'yan uwa da abokai suka gano

○ 0 ○ 1 ○ 2 ○ 3 ○ 4 ○ 5 ○ 6 ○ 7 ○ 8 ○ 9 ○ 10

71j. Bring up the topic of family planning with a health care provider
Tattaunawa akan hutun haihuwa tare da maaikaciyar lafiya

○ 0 ○ 1 ○ 2 ○ 3 ○ 4 ○ 5 ○ 6 ○ 7 ○ 8 ○ 9 ○ 10

71k. Ask a provider questions I have about family planning methods
Tambayar ma'aikaciyar lafiya akan tambayoyi da naske da su

○ 0 ○ 1 ○ 2 ○ 3 ○ 4 ○ 5 ○ 6 ○ 7 ○ 8 ○ 9 ○ 10

71l. Ask a provider if I'm not sure something I've heard about family planning is true or not, such as a potential side effect I have heard from other women
Zan tambayi maaikaciyar lafiya a akan abin da nake shakku akan wanda naji a wajen mata game da hutun haihuwa

◯ 0 ◯ 1 ◯ 2 ◯ 3 ◯ 4 ◯ 5 ◯ 6 ◯ 7 ◯ 8 ◯ 9 ◯ 10

71m. Ask a provider to clarify something they have told me about family planning if I'm not sure I understand
Tambayi maaikaciyar lafiya to wayar mini da kai akan ain da naji akan hutun haihuwa in ban tabbatar ba ko ban gane ba

◯ 0 ◯ 1 ◯ 2 ◯ 3 ◯ 4 ◯ 5 ◯ 6 ◯ 7 ◯ 8 ◯ 9 ◯ 10

71n. Tell a provider what's important to me in choosing a family planning method
Fadawa ma'aikaciyar lafiyar abin da ya ke da mahummaci dani a zabin bina na hutun haihuwa

◯ 0 ◯ 1 ◯ 2 ◯ 3 ◯ 4 ◯ 5 ◯ 6 ◯ 7 ◯ 8 ◯ 9 ◯ 10

71o. Have some control over if and when I get pregnant again
Samun cikakken iko kan samun juna biyu

◯ 0 ◯ 1 ◯ 2 ◯ 3 ◯ 4 ◯ 5 ◯ 6 ◯ 7 ◯ 8 ◯ 9 ◯ 10

71p. Choose a family planning method that will work well for me
Zabin hutun haihuwa da zaiyi mini aiki da kyau

◯ 0 ◯ 1 ◯ 2 ◯ 3 ◯ 4 ◯ 5 ◯ 6 ◯ 7 ◯ 8 ◯ 9 ◯ 10

71q. Obtain the method of family planning I want, if I want one
Karbar hutun haihuwa dana keso inda akwai

◯ 0 ◯ 1 ◯ 2 ◯ 3 ◯ 4 ◯ 5 ◯ 6 ◯ 7 ◯ 8 ◯ 9 ◯ 10

71r. Obtain a different method of family planning if the one I want isn't available
Karbar wani hutun haihuwa indan wanda nake so babu

◯ 0 ◯ 1 ◯ 2 ◯ 3 ◯ 4 ◯ 5 ◯ 6 ◯ 7 ◯ 8 ◯ 9 ◯ 10

71s. Find solutions to bothersome side effects from family planning or switch methods if needed because of bothersome side effects
Neman mafita ka matsalar hanyar hutun haihuwa ta daban Ko kuma canja hanyar hutun haihuwa don gudun matsalar hanyar hutun haihuwa ta daban

◯ 0 ◯ 1 ◯ 2 ◯ 3 ◯ 4 ◯ 5 ◯ 6 ◯ 7 ◯ 8 ◯ 9 ◯ 10

71t. Use a family planning method according to instructions to prevent pregnancy
Amfani da hutun haihuwa yadda aka nuna mini na kare samun junu baiyu

◯ 0 ◯ 1 ◯ 2 ◯ 3 ◯ 4 ◯ 5 ◯ 6 ◯ 7 ◯ 8 ◯ 9 ◯ 10

71u. Stop using family planning and get pregnant again if/when I want to
Dakatar da hutun huihuwa in ina so nsa samu juna biyu in ina so

◯ 0 ◯ 1 ◯ 2 ◯ 3 ◯ 4 ◯ 5 ◯ 6 ◯ 7 ◯ 8 ◯ 9 ◯ 10
