# Supplementary material for: Group versus individual antenatal and first year postpartum care: Study protocol for a multi-country cluster randomized controlled trial in Kenya and Nigeria
Source: Gates Open Res. 2019 Jul 4;2:56. Originally published 2018 Nov 5. [Version 2] doi: 10.12688/gatesopenres.12867.2 (PMC6350506; doi:10.12688/gatesopenres.12867.2)
Supplement: Supplementary file 1 [file gatesopenres-2-14160-s0000.tgz › 72242879-e244-48c8-a71c-b86050fb7704_Supplemental_files-_Study_manual,_Data_tools,_and_recruitment_,materials.zip!/Supplemental files- Study manual, Data tools, and recruitment ,materials/Screening and Consent Form_GroupA.pdf]

Screening and Consent Form

PARTICIPANT STUDY ID	__________________
Date and Time of record creation - Data Quality Check	__________________
Screening and Consent Survey - Latitude	__________________
Screening and Consent Survey - Longitude	__________________
Name of data collector - Data quality check	__________________
Facility Name	◯ PHC Nasarawa Eggon<br>◯ PHC New Karu<br>◯ PHC Tsonhon Kasuwa<br>◯ PHC Karshi I<br>◯ PHC Agyaragu Yakubu<br>◯ MPHC Bakyano<br>◯ PHC Akruba<br>◯ PHC Masaka<br>◯ PHC Lafia East<br>◯ PHC Doma Road, Lafia<br>◯ PHC I Garaku<br>◯ PHC Barkin Abdullahi (B.A.D)<br>◯ PHC Tundungwadara<br>◯ GH Akwanga<br>◯ Medical Centre, Mararaba<br>◯ PHC Mararaba<br>◯ PHC Kofar Pada<br>◯ PHC Mana, Emir's Palace, Lafia<br>◯ PHC Angwan Waje<br>◯ PHC Azara
LGA	__________________<br>(4 - Nasarwa (LGA))
Intervention or control facility	__________________<br>(1 - Intervention site; 2 - Control site)
Facility code	__________________
Date of data collection/interview	__________________<br>(DD/MM/YYYY)
Gestational age of client today - IN WEEKS	__________________

Gestational age of client today - IN DAYS

○ 0 ○ 1 ○ 2 ○ 3 ○ 4 ○ 5 ○ 6

Gestational age on the day of data collection

____
(This is a calculated field. You need to input the GA weeks and GA days)

GA determined by

[Wacce hanya aka gano watannin ciki]

☐ LMP [Baton wata]
☐ Ultrasound [Hoto]
☐ Fundal height [Tsawo/tashin ciki]
☐ Pelvic exam [Binciken kunkuru]
(Check all that apply)

When is your EDD (Expected Date of Delivery)?

____

Client eligible by Gestational Age?

____
(1 - Client eligible by GA i.e. GA is < =24 weeks for both Control and Intervention sites; 2 - Client ineligible by GA. Client to proceed with routine ANC)

Able to speak and understand any of the following languages

☐ English
☐ Hausa

IF CLIENT AGREES TO SCREENING, READ CLIENT STUDY INFORMATION SHEET. REQUEST PERMISSION TO CONTINUE SCREENING

Client agreed to be screened?

○ Agreed to screening [Ta yarda a tantance ta]
○ Declined screening [Ba ta yarda a tantance ta ba]

Inclusion Criteria

Age at last birthday

[Shekarun haifuwa a jego na karshe?]

____
(Should be greater than or equal to 15 years old.)

Are you interested in participating, including follow up for up to 12 months after you deliver your baby?

[Kina da ra'ayin ki kasance cikin wannan binciken, tare da kulawa ta wata goma sha biyu bayan haifuwa?]

○ Yes [Eh]
○ No [A'a]

Are you planning to stay in this area for the next 1 and a half years and continue your antenatal care at this facility?

[Akwai yiwuwar kasancewarki a wannan unguwar har zuwa shekara daya da rabi nan gaba sannan za ki ci gaba da awon cikinki a wannan Cibiyar?]

○ Yes [Eh]
○ No [A'a]

Are you planning to be away from home (near this facility) for more than 4 weeks in a row at any time during your pregnancy or more than 3 months in a row in the year after you have your baby?<br><br>[Akwai yiwuwar yin wata tafiya ta fiye da bakwai hudu nan gaba kafin haifuwa ko tafiyar fiye da wata ukku cikin shekara daya bayan haifuwa?]	◯ Yes [Eh]<br>◯ No [A'a]
Able and willing to provide phone number where we can reach you to remind you of upcoming ANC visits and/or to set up appointments for data collection?<br><br>[Za ki iya ba mu lambar wayar da yardarki domin mu kira ki mu tuna maki da lokacin awo na gaba ko kuma mu shirya wata haduwa domin tattara wasu bayanai ?[Nuna ko sunfi son kira (K) ko sakon kar ta kwana (S)]]	◯ Phone (P)<br>◯ Text/SMS (T)<br>◯ None
Meets all inclusion criteria?<br><br>[An cika dukkan Ka'idojin shiga?]	__________________________________<br>(1 - Meets all inclusion criteria; 2 - Does not meet all inclusion criteria)

Basic Demographic Questions

What is the highest level of education you completed?<br><br>[Wane matakin ilimi kika cimma?]	◯ No school [Ba karatun komi]<br>◯ Primary [Matakin faramare]<br>◯ Qu'aranic education only [Karatun allo kawai]<br>◯ Secondary [Matakin sakandare]<br>◯ Post Secondary [Matakin gaba da sakandare]
Are you able to read and write<br><br>[Kina iya karatu da rubutu?]	◯ Yes [Eh]<br>◯ No [A'a]
What is your religion?<br><br>[Wane addini ki ke yi?]	◯ Catholic [Katolika]<br>◯ Islam [Musulunci]<br>◯ Other Christian [Wata darikar kiristanci]<br>◯ Traditionalist [Addinin gargajiya]<br>◯ Other [Wasu addinai]
Other religion specify<br><br>[Wasu addinai]	__________________________________
How do you usually get here, to the health facility? What form of transportation do you use?<br><br>[Yaya kike zuwa nan asibitin (wurin awo) ? Wace hanyar sufuri kike anfani da ita?]	◯ Walk [Tafiyar kasa]<br>◯ Public transport [Hanyar sufurin kowa da kowa]<br>◯ Personal [Abin hawa na gida]<br>◯ Other [Wasu addinai]
Other transport mode specify<br><br>[Wace hanyar kuma]	__________________________________

How long does it take you to get here using the mode of transportation?<br><br>[Wane lokaci kike dauka kafin ki iso wurin awo ta amfani da hanyar da kika bayyana a sama?]	__________________________________<br>(The time should be in minutes)
What is your current marital status?<br><br>[Mene ne matsayin aurenki a yanzu?]	◯ Married [Mai aure]<br>◯ Single, not in union [Ba aure, ba zaman tare]<br>◯ Cohabiting [Zaman tare]<br>◯ Separated/divorced [Zawara (miji ya saki)]<br>◯ Widowed [Gwamruwa (miji ya mutu)]<br>◯ Never married [Ba'a taba aure ba]
How many times have you given birth before?<br><br>[Sau nawa kika taba haifuwa?]	__________________________________<br>(Regardless of outcome)
Do you participate in any other groups at this facility, where you meet with the same people on a regular basis?<br><br>[Ko kin taba kasancewa cikin wata kungiya a wannan cibiyar, inda kuka hadu da wadannan mutane bisa wasu kayyadadun lokutta ?]	◯ Yes [Eh]<br>◯ No [A'a]
Date of first meeting for the G-ANC cohort<br><br>[Ranar haduwar farko ta kungiya 1]	__________________________________
Time of first meeting for the G-ANC cohort	__________________________________
Gestational Age of client at next group visit 1<br><br>[Watannin cikin mai awo a haduwa ta gaba]	__________________________________<br>(This is a calculated field. You need to input the following: [GA weeks], [GA days], [Interview date], and [Date of scheduled group cohort meeting])

CLIENT WILL NOT BE ELIGIBLE FOR THE COHORT AS HER GESTATIONAL AGE WILL BE [intervention_cohort_ga] WEEKS ON [group_visit_date].

THIS IS BELOW 20 WEEKS WHICH IS REQUIRED TO BE PART OF THIS COHORT

PLEASE ADD HER TO THE NEXT AVAILABLE COHORT TO SEE IF SHE WILL BE ELIGIBLE.

CLIENT WILL NOT BE ELIGIBLE FOR THE COHORT AS HER GESTATIONAL AGE WILL BE [intervention_cohort_ga] WEEKS ON [group_visit_date].

THIS IS OVER 24 WEEKS HENCE SHE CANNOT BE PART OF THIS COHORT

CHECK TO SEE IF SHE CAN BE PART OF AN EARLIER COHORT, OTHERWISE TELL THEM THEY ARE NOT ELIGIBLE FOR THE STUDY AND SHOULD RETURN FOR THEIR NEXT NORMALLY SCHEDULED ANC VISIT AS DESCRIBED BY THEIR PROVIDER.

Will you be able to come back for the first group visit on [group_visit_date] at [cohort_time]<br><br>Ko za ki iya dawowa ranar: [group_visit_date], [cohort_time]	◯ Yes/Probably [Eh / wata kila]<br>◯ Unsure [Babu tabbas]<br>◯ No/probably not [A'a/Wata kila a'a]<br>(If Yes/probably/unsure, place client in cohort; If No/probably not, thank client. Tell them they are not eligible for the study and should return for their next normally scheduled ANC visit as described with their provider.)
Group cohort number<br><br>[Kungiya #]	________________________
Final check to make sure client is eligible for study	________________________
Consent	◯ Agree to participate<br>◯ Did not agree<br>(YOU MUST SELECT WHETHER THE PARTICIPANT AGREED T BE PART OF THE STUDY. THIS IS IMPORTANT FOR BASELINE AND FOLLOW UP QUESTIONS)

Consent Log

First and Last Name of New ANC client	________________________<br>(Enter letters only. Do not enter numbers or special characters for the name)
Name usually addressed by	________________________
Primary phone number	________________________
Secondary phone number	________________________
Address/description of residence	______________________________
