# Supplementary material for: Group versus individual antenatal and first year postpartum care: Study protocol for a multi-country cluster randomized controlled trial in Kenya and Nigeria
Source: Gates Open Res. 2019 Jul 4;2:56. Originally published 2018 Nov 5. [Version 2] doi: 10.12688/gatesopenres.12867.2 (PMC6350506; doi:10.12688/gatesopenres.12867.2)
Supplement: Supplementary file 1 [file gatesopenres-2-14160-s0000.tgz › 72242879-e244-48c8-a71c-b86050fb7704_Supplemental_files-_Study_manual,_Data_tools,_and_recruitment_,materials.zip!/Supplemental files- Study manual, Data tools, and recruitment ,materials/Study Information sheet.pdf]

Group ANC Study Information Sheet:

Study Title: Cluster randomized control trial to evaluate Group Antenatal Care (G-ANC) compared with standard individual care in Kenya and Nigeria

JHSPH PI: Jeff Smith
In-Country PI (Kenya): Anthony Gichangi
In-Country PI (Nigeria): Emmanuel Ugwa

General Study description: Our facility is taking part in a research study supported by the Ministry of Health evaluating group antenatal care with standard individual care. Group antenatal care models have been implemented in different settings as one way of improving care, healthy outcomes, satisfaction, and increased use of other important health services such as facility birth, family planning and immediate post natal care – the results are encouraging but there is limited experience and evidence for providing care this way in Africa. There is much interest in the potential benefits of group care for women, their infants, and providers. We have designed a study to evaluate the effectiveness, acceptability, and feasibility of group ANC compared to traditional, individual ANC in Kenya and Nigeria. Our study will also look at continuing group care through the first year after birth to determine it's full range of benefits to the mother and baby during the first year of life. The results will help guide how care is delivered in the future.

***[Intervention group]**:* You will be given specific dates and times to come for antenatal care and spend 1-2 hours receiving care facilitated by a provider with a group of women who are at the same stage of pregnancy. You will receive care by the same provider and with the same group of women throughout pregnancy (1x month) and after delivery (every 3 months). During group visits you will receive all standard antenatal care while also having more time to learn about and discuss issues important to your health, pregnancy and the health of your baby with other mothers.

***[Control group]**:* You will receive standard, individual antenatal care. Participating in the study means that the study will follow up with you one month after delivery and when your baby is 1 year old to see how your outcomes and satisfaction with care differ from those who received care in a group. This information is very important so that the study can tell which form of care is preferable.

Questions/contact info: If you are asked to participate, we hope that you will consider it seriously and spare some time to take part in this study. If you do participate and have any concerns about the intervention or the study during your participation, you are free to contact any of the following members of the study team:

Dr. Anthony Gichangi	Dr. Emmanuel Ugwa
**(+254)714-918169**	**(+234)803-585-1872**

We thank you for this opportunity to work together to improve the way antenatal care is provided.

END

[Instructions for Service Providers]: Circle the section, intervention/control, which corresponds to this study site prior to giving sheet to client.
