# Supplementary material for: Group versus individual antenatal and first year postpartum care: Study protocol for a multi-country cluster randomized controlled trial in Kenya and Nigeria
Source: Gates Open Res. 2019 Jul 4;2:56. Originally published 2018 Nov 5. [Version 2] doi: 10.12688/gatesopenres.12867.2 (PMC6350506; doi:10.12688/gatesopenres.12867.2)
Supplement: Peer review history

# Peer review: Reviewer response for version 1

**DOI:** 10.21956/gatesopenres.13951.r26741

**Published:** 2019-01-08

**Authors:** Crystal L. Patil

**Affiliations:** 1 Women, Children and Family Health Science, College of Nursing, University of Illinois at Chicago (UIC), Chicago, IL, USA

**Summary**: This paper describes the protocol being used in a cluster randomized clinical trial (cRCT) being conducted in Kenya and Nigeria. The primary outcome associated with each phase is to assess if group antenatal care (G-ANC) is associated with 15% more health facility births (phase 1) and 10% greater use of modern family planning (phase 2) among women attending facilities where G-ANC is being offered compared to facilities offering standard ANC. Several secondary outcomes are being measured as well (e.g., service utilization, health literacy, breastfeeding behaviors, and knowledge). This team plans to qualitatively assess G-ANC as well by talking to the women and their health care providers. No results are provided as this is a protocol paper. 

**Study justification**: The background literature provided supports the rationale for conducting this cRCT in Kenya and Nigeria. 

**Design & sample**: The overall design of this cRCT is strong and sample size calculations are appropriate and justified. 

**Methods**: The data collection procedures and data quality assurance strategies are clearly outlined and the items being used to assess various outcomes are provided (see supplementary File 1). 

**Minor critiques**: 

1. Why are women without a cell phone excluded? I believe that other studies have shown greater impact of group ANC on more vulnerable populations. If cell phone ownership is a reflection of SES, this may inadvertently exclude the lowest end of the SES continuum. Minor limitation possibly.
2. Is the G-ANC model itself grounded in social/behavioral theory? Some details about the model's development and how it is building from an evidence-based could enhance this protocol. For example, in qualitative research a focus group discussion size of 8-12 is recommended, so that everyone feels they can participate. How was 15 decided upon? Another example is that the group is deciding their scheduling day/time - how will this affect scale-up? This might limit a health facility's ability implement if the women decide rather than being setup by staff.
3. The WHO is now recommending 8 ANC visits. This study proposes 5 for women in G-ANC. I am unable to assess how many ANC visits women at the control facilities are expected to attend. Are Kenya and Nigeria still recommending 4 visits? If so, why are those in G-ANC offered more visits than those in regular care? If different, how will this be accounted for in analyses?  
4. An implementation model and detailed strategies to assess fidelity are not presented. Will the intervention be offered the same way at all facilities and in both countries? Might this change over time? If there are no improvements in the two primary outcomes, will the authors be able to differentiate whether it was the intervention that had no effect or if the cause was implementation deviations (not remaining faithful to one G-ANC model throughout)? 
5. Slightly more details about the ANC and postnatal curriculum decisions would be helpful - but I suspect these will come out in a different publication. 

I look forward to seeing both the quantitative and qualitative results of this study. The study will make a major contribution to the literature and has policy implications.

I confirm that I have read this submission and believe that I have an appropriate level of expertise to confirm that it is of an acceptable scientific standard.

# Peer review: Reviewer response for version 1

**DOI:** 10.21956/gatesopenres.13951.r26740

**Published:** 2018-11-26

**Authors:** Jody R. Lori

**Affiliations:** 1 Department of Health Behavior and Biological Sciences, University of Michigan School of Nursing, Ann Arbor, MI, USA

Thank you for the opportunity to review the manuscript “Group versus individual antenatal and first year postpartum care: study protocol for a multi-country cluster randomized controlled trial in Kenya and Nigeria” by Kabue, MM et. al. The manuscript describes the research protocol of the first cluster randomized controlled trial of group antenatal care with care continuing to be provided in groups for the first year postpartum to women and their infants in Africa. This manuscript is well-written and clearly describes the protocol used to conduct the above trial. This detailed protocol will add to the literature on approaches to study group antenatal care in low resource settings and follow-up care utilizing groups for one-year postpartum. The authors present extensive background and rationale for the study by framing it within the context of the *WHO Recommendations on Antenatal Care for a Positive Pregnancy Experience* and citing what is known about group antenatal care from the literature. 

 The methods section provides appropriate detail on the study design and setting including power calculations and rationale. The stated outcomes are clear. Data collection and data quality assurance are clearly presented. All tables and figures are clear and do not repeat information provided in the text. Supplementary files provide additional details for ease in replication of the study. The Consolidated Standards of Reporting Trials guidelines will be used for data analysis, which is appropriate for this type of trial. Statistical analysis for both quantitative and qualitative results is appropriate. No results or conclusions are reported as this manuscript is a descriptive study protocol.

 It is not clear from the manuscript why five antenatal care visits were used for the intervention instead of the recommended eight visits in the 2016 *WHO Recommendations on Antenatal Care for a Positive Pregnancy Experience*. It would help the reader to understand why this deviation from the recommendations was made. It is also not completely clear if the sites randomized for the intervention (group care) exclusively provided care using the group model or if they continued to offer individual care as well. I believe all sites continued to offer individual care but I am not completely clear on this point from the details provided in the manuscript.  Finally, related to follow-up with study participants, the authors state “physical tracing was done” if phone numbers were not functional or they were unable to reach the women after five attempts. Additional details would help the reader understand how this was operationalized and allow replication.

 Overall this is a clear and precise manuscript outlining the details of a multi-country, multi-site trial of group antenatal and postpartum care in low-resource settings. I commend the authors for their detailed description of each step of the study allowing replication by other researchers and look forward to reading the results from their study.

I confirm that I have read this submission and believe that I have an appropriate level of expertise to confirm that it is of an acceptable scientific standard.
